# Catalyst-Controlled Regiodivergent C–H Olefination of Furanyl Carbamates through a Rational Approach

**DOI:** 10.1021/jacsau.6c00334

**Published:** 2026-05-28

**Authors:** François Richard, Morgan Languet, Cora Escande de Messières, Ahmed M. Zaitoun, Alexandre Dupas, Xacobe C. Cambeiro, Janine Cossy, Stellios Arseniyadis

**Affiliations:** † 4617 Queen Mary University of London, Department of Chemistry, Mile End Road, London E1 4NS, UK; ‡ 57474 ESPCI Paris − PSL, 10 Rue Vauquelin, Paris 75005, France; § 218219 University of Greenwich, School of Science, Central Ave, Chatham ME4 4TB, UK

**Keywords:** C−H activation, olefination, rhodium catalysis, catalyst control, regiodivergent, DFT, furan, pyrrole, thiophene

## Abstract

Selective functionalization of heteroarenes remains a central challenge in synthetic organic chemistry, given their widespread presence in natural products and pharmacologically active molecules. Although numerous strategies exist for the olefination of five-membered heteroarenes, few enable precise control over regioselectivity. Herein, we report a DFT-supported, catalyst-controlled C–H olefination of cyclic dienol carbamates that allows regioselective access to either C3- or C5-substituted heteroarenes. This strategy provides a rational and versatile platform for achieving positional selectivity in heteroarene functionalization.

## Graphical abstract

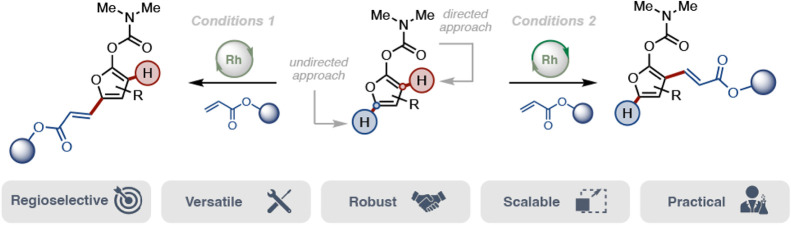

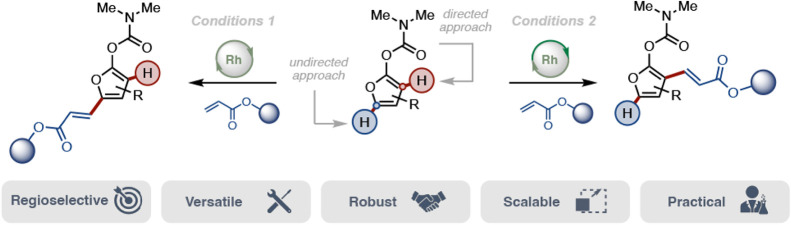

## Introduction

Metal-catalyzed C–H activation has emerged as an unparalleled strategy in modern synthetic chemistry, transforming inherently inert C–H bonds into versatile synthetic handles for functionalization. Extensive studies have demonstrated its applicability across aromatic, heteroaromatic, olefinic, and even saturated aliphatic substrates.^1–5^ Given the ubiquity of C–H bonds in organic molecules, these transformations provide a powerful platform for both early- and late-stage diversification, thereby enabling the rapid assembly of structurally diverse chemical libraries. Yet, this ubiquity renders regioselectivity a central challenge in the field. While directing groups and template strategies have provided powerful solutions for steering reactivity, the development of broadly applicable approaches that enable predictable and versatile site-selectivity remains a key frontier in C–H functionalization.^6–8^ However, the requirement for pre-functionalization imposes significant limitations on substrate scope and can become a liability when subsequent removal of the directing group is necessary to access the desired product. Moreover, targeting different C–H sites on the same scaffold often necessitates *de novo* syntheses to introduce new directing groups, thereby diminishing the efficiency and versatility that make C–H activation so attractive for chemical library generation. In response, traceless transient directing groups (TDGs) have emerged as a powerful alternative. In this strategy, the directing group engages the substrate through reversible binding, providing the necessary anchoring for selective C–H activation while ultimately leaving no trace in the final product.^9–11^ Nonetheless, TDG-based strategies are not universally applicable, which has led to a paradigm shift. Careful fine-tuning of the catalytic system and reaction conditions has slowly emerged as a powerful approach to access distinct C–H sites within the same substrate (Figure 1A). These regiodivergent processes are particularly appealing, as they enable rapid exploration of broad chemical space from a single molecular scaffold.^12^ This strategy has been most extensively explored in five-membered heteroaromatics, whose C–H bonds often display markedly distinct reactivities.^13–16^ A representative example is the regiodivergent olefination of pyrazoles reported by Lin, Baik, Joo and coworkers (Figure 1B).^17^ In this study, an electrophilic Pd catalyst is used in combination with 4,5-diazafluoren-9-one (4,5-DAF) and trifluoroacetic acid to selectively functionalize the more nucleophilic C4 position. In contrast, tuning the catalytic system with a mono-*N*-protected amino acid ligand under basic conditions exploited the enhanced acidity of the C5–H bond, thereby switching the regioselectivity. In parallel, Van Gemmeren and coworkers demonstrated that steric factors are equally critical in dictating selectivity. In their studies on the alkenylation of 3-substituted thiophenes (Figure 1C),^18^ increasing the steric demand of an amino-acid-derived ligand directed olefination to the sterically more accessible C5 position. Conversely, functionalization at the electronically favored C2 position was achieved by reducing ligand bulk and enhancing catalyst electrophilicity through installation of a trifluoroacetyl group on the ligand’s nitrogen. Together, these studies underscore how subtle tuning of steric and electronic factors within the catalytic system can be harnessed to dictate regioselectivity in C–H activation. Based on these studies and our long-standing interest in the reactivity of butenolide-type structures,^19–23^ we envisioned that a regiodivergent C–H alkenylation could be developed on the conjugated dienolate form of butenolides.

**Figure 1:**
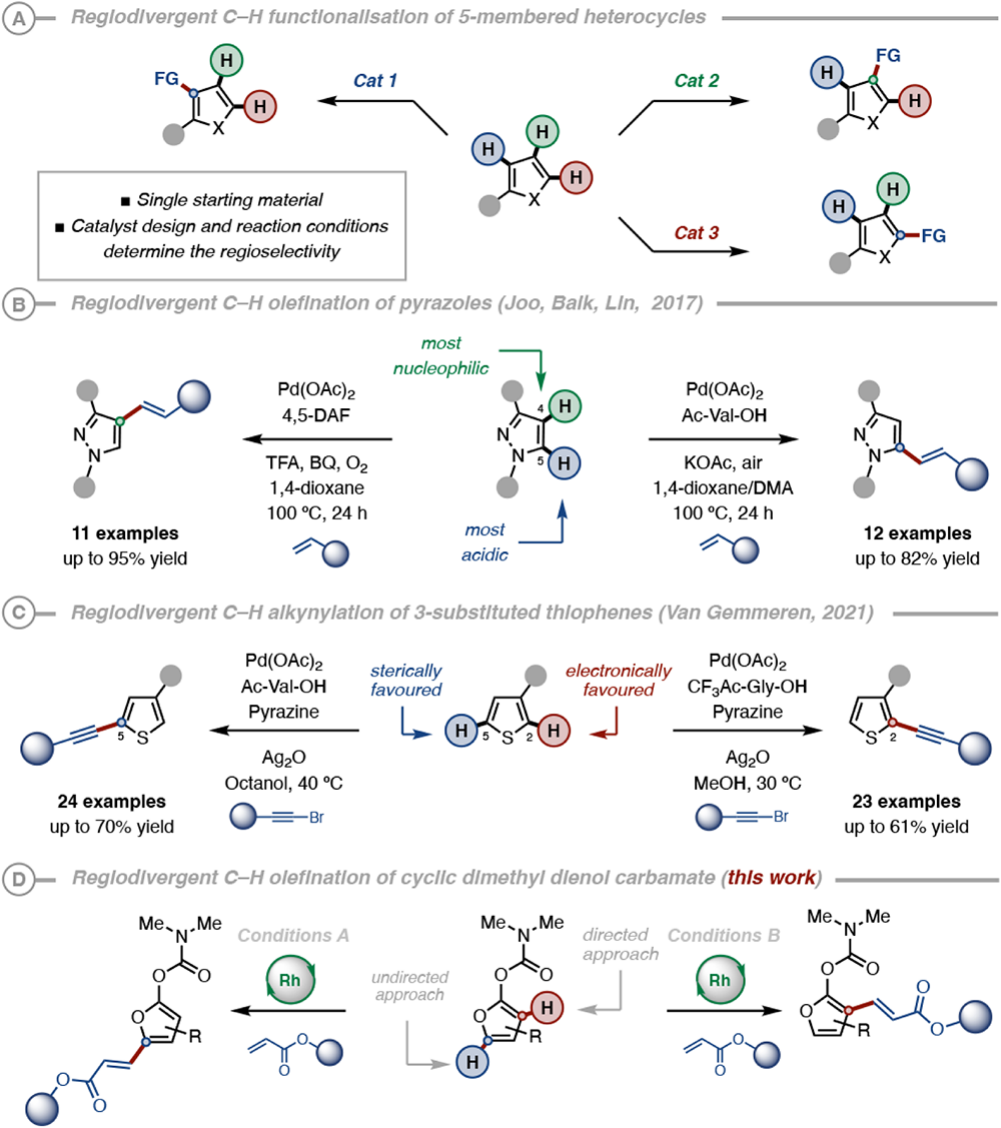
General methods for the selective functionalization of heteroarenes.

We hypothesized that the C3-functionalization could be achieved by exploiting the exocyclic oxygen atom to install a suitable directing group. Over the years, enol carbamates have been successfully applied in metal-catalyzed C–H functionalization,^24–27^ and therefore selected a cyclic dienol dimethylcarbamate as a model substrate for this study. Consistent with literature precedents,^25–27^ conditions generating a cationic, coordinatively unsaturated Rh catalyst were expected to promote coordination of the directing group and thus favor C3 functionalization. In contrast, conditions leading to neutral, coordinatively saturated Rh catalysts were expected to render the directing group inoperative and therefore favor the intrinsic C5 selectivity previously observed in the C–H olefination of furans (Figure 1D).^28–31^

## Results and Discussion

Based on the literature,^32,33^ we proposed a catalytic cycle (Figure 2A) involving C–H activation, alkene insertion, and β-hydride elimination to afford the olefinated product and a Rh­(III) hydride. Base-promoted reductive elimination, followed by reoxidation, would regenerate the Rh­(III) catalyst and close the cycle. Within this general mechanistic picture, substantial variation in catalyst speciation may arise depending on the reaction conditions and additives present in the mixture. Accordingly, the active Rh species could be either cationic or neutral and could bear a variety of anionic ligands such as chloride, acetate or carbonate.

We began by using density functional theory (DFT) to examine trends in C3 versus C5 regioselectivity in the C–H activation step for several plausible catalytic species. Rh-catalyzed C–H activation using [Rh­(Cp*)­Cl_2_]_2_ as pre-catalyst in combination with a Ag^+^ salt and Cu­(OAc)_2_ has been reported previously. Under these conditions, the Ag+ salt is proposed to abstract chloride ligands from Rh, generating cationic, coordinatively unsaturated active species,^25,32^ whereas Cu­(OAc)_2_ acts as both oxidant and source of acetate base/ligand.

A previous study on a closely related reaction between an enol dimethyl carbamate and acrylates showed that the C–H activation was rate-determining (*k*_*H*_ */k*_*D*_ = 5).^25^ Although alkene insertion has been proposed to be rate-determining in other related systems, we reasoned that the selectivity of the C–H activation step should nonetheless provide a reasonable proxy for the overall regioselectivity. Accordingly, we used DFT calculations to predict the regioselectivity of the C–H activation with cationic [Rh­(Cp*)­(OAc)]^+^ (**I**, Figure 2B). Calculations were carried out with the hybrid ωB97X-D functional,^34^ which includes dispersion corrections, using the SMD solvation model,^35^ the SDD basis set and effective core potential for Rh, and 6–31G­(d,p) for all other atoms, as implemented in Gaussian 16.^36^

We identified suitable transition states for both C3 and C5 C–H activation (**I-TS**_**C3**_ and **I-TS**_**C5**_, respectively; Figure 2C). Frequency analysis confirmed that each transition state had a single imaginary frequency corresponding to motion along the expected reaction coordinate. In addition, intrinsic reaction coordinate (IRC) calculations showed that each transition state connected the corresponding reactant and product minima (**I-R**_**C3**_, **I-R**_**C5**_, **I-P**_**C3**_, and **I-P**_**C5**_).

**Figure 2:**
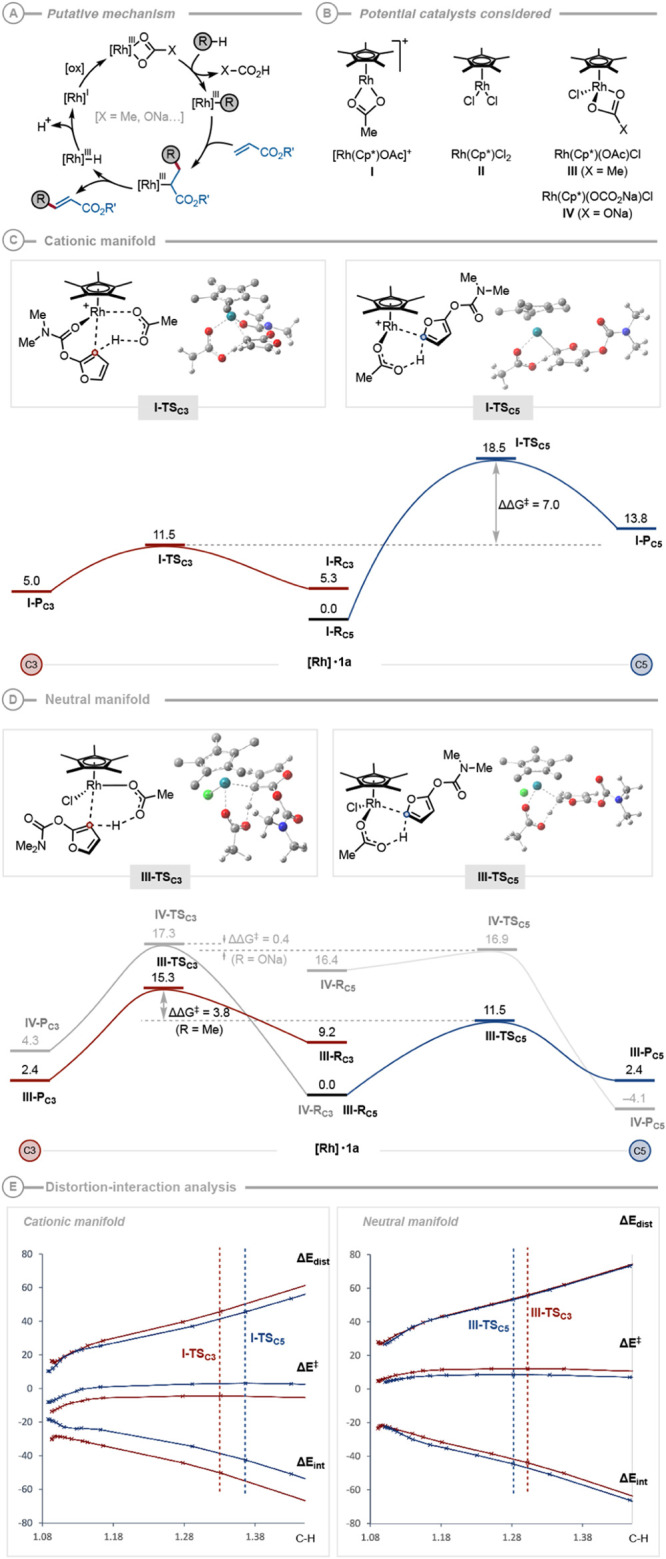
Accepted mechanism for Rh-catalyzed C–H alkenylations (**A**). Catalyst considered (**B**). Free energy profile of the C3 selective C–H olefination with cationic compound **I** (**C**). Free energy profile of the C5-selective C–H olefination with neutral acetate complex **III** (red and blue lines) and carbonate complex **IV** (grey lines) (**D**). Distortion-interaction analyses (**E**). Energy values in reaction profiles are Gibbs free energy in kcal/mol. Those in distortion-interaction plots are electronic potential energies in kcal/mol.

Both **I-TS**_**C3**_ and **I-TS**_**C5**_ involve concerted deprotonation of the C–H bond by the acetate ligand accompanied by C–Rh bond formation, consistent with carboxylate-assisted metalation.^37^ In both cases, the reactant minima show coordination of the substrate aromatic ring to Rh. For **I-R**_**C3**_, this is assisted by coordination of the carbamate directing group, while the acetate ligand is monodentate; for **I-R**_**C5**_, only furan coordination is observed, and the acetate ligand is bidentate. A similar pattern is found in the product minima: bidentate coordination of the substrate is maintained in **I-P**_**C3**_, whereas in **I-P**_**C5**_ the substrate remains monodentate. Direct comparison of the two regioisomeric transition states shows that C3 activation is favored by ΔΔG^‡^ = 7.0 kcal/mol. We therefore predicted that conditions employing an Ag^+^ additive would favor C3 selectivity.

Ag-free C–H olefination has been reported in a number of cases with Rh and other metals.^38^ Common additives in Ag^+^-free methods include Cu­(OAc)_2_, which serves as oxidant and acetate source, and Na_2_CO_3_ or K_2_CO_3_, which act as terminal base. Under such conditions, catalyst speciation is potentially more complex and is likely dominated by neutral species retaining at least one chloride ligand. We therefore considered Rh­(Cp*)­Cl_2_ (**II**), Rh­(Cp*)­(OAc)Cl (**III**), and Rh­(Cp*)­(OCO_2_Na)Cl (**IV**) as plausible active catalysts in this manifold (Figure 2B).

For dichloride complex **II**, we attempted to locate transition states for both a σ-bond metathesis pathway involving HCl extrusion and an S_E_Ar-type mechanism involving π-coordination of the furan followed by deprotonation by an external base. All such attempts were unsuccessful. In the absence of well-defined transition states, we instead carried out relaxed scans of the C–H bond distance as a proxy for the reaction coordinate. These scans indicated very high barriers to C–H bond cleavage, leading us to rule out dichloride **II** as a likely active catalyst.

For the acetate (**III**) and carbonate (**IV**) complexes, the system is considerably more complicated because of the presence of diastereomers (arising from a chiral center at Rh and planar chirality of the coordinated furan ring), in addition to rotational isomers about the Rh-furan bond. We therefore performed an exhaustive search with a slightly simplified model system (Cp in place of Cp*), systematically identifying all relevant minima and transition states (see SI for details). Catalytically relevant structures were then selected for reoptimization with the full Cp* system. In both cases, we identified transition states with geometries consistent with ligand-assisted metalation (**III-TS**_**C3**_ and **III-TS**_**C5**_ for acetate; **IV-TS**_**C3**_ and **IV-TS**_**C5**_ for carbonate). As in the cationic manifold, each transition state was characterized by a single imaginary frequency corresponding to motion along the expected reaction coordinate, and IRC calculations connected them to the corresponding reactants (**III-R**_**C3**_, **III-R**_**C5**_, **IV-R**_**C3**_, and **IV-R**_**C5**_) and products (**III-P**_**C3**_, **III-P**_**C5**_, **IV-P**_**C3**_, and **IV-P**_**C5**_).

The acetate pathway showed the lower overall activation energies relative to its lowest point (Figure 2D, colored traces) and favored C5 activation with ΔΔG^‡^ = 3.8 kcal/mol. The carbonate pathway (Figure 2D, gray traces) showed higher intrinsic barriers and also favored C5 activation, although only by ΔΔG^‡^ = 0.4 kcal/mol. Unfortunately, DFT does not allow the relative stabilities of **III** and **IV** to be determined unequivocally in the presence of both Cu­(OAc)_2_ and Na_2_CO_3_, and direct comparison with respect to a common reference is therefore not meaningful. The corresponding acetate/carbonate exchange is a solution-phase ion-metathesis equilibrium involving small charged species, counterions, and solvent-dependent ion pairing. Computational studies in homogeneous catalysis have shown that such ligand-exchange processes are often not described reliably by continuum solvation alone and are highly sensitive to small changes in the solvation model.^39^ In addition, the solubilities of Cu­(OAc)_2_ and Na_2_CO_3_ are expected to have a strong influence on this metathesis equilibrium. Whereas Cu­(OAc)_2_ is soluble in 1,4-dioxane at room temperature to at least 0.06 M,^40^ Na_2_CO_3_ is generally treated in the literature as essentially insoluble.^41^ These considerations, together with the experimental results discussed below, which show complete C5 selectivity, support the conclusion that **III** is a more plausible active catalyst than **IV**. We also identified additional transition states for the carbonate pathway corresponding to a σ-bond metathesis mechanism involving the Rh-bound oxygen atom of the carbonate ligand. This pathway likewise favored C5 activation (ΔΔG^‡^ = 5.6 kcal/mol), but its substantially higher barriers led us to rule it out as a viable mechanism (see SI for details). Thus, our computational study, without attempting to provide a complete mechanistic picture of the reaction, strongly supports C3-selectivity in the presence of Ag^+^ additive and C5 selectivity in its absence.

To rationalize the divergent predicted selectivities, we performed distortion-interaction analysis^42^ on the transition states of the cationic (**I-TS**_**C3**_ and **I-TS**_**C5**_) and neutral acetate (**III-TS**_**C3**_ and **III-TS**_**C5**_) manifolds. For each transition state, we selected twenty geometries along the calculated intrinsic reaction coordinate. At each point, the geometry was held fixed, the structure was partitioned into Rh catalyst and **1a** fragments, and single-point calculations were performed on each fragment. The interaction energy (E_int_) was defined as the difference between the energy of the parent structure and the sum of the energies of the two corresponding fragments. The distortion energy (E_dis_) was defined as the difference between the energies of these fragments and those of their respective ground-state optimized geometries. Figure 2E shows plots of E_dis_ and E_int_ against C–H bond length, used here as a proxy for the reaction coordinate, with the transition-state geometry marked by a vertical line. For both catalysts, the E_int_ is the principal factor underlying the observed selectivity. For cationic catalyst **I**, the C3 pathway shows a significantly more favorable interaction energy (presumably due to coordination of the directing group), partially offset by increased distortion. For neutral acetate catalyst **III**, the distortion energies are nearly identical, whereas the interaction energy favors C5 activation. This may reflect either steric hindrance from the substituent, which in this case is unable to act effectively as a directing group, or the more electron-rich character of the C5 position of the furan.

Following the conclusions from our calculations, we attempted to develop the C3-selective C–H olefination of our cyclic dienol carbamate. Running the reaction in DCE with *n-*butyl acrylate as the coupling partner using 2.5 mol% of [RhCp*Cl_2_]_2_, 2.1 equiv. of Ag­(OAc) as the oxidant, and 10 mol% of AgSbF_6_ to form a cationic Rh species, afforded the desired C3-olefinated product **2a** in 30% yield (Figure 3A, entry 1). After a thorough optimization of the reaction parameters and the nature of the directing group (see SI for full details), the C3-olefinated product could be obtained in 77% yield when using 5 equiv. of acrylate and Cu­(OAc)_2_ as the oxidant (Figure 3A, entry 2). The yield could be further increased to 91% by running the reaction using 10 equiv. of the acrylate (Figure 3A, entry 3). Notably, the C5-olefinated product was not observed under any of the initial conditions screened.

**Figure 3:**
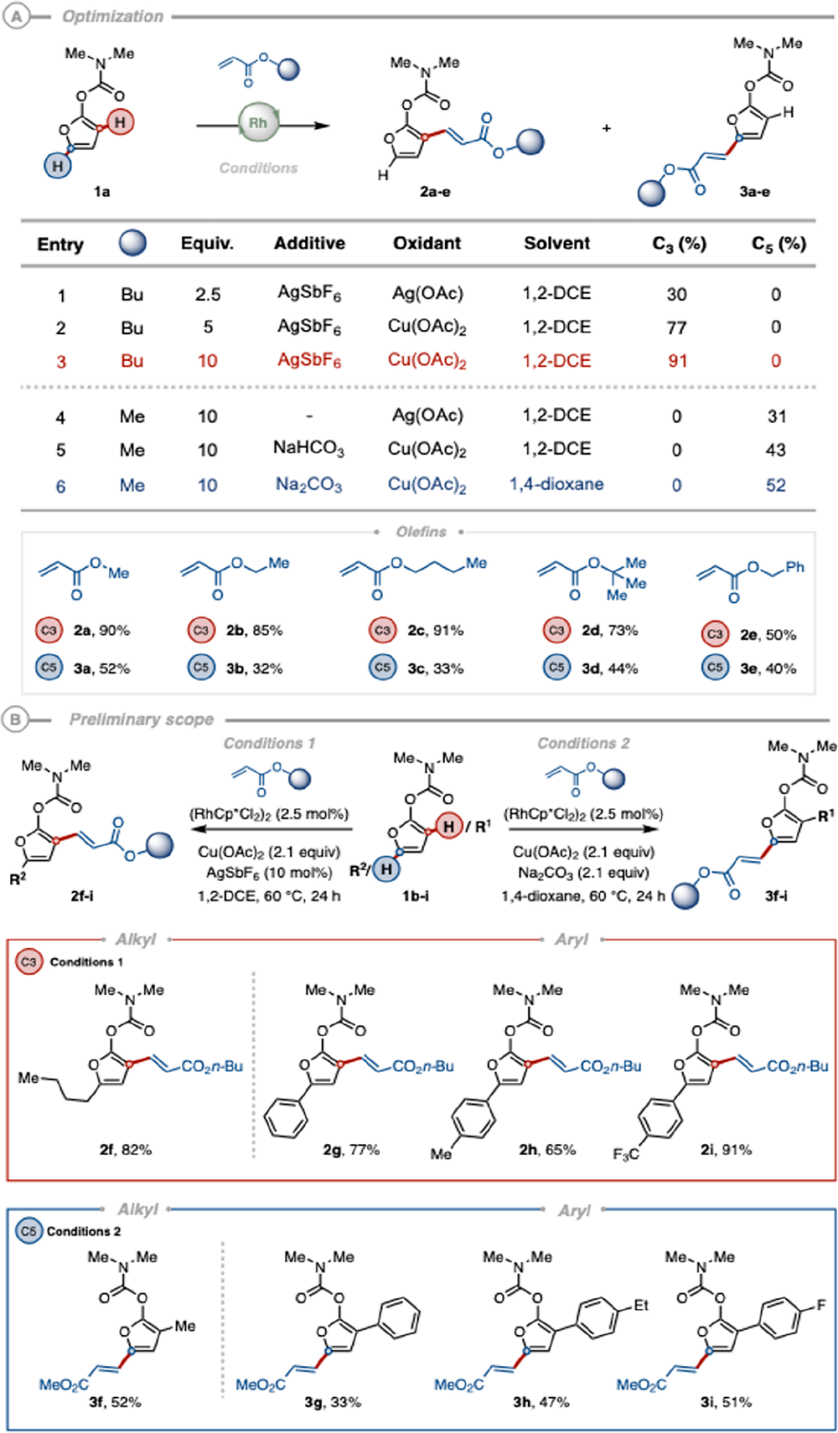
Systematic study (**A**). Initial substrate scope (**B**).

To reverse the regioselectivity in favor of C5 functionalization, AgSbF_6_ was excluded from the reaction while the large excess of methyl acrylate (10 equiv.) was retained. Under these modified conditions, the C5-olefinated product **3a** was obtained exclusively in 31% yield (Figure 3A, entry 4), with no detectable C3 isomer. The addition of NaHCO_3_ improved the yield to 43% (Figure 3A, entry 5). Assessment of different bases, oxidants, solvents and temperatures (see SI for full details) led to the identification of Na_2_CO_3_ and 1,4-dioxane as the optimal base and solvent for this transformation affording **3a** in 52% yield (Figure 3A, entry 6).

With both sets of conditions in hand, we next explored the reaction scope with respect to various acrylate coupling partners (Figure 3A). Methyl acrylate afforded high yields for both C3-olefination (**2a**, 90%) and C5-olefination (**3a**, 52%). In contrast, the use of ethyl acrylate and *n*-butyl acrylate resulted in a notable decrease in C5-olefination efficiency, affording products **3b** and **3c** in 32 and 33% yield, respectively. Interestingly, bulkier acrylate partners such as *tert*-butyl acrylate (**3d**, 44%) and benzyl acrylate (**3e**, 40%) provided improved C5-olefination yields, although the corresponding C3-olefination products **2d** and **2e** were obtained in slightly lower yields (73 and 50%, respectively). While complete regioselectivity was maintained in all cases, these results suggest that increased steric bulk in the acrylate partner disfavors C3-functionalization and enhances the efficiency of the C5-olefination.

Evaluation of the influence of ring substituents was subsequently undertaken (Figure 3B). In the case of alkyl groups, both C3 and C5 olefinations proceeded efficiently. Hence, the C3-olefination of 5-butyl furanyl carbamate delivered the desired product **2f** in 82% yield, while the C5-olefination of the 3-methyl furanyl carbamate derivative led to **3f** in 52% yield. Building on this, the impact of more diverse and functionally varied aryl substituents was next explored. Interestingly, incorporation of a phenyl group at the C5-position had minimal effect on the C3-olefination, affording product **2g** in 77% yield. In contrast, the introduction of an aryl substituent at the C3-position significantly diminished the efficiency of the C5-olefination, as **3g** was obtained in only 33% yield. Notably, the presence of a small alkyl group on the aromatic ring, such as a *p*-methyl or a *p*-ethyl, reduced the yield of the C3-olefination but improved the C5-olefination. Notably, a marked increase in C3-olefination efficiency was observed with the electron-deficient 5-(trifluoromethyl)­phenyl-substituted substrate **2i** (91%). Similarly, C5-olefination of 3­(4-fluorophenyl)­furanyl carbamate **1i** proceeded in a respectable 51% yield (**3i**).

In this preliminary scope evaluation, the C3-olefination consistently outperformed the C5-olefination, generally affording products in higher yields. Encouraged by its robustness, we further investigated the substrate scope of the C3-olefination (Figure 4). Gratifyingly, the reaction proceeded efficiently across a broad range of substrates. For the C4- and C5-alkyl derivatives (**1j-m**), good to excellent yields were obtained ranging from 61 to 91%.

**Figure 4:**
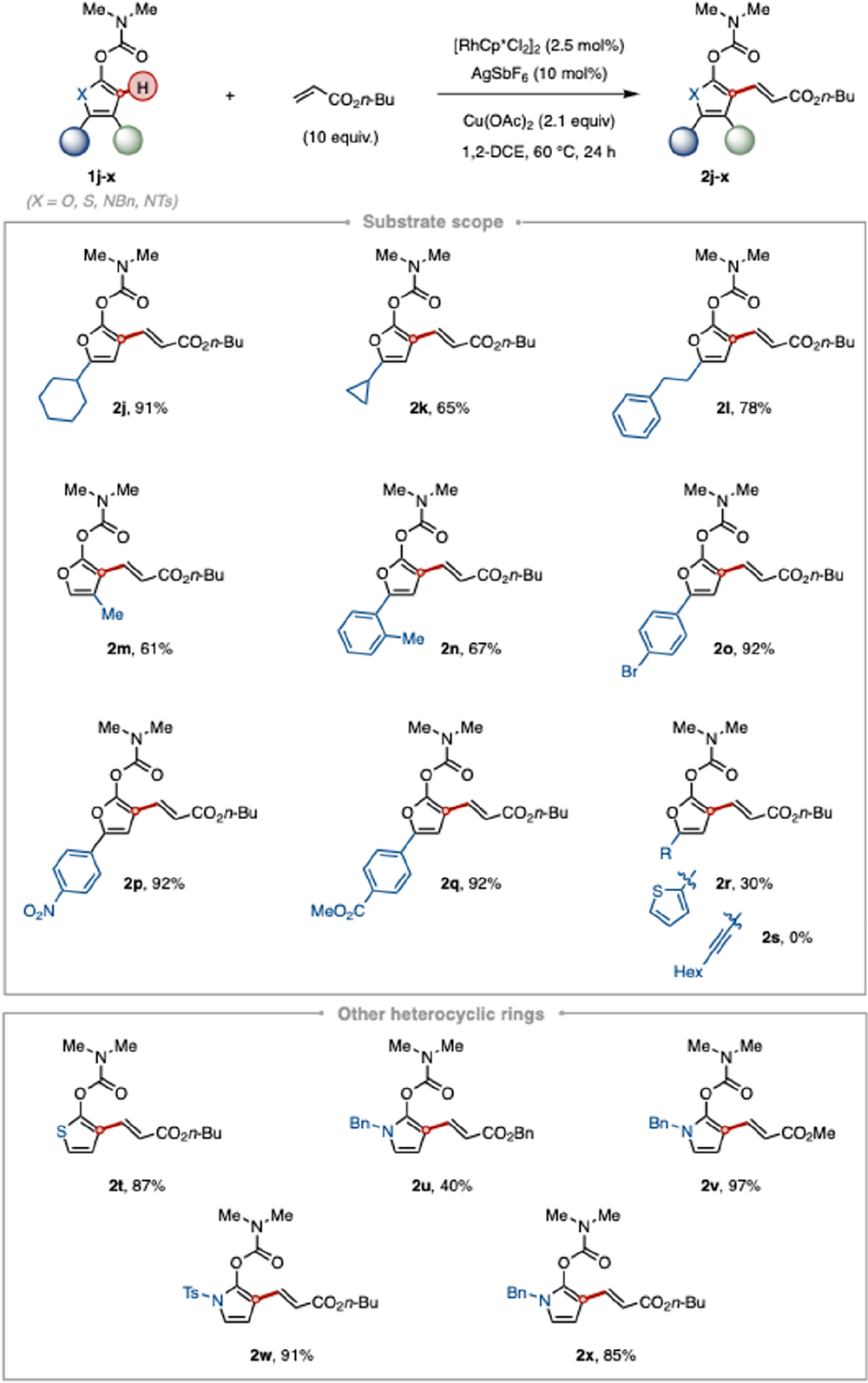
C3-selective C–H olefination – Substrate scope. All reactions were run on a 0.2 mmol scale under N2 atmosphere with dry 1,2-dichloroethane. Yields of isolated products are given.

The reaction was further evaluated on furanyl carbamates bearing aromatic substituents at the C5 position. As previously noted, the C3-olefinated product was obtained in 77% yield for the 5-phenyl-substituted substrate **2g**, with a slight decrease to 65% for the 5­(4-methylphenyl) analogue **2h**. No further drop in yield was observed when the substituent was brought closer to the furanyl ring, as in the case of 5­(*o-*tolyl) furanyl carbamate (**2n**). Consistent with trends observed in the preliminary scope evaluation (*e.g*., CF_3_-substituted **2i**), electron-withdrawing aryl groups such as 4-nitrophenyl (**2p**) and 4-methylbenzoate (**2q**) significantly enhanced reactivity, delivering the desired products in 92% yield. These groups likely reduce the electron density of the butenolide ring, thereby lowering the pK_a_ of the α-C–H bond and accelerating the C–H olefination. The reaction also demonstrated tolerance to synthetically versatile functionalities. For example, 5-(4-bromophenyl)­furanyl carbamate afforded product **2o** in 92% yield. In contrast, its analogue bearing a thiophene ring at C5 (**2r**) was only obtained in 30% yield. This reduced efficiency may arise from bis-coordination of the Rh catalyst to both the sulfur atom of thiophene and the inner oxygen of the butenolide core, leading to catalyst deactivation. A similar effect likely accounts for the complete loss of reactivity observed with 5-hexynyl-substituted furanyl carbamate **1s**, possibly due to strong coordination of the alkyne moiety. Pleasingly, the C3-olefination protocol was successfully extended to sulfur- (**2t**) and nitrogen-containing (**2u-x**) heteroarenes, with no significant loss in efficiency. Both classes of substrates were well tolerated, delivering the corresponding thiophene (**2t**, 87%) and pyrrole derivatives (**2u-x**, 41–97%) in good to excellent yields.

The reaction also proved to be scalable, as demonstrated by a 10 mmol-scale experiment that delivered the desired product **2c** in 75% yield (Figure 5A).

**Figure 5:**
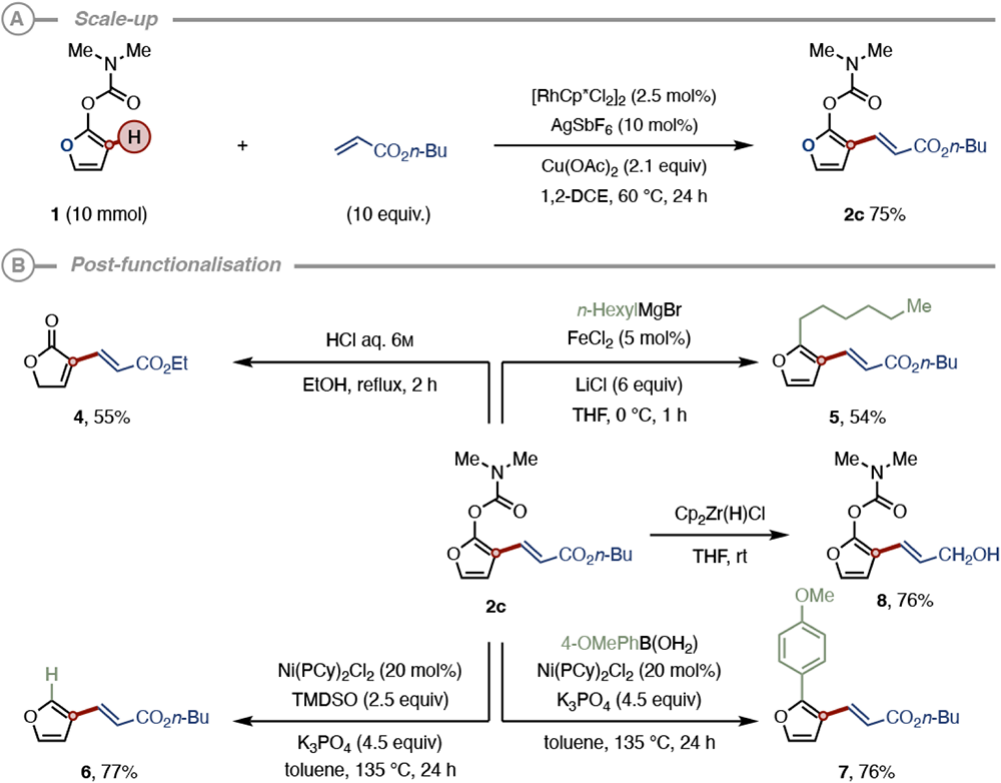
Scale-up and post-functionalizations.

With the broad substrate scope established, we next sought to demonstrate the synthetic utility of the C3-olefination protocol (Figure 5B). Hence, cleavage of the carbamate directing group under acidic conditions in refluxing ethanol furnished the corresponding butenolide **4** in 55% yield. Beyond serving as a directing group, the dimethyl carbamate also proved to be a versatile synthetic handle for further functionalization of the furan core. For example, Fe-catalyzed cross-coupling of carbamate **2c** with *n-*hexylmagnesium bromide, following the procedure reported by Shi and coworkers, delivered 2,3-disubstituted furan **5** in 54% yield.^43^ Ni-catalyzed cross-coupling reactions were also effective: 1,1,3,3-tetramethyldisiloxane (TMDSO)-mediated reductive cleavage afforded the corresponding 3-substituted furan **6** in 77% yield, while Suzuki-Miyaura coupling with 4-methoxyphenylboronic acid yielded the 2,3-disubstituted product **7** in 76% yield.^44,45^ Finally, Cp_2_Zr­(H)­Cl-mediated reduction of 2c, led to the corresponding allylic alcohol **8** in 76% yield.

## Conclusion

In summary, a DFT-supported strategy enabled the development of two complementary sets of reaction conditions for the selective C3- or C5-functionalization of heteroarenes *via* catalyst-controlled, regiodivergent C–H olefination. The method appears to be especially effective for the C3-olefination, offering a broad substrate scope, tolerating both electron-donating and electron-withdrawing substituents, and showing compatibility with various acrylate coupling partners. The reaction was successfully scaled to a synthetically useful 10 mmol scale, and the resulting products were readily transformed into structurally diverse derivatives, underscoring the utility and versatility of this approach. Further applications to other heteroarenes are currently under investigation.

## Supplementary Material



## Data Availability

Complete set of original Gaussian output files is fully accessible at the IoChemBD database (DOI: 10.19061/iochem-bd-6-361).

## Notes

Details of molecular simulation protocol, synthetic procedures, and characterization data (PDF)
