# Supplementary material for: Catalyst-Controlled Regiodivergent C–H Olefination of Furanyl Carbamates through a Rational Approach
Source: JACS Au. 2026 May 28;6(6):3294–300. doi: 10.1021/jacsau.6c00334 (PMC13291970; doi:10.1021/jacsau.6c00334)
Supplement: Supplementary file 1 [file au6c00334_si_001.pdf]

Supporting Information

Catalyst-controlled Regiodivergent C–H Olefination of Furanyl Carbamates through a Rational Approach

Francois Richard,[†,#] Morgan Languet,[§,#] Cora Escande de Messières,[†] Ahmed M. Zaitoun,[‡] Alexandre Dupas,[§] Xacobe C. Cambeiro,[‡] Janine Cossy,*[,§] and Stellios Arseniyadis*[,†]

[†] *Queen Mary University of London, Department of Chemistry, Mile End Road, E1 4NS, London, UK*

[§] *ESPCI Paris – PSL, 10 Rue Vauquelin, 75005 Paris, France*

[‡] *University of Greenwich, School of Science, Central Ave, ME4 4TB, Chatham, UK*

Table of contents

**1.**	**General methods**	**S2**
**2.**	**Optimization**	**S3**
	a. C3-olefination	S3
	b. C5-olefination	S5
**3.**	**Synthesis of starting materials**	**S8**
	a. Synthesis of the dienol carbamates	S8
	b. Synthesis of starting furanones and lactams	S26
**4.**	**C3-olefination**	**S42**
	a. General procedure for the C3-olefination:	S42
	b. Characterization data	S43
**5.**	**C5-olefination**	**S62**
	a. General procedure	S62
	b. Characterization data	S62
**6.**	**Post-functionalisation**	**S70**
**7.**	**DFT calculations**	**S75**
**8.**	**Distortion-interaction analyses**	**S121**
**9.**	**NMR data**	**S127**
**10.**	**References**	**S200**

1. General methods

Reactions were conducted under a positive pressure of dry nitrogen or argon in oven-dried or flame-dried glassware, and at ambient rt, unless specified otherwise. Anhydrous solvents were either obtained from commercial sources or dried with a MBRAUN Solvent Purification System SPS-800. Petroleum ether refers to the 40-60˚C boiling fraction. Commercially available chemicals were purchased from Sigma Aldrich, Alfa Aesar, Fluorochem, TCI or Acros Organics and used as received unless stated otherwise. Microwave experiments were conducted in sealed reaction vessels, in a Monowave 400 reactor from Anton Paar. Evaporations were carried out on Büchi rotavapors under reduced pressure with a bath temperature of 35-40˚C using a dry ice condenser. Further drying of pure compounds was ensured by exposure to high vacuum for a few hours. Analytical TLC was performed with Merck silica gel plates, pre-coated with silica gel 60 F254 (0.2 mm). Visualization was affected by quenching of UV fluorescence (λ_{max} = 254 nm or 360 nm) and by staining with p-anisaldehyde, potassium permanganate or vanillin TLC stain solutions, followed by heating. Flash column chromatography employed VWR (230-400 mesh) silica gel. NMR spectra were recorded at 298 K using a Bruker AVANCE 400 spectrometer. ^{1}H NMR spectra were recorded at 400 MHz and residual solvent peaks were used as an internal reference ($CHCl_3$ δ 7.26). Data are reported as follows: chemical shift in ppm, multiplicity (s = singlet, brs = broad singlet, d = doublet, t = triplet, q = quartet, m = multiplet or overlap of non-equivalent resonances), coupling constants, and integration. ^{13}C NMR spectra were recorded at 101 MHz and residual solvent peaks were used as an internal reference ($CHCl_3$ δ 77.16). Data are reported as follows: chemical shift in ppm, multiplicity deduced from DEPT experiments (CH_3, CH_2, CH and Cq). The assignment of ^{1}H and ^{13}C signals was assisted by COSY, HSQC and HMBC experiments where necessary. IR spectra were recorded on a Perkin Elmer Spectrum 65 FT-IR spectrometer and are reported in frequency of absorption at the peak maximum (cm^{-1}). Low-resolution mass spectra were recorded on an Agilent 1100 series LC-MS (with a 6310 ion trap) under electrospray ionisation (ESI). High resolution mass spectra were recorded on a Waters SYNAPT G2-Si High Definition Mass Spectrometry system equipped with an Acquity UPLC BEH C18 column (2.1 x 50 mm; 130 Å) using a solvent gradient (0 >100% Acetonitrile in Water + 0.1% Formic acid) in positive electrospray ionisation (ESI+) mode. The instrument was tuned using a Leucin Enkephalin mix to optimum resolution and signal intensity and was calibrated using a Waters Major Mix IMS/ToF in a range of m/z 50-1200.

2. Optimisation

a. C3-olefination

i. Screening of directing groups

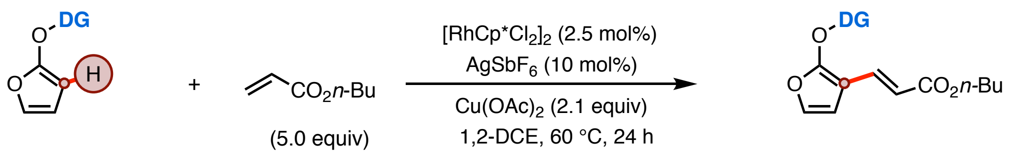

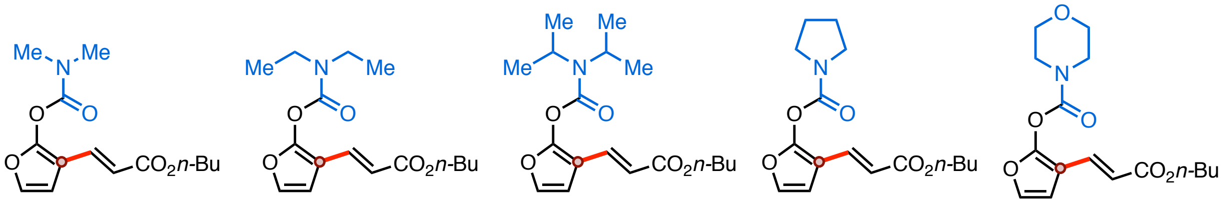

78% 61% 52% 71% 70%

ii. Reaction parameters optimization

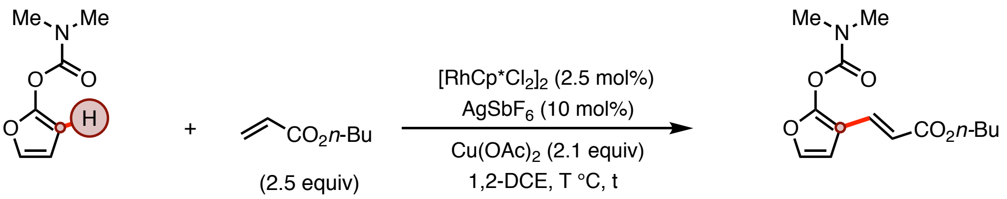

Entry	T (°C)	t (h)	Yield (%)
**1**	**60**	**24**	**55**
2	85	24	36
3	120	24	10
4	60	48	45

iii. Reaction conditions optimization

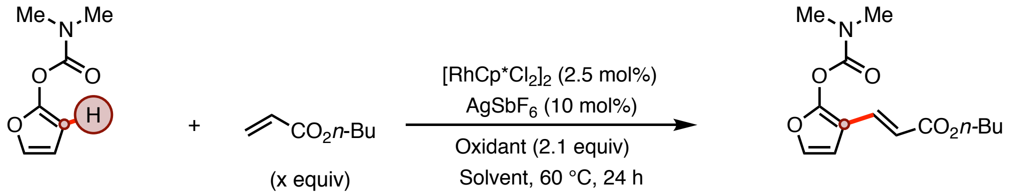

Entry	Oxidant	x (equiv.)	Solvent	Yield (%)
1	$Cu(OAc)_2$	2.5	MeOH	<5
2	$Cu(OAc)_2$	2.5	*t*-amylOH	<5
3	$Cu(OAc)_2$	2.5	HFIP	<5
4	$Cu(OAc)_2$	2.5	toluene	<5
5	$Cu(OAc)_2$	2.5	chlorobenzene	37
6	$Cu(OAc)_2$	2.5	Hexafluorobenzene	23
7	$Cu(OAc)_2$	2.5	1,4-dioxane	26
8	$Cu(OAc)_2$	2.5	THF	40
9	$Cu(OAc)_2$	2.5	MeCN	<5
10	$Cu(OAc)_2$	2.5	DMF	25
11	$Cu(OAc)_2$	2.5	DME	47
12	$Cu(OAc)_2$	2.5	1,2-DCE	55
13	$Cu(OAc)_2$	5	1,2-DCE	78
14	Ag(OAc)	5	1,2-DCE	30
15	$K_2S_2O_8$	5	1,2-DCE	21
16	$Cu(OTf)_2$	5	1,2-DCE	0
17	PIDA	5	1,2-DCE	0
**18**	**$Cu(OAc)_2$**	**10**	**1,2-DCE**	**91**

b. C5-olefination

i. Ligands screening

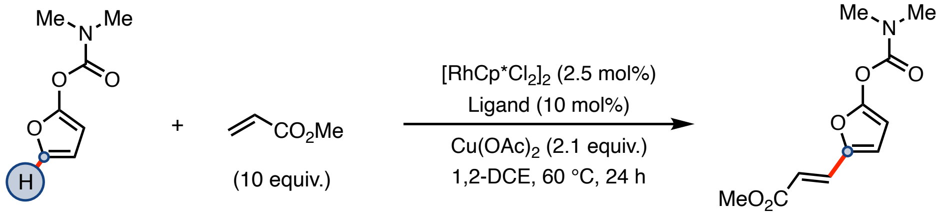

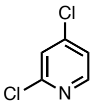

L1, 15%

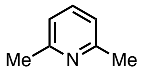

L2, 18%

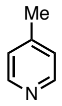

L3, 17%

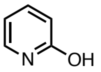

L4, 14%

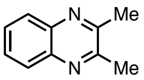

L5, 31%

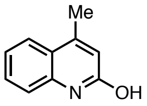

L6, 22%

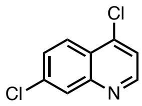

L7, 22%

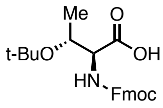

L8, 5%

ii. Oxidant and base screening

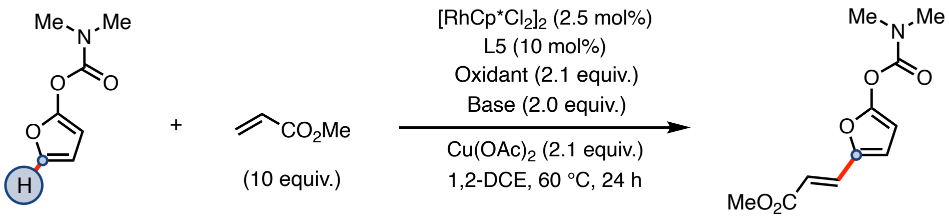

Entry	Oxidant	Base	Yield (%)
1	$Cu(OAc)_2$	-	31
2	Ag_2CO_3	-	0
3	Ag_2O	-	10
4	Ag(OAc)	-	10
5	$Zn(OTf)_2$	-	12
6	$Cu(OAc)_2$	CsOAc	34
7	$Cu(OAc)_2$	Na_2CO_3	39
**8**	**$Cu(OAc)_2$**	**$NaHCO_3$**	**43**
9	$Cu(OAc)_2$	K_2CO_3	24
10	$Cu(OAc)_2$	Li_2CO_3	22

iii. Solvent screening

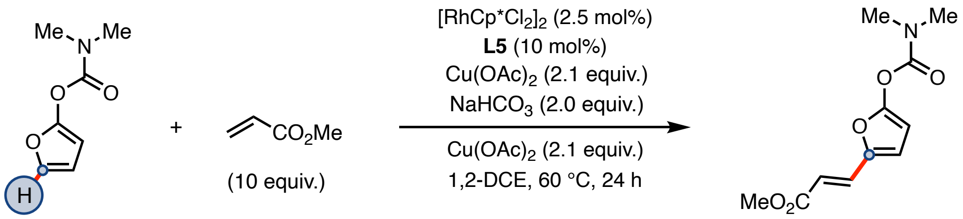

Entry	Oxidant	Yield (%)
1	1,2-DCE	43
2	toluene	17
3	CH_2Cl_2	18
4	DMSO	*traces*
5	MeOH	0
6	TFE	6
7	*t*-BuOH	14
8	*t*-amylOH	41
9	DME	15
**10**	**1,4-dioxane**	**51**

iv. Final optimization

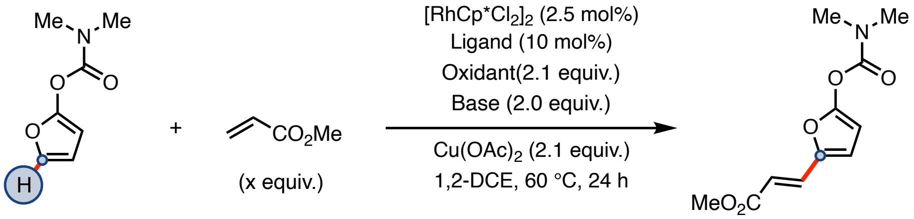

Entry	X (equiv.)	Ligand	Oxidant	Base	Yield (%)
1	10	L5	$Cu(OAc)_2$	$NaHCO_3$	51
2	10	L5	-	$NaHCO_3$	7
**3**	**10**	**-**	**$Cu(OAc)_2$**	**$NaHCO_3$**	**51**
4	5	-	$Cu(OAc)_2$	Na_2CO_3	39
5	5	-	benzoquinone	Na_2CO_3	24
6	5	-	$Cu(OAc)_2$, V_2O_5	Na_2CO_3	34
7	5	-	$CuCl_2$, V_2O_5	Na_2CO_3	39

v. Optimization for C3-methyl substrate

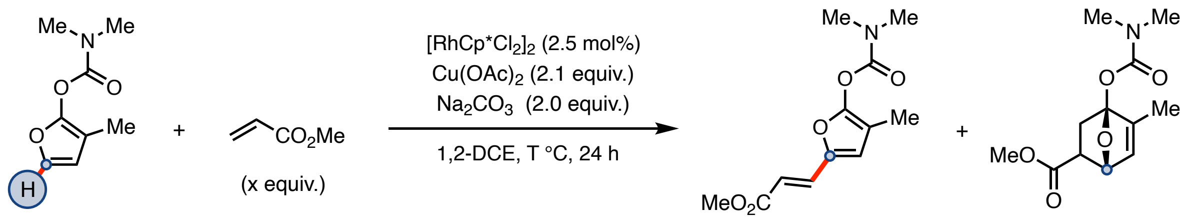

Entry	X (equiv.)	Base	T (°C)	Yield (%)	Yield (%)
1	10	$NaHCO_3$	60	30	55
2	5	$NaHCO_3$	60	37	37
3	3	$NaHCO_3$	60	28	18
4	3	Na_2CO_3	60	37	12
5	5	Na_2CO_3	40	37	0
**6**[a]	**5**	**Na_2CO_3**	**40**	**50**	**0**

[a]Reaction run for 120 h.

3. Synthesis of starting materials

a. Synthesis of the dienol carbamates

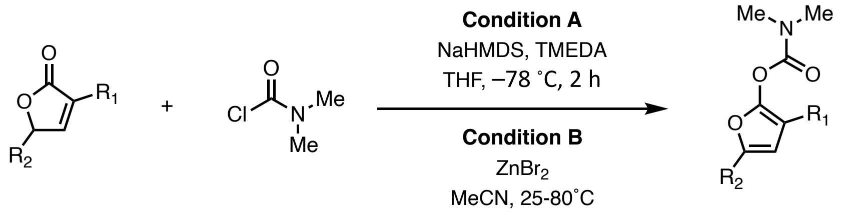

General procedure A: *Adapted from literature procedure*.[1] To a solution of anhydrous THF (0.1 M) was added NaHMDS (1.0 M in THF, 1.2 equiv.) and TMEDA (3.0 equiv.). The mixture was cooled to –78 °C and a solution of the desired heterocycle (1.0 equiv.) in anhydrous THF was slowly added to the reaction mixture. After stirring for 1H at –78 °C, the corresponding acyl chloride (5.0 equiv.) was added, and after 15 min at this temperature, the reaction mixture was slowly warmed to rt and stirred for an additional 2 h. A saturated aqueous solution of NH_4Cl was then added and the aqueous phase was extracted 3 times with EtOAc. The combined organic phases were washed 3 times with H_2O then with brine, dried over anhydrous $MgSO_4$, filtered and concentrated under vacuum to afford a crude residue, which was purified by flash column chromatography over silica gel to afford the corresponding dienol carbamate.

General procedure B: To a solution of furanone (1.00 equiv.) in acetonitrile (c = 0.25 M) was added triethylamine (2.50 equiv.) at rt. Aside, zinc bromide (2.00 equiv.) was activated by heating under vacuum until melting. After cooling to rt, acetonitrile (25 mL) and dimethylcarbamyl chloride (2.00 equiv.) were added to the activated zinc bromide. The white suspension was stirred for 5 min at rt. The furanone solution was then added dropwise to the zinc bromide suspension. The resulting mixture was stirred at the appropriate temperature overnight. After completion, the reaction mixture was concentrated under vacuum, and the residue was dissolved in EtOAc. Water was added and the phases were separated. The aqueous layer was extracted three times with EtOAc and the combined organic phase was washed with a saturated aqueous solution of brine, dried over anhydrous $MgSO_4$ and concentrated under vacuum to afford a crude red oil which was purified by flash column chromatography over silica gel.

Furan-2-yl dimethylcarbamate (1a)

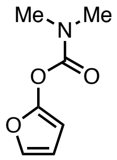

MW (g/mol): 155.15 **Molecular formula:** $C_7H_9NO_3$

Synthesized according to ***general procedure A***. The desired product was obtained as a yellow oil (190 mg, 1.23 mmol, 41% yield) after purification by flash column chromatography over silica gel (*n*-Hexane/Ethyl acetate = 90:10). When using ***general procedure B,*** the product is obtained in 85% yield.

^{1}H NMR (400 MHz, CDCl$_3$) δ 7.04 (dd, *J* = 2.2, 1.1 Hz, 1H), 6.33 (dd, *J* = 3.3, 2.2 Hz, 1H), 5.79 (dd, *J* = 3.3, 1.1 Hz, 1H), 3.07 (s, 3H), 2.99 (s, 3H).

^{13}C NMR (101 MHz, CDCl$_3$) δ 152.7, 151.8, 135.7, 111.2, 92.5, 37.0, 36.6.

HRMS (ESI): Calculated for $C_7H_9NO_3Na$ [M+Na$^+$]: 178.04746 found: 178.04760.

IR (neat): 2939, 1738, 1617, 1488, 1398, 1371, 1237, 1222, 1142, 1065, 998, 945, 838, 787, 718, 651, 600 cm^{-1}.

Furan-2-yl diethylcarbamate

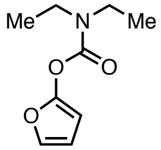

MW (g/mol): 183.21 **Molecular formula:** $C_9H_{13}NO_3$

Synthesized according to ***general procedure A***. The desired product was obtained as a yellow oil (198 mg, 1.08 mmol, 36% yield) after purification by flash column chromatography over silica gel (*n*-Hexane/Ethyl acetate = 90:10).

^{1}H NMR (400 MHz, CDCl$_3$) δ 7.04 (dd, *J* = 2.3, 1.1 Hz, 1H), 6.33 (dd, *J* = 3.3, 2.1 Hz, 1H), 5.79 (dd, *J* = 3.3, 1.1 Hz, 1H), 3.37 (q, *J* = 7.1 Hz, 4H), 1.24-1.17 (m, 6H).

^{13}C NMR (101 MHz, CDCl$_3$) δ 152.1, 151.9, 135.6, 111.2, 92.4, 42.7, 42.2, 14.1, 13.2.

HRMS (ESI): Calculated for $C_9H_{13}NO_3Na$ [M+Na$^+$]: 206.07876 found: 206.07882.

IR (neat): 2978, 1734, 1617, 1515, 1474, 1420, 1380, 1272, 1238, 1214, 1143, 1067, 1036, 1008, 965, 920, 880, 785, 716, 600 cm^{-1}.

Furan-2-yl diisopropylcarbamate

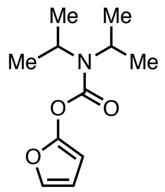

MW (g/mol): 211.26 **Molecular formula:** $C_{11}H_{17}NO_3$

Synthesized according to ***general procedure A***. The desired product was obtained as a yellow oil (285 mg, 1.35 mmol, 45% yield) after purification by flash column chromatography over silica gel (*n*-Hexane/Ethyl acetate = 90:10).

^{1}H NMR (400 MHz, CDCl$_3$) δ 6.98 (dd, *J* = 2.2, 1.0 Hz, 1H), 6.26 (dd, *J* = 2.2, 3.2 Hz, 1H), 5.72 (dd, *J* = 3.2, 1.0 Hz, 1H), 3.98 (q, *J* = 7.0 Hz, 2H), 1.29 (t, *J* = 5.8 Hz, 12H).
^{13}C NMR (101 MHz, CDCl$_3$) δ 151.8, 151.5, 135.6, 111.1, 92.5, 47.2, 46.8, 21.4, 20.3.
HRMS (ESI): Calculated for $C_{11}H_{17}NO_3Na$ [M+Na$^+$]: 234.11006 found: 234.11022.
IR (neat): 2966, 1731, 1614, 1511, 1428, 1372, 1306, 1236, 1217, 1206, 1158, 1128, 1064, 1037, 976, 937, 888, 819, 726, 601 cm^{-1}.

Furan-2-yl pyrrolidine-1-carboxylate

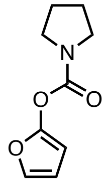

MW (g/mol): 181.19 **Molecular formula:** $C_9H_{11}NO_3$

Synthesized according to ***general procedure A***. The desired product was obtained as a yellow oil (239 mg, 1.32 mmol, 44% yield) after purification by flash column chromatography over silica gel (*n*-Hexane/Ethyl acetate = 90:10).

^{1}H NMR (400 MHz, CDCl$_3$) δ 6.86 (dd, *J* = 2.1, 1.1 Hz, 1H), 6.16 (dd, *J* = 3.3, 2.1 Hz, 1H), 6.04 (dd, *J* = 3.3, 1.1 Hz, 1H), 3.18 (t, *J* = 6.8 Hz, 2H), 3.07 (t, *J* = 6.8 Hz, 2H), 1.20-1.14 (m, 4H).

^{13}C NMR (101 MHz, CDCl$_3$) δ 151.7, 150.6, 135.3, 111.0, 92.1, 46.6, 46.4, 25.6, 24.7.

HRMS (ESI): Calculated for $C_9H_{11}NO_3Na$ [M+Na$^+$]: 204.06311 found: 204. 06315.

IR (neat): 2977, 2882, 1742, 1617, 1514, 1407, 1371, 1341, 1240, 1222, 1173, 1049, 1005, 944, 858, 721 cm^{-1}.

Furan-2-yl morpholine-4-carboxylate

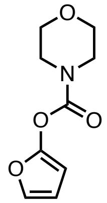

MW (g/mol): 197.19 **Molecular formula:** $C_9H_{11}NO_4$

Synthesized according to ***general procedure A***. The desired product was obtained as a yellow oil (414 mg, 2.10 mmol, 70% yield) after purification by flash column chromatography over silica gel (*n*-Hexane/Ethyl acetate = 90:10 to 75:25).

^{1}H NMR (400 MHz, CDCl$_3$) δ 7.03 (dd, *J* = 2.2, 1.1 Hz, 1H), 6.32 (dd, *J* = 3.3, 2.2 Hz, 1H), 5.79 (dd, *J* = 3.3, 1.1 Hz, 1H), 3.68 (s, 4H), 3.55 (dt, *J* = 29.5, 4.7 Hz, 4H).

^{13}C NMR (101 MHz, CDCl$_3$) δ 151.32, 151.27, 135.7, 111.2, 92.6, 66.4, 66.2, 44.9, 44.3.

HRMS (ESI): Calculated for $C_9H_{11}NO_4Na$ [M+Na$^+$]: 220.05803 found: 220.05799.

IR (neat): 3675, 3128, 2987, 2926, 2859, 1747, 1617, 1510, 1427, 1377, 1277, 1228, 1207, 1116, 1038, 976, 941, 850, 828, 730, 600 cm^{-1}.

5-Butylfuran-2-yl dimethylcarbamate (1b)

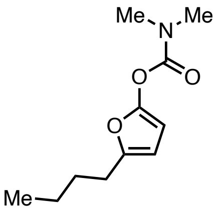

MW (g/mol): 211.26 **Molecular formula:** $C_{11}H_{17}NO_3$

Synthesized according to ***general procedure A***. The desired product was obtained as a yellow oil (336 mg, 1.59 mmol, 53% yield) after purification by flash column chromatography over silica gel (*n*-Hexane/Ethyl acetate = 90:10).

^{1}H NMR (400 MHz, $CDCl_3$) δ 5.90 (d, *J* = 3.1 Hz, 1H), 5.65 (d, *J* = 3.1 Hz, 1H), 3.06 (s, 3H), 2.99 (s, 3H), 2.55–2.51 (m, 2H), 1.62-1.56 (m, 2H), 1.39-1.33 (m, 2H), 0.91 (t, *J* = 7.3 Hz, 3H).
^{13}C NMR (101 MHz, $CDCl_3$) δ 153.1, 149.9, 149.6, 105.8, 92.8, 37.0, 36.6, 30.0, 27.8, 22.3, 13.9.
HRMS (ESI): Calculated for $C_{11}H_{17}NO_3Na$ [M+Na$^+$]: 234.11006 found: 234.10947.
IR (neat): 2957, 2932, 1742, 1627, 1577, 1458, 1391, 1363, 1235, 1144, 1064, 1008, 971, 950, 840, 771, 747 cm^{-1}.

5-Phenylfuran-2-yl dimethylcarbamate (1c)

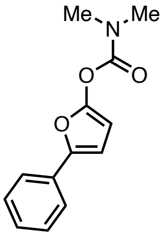

MW (g/mol): 231.25 **Molecular formula:** $C_{13}H_{13}NO_3$

Synthesized according to ***general procedure A***. The desired product was obtained as a brown oil (291 mg, 1.26 mmol, 42% yield) after purification by flash column chromatography over silica gel (*n*-Hexane/Ethyl acetate = 90:10).

^{1}H NMR (400 MHz, $CDCl_3$) δ 7.63-7.59 (m, 2H), 7.38-7.33 (m, 2H), 7.25-7.20 (m, 1H), 6.62 (d, *J* = 3.4 Hz, 1H), 5.93 (d, *J* = 3.4 Hz, 1H), 3.08 (s, 3H), 3.01 (s, 3H).

^{13}C NMR (101 MHz, CDCl$_3$) δ 152.5, 151.4, 146.8, 130.5, 128.6, 127.0, 123.2, 106.2, 94.6, 36.9, 36.5.

HRMS (ESI): Calculated for $C_{13}H_{13}NO_3Na$ [M+Na$^+$]: 254.07876 found: 254.07892.

IR (neat): 3059, 2932, 1740, 1623, 1600, 1555, 1487, 1449, 1388, 1357, 1284, 1237, 1142, 1056, 1011, 969, 828, 757, 692 cm^{-1}.

5-(*p*-Tolyl)furan-2-yl dimethylcarbamate (1d)

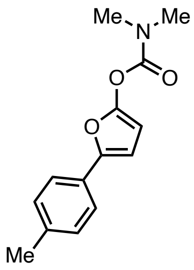

MW (g/mol): 245.28 **Molecular formula:** $C_{14}H_{15}NO_3$

Synthesized according to ***general procedure A***. The desired product was obtained as a brown oil (385 mg, 1.57 mmol, 87% yield) after purification by flash column chromatography over silica gel (*n*-Hexane/Ethyl acetate = 90:10).

^{1}H NMR (400 MHz, CDCl$_3$) δ 7.52-7.47 (m, 2H), 7.18-7.14 (m, 2H), 6.54 (d, *J* = 3.4 Hz, 1H), 5.89 (d, *J* = 3.4 Hz, 1H), 3.10 (s, 3H), 3.02 (s, 3H), 2.35 (s, 3H).

^{13}C NMR (101 MHz, CDCl$_3$) δ 152.6, 151.1, 147.1, 136.8, 129.3, 127.9, 123.3, 105.5, 94.6, 37.0, 36.7, 21.3.

HRMS (ESI): Calculated for $C_{14}H_{15}NO_3Na$ [M+Na$^+$]: 268.09441 found: 268.09415.

IR (neat): 3026, 2924, 1741, 1624, 1557, 1500, 1447, 1388, 1357, 1285, 1237, 1143, 1052, 1011, 969, 918, 815, 774, 734, 644, 603 cm^{-1}.

5-(4-(Trifluoromethyl)phenyl)furan-2-yl dimethylcarbamate (1e)

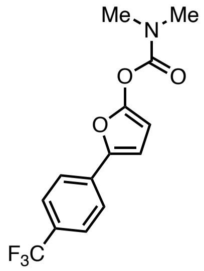

MW (g/mol): 299.25 **Molecular formula:** $C_{14}H_{12}F_3NO_3$

Synthesized according to ***general procedure A***. The desired product was obtained as a yellow oil (109 mg, 0.36 mmol, 56% yield) after purification by flash column chromatography over silica gel (*n*-Hexane/Ethyl acetate = 90:10).

^{1}H NMR (400 MHz, CDCl$_3$) δ 7.69-7.65 (m, 2H), 7.61-7.55 (m, 2H), 6.72 (d, *J* = 3.4 Hz, 1H), 5.95 (d, *J* = 3.4 Hz, 1H), 3.12 (s, 3H), 3.04 (s, 3H).

^{13}C NMR (101 MHz, CDCl$_3$) δ 152.4, 152.3, 145.4, 133.7, 128.6 (q, *J* = 32.4 Hz), 125.7 (q, *J* = 3.9 Hz), 124.3 (q, *J* = 271.7 Hz), 123.2, 108.5, 95.2, 37.1, 36.8.

HRMS (ESI): Calculated for $C_{14}H_{12}F_3NO_3Na$ [M+Na$^+$]: 322.06615 found: 322.06631.

IR (neat): 3142, 2931, 2361, 1742, 1609, 1555, 1497, 1390, 1361, 1322, 1241, 1145, 1110, 1070, 1018, 969, 833, 783, 739, 662 cm^{-1}.

3-Methylfuran-2-yl dimethylcarbamate (1f)

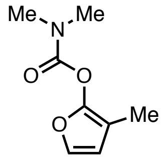

MW (g/mol): 169.18 **Molecular formula:** $C_8H_{11}NO_3$

Synthesized according to ***general procedure B***. The desired product was obtained as a colourless oil (124 mg, 0.54 mmol, 36% yield) after purification by flash column chromatography over silica gel (*n*-Hexane/Ethyl acetate = 90:10 to 80:20).

^{1}H NMR (400 MHz, CDCl$_3$) δ 7.00 (d, *J* = 2.1 Hz, 1H, H_1), 6.22 (d, *J* = 2.1 Hz, 1H, H_2), 3.09 (s, 3H, H_6), 3.01 (s, 3H, H_6), 1.89 (s, 3H, H_7).

^{13}C NMR (101 MHz, CDCl$_3$) δ 153.3, 147.8, 135.4, 113.7, 102.2, 37.1, 36.7, 8.6.
HRMS (ESI): Calculated for $C_8H_{10}NO_3Na$ [M+Na$^+$]: 192.0637, found: 192.0636.
IR (neat): 2927, 1740, 1655, 1372, 1223, 1152, 1106, 996, 886 cm^{-1}.

3-Methylfuran-2-yl dimethylcarbamate (1g)

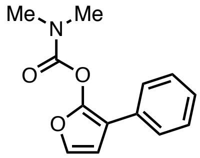

MW (g/mol): 231.25 **Molecular formula:** $C_{13}H_{13}NO_3$

Synthesized according to ***general procedure B***. The desired product was obtained as a yellow oil (74 mg, 0.32 mmol, 16% yield) after purification by flash column chromatography over silica gel (*n*-Hexane/Ethyl acetate = 75:25).

^{1}H NMR (400 MHz, CDCl$_3$) δ 7.49-7.45 (m, 2H, H$_8$), 7.40-7.34 (m, 2H, H$_9$), 7.26 (s, 1H, H$_{10}$), 7.15 (d, *J* = 2.4 Hz, 1H, H$_1$), 6.65 (d, *J* = 2.3 Hz, 1H, H$_2$), 3.15 (s, 3H, H$_6$), 3.03 (s, 3H, H$_6$).
^{13}C NMR (101 MHz, CDCl$_3$) δ 152.9, 147.3, 136.3, 131.6, 128.8, 126.8, 126.4, 111.0, 108.4, 37.2, 36.8.
HRMS (ESI): Calculated for $C_{13}H_{12}NO_3Na$ [M+Na$^+$]: 254.0793 found: 254.0785.
IR (neat): 2922, 1748, 1638, 1371, 1229, 1222, 1147, 1133, 770, 753 cm^{-1}.

3-(4-Ethylphenyl)furan-2-yl dimethylcarbamate (1h)

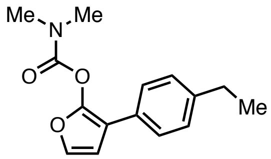

MW (g/mol): 259.30 **Molecular formula:** $C_{15}H_{17}NO_3$

Synthesized according to ***general procedure A***. The desired product was obtained as a yellow oil (31 mg, 0.12 mmol, 6% yield) after purification by flash column chromatography over silica gel (*n*-Hexane/Ethyl acetate = 75:25).

^{1}H NMR (400 MHz, CDCl$_3$) δ 7.39 (d, *J* = 8.2 Hz, 2H), 7.21 (d, *J* = 8.1 Hz, 2H), 7.14 (d, *J* = 2.2 Hz), 6.63 (d, *J* = 2.2 Hz), 3.15 (s, 3H), 3.03 (s, 3H), 2.68-2.63 (q, *J* = 7.6 Hz, 2H), 1.27-1.23 (t, *J* = 7.6 Hz, 3H).

^{13}C NMR (101 MHz, CDCl$_3$) δ 152.9, 146.9, 142.7, 136.1, 128.8, 128.2, 126.3, 111.0, 108.2, 37.1, 36.7, 28.6, 15.5.

HRMS (ESI): Calculated for $C_{15}H_{16}NO_3Na$ [M+Na$^+$]: 282.1106 found: 282.1105.

IR (neat): 2929, 1744, 1638, 1522, 1456, 1371, 1259, 1229, 1142, 1061, 1001, 971, 836, 789, 745 cm^{-1}.

3-(4-Fluorophenyl)furan-2-yl dimethylcarbamate (1i)

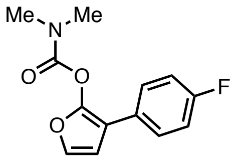

MW (g/mol): 249.24 **Molecular formula:** $C_{13}H_{12}NO_3F$

Synthesized according to ***general procedure A***. The desired product was obtained as a yellow oil (24.9 mg, 0.10 mmol, 5% yield) after purification by flash column chromatography over silica gel (*n*-Hexane/Ethyl acetate = 75:25).

^{1}H NMR (400 MHz, CDCl$_3$) δ 7.44-7.40 (dd, *J* = 8.9, 5.3 Hz, 2H), 7.15 (d, *J* = 2.3 Hz), 7.08-7.03 (t, *J* = 8.8 Hz, 2H), 6.60 (d, *J* = 2.3 Hz), 3.14 (s, 3H), 3.03 (s, 3H).

^{13}C NMR (101 MHz, CDCl$_3$) δ 162.9, 160.4, 152.7, 147.0, 136.3, 128.0, 115.7, 110.9, 107.6, 37.1, 36.7.

HRMS (ESI): Calculated for $C_{13}H_{11}NO_3FNa$ [M+Na$^+$]: 272.0699, found: 272.0688.

IR (neat): 2927, 1746, 1636, 1601, 1509, 1372, 1259, 1231, 1144, 1059, 1004, 839, 798, 744 cm^{-1}.

5-Cyclohexylfuran-2-yl dimethylcarbamate (1j)

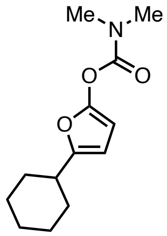

MW (g/mol): 237.30 **Molecular formula:** $C_{13}H_{19}NO_3$

Synthesized according to ***general procedure A***. The desired product was obtained as a yellow oil (183 mg, 0.77 mmol, 43% yield) after purification by flash column chromatography over silica gel (*n*-Hexane/Ethyl acetate = 95:5 to 90:10).

^{1}H NMR (400 MHz, CDCl$_3$) δ 5.87 (d, *J* = 3.2, 1H), 5.64 (d, *J* = 3.2 Hz, 1H), 3.06 (s, 3H), 2.99 (s, 3H), 2.02-1.96 (m, 1H), 1.91-1.54 (m, 4H), 1.37-1.17 (m, 6H).

^{13}C NMR (101 MHz, CDCl$_3$) δ 153.9, 153.1, 149.8, 103.9, 92.6, 37.1, 37.0, 36.6, 31.4, 26.2, 26.0.

HRMS (ESI): Calculated for $C_{13}H_{19}NO_3Na$ [M+Na$^+$]: 260.12571 found: 260.12554.

IR (neat): 2927, 2854, 1741, 1625, 1573, 1449, 1391, 1352, 1232, 1145, 1009, 970, 952, 891, 839, 772, 747 cm^{-1}.

5-Cyclopropylfuran-2-yl dimethylcarbamate (1k)

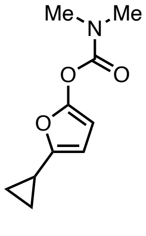

MW (g/mol): 195.22 **Molecular formula:** $C_{10}H_{13}NO_3$

Synthesized according to ***general procedure A***. The desired product was obtained as a yellow oil (84 mg, 0.43 mmol, 43% yield) after purification by flash column chromatography over silica gel (*n*-Hexane/Ethyl acetate = 95:5).

^{1}H NMR (400 MHz, CDCl$_3$) δ 5.87 (d, *J* = 3.2 Hz, 1H), 5.63 (d, *J* = 3.2 Hz, 1H), 3.05 (s, 3H), 2.98 (s, 3H), 1.79 (m 1H), 0.82-0.77 (m, 2H), 0.71–0.67 (m, 2H).

^{13}C NMR (101 MHz, $CDCl_3$) δ 153.0, 150.5, 149.5, 104.9, 93.0, 37.0, 36.6, 8.6, 6.2.

HRMS (ESI): Calculated for $C_{10}H_{14}NO_3$ [M+H$^+$]: 196.09682 found: 196.09711.

IR (neat): 3011, 2934, 1740, 1627, 1581, 1488, 1451, 1391, 1364, 1341, 1234, 1145, 1066, 1054, 1009, 970, 954, 872, 839, 772 cm^{-1}.

5-Phenethylfuran-2-yl dimethylcarbamate (1l)

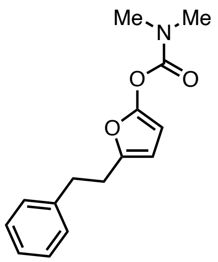

MW (g/mol): 259.31 **Molecular formula:** $C_{15}H_{17}NO_3$

Synthesized according to ***general procedure A***. The desired product was obtained as a brown oil (270 mg, 1.04 mmol, 45% yield) after purification by flash column chromatography over silica gel (*n*-Hexane/Ethyl acetate = 90:10 to 80:20).

^{1}H NMR (400 MHz, $CDCl_3$) δ 7.32-7.28 (m, 2H), 7.23-7.18 (m, 3H), 5.93 (dt, *J* = 3.2, 0.9 Hz, 1H), 5.69 (d, *J* = 3.2 Hz, 1H), 3.09 (s, 3H), 3.02 (s, 3H), 2.98-2.93 (m, 2H), 2.89-2.84 (m, 2H).

^{13}C NMR (101 MHz, $CDCl_3$) δ 153.0, 150.1, 148.3, 141.2, 128.44, 128.43, 126.1, 106.5, 92.9, 37.0, 36.6, 34.3, 30.1.

HRMS (ESI): Calculated for $C_{15}H_{17}NO_3Na$ [M+Na$^+$]: 282.1101 found: 282.1097.

IR (neat): 2930, 1740, 1626, 1577, 1495, 1454, 1363, 1234, 1143, 1015, 957, 840, 775, 746, 698 cm^{-1}.

4-Methylfuran-2-yl dimethylcarbamate (1m)

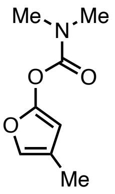

MW (g/mol): 169.18 **Molecular formula:** $C_8H_{11}NO_3$

Synthesized according to ***general procedure A***. The desired product was obtained as a yellow oil (178 mg, 1.05 mmol, 35% yield) after purification by flash column chromatography over silica gel (*n*-Hexane/Ethyl acetate = 90:10).

^{1}H NMR (400 MHz, $CDCl_3$) δ 6.82 (Hept, *J* = 1.3 Hz, 1H), 5.67 (t, *J* = 1.3 Hz, 1H), 3.06 (s, 3H), 2.99 (s, 3H), 1.92 (d, *J* = 1.3 Hz, 3H).

^{13}C NMR (101 MHz, $CDCl_3$) δ 152.8, 151.5, 132.1, 121.6, 95.1, 37.0, 36.6, 10.6.

HRMS (ESI): Calculated for $C_8H_{11}NO_3Na$ [M+Na$^+$]: 192.0631 found: 192.0629.

IR (neat): 3735, 2927, 2360, 2342, 1742, 1630, 1387, 1365, 1290, 1269, 1211, 1159, 1002, 950, 913, 841, 788, 733 cm^{-1}.

5-(*o*-Tolyl)furan-2-yl dimethylcarbamate (1n)

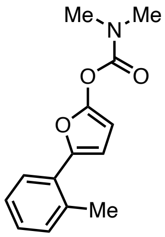

MW (g/mol): 245.28 **Molecular formula:** $C_{14}H_{15}NO_3$

Synthesized according to ***general procedure A***. The desired product was obtained as a brown oil (314 mg, 1.28 mmol, 64% yield) after purification by flash column chromatography over silica gel (*n*-Hexane/Ethyl acetate = 90:10).

^{1}H NMR (400 MHz, $CDCl_3$) δ 7.64 (m, 1H), 7.25-7.16 (m, 3H), 6.49 (d, *J* = 3.4 Hz, 1H), 5.94 (d, *J* = 3.4 Hz, 1H, H_2), 3.11 (s, 3H), 3.03 (s, 3H), 2.49 (s, 3H, H_{11}).

^{13}C NMR (101 MHz, $CDCl_3$) δ 152.6, 151.2, 146.4, 134.4, 131.1, 129.9, 127.2, 126.7, 126.0, 110.1, 94.3, 37.1, 36.7, 21.9.

HRMS (ESI): Calculated for $C_{14}H_{15}NO_3Na$ [M+Na$^+$]: 268.09441 found: 268.09428.

IR (neat): 2932, 1742, 1621, 1546, 1487, 1460, 1389, 1354, 1240, 1144, 1013, 972, 919, 829, 759, 720 cm^{-1}.

5-(4-Bromophenyl)furan-2-yl dimethylcarbamate (1o)

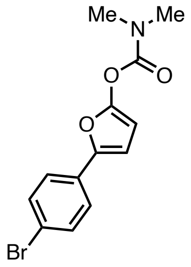

MW (g/mol): 310.15 **Molecular formula:** $C_{13}H_{12}BrNO_3$

Synthesized according to ***general procedure A***. The desired product was obtained as a brown oil (298 mg, 0.96 mmol, 80% yield) after purification by flash column chromatography over silica gel (*n*-Hexane/Ethyl acetate = 95:5 to 90:10).

^{1}H NMR (400 MHz, CDCl$_3$) δ 7.48-7.42 (m, 4H), 6.60 (d, *J* = 3.4 Hz, 1H), 5.91 (d, *J* = 3.4 Hz, 1H), 3.11 (s, 3H), 3.03 (s, 3H).

^{13}C NMR (101 MHz, CDCl$_3$) δ 152.5, 151.7, 145.9, 131.8, 129.5, 124.8, 120.8, 107.0, 95.0, 37.1, 36.7.

HRMS (ESI): Calculated for $C_{13}H_{12}BrNO_3Na$ [M+Na$^+$]: 333.98723 found: 333.98716.

IR (neat): 2933, 2361, 1743, 1622, 1572, 1548, 1480, 1388, 1356, 1238, 1146, 1073, 1008, 970, 820, 777 cm^{-1}.

5-(4-Nitrophenyl)furan-2-yl dimethylcarbamate (1p)

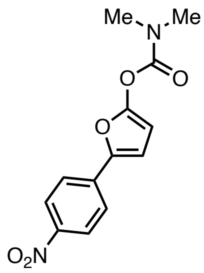

MW (g/mol): 276.25 **Molecular formula:** $C_{13}H_{12}N_2O_5$

Synthesized according to ***general procedure A***. The desired product was obtained as a brown oil (113 mg, 0.41 mmol, 34% yield) after purification by flash column chromatography over silica gel (*n*-Hexane/Ethyl acetate = 90:10).

^{1}H NMR (400 MHz, CDCl$_3$) δ 8.19 (d, *J* = 8.8 Hz, 2H), 7.67 (d, *J* = 8.8 Hz, 2H), 6.83 (d, *J* = 3.7 Hz, 1H), 6.00 (d, *J* = 3.7 Hz, 1H), 3.12 (s, 3H), 3.03 (s, 3H).

^{13}C NMR (101 MHz, CDCl$_3$) δ 153.2, 152.1, 146.1, 144.5, 136.2, 124.4, 123.3, 110.8, 95.8, 37.2, 36.8.

HRMS (ESI): Calculated for $C_{13}H_{12}N_2O_5Na$ [M+Na$^+$]: 299.06384 found: 299.06400.

IR (neat): 3110, 2928, 1750, 1640, 1592, 1550, 1513, 1389, 1335, 1242, 1147, 1109, 1021, 970, 853, 752 cm^{-1}.

Methyl 4-(5-((dimethylcarbamoyl)oxy)furan-2-yl)benzoate (1q)

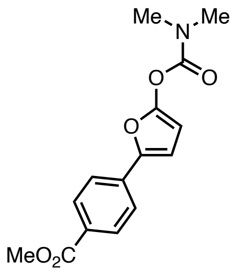

MW (g/mol): 289.29 **Molecular formula:** $C_{15}H_{15}NO_5$

Synthesized according to ***general procedure A***. The desired product was obtained as a yellow oil (190 mg, 0.66 mmol, 47% yield) after purification by flash column chromatography over silica gel (*n*-Hexane/Ethyl acetate = 90:10 to 70:30).

^{1}H NMR (400 MHz, CDCl$_3$) δ 8.01 (d, *J* = 8.5 Hz, 2H), 7.63 (d, *J* = 8.5 Hz, 2H), 6.74 (d, *J* = 3.3 Hz, 1H), 5.95 (d, *J* = 3.3 Hz, 1H), 3.91 (s, 3H), 3.11 (s, 3H), 3.03 (s, 3H).

^{13}C NMR (101 MHz, CDCl$_3$) δ 166.9, 152.3, 145.8, 134.5, 130.2, 128.3, 122.8, 108.8, 95.3, 52.2, 37.1, 36.8 *(C5 carbon was not detected).*

HRMS (ESI): Calculated for $C_{15}H_{15}NO_5Na$ [M+Na$^+$]: 312.08424 found: 312.08399.

IR (neat): 2949, 1747, 1715, 1603, 1573, 1551, 1436, 1389, 1356, 1275, 1242, 1145, 1109, 1014, 969, 919, 857, 827, 769, 699 cm^{-1}.

5-5-(Thiophen-2-yl)furan-2-yl dimethylcarbamate (1r)

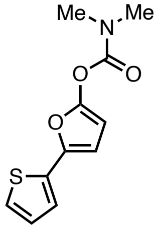

MW (g/mol): 237.27 **Molecular formula:** $C_{11}H_{11}NO_3S$

Synthesized according to ***general procedure A***. The desired product was obtained as a brown oil (256.3 mg, 1.08 mmol, 36% yield) after purification by flash column chromatography over silica gel (*n*-Hexane/Ethyl acetate = 90:10).

^{1}H NMR (400 MHz, CDCl$_3$) δ 7.19-7.16 (m, 2H), 6.99 (dd, *J* = 4.8, 3.8 Hz, 1H), 6.44 (d, *J* = 3.4 Hz, 1H), 5.87 (d, *J* = 3.4 Hz, 1H), 3.10 (s, 3H), 3.02 (s, 3H).

^{13}C NMR (101 MHz, CDCl$_3$) δ 152.5, 150.9, 142.6, 133.5, 127.6, 123.7, 122.3, 106.4, 94.7, 37.1, 36.7.

HRMS (ESI): Calculated for $C_{11}H_{11}NO_3SNa$ [M+Na$^+$]: 260.0352 found: 260.0347.

IR (neat): 2925, 1742, 1621, 1569, 1493, 1390, 1235, 1144, 1048, 1000, 967, 845, 775, 743, 697 cm^{-1}.

5-(Oct-1-yn-1-yl)furan-2-yl dimethylcarbamate (1s)

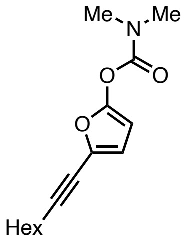

MW (g/mol): 277.32 **Molecular formula:** $C_{15}H_{19}NO_4$

Synthesized according to ***general procedure A***. The desired product was obtained as a yellow oil (255 mg, 0.92 mmol, 46% yield) after purification by flash column chromatography over silica gel (*n*-Hexane/Ethyl acetate = 95:5).

^{1}H NMR (400 MHz, CDCl$_3$) δ 6.39 (d, *J* = 3.4 Hz, 1H), 5.77 (d, *J* = 3.4 Hz, 1H), 3.04 (s, 3H), 2.98 (s, 3H), 2.38 (t, *J* = 7.1 Hz, 2H), 1.59-1.52 (m, 2H), 1.44-1.38 (m, 2H), 1.33-1.27 (m, 4H), 0.88 (t, *J* = 6.9 Hz, 3H).

^{13}C NMR (101 MHz, CDCl$_3$) δ 152.2, 150.7, 130.8, 115.4, 94.3, 93.6, 70.7, 37.0, 36.6, 31.4, 28.7, 28.5, 22.6, 19.5, 14.2.

HRMS (ESI): Calculated for $C_{15}H_{21}NO_3Na$ [M+Na$^+$]: 286.1414 found: 286.1407.

IR (neat): 2929, 2858, 1747, 1614, 1548, 1389, 1352, 1236, 1194, 1065, 1007, 969, 829, 776, 743 cm^{-1}.

Thiophen-2-yl dimethylcarbamate (1t)

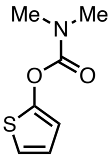

MW (g/mol): 171.21 **Molecular formula:** $C_7H_9NO_2S$

Synthesized according to ***general procedure A***. The desired product was obtained as a yellow oil (231 mg, 1.35 mmol, 45% yield) after purification by flash column chromatography over silica gel (*n*-Hexane/Ethyl acetate = 90:10).

^{1}H NMR (400 MHz, CDCl$_3$) δ 6.83 (dd, *J* = 5.8, 1.6 Hz, 1H), 6.79 (dd, *J* = 5.8, 3.8 Hz, 1H), 6.62 (dd, *J* = 3.8, 1.6 Hz, 1H), 3.08 (s, 3H), 3.02 (s, 3H).

^{13}C NMR (101 MHz, CDCl$_3$) δ 153.3, 153.2, 123.1, 117.5, 112.6, 36.8, 36.3.

HRMS (ESI): Calculated for $C_7H_{10}NO_2S$ [M+H$^+$]: 172.04268 found: 172.04280.

IR (neat): 2935, 2362, 1723, 1542, 1499, 1437, 1382, 1271, 1234, 1198, 1149, 1064, 1034, 995, 913, 847, 806, 749, 681, 646, 603 cm^{-1}.

1-Benzyl-1*H*-pyrrol-2-yl dimethylcarbamate (1u)

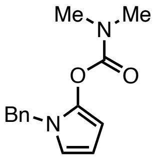

MW (g/mol): 244.29 **Molecular formula:** $C_{14}H_{16}N_2O_2$

Synthesized according to ***general procedure A***. The desired product was obtained as a brown oil (388 mg, 1.59 mmol, 53% yield) after purification by flash column chromatography over silica gel (*n*-Hexane/Ethyl acetate = 90:10 to 70:30).

^{1}H NMR (400 MHz, CDCl$_3$) δ 7.34-7.27, m, 3H), 7.13 (d, *J* = 6.9 Hz, 2H), 6.36 (dd, *J* = 3.2, 2.0 Hz, 1H), 6.08 (t, *J* = 3.5 Hz, 1H), 5.82 (dd, *J* = 3.7, 2.0 Hz, 1H), 4.93 (s, 2H), 2.95 (s, 3H), 2.90 (s, 3H).

^{13}C NMR (101 MHz, CDCl$_3$) δ 153.8, 137.7, 137.0, 128.7, 127.6, 127.1, 115.4, 106.2, 95.3, 49.3, 37.0, 36.5.

HRMS (ESI): Calculated for $C_{14}H_{16}N_2O_2Na$ [M+Na$^+$]: 267.11040 found: 267.11024.

IR (neat): 2979, 2362, 1727, 1698, 1561, 1496, 1453, 1380, 1302, 1269, 1211, 1151, 1065, 1028, 995, 892, 843, 796, 751, 694, 641 cm^{-1}.

1-Tosyl-1*H*-pyrrol-2-yl dimethylcarbamate (1v)

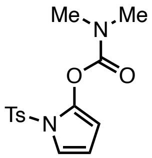

MW (g/mol): 308.35 **Molecular formula:** $C_{14}H_{16}N_2O_4S$

Synthesized according to ***general procedure A***. The desired product was obtained as a brown oil (222 mg, 0.72 mmol, 48% yield) after purification by flash column chromatography over silica gel (*n*-Hexane/Ethyl acetate = 90:10 to 80:20).

^{1}H NMR (400 MHz, CDCl$_3$) δ 7.71 (d, *J* = 8.4 Hz, 2H), 7.29 (d, *J* = 8.2 Hz, 2H), 6.95 (dd, *J* = 3.8, 1.9 Hz, 1H), 6.15 (t, *J* = 3.7 Hz, 1H), 5.87 (dd, *J* = 3.6, 1.9 Hz, 1H), 3.09 (s, 3H), 2.96 (s, 3H), 2.40 (s, 3H).

^{13}C NMR (101 MHz, CDCl$_3$) δ 152.8, 145.2, 137.3, 136.1, 130.0, 127.1, 116.7, 110.3, 101.2, 37.1, 36.6, 21.8.

HRMS (ESI): Calculated for $C_{14}H_{16}N_2O_4SNa$ [M+Na$^+$]: 331.07230 found: 331.07231.

IR (neat): 2930, 1744, 1596, 1482, 1399, 1372, 1290, 1239, 1191, 1177, 1150, 1089, 1048, 1019, 985, 841, 814, 745, 704, 672 cm^{-1}.

b. Synthesis of starting furanones and lactams

i. Synthesis of 5-substituted furanones

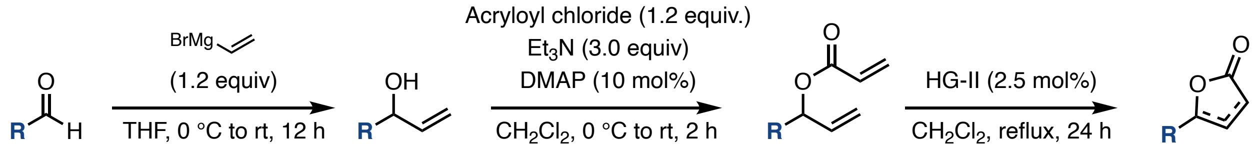

General procedure C:

To a solution of aldehyde (1.0 equiv.) in THF (0.1 M) at 0 °C, was added vinyl magnesium bromide (1.5 equiv., 1.0 M in THF) dropwise under argon. After stirring for 15 min, the reaction mixture was stirred for 2 h at rt. A saturated aqueous solution of NH_4Cl was added, and the mixture was extracted three times with EtOAc. The combined organic layers were washed with a saturated aqueous solution of brine, dried over anhydrous $MgSO_4$, filtered and concentrated under reduced pressure to give the crude allylic alcohol. *The allylic alcohol was directly engaged in the next step without further purification.*

To a solution of the crude allylic alcohol (1.0 equiv.), Et_3N (3.0 equiv.) and 4-DMAP (10 mol%) in CH_2Cl_2 (0.2 M) at 0 °C, acryloyl chloride (1.5 equiv.) was added dropwise under argon. After stirring for 30 min at 0 °C, the reaction mixture was stirred 2 h at rt. Water was added and the mixture was extracted three times with CH_2Cl_2. The combined organic layers were washed with an aqueous solution of HCl (1.0 M) then water and a saturated aqueous solution of brine, dried over anhydrous $MgSO_4$, filtered and concentrated under reduced pressure. The resulting crude residue was diluted into a pentane/Et_2O (4:1) mixture and filtered through a short pad of silica gel and the filtrate was concentrated under reduced pressure to afford the crude allylic ester. *The allylic ester was directly engaged in the next step without further purification.*

To a solution of the crude allylic ester (1.0 equiv.) in CH_2Cl_2 (0.2 M) was added the Hoveyda-Grubbs II catalyst (**H-G II**) (2.5 mol%). The reaction mixture was stirred 24 h at 50 °C and then cooled to rt, diluted with CH_2Cl_2 and filtered through a short pad of Celite®. The filtrate was concentrated under reduced pressure, and the crude residue was finally purified by flash column chromatography over silica gel (pentane/Et_2O = 19:1 to 1:1) to afford the corresponding substituted furan-2(5*H*)-one.

5-Cyclohexylfuran-2(5*H*)-one

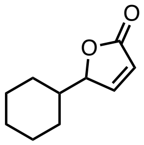

MW (g/mol): 166.22 **Molecular formula:** $C_{10}H_{14}O_2$

Prepared from cyclohexylcarboxaldehyde (1.1 g, 10.0 mmol, 1.0 equiv.) and vinylmagnesium bromide (15 mL, 1.0 M in THF, 1.5 equiv.) following ***general procedure C***. The product was purified by flash column chromatography over silica gel (Pentane/Et_2O) to give the titled compound (303 mg, 1.82 mmol, 18%) as a brown oil. *The spectral data were in agreement with the literature.*[2]

^{1}H NMR (400 MHz, $CDCl_3$) δ 7.46 (dd, *J* = 5.8, 1.5 Hz, 1H), 6.11 (dd, *J* = 5.8, 2.0 Hz, 1H), 4.84 (dt, *J* = 5.6, 1.8 Hz, 1H), 1.80-1.65 (m, 6H, H_7), 1.26-1.06 (m, 5H, H_5).
^{13}C NMR (101 MHz, $CDCl_3$) δ 173.3, 155.2, 122.0, 87.7, 41.4, 28.7, 28.3, 26.2, 25.9, 25.8.

5-Cyclopropylfuran-2(5*H*)-one

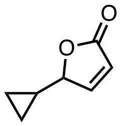

MW (g/mol): 124.14 **Molecular formula:** $C_7H_8O_2$

Prepared from cyclopropanecarboxaldehyde (1.0 g, 14.27 mmol, 1.0 equiv.) and vinylmagnesium bromide (21.4 mL, 1.0 M in THF, 1.5 equiv.) following ***general procedure C***. The product was purified by flash column chromatography over silica gel (Pentane/Et_2O) to give the titled compound (125 mg, 1.01 mmol, 7%) as a brown oil.

^{1}H NMR (400 MHz, $CDCl_3$) δ 7.44-7.40 (m, 1H), 6.04 (dd, *J* = 5.8, 1.9 Hz, 1H), 4.48 (dt, *J* = 7.9, 1.7 Hz, 1H), 0.91 (m, 1H), 0.58 (m, 2H), 0.46-0.35 (m, 2H).
^{13}C NMR (101 MHz, $CDCl_3$) δ 173.0, 155.6, 121.6, 86.6, 12.8, 3.0, 1.4.
HRMS (ESI): Calculated for $C_7H_8O_2Na$ [M+Na$^+$]: 147.04165 found: 147.04154.
IR (neat): 3088, 3011, 2361, 2341, 1775, 1744, 1602, 1311, 1161, 1087, 1038, 988, 886, 870, 814, 699 cm^{-1}.

5-Phenethylfuran-2(5*H*)-one

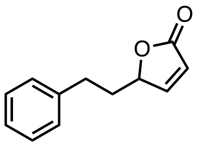

MW (g/mol): 188.23 **Molecular formula:** $C_{12}H_{12}O_2$

Prepared from hydrocinnamaldehyde (1.0 g, 7.46 mmol, 1.0 equiv.) and vinylmagnesium bromide (11.2 mL, 1.0 M in THF, 1.5 equiv.) following ***general procedure C***. The product was purified by flash column chromatography over silica gel (Pentane/Et_2O) to give the titled compound (450 mg, 2.39 mmol, 32%) as a brown oil. *The spectral data were in agreement with the literature.*[3]

^{1}H NMR (400 MHz, $CDCl_3$) δ 7.40 (dd, *J* = 5.8, 1.5 Hz, 1H), 7.34-7.15 (m, 5H), 6.10 (dd, *J* = 5.7, 2.0 Hz, 1H), 5.01 (m, 1H), 2.81 (m, 2H), 2.12-2.03 (m, 1H), 1.93 (m, 1H).
^{13}C NMR (101 MHz, $CDCl_3$) δ 173.1, 156.3, 140.3, 128.7, 128.6, 126.4, 121.6, 82.5, 35.0, 31.4.

5-Phenylfuran-2(5*H*)-one

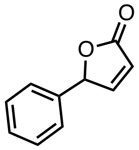

MW (g/mol): 160.17 **Molecular formula:** $C_{10}H_8O_2$

Prepared from benzaldehyde (2.0 g, 18.9 mmol, 1.0 equiv.) and vinylmagnesium bromide (28.3 mL, 1.0 M in THF, 1.5 equiv.) following the ***general procedure C***. The product was purified by flash column chromatography over silica gel (Pentane/Et_2O) to give the titled compound (484 mg, 3.02 mmol, 16%) as a red oil. *The spectral data were in agreement with the literature.*[4]

^{1}H NMR (400 MHz, $CDCl_3$) δ 7.46 (dd, *J* = 5.6, 1.7 Hz, 1H), 7.34-7.29 (m, 3H), 7.21-7.18 (m, 2H), 6.15 (dd, *J* = 5.6, 2.1 Hz, 1H), 5.94 (t, *J* = 2.1 Hz, 1H).
^{13}C NMR (101 MHz, $CDCl_3$) δ 173.2, 156.0, 134.3, 129.5, 129.2, 126.6, 121.1, 84.5.

5-(*p*-Tolyl)furan-2(5*H*)-one

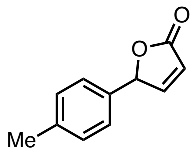

MW (g/mol): 174.20 **Molecular formula**: $C_{11}H_{10}O_2$

Prepared from 4-methylbenzaldehyde (1.0 g, 8.33 mmol, 1.0 equiv.) and vinylmagnesium bromide (12.5 mL, 1.0 M in THF, 1.5 equiv.) following the ***general procedure C***. The product was purified by flash column chromatography over silica gel (Pentane/Et_2O) to give the title compound (315 mg, 1.81 mmol, 22%) as a red oil. *The spectral data were in agreement with the literature.*[5]

^{1}H NMR (400 MHz, $CDCl_3$) δ 7.51 (dd, *J* = 5.6, 1.6 Hz, 1H), 7.21–7.12 (m, 4H), 6.21 (dd, *J* = 5.6, 2.1 Hz, 1H), 5.98 (t, *J* = 1.9 Hz, 1H), 2.35 (s, 3H).

^{13}C NMR (101 MHz, $CDCl_3$) δ 173.3, 156.0, 139.5, 131.2, 129.8, 126.6, 121.0, 84.5, 21.3.

5-(*o*-Tolyl)furan-2(5*H*)-one

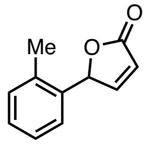

MW (g/mol): 174.20 **Molecular formula:** $C_{11}H_{10}O_2$

Prepared from 2-methylbenzaldehyde (1.0 g, 8.33 mmol, 1.0 equiv.) and vinylmagnesium bromide (12.5 mL, 1.0 M in THF, 1.5 equiv.) following the ***general procedure C***. The product was purified by flash column chromatography over silica gel (Pentane/Et_2O) to give the titled compound (380 mg, 2.18 mmol, 26%) as a brown oil.

^{1}H NMR (400 MHz, $CDCl_3$) δ 7.63–7.56 (m, 1H), 7.30-7.18 (m, 3H), 7.09 (dd, *J* = 7.7, 1.4 Hz, 1H), 6.27-6.22 (m, 2H), 2.46 (s, 3H).

^{13}C NMR (101 MHz, $CDCl_3$) δ 173.3, 155.4, 136.2, 132.6, 131.1, 129.3, 126.7, 126.1, 121.5, 82.0, 19.2.

HRMS (ESI): Calculated for $C_{11}H_{11}O_2$ [$M+H^+$]: 175.07536 found: 175.07546.

IR (neat): 2919, 2361, 1792, 1753, 1601, 1462, 1291, 1268, 1160, 1085, 1032, 897, 851, 818, 756 cm^{-1}.

5-(4-Bromophenyl)furan-2(3*H*)-one

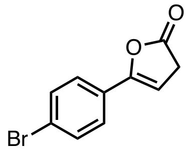

MW (g/mol): 239.07 **Molecular formula:** $C_{10}H_7BrO_2$

Prepared from 4-bromobenzaldehyde (1.0 g, 5.41 mmol, 1.0 equiv.) and vinylmagnesium bromide (8.0 mL, 1.0 M in THF, 1.5 equiv.) following ***general procedure C***. The product was purified by flash column chromatography over silica gel (Pentane/Et_2O) to give the titled compound (300 mg, 1.26 mmol, 23%) as a colorless oil. *The spectral data were in agreement with the literature.*[6]

^{1}H NMR (400 MHz, $CDCl_3$) δ 7.57-7.52 (m, 2H), 7.50–7.42 (m, 2H), 5.81 (t, *J* = 2.8 Hz, 1H), 3.41 (d, *J* = 2.8 Hz, 2H).
^{13}C NMR (101 MHz, $CDCl_3$) δ 175.6, 153.2, 132.1, 127.4, 126.4, 123.9, 98.5, 34.8.

5-(4-(Trifluoromethyl)phenyl)furan-2(3*H*)-one

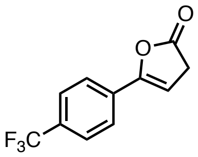

MW (g/mol): 228.17 **Molecular formula:** $C_{11}H_7F_3O_2$

Prepared from 4-(trifluoromethyl)benzaldehyde (1.0 g, 5.75 mmol, 1.0 equiv.) and vinylmagnesium bromide (8.6 mL, 1.0 M in THF, 1.5 equiv.) following the ***general procedure C***. The product was purified by flash column chromatography over silica gel (Pentane/Et_2O) to give the title compound (150 mg, 0.658 mmol, 11%) as a yellow oil.

^{1}H NMR (400 MHz, $CDCl_3$) δ 7.75-7.64 (m, 4H), 5.94 (t, *J* = 2.8 Hz, 1H), 3.47 (d, *J* = 2.7 Hz, 2H).

^{13}C NMR (101 MHz, $CDCl_3$) δ 175.2, 152.9, 131.7, 131.5 (q, *J* = 32.6 Hz), 125.9 (q, *J* = 3.9 Hz), 125.3 (q, *J* = 253.4 Hz), 125.2, 100.3, 34.8.

HRMS (ESI): Calculated for $C_{11}H_7F_3O_2Na$ [M+Na$^+$]: 251.02904 found: 251.02939.

IR (neat): 2940, 2361, 1808, 1735, 1702, 1414, 1325, 1298, 1249, 1168, 1113, 1068, 1012, 847 cm^{-1}.

5-(4-Nitrophenyl)furan-2(3*H*)-one

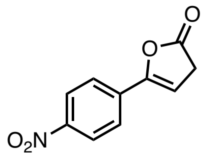

MW (g/mol): 205.17 **Molecular formula:** $C_{10}H_7NO_4$

Prepared from 4-nitrobenzaldehyde (1.0 g, 6.62 mmol, 1.0 equiv.) and vinylmagnesium bromide (9.9 mL, 1.0 M in THF, 1.5 equiv.) following ***general procedure C***. The product was purified by flash column chromatography over silica gel (Pentane/Et_2O) to give the titled compound (258 mg, 1.26 mmol, 19%) as a brown solid.

^1H NMR (400 MHz, $CDCl_3$) δ 8.33–8.22 (m, 2H), 7.81–7.73 (m, 2H), 6.05 (t, *J* = 2.8 Hz, 1H), 3.50 (d, *J* = 2.8 Hz, 2H).

^{13}C NMR (101 MHz, $CDCl_3$) δ 174.7, 152.2, 148.3, 134.1, 125.7, 124.3, 102.3, 34.9.

HRMS (ESI): Calculated for $C_{10}H_6NO_4$ [M-H$^+$]: 204.0291 found: 204.0283.

IR (neat): 3734, 2928, 2361, 2341, 1808, 1604, 1593, 1514, 1412, 1388, 1350, 1338, 1310, 1108 cm^{-1}.

Melting point: 145−147 °C

Methyl 4-(5-oxo-4,5-dihydrofuran-2-yl)benzoate

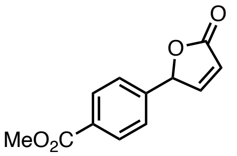

MW (g/mol): 218.21 **Molecular formula:** $C_{12}H_{10}O_4$

Prepared from methyl 4-formylbenzoate (1.0 g, 6.10 mmol, 1.0 equiv.) and vinylmagnesium bromide (9.1 mL, 1.0 M in THF, 1.5 equiv.) following ***general procedure C***. The product was purified by flash column chromatography over silica gel (Pentane/Et_2O) to give the titled compound (346 mg, 1.59 mmol, 26%) as a brown solid. *The spectral data were in agreement with the literature.*[7]

^{1}H NMR (400 MHz, $CDCl_3$) δ 8.00 (d, *J* = 8.4 Hz, 2H), 7.48 (dd, *J* = 5.7, 1.7 Hz, 1H), 7.29 (d, *J* = 8.0 Hz, 2H), 6.18 (dd, *J* = 5.6, 2.1 Hz, 1H), 6.00 (t, *J* = 1.9 Hz, 1H), 3.86 (s, 3H).
^{13}C NMR (101 MHz, $CDCl_3$) δ 172.8, 166.5, 155.5, 139.3, 131.1, 130.4, 126.4, 121.4, 83.7, 52.4.

5-Hydroxyfuran-2(5*H*)-one

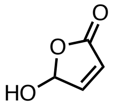

MW (g/mol): 100.07 **Molecular formula:** $C_4H_4O_3$

According to the literature procedure.[8] To a three-neck rounded bottom flask equipped with a condenser was introduced furfural (50 g, 0.52 mol, 1 equiv.), dry MeOH (260 mL) and rose bengal (1.09 g, 1.04 mmol, 0.2 mol%). Oxygen was bubbled (gentle flow) from the bottom of the reactor, and the solution was irradiated with a lamp during 48 h. The reaction temperature was maintained below 30 °C (air stream can be used for cooling). The solution was carefully concentrated under reduced pressure. The resulting solid was taken up in cold $CHCl_3$ (–78 °C) and the resulting solid was collected by filtration under *vacuum*, washed with $CHCl_3$ then 3 times with petroleum ether and dried under high *vacuum* to afford 5-hydroxyfuran-2(5*H*)-one (230 mmol, 23 g, 44%) as a pink solid. *The spectral data were in agreement with the literature.*[8]

^{1}H NMR (400 MHz, CDCl$_3$) δ 7.31 (dd, *J* = 5.7, 1.2 Hz, 1H), 6.25 (m, 1H), 6.21 (dd, *J* = 5.7, 1.2 Hz, 1H), 5.43 (br s, 1H).
^{13}C NMR (101 MHz, CDCl$_3$) δ 172.0, 152.5, 124.6, 99.1.

5-Butylfuran-2(5*H*)-one

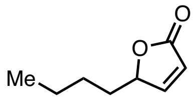

MW (g/mol): 140.18 **Molecular formula:** $C_8H_{12}O_2$

According to the literature procedure.[9] To a solution 5-hydroxy-2(5*H*)-furanone (1.0 g, 10.0 mmol, 1.0 equiv.) in THF (100 mL, 0.1 M) at –78 °C was slowly added *n*-BuLi (8.0 mL, 2.5 M in hexane, 2.0 equiv.). After 1H at –78 °C the reaction mixture was allowed to warm to 0 °C. An aqueous solution of HCl (3 M) was slowly added until pH 1 and the reaction mixture was extracted three times with Et_2O. The combined organic layers were washed with a saturated aqueous solution of brine, dried over anhydrous $MgSO_4$, filtered and concentrated under reduced pressure. The resulting oil was kept at 0 °C during 24 h (without stirring). Purification by flash column chromatography over silica gel (pentane/Et_2O) afforded the desired compound (955 mg, 6.81 mmol, 68%) as a yellow oil. *The spectral data were in agreement with the literature.*[4]

^{1}H NMR (400 MHz, CDCl$_3$) δ 7.45 (dd, *J* = 5.7, 1.5 Hz, 1H), 6.09 (dt, *J* = 5.7, 1.5 Hz, 1H), 5.02 (ddt, *J* = 7.2, 5.3, 1.7 Hz, 1H), 1.80-1.62 (m, 2H), 1.46-1.32 (m, 4H), 0.90 (t, *J* = 7.2 Hz, 3H).
^{13}C NMR (101 MHz, CDCl$_3$) δ 173.3, 156.5, 121.6, 83.5, 33.0, 27.1, 22.5, 13.9.

5-(Thiophen-2-yl)furan-2(5*H*)-one

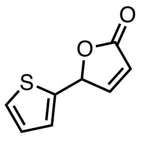

MW (g/mol): 166.19 **Molecular formula:** $C_8H_6O_2S$

Prepared according to a reported procedure.[11] To a solution of thiophene (0.8 mL, 10.0 mmol, 2.0 equiv.) in 10 mL of THF at 0 °C, *n*-BuLi (4.0 mL, 2.5 M in hexane, 2.0 equiv.) was slowly added and the reaction mixture was maintained at this temperature during 30 min. After

30 min, this solution was transferred through a cannula to a solution of 5-hydroxy-2(5*H*)-furanone (500 mg, 5.0 mmol, 1.0 equiv.) in THF (50 mL, 0.1 M) at –78 °C. After 1 h at –78 °C, the reaction mixture was allowed to warm to 0 °C. An aqueous solution of HCl (3 M) was slowly added until pH 1 and the reaction mixture was extracted three times with Et_2O. The combined organic layers were washed with a saturated aqueous solution of brine, dried over anhydrous $MgSO_4$, filtered and concentrated under reduced pressure. The resulting oil was kept at 0 °C during 24 h (without stirring). Purification by flash column chromatography over silica gel (pentane/Et_2O) afforded the desired compound (640 mg, 3.85 mmol, 77%) as a yellow oil. *The spectral data were in agreement with the literature.*[10]

^{1}H NMR (400 MHz, $CDCl_3$) δ 7.56 (dd, *J* = 5.3, 2.0 Hz, 1H), 7.36 (dd, *J* = 5.1, 1.2 Hz, 1H), 7.09 (dd, *J* = 3.6, 1.2 Hz, 1H), 7.01 (dd, *J* = 5.1, 3.5 Hz, 1H), 6.26-6.22 (m, 2H).
^{13}C NMR (101 MHz, $CDCl_3$) δ 172.5, 154.7, 136.5, 127.8, 127.6, 127.4, 122.0, 79.7.

5-(Oct-1-yn-1-yl)furan-2(5*H*)-one

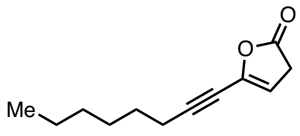

MW (g/mol): 192.26 **Molecular formula:** $C_{12}H_{16}O_2$

According to the literature procedure.[11] To a solution of 1-octyne (1.5 mL, 10.0 mmol, 2.0 equiv.) in 10 mL of THF at 0 °C, *n*-BuLi (4.0 mL, 2.5 M in hexane, 2.0 equiv.) was slowly added and the reaction mixture was maintained at this temperature during 30 min. After 30 min, this solution was transferred through a cannula to a solution of 5-hydroxy-2(5*H*)-furanone (500 mg, 5.0 mmol, 1.0 equiv.) in THF (50 mL, 0.1 M) at –78 °C. After 1H at –78 °C, the reaction mixture was allowed to warm to 0 °C. An aqueous solution of HCl (3 M) was slowly added until pH 1, and the reaction mixture was extracted three times with Et_2O. The combined organic layers were washed with a saturated aqueous solution of brine, dried over anhydrous $MgSO_4$, filtered and concentrated under reduced pressure. The resulting oil was kept at 0 °C during 24 h (without stirring). Purification by flash column chromatography over silica gel

(pentane/Et_2O) afforded the desired 5-(oct-1-yn-1-yl)furan-2(5*H*)-one (44 mg, 2.30 mmol, 46%) as a yellow oil.

^{1}H NMR (400 MHz, $CDCl_3$) δ 5.61-5.68 (m, 1H), 3.26-3.24 (m, 2H), 2.39-2.35 (m, 2H), 1.58-1.53 (m, 2H), 1.33-1.24 (m, 6H), 0.90-0.87 (m, 3H).

^{13}C NMR (101 MHz, $CDCl_3$) δ 175.0, 138.3, 108.2, 97.0, 69.6, 34.2, 31.4, 28.6, 28.1, 22.6, 19.4, 14.2.

HRMS (ESI): Calculated for $C_{12}H_{16}O_2Na$ [M+Na$^+$]: 215.1043 found: 215.1038.

IR (neat): 2929, 2858, 2240, 1802, 1634, 1466, 1389, 1329, 1282, 1260, 1222, 1099, 980, 942, 880, 839, 744, 673 cm^{-1}.

ii. Synthesis of 4-substituted furanones

4-Methylfuran-2(5*H*)-one

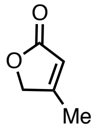

MW (g/mol): 98.10 **Molecular formula:** $C_5H_6O_2$

Prepared according to reported procedure.[12] To a stirred solution of citraconic anhydride (13.4 mmol, 1.5 g, 1.0 equiv.) in THF (30 mL, 0.45 M) at 0 °C was added $NaBH_4$ (34.4 mmol, 1.3 g, 2.5 equiv.) in one portion. The reaction was allowed to stir at 0 °C for 2 h. The reaction was quenched with water, acidified with dilute HCl (1 M) and extracted with EtOAc (3 times). The organic layers were washed with water, a saturated aqueous solution of brine and dried over anhydrous $MgSO_4$. After filtration, the crude mixture was concentrated *in vacuo*. The purification of the residue over silica gel (*n*-Hexane/Ethyl acetate = 70:30) furnished 4-methylfuran-2(5*H*)-one (6.1 mmol, 600 mg, 46%) as a colourless oil. *The spectral data were in agreement with the literature.*[12]

^{1}H NMR (400 MHz, $CDCl_3$) δ 5.78-5.72 (m, 1H), 4.67-4.64 (m, 2H), 2.07 (s, 3H).

^{13}C NMR (101 MHz, $CDCl_3$) δ 174.2, 166.3, 116.2, 74.0, 14.0.

iii. Synthesis of 3-substituted furanones

3-Bromo-2,5-dihydrofuran-2-one

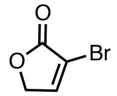

MW (g/mol): 162.97 **Molecular formula:** $C_4H_3BrO_2$

An oven-dried 100 mL round-bottom flask under an argon atmosphere was charged with a solution of 2(*5H*)-furanone (2.0 mL, 28.2 mmol, 1.0 equiv.) in benzene (30 mL), followed by bromine (1.7 mL, 31.0 mmol, 1.1 equiv.). The solution was stirred for 24 h at rt and was then cooled to 0 °C. Pyridine (6.8 mL, 85 mmol, 3.0 equiv.) was added dropwise and the mixture was stirred at 0 °C for 2 h and at rt for another 2 h. The reaction mixture was cooled to 0 °C, filtered on a silica gel pad to remove the pyridine salts and rinsed with cold diethyl ether. The filtrate was concentrated under vacuum, and the crude residue was purified by flash column chromatography over silica gel. The desired product was obtained as a beige solid (3.76 g, 23.1 mmol, 82% yield). *The spectral data were in agreement with the literature.*[13]

^{1}H NMR (400 MHz, $CDCl_3$) δ 7.64 (t, J = 1.9 Hz, 1H, H_3), 4.86 (d, J = 1.9 Hz, 2H, H_2).
^{13}C NMR (101 MHz, $CDCl_3$) δ 169.0 (C_q, C_1), 149.4 (CH, C_3), 113.1 (C_q, C_4), 71.7 (CH_2, C_2).

Suzuki coupling for the synthesis of 3-aryl furanones:

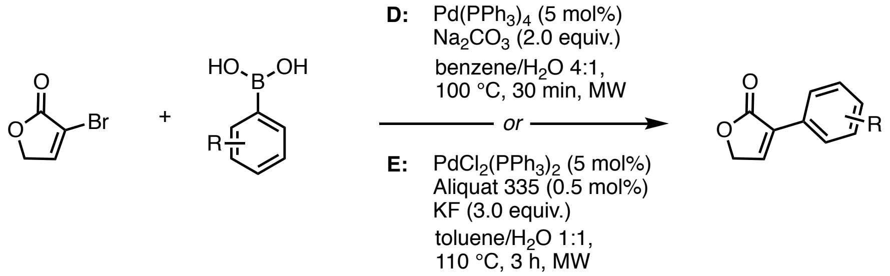

General procedure D:

An oven-dried microwave tube under an argon atmosphere was charged with a solution of vinyl bromide (163 mg, 1.0 mmol, 1.0 equiv.) in benzene (3.0 mL), followed by the desired boronic acid (1.5 equiv.), $Pd(PPh_3)_4$ (0.05 equiv.) and a solution of Na_2CO_3 (2.0 equiv.) in distilled H_2O (1.0 mL). The reaction mixture was heated in a sealed vessel at 100 °C for 30 min under

microwave irradiation (300 W). A saturated aqueous solution of brine was then added, and the aqueous phase was extracted three times with EtOAc. The combined organic layers were finally dried over anhydrous $MgSO_4$, filtered and concentrated under reduced pressure. The crude residue was purified by flash column chromatography over silica gel to afford the desired product.

General procedure E:

An oven-dried microwave tube under an argon atmosphere was charged with a solution of vinyl bromide (163 mg, 1.0 mmol, 1.0 equiv.) in 1:1 mixture of toluene/H_2O (4.0 mL), followed by the desired boronic acid (2.0 equiv.), $PdCl_2(PPh_3)_2$ (0.05 equiv.), KF (3.0 equiv.) and aliquat® 336 (0.005 equiv.). The reaction mixture was heated in a sealed vessel at 110 °C for 3H under microwave irradiation (300 W). A saturated aqueous solution of brine was then added, and the aqueous phase was extracted three times with EtOAc. The combined organic layers were dried over anhydrous $MgSO_4$, filtered and concentrated under reduced pressure. The crude residue was finally purified by flash column chromatography over silica gel to afford the desired product.

3-Phenyl-2,5-dihydrofuran-2-one

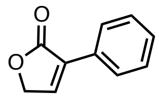

MW (g/mol): 160.17 **Molecular formula:** $C_{10}H_8O_2$

Prepared according to ***general procedure D***. The desired product was obtained as a yellow solid (88.0 mg, 0.55 mmol, 55% yield) after purification by flash column chromatography over silica gel (*n*-Hexane/Ethyl acetate = 75:25). *The spectral data were in agreement with the literature.*[13]

^{1}H NMR (400 MHz, $CDCl_3$) δ 7.86 (m, 1H), 7.84 (m, 1H), 7.65 (t, *J* = 2.0 Hz, 1H), 7.46-7.36 (m, 3H), 4.92 (d, *J* = 2.0 Hz, 2H).

^{13}C NMR (101 MHz, $CDCl_3$) δ 172.3, 144.5, 131.6, 129.5, 129.3, 128.7, 127.0, 69.6.

3-(4-Ethylphenyl)furan-2(5*H*)-one

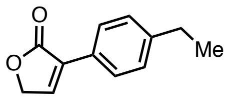

MW (g/mol): 188.23 **Molecular formula:** $C_{12}H_{12}O_2$

Prepared according to ***general procedure D***. The desired product was obtained as yellow oil (111 mg, 0.59 mmol, 59% yield) after purification by flash column chromatography over silica gel (*n*-Hexane/Ethyl acetate = 100:0 to 85:15). *The spectral data were in agreement with the literature.*[13]

^{1}H NMR (400 MHz, CDCl$_3$) δ 7.83-7.73 (m, 2H), 7.59 (t, *J* = 2.0 Hz), 7.27-7.24 (m), 4.92 (d, *J* = 2.0 Hz), 2.68 (q, *J* = 7.6 Hz), 1.25 (t, *J* = 7.6 Hz).

^{13}C NMR (101 MHz, CDCl$_3$) δ 172.5, 145.9, 143.4, 131.8, 128.3, 127.1, 69.6, 28.9, 15.6.

3-(4-Fluorophenyl)furan-2(5*H*)-one

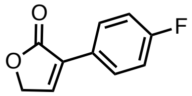

MW (g/mol): 178.16 **Molecular formula:** $C_{10}H_7FO_2$

Prepared according to ***general procedure E***. The desired product was obtained as a yellow solid (77.0 mg, 0.43 mmol, 43% yield) after purification by flash column chromatography over silica gel (*n*-Hexane/Diethyl ether = 100:0 to 80:20). *The spectral data were in agreement with the literature.*[13]

^{1}H NMR (400 MHz, CDCl$_3$) δ 7.91-7.83 (m, 2H, H_6), 7.62 (t, *J* = 2.0 Hz, 1H, H_3), 7.16-7.07 (m, 2H, H_7), 4.94 (d, *J* = 2.0 Hz, 2H, H_2).

^{13}C NMR (101 MHz, CDCl$_3$) δ 172.2, 163.3 (d, *J* = 251.1 Hz), 143.8, 130.7, 128.9, 125.7, 115.7 (d, *J* = 22.4 Hz), 69.5.

iv. Synthesis of lactams

1-Tosylpyrrolidin-2-one

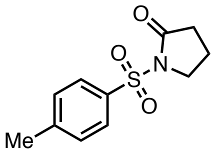

MW (g/mol): 239.29 **Molecular formula:** $C_{11}H_{13}NO_3S$

Adapting from literature procedure.[14] To a solution of NaH (18.7 mmol, 750 mg, 1.1 equiv.) in dry Et_2O (85 mL, 0.2 M) cooled to 0 °C, 2-pyrrolidone was added (17 mmol, 1.35 mL, 1.0 equiv.) under argon atmosphere. The reaction was allowed to stir at the same temperature for 1 h, then TsCl (18.7 mmol, 3.6 g, 1.1 equiv.) was added in one portion and the reaction mixture was allowed to warm to rt and the stirring is maintaining for 16 h. The reaction mixture was quenched with a saturated aqueous solution of NH_4Cl. The mixture was extracted with EtOAc (3 times). The combined organic layers were washed with a saturated aqueous solution of brine, dried over anhydrous $MgSO_4$, filtrated and concentrated under reduced pressure. The crude residue was finally purified by flash column chromatography over silica gel (Pentane/Ethyl acetate = 70:30) to afford the title compound (3.4 g, 9.99 mmol, 59%). *The spectral data are consistent with the literature.*[14]

^{1}H NMR (400 MHz, $CDCl_3$) δ 7.93 (d, *J* = 8.3 Hz, 2H), 7.34 (d, *J* = 7.9 Hz, 2H), 3.92-3.87 (m, 2H), 2.45-2.41 (m, 5H), 2.11-2.03 (m, 2H).

^{13}C NMR (101 MHz, $CDCl_3$) δ 173.5, 145.3, 135.2, 129.8, 128.2, 47.4, 32.3, 21.8, 18.3.

3-(Phenylselanyl)-1-tosylpyrrolidin-2-one

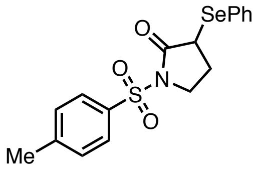

MW (g/mol): 394.36 **Molecular formula:** $C_{17}H_{17}NO_3SSe$

Adapting from literature procedure.[14] To a flame-dried flask charged with *i*Pr_2NEt (11.6 mmol, 1.6 mL, 1.1 equiv.) and anhydrous THF (52 mL, 0.2 M). *n*-BuLi (2.5 M in hexane, 12.6 mmol, 5.1 mL, 1.2 equiv.) was slowly added dropwise at –78 °C. After 30 min, a solution of (10.5 mmol, 2.5 g, 1.0 equiv.) in THF (10 mL) was added *via* cannula. After 1 h, PhSeCl (12.6 mmol, 2.4 g, 1.2 equiv.) was added in one portion. The reaction mixture was allowed to warm to rt and the stirring was maintained for 2 h. The reaction was quenched with a saturated aqueous solution of NH_4Cl, extracted three times with EtOAc. The combined organic layers were washed with a saturated aqueous solution of brine, dried over anhydrous $MgSO_4$, filtered and concentrated *in vacuo*. The crude residue was finally purified by flash column chromatography over silica gel (Pentane/EtOAc = 70:30) to afford the title compound (870 mg, 2.20 mmol, 21%). *The spectral data are consistent with the literature.*[14]

^{1}H NMR (400 MHz, $CDCl_3$) δ 7.90 (d, *J* = 8.3 Hz, 2H), 7.43-7.41 (m, 2H), 7.36-7.30 (m, 3H), 7.21-7.18 (m, 2H), 3.82-3.79 (m, 2H), 3.60-3.55 (m, 1H), 2.46 (s, 3H), 2.15-1.96 (m, 2H).

1-Tosyl-1,5-dihydro-2*H*-pyrrol-2-one

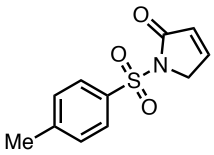

MW (g/mol): 237.27 **Molecular formula:** $C_{11}H_{11}NO_3S$

A solution of 3-(phenylselanyl)-1-tosylpyrrolidin-2-one (2.2 mmol, 870 mg, 1.0 equiv.) in EtOAc (6 mL, 0.35 M) was cooled to –20 °C. An aqueous solution of H_2O_2 (30%, 1.3 mL) was added and the reaction mixture was allowed to stir at the same temperature for 4 h. The reaction mixture was allowed to warm to rt, quenched with a saturated aqueous solution of NH_4Cl, extracted

three times with EtOAc, dried over anhydrous $MgSO_4$, filtered and concentrated *in vacuo*. The residue was purified by flash chromatography over silica gel (Pentane/Ethyl acetate = 10:90) to afford the title compound (380 mg, 1.60 mmol, 72%). *The spectral data are consistent with the literature.*[14]

^{1}H NMR (400 MHz, $CDCl_3$) δ 7.96, *J* = 8.5 Hz, 2H), 7.30 (d, *J* = 8.5 Hz, 2H), 7.22-7.20 (m, 1H), 6.03(dt, *J* = 6.1, 1.9 Hz, 1H), 4.46 (t, *J* = 2 Hz, 2H), 2.39 (s, 3H).

1-Benzyl-1,5-dihydro-2*H*-pyrrol-2-one

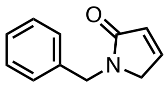

MW (g/mol): 173.22 **Molecular formula:** $C_{11}H_{11}NO$

Adapting from literature procedure.[15] To a solution of 2,5-dimethoxy-2,5-dihydrofuran (19.2 mmol, 2.3 mL, 1 equiv.) and benzyl amine (19.2 mmol, 2.1 mL, 1 equiv.) in water (75 mL, 0.26 M), 9.7 mL of concentrated HCl (10%) was added. The reaction mixture was stirred at rt for 4 h. The mixture was carefully neutralized by the slowly addition of solid $NaHCO_3$ and then extracted three times with CH_2Cl_2. The combined organic layers were washed with a saturated aqueous solution of brine, dried over anhydrous $MgSO_4$, filtrated and concentrated *in vacuo*. The residue was purified by flash chromatography over silica gel (Pentane/Ethyl acetate = 20:80) to give the title compound (6.5 mmol, 1.13 g, 34%). *The spectral data are consistent with the literature.*[15]

^{1}H NMR (400 MHz, $CDCl_3$) δ 7.41-7.20 (m, 5H), 7.04 (dt, *J* = 6.0, 1.8 Hz, 1H), 6.21 (dt, *J* = 6.1, 1.9 Hz, 1H), 4.63 (s, 2H), 3.86 (t, *J* = 1.8 Hz, 2H).

4. C3-olefination

a. General procedure for the C3-olefinaiton (general procedure F):

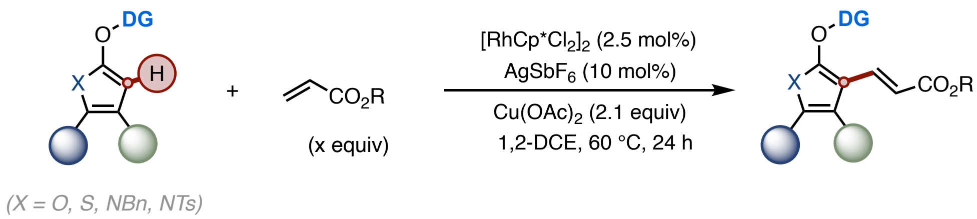

A microwave tube (10 mL) equipped with a stirring bar was flame-dried under *vacuum* and cooled under argon. $[Cp^*RhCl_2]_2$ (3.1 mg, 0.005 mmol, 0.025 equiv.), $AgSbF_6$ (6.9 mg, 0.02 mmol, 0.1 equiv.) and $Cu(OAc)_2$ (76.3 mg, 0.42 mmol, 2.1 equiv.) were introduced into the reaction vessel. A solution of dienol carbamate (carbonate or ester) (1.0 equiv.) in dry solvent (1 mL, 0.2 M) was added followed by the corresponding olefin (287 µL, 2 mmol, 10 equiv.). The reaction vessel was flushed with argon, sealed and allowed to stir at 60 °C for 24 h. The reaction mixture was cooled to rt, diluted with CH_2Cl_2 and the resulting solution was filtered through a short pad of Celite® and concentrated under reduced pressure. The residue was purified by flash chromatography over silica gel to afford the desired product.

Failed olefin partners

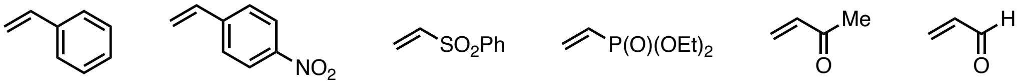

Failed substrates

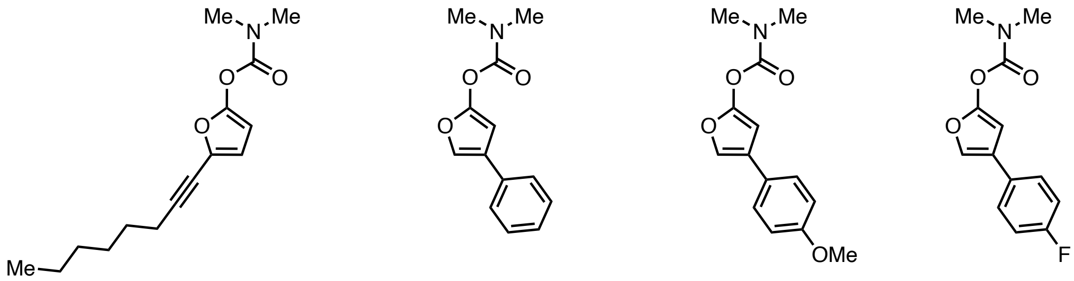

b. Characterization data

Methyl (*E*)-3-(2-((dimethylcarbamoyl)oxy)furan-3-yl)acrylate (2a)

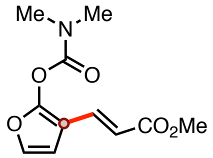

MW (g/mol): 239.23 **Molecular formula:** $C_{11}H_{13}NO_5$

Synthesized according to ***general procedure F***. The desired product was obtained as a yellow oil (43.1 mg, 0.18 mmol, 90% yield) after purification by flash column chromatography over silica gel (*n*-Hexane/Ethyl acetate = 95:5 to 90:10).

^{1}H NMR (400 MHz, $CDCl_3$) δ 7.39 (d, *J* = 15.9 Hz, 1H), 7.08 (d, *J* = 2.4 Hz, 1H), 6.51 (d, *J* = 2.4 Hz, 1H), 6.06 (d, *J* = 15.9 Hz, 1H), 3.74 (s, 3H), 3.10 (s, 3H), 3.00 (s, 3H).

^{13}C NMR (101 MHz, $CDCl_3$) δ 167.5, 152.1, 151.4, 137.4, 133.3, 116.8, 108.8, 105.8, 51.6, 37.2, 36.8.

HRMS (ESI): Calculated for $C_{11}H_{13}NO_5Na$ [$M+Na^+$]: 262.06859 found: 262.06860.

IR (neat): 2951, 1750, 1714, 1645, 1609, 1435, 1382, 1318, 1265, 1231, 1138, 1082, 983, 893, 859, 742, 678, 656 cm^{-1}.

Ethyl (*E*)-3-(2-((dimethylcarbamoyl)oxy)furan-3-yl)acrylate (2b)

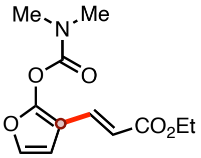

MW (g/mol): 239.23 **Molecular formula:** $C_{11}H_{13}NO_5$

Synthesized according to ***general procedure F***. The desired product was obtained as a yellow oil (40.6 mg, 0.17 mmol, 85% yield) after purification by flash column chromatography over silica gel (*n*-Hexane/Ethyl acetate = 95:5 to 90:10).

^{1}H NMR (400 MHz, CDCl$_3$) δ 7.38 (d, *J* = 15.8 Hz, H7), 7.07 (d, *J* = 2.4 Hz, H1), 6.51 (d, *J* = 2.4 Hz, H2), 6.05 (d, *J* = 15.8 Hz, H8), 4.19 (q, *J* = 7.1 Hz, 2H, H10), 3.09 (s, 3H, H6), 2.99 (s, 3H, H6), 1.27 (t, *J* = 7.1 Hz, 3H, H11).

^{13}C NMR (101 MHz, CDCl$_3$) δ 167.0, 152.1, 151.3, 137.3, 133.0, 117.2, 108.8, 105.7, 60.3, 37.1, 36.8, 14.3.

HRMS (ESI): Calculated for $C_{12}H_{14}NO_5Na$ [M+Na$^+$]: 276.0848 found: 276.0812.

IR (neat): 2939, 1748, 1705, 1643, 1380, 1312, 1262, 1229, 1179, 1133, 1081, 1032, 979, 900, 858, 829, 739 cm^{-1}.

Butyl (*E*)-3-(2-((dimethylcarbamoyl)oxy)furan-3-yl)acrylate (2c)

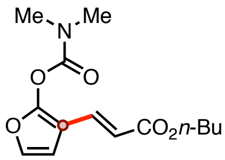

MW (g/mol): 281.31 **Molecular formula:** $C_{14}H_{19}NO_5$

Synthesized according to ***general procedure F***. The desired product was obtained as a yellow oil (50.6 mg, 0.18 mmol, 91% yield) after purification by flash column chromatography over silica gel (*n*-Hexane/Ethyl acetate = 95:5 to 80:20).

Scale-up procedure: A round-bottom flask (250 mL) equipped a condenser, a stirring bar was flame-dried under *vacuum* and cooled under argon. [Cp*RhCl$_2$]$_2$ (155 mg, 0.25 mmol, 0.025 equiv.), AgSbF$_6$ (345 mg, 1.00 mmol, 0.1 equiv.) and Cu(OAc)$_2$ (3.82 g, 21.0 mmol, 2.1 equiv.) were introduced into the reaction vessel. The flask was then evacuated and filled with argon (3 cycles). A solution of dienol carbamate 1c (1.56 g, 10 mmol, 1.0 equiv.) in dry degassed solvent (50 mL, 0.2 M) was added followed by the corresponding olefin (14 mL, 100 mmol, 10 equiv.). Following this, the reaction vessel was allowed to stir at 60 °C for 24 h. The reaction mixture was cooled to rt, diluted with CH_2Cl_2 and the resulting solution was filtered through a short pad of Celite® and concentrated under reduced pressure. The residue was purified by flash chromatography over silica gel to afford the desired product. The desired

product was obtained as a yellow oil (2.11 g, 7.51 mmol, 75% yield) after purification by flash column chromatography over silica gel (*n*-Hexane/Ethyl acetate = 95:5 to 80:20).

^{1}H NMR (400 MHz, CDCl$_3$) δ 7.38 (d, *J* = 15.8 Hz, 1H), 7.08 (d, *J* = 2.4 Hz, 1H), 6.51 (d, *J* = 2.4 Hz, 1H), 6.06 (d, *J* = 15.9 Hz, 1H), 4.15 (t, *J* = 6.7 Hz, 2H), 3.10 (s, 3H), 3.00 (s, 3H), 1.67-1.61 (m, 2H), 1.42-1.36 (m, 2H), 0.93 (t, *J* = 7.4 Hz, 3H).

^{13}C NMR (101 MHz, CDCl$_3$) δ 167.2, 152.1, 151.4, 137.4, 133.0, 117.3, 108.8, 105.8, 64.3, 37.1, 36.8, 30.8, 19.2, 13.8.

HRMS (ESI): Calculated for $C_{14}H_{19}NO_5Na$ [M+Na$^+$]: 304.1155 found: 304.1145.

IR (neat): 2959, 1749, 1707, 1643, 1380, 1313, 1261, 1229, 1176, 1134, 1081, 1063, 1041, 980, 898, 859, 829, 741, 676, 655 cm^{-1}

Butyl (*E*)-3-(2-((diethylcarbamoyl)oxy)furan-3-yl)acrylate

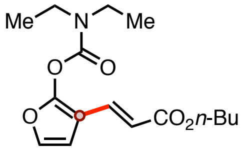

MW (g/mol): 309.36 **Molecular formula:** $C_{16}H_{23}NO_5$

Synthesized according to ***general procedure F***. The desired product was obtained as a yellow oil (37.1 mg, 0.12 mmol, 61% yield) after purification by flash column chromatography over silica gel (*n*-Hexane/Ethyl acetate = 95:5 to 80:20).

^{1}H NMR (400 MHz, CDCl$_3$) δ 7.40 (d, *J* = 15.7 Hz, 1H), 7.10 (d, *J* = 2.4 Hz, 1H), 6.52 (d, *J* = 2.4 Hz, 1H), 6.07 (d, *J* = 15.8 Hz, 1H), 4.16 (t, *J* = 6.7 Hz, 2H), 3.41 (dq, *J* = 26.9, 7.1 Hz, 4H), 1.68-1.62 (m, 2H), 1.44-1.37 (m, 2H), 1.25 (dt, *J* = 25.4, 7.0 Hz, 6H), 0.94 (t, *J* = 7.4 Hz, 3H).

^{13}C NMR (101 MHz, CDCl$_3$) δ 167.2, 151.5, 151.6, 137.4, 133.2, 117.3, 108.9, 105.8, 64.3, 43.0, 42.5, 30.9, 19.3, 14.2, 13.8, 13.2.

HRMS (ESI): Calculated for $C_{16}H_{23}NO_5Na$ [M+Na$^+$]: 332.1468 found: 332.1492.

IR (neat): 2961, 2361, 1746, 1710, 1645, 1610, 1525, 1459, 1407, 1383, 1313, 1260, 1234, 1216, 1177, 1139, 1081, 1025, 977, 948, 931, 859, 785, 742, 674, 652 cm^{-1}.

Butyl (*E*)-3-(2-((diisopropylcarbamoyl)oxy)furan-3-yl)acrylate

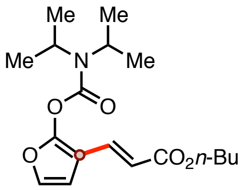

MW (g/mol): 337.41 **Molecular formula:** $C_{18}H_{27}NO_5$

Synthesized according to ***general procedure F***. The desired product was obtained as an orange oil (35.1 mg, 0.10 mmol, 52% yield) after purification by flash column chromatography over silica gel (*n*-Hexane/Ethyl acetate = 95:5 to 80:20).

^{1}H NMR (400 MHz, $CDCl_3$) δ 7.44-7.35 (d, *J* = 15.8 Hz, 1H), 7.10 (dd, *J* = 2.3, 0.7 Hz, 1H), 6.52 (dd, *J* = 2.4, 0.5 Hz, 1H), 6.06 (d, *J* = 15.8 Hz, 1H), 4.15 (t, *J* = 6.6 Hz, 2H), 4.10 (dt, *J* = 14.3, 6.7 Hz, 2H), 1.68-1.61 (m, 2H), 1.43-1.39 (m, 2H), 1.36-1.28 (m, 12H), 0.93 (t, *J* = 7.4 Hz, 3H).

^{13}C NMR (101 MHz, $CDCl_3$) δ 167.2, 151.6, 150.9, 137.4, 133.2, 117.2, 108.9, 105.8, 64.3, 47.7, 47.3, 30.9, 21.4, 20.3, 19.3, 13.8.

HRMS (ESI): Calculated for $C_{18}H_{27}NO_5Na$ [$M+Na^+$]: 360.1781 found: 360.1778.

IR (neat): 2961, 2361, 2341, 1737, 1711, 1645, 1525, 1417, 1304, 1268, 1232, 1206, 1176, 1139, 1082, 1037, 978, 957, 912, 861, 732 cm^{-1}.

(*E*)-3-(3-Butoxy-3-oxoprop-1-en-1-yl)furan-2-yl pyrrolidine-1-carboxylate

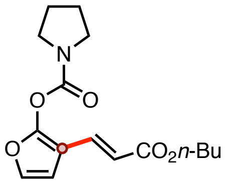

MW (g/mol): 307.35 **Molecular formula:** $C_{16}H_{21}NO_5$

Synthesized according to ***general procedure F***. The desired product was obtained as a brown oil (43.6 mg, 0.142 mmol, 71% yield) after purification by flash column chromatography over silica gel (*n*-Hexane/Ethyl acetate = 90:10).

^{1}H NMR (400 MHz, CDCl$_3$) δ 7.42 (d, *J* = 15.8 Hz, 1H), 7.09 (dd, *J* = 2.4, 0.7 Hz, 1H), 6.52 (d, *J* = 2.4 Hz, 1H), 6.07 (d, *J* = 15.8 Hz, 1H), 4.16 (t, *J* = 6.7 Hz, 2H), 3.58 (t, *J* = 6.6 Hz, 2H), 3.47 (t, *J* = 6.6 Hz, 2H), 2.03-1.89 (m, 4H), 1.69-1.61 (m, 2H), 1.45-1.36 (m, 2H), 0.94 (t, *J* = 7.4 Hz, 3H).
^{13}C NMR (101 MHz, CDCl$_3$) δ 167.3, 151.5, 150.3, 137.3, 133.3, 117.2, 108.8, 105.7, 64.3, 47.0, 46.9, 30.9, 25.8, 25.0, 19.3, 13.8.
HRMS (ESI): Calculated for $C_{16}H_{21}NO_5Na$ [M+Na$^+$]: 330.1312 found: 330.1323.
IR (neat): 2959, 2876, 2363, 1753, 1708, 1644, 1525, 1462, 1436, 1381, 1312, 1276, 1227, 1172, 1140, 1083, 1035, 998, 964, 913, 897, 856, 813, 742, 664, 642 cm^{-1}.

(*E*)-3-(3-Butoxy-3-oxoprop-1-en-1-yl)furan-2-yl morpholine-4-carboxylate

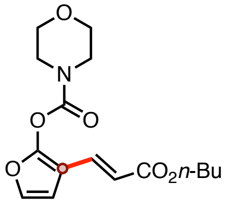

MW (g/mol): 323.34 **Molecular formula:** $C_{16}H_{21}NO_6$

Synthesized according to ***general procedure F***. The desired product was obtained as a brown oil (45.3 mg, 0.14 mmol, 70% yield) after purification by flash column chromatography over silica gel (*n*-Hexane/Ethyl acetate = 90:10 to 70:30).

^{1}H NMR (400 MHz, CDCl$_3$) δ 7.38 (d, *J* = 15.8 Hz, 1H), 7.10 (dd, *J* = 2.4, 0.7 Hz, 1H), 6.53 (dd, *J* = 2.4, 0.6 Hz, 1H), 6.07 (d, *J* = 15.9 Hz, 1H), 4.16 (t, *J* = 6.7 Hz, 2H), 3.79-3.71 (m, 4H), 3.71 3.63 (m, 2H), 3.59-3.51 (m, 2H), 1.71-1.59 (m, 2H), 1.47-1.33 (m, 2H), 0.94 (t, *J* = 7.4 Hz, 3H).
^{13}C NMR (101 MHz, CDCl$_3$) δ 167.2, 151.0, 150.9, 137.6, 132.8, 117.6, 109.0, 106.0, 66.5, 66.4, 64.4, 45.3, 44.6, 30.8, 19.3, 13.8.
HRMS (ESI): Calculated for $C_{16}H_{21}NO_6Na$ [M+Na$^+$]: 346.1261 found: 346.1258.
IR (neat): 2960, 1746, 1707, 1644, 1439, 1403, 1314, 1271, 1212, 1175, 1140, 1116, 1082, 1050, 1030, 974, 919, 896, 854, 734, 654 cm^{-1}.

tert-Butyl (_E_)-3-(2-((dimethylcarbamoyl)oxy)furan-3-yl)acrylate (2d)

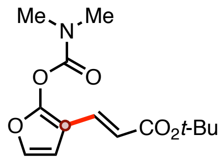

MW (g/mol): 281.21 **Molecular formula:** $C_{14}H_{19}NO_5$

Synthesized according to ***general procedure F***. The desired product was obtained as a yellow oil (41.1 mg, 0.15 mmol, 73% yield) after purification by flash column chromatography over silica gel (*n*-Hexane/Ethyl acetate = 95:5 to 90:10).

^{1}H NMR (400 MHz, CDCl$_3$) δ 7.31 (d, *J* = 15.8 Hz, 1H), 7.07 (d, *J* = 2.4 Hz, 1H), 6.50 (d, *J* = 2.4 Hz, 1), 6.00 (d, *J* = 15.8 Hz, 1H), 3.09 (s, 3H), 3.00 (s, 3H), 1.49 (s, 9H).

^{13}C NMR (101 MHz, CDCl$_3$) δ 166.5, 152.3, 151.2, 137.3, 132.0, 119.1, 108.9, 105.8, 80.4, 37.2, 36.8, 28.3.

HRMS (ESI): Calculated for $C_{14}H_{19}NO_5Na$ [M+Na$^+$]: 304.1155 found: 304.1159.

IR (neat): 2930, 1750, 1703, 1644, 1368, 1319, 1260, 1234, 1132, 1080, 980, 900, 858, 831, 819, 742, 674, 653 cm^{-1}.

Benzyl (_E_)-3-(2-((dimethylcarbamoyl)oxy)furan-3-yl)acrylate (2e)

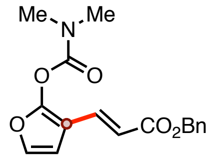

MW (g/mol): 315.32 **Molecular formula:** $C_{17}H_{17}NO_5$

Synthesized according to ***general procedure F***. The desired product was obtained as a sticky yellow solid (31.5 mg, 0.1 mmol, 50% yield) after purification by flash column chromatography over silica gel (*n*-Hexane/Ethyl acetate = 95:5 to 90:10).

^{1}H NMR (400 MHz, CDCl$_3$) δ 7.36 (dt, *J* = 15.8, 0.7 Hz, 1H), 7.30-7.21 (m, 5H), 6.99 (dd, *J* = 2.4, 0.7 Hz, 1H), 6.42 (dd, *J* = 2.4, 0.6 Hz, 1H), 6.03 (d, *J* = 15.8 Hz, 1H), 5.12 (s, 2H), 3.00 (s, 3H), 2.90 (s, 3H).

^{13}C NMR (101 MHz, CDCl$_3$) δ 166.9, 152.1, 151.5, 137.4, 136.2, 133.7, 128.6, 128.3, 128.2, 116.8, 108.8, 105.8, 66.2, 37.1, 36.8.

HRMS (ESI): Calculated for $C_{17}H_{17}NO_5Na$ [M+Na$^+$]: 338.0999 found: 338.0994.

IR (neat): 1749, 1708, 1643, 1497, 1455, 1379, 1312, 1262, 1230, 1168, 1135, 1081, 981, 909, 727, 697, 655 cm^{-1}.

Butyl (*E*)-3-(5-butyl-2-((dimethylcarbamoyl)oxy)furan-3-yl)acrylate (2f)

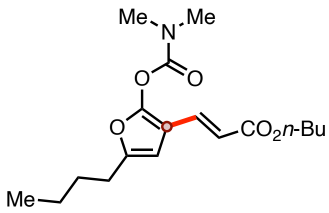

MW (g/mol): 337.42 **Molecular formula:** $C_{18}H_{27}NO_5$

Synthesized according to ***general procedure F***. The desired product was obtained as a yellow oil (55.3 mg, 0.16 mmol, 82% yield) after purification by flash column chromatography over silica gel (*n*-Hexane/Ethyl acetate= 95:5).

^{1}H NMR (400 MHz, CDCl$_3$) δ 7.36 (d, *J* = 15.8 Hz, 1H), 6.10 (s, 1H), 5.99 (d, *J* = 15.8 Hz, 1H), 4.15 (t, *J* = 6.7 Hz, 2H), 3.10 (s, 3H), 3.00 (s, 3H), 2.52 (t, *J* = 7.6 Hz, 2H), 1.66-1.55 (m, 4H), 1.42-1.32 (m, 4H), 0.93 (t, *J* = 7.4 Hz, 3H), 0.90 (t, *J* = 7.4 Hz, 3H).

^{13}C NMR (101 MHz, CDCl$_3$) δ 167.4, 152.4, 151.5, 149.9, 133.5, 116.5, 106.3, 103.4, 64.2, 37.2, 36.8, 30.9, 29.6, 27.7, 22.2, 19.3, 13.8.

HRMS (ESI): Calculated for $C_{18}H_{27}NO_5Na$ [M+Na$^+$]: 360.17814 found: 360.17697.

IR (neat): 2959, 2933, 2874, 1751, 1707, 1644, 1591, 1465, 1381, 1305, 1259, 1227, 1175, 1136, 1107, 1063, 1025, 978, 911, 858, 829, 729, 648 cm^{-1}.

Butyl (*E*)-3-(2-((dimethylcarbamoyl)oxy)-5-phenylfuran-3-yl)acrylate (2g)

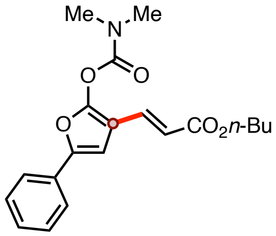

MW (g/mol): 357.40 **Molecular formula:** $C_{20}H_{23}NO_5$

Synthesized according to ***general procedure F***. The desired product was obtained as a yellow oil (55.0 mg, 0.15 mmol, 77% yield) after purification by flash column chromatography over silica gel (*n*-Hexane/Ethyl acetate = 90:10).

^{1}H NMR (400 MHz, $CDCl_3$) δ 7.51 (d, *J* = 7.1 Hz, 2H), 7.35 (d, *J* = 15.8 Hz, 1H), 7.28 (t, *J* = 7.6 Hz, 2H), 7.20-7.16 (m, 1H), 6.67 (s, 1H), 6.05 (d, *J* = 15.8 Hz, 1H), 4.10 (t, *J* = 6.7 Hz, 2H), 3.06 (s, 3H), 2.95 (s, 3H), 1.62-1.55 (m, 2H), 1.37-1.31 (m, 2H), 0.87 (t, *J* = 7.4 Hz, 3H).

^{13}C NMR (101 MHz, $CDCl_3$) δ 167.3, 152.2, 150.9, 148.6, 133.0, 129.6, 128.8, 128.0, 123.7, 117.4, 108.0, 103.1, 64.4, 37.3, 36.9, 30.9, 19.3, 13.9.

HRMS (ESI): Calculated for $C_{20}H_{24}NO_5$ [$M+H^+$]: 358.16490 found: 358.16507.

IR (neat): 2960, 2933, 2874, 1757, 1717, 1644, 1598, 1450, 1382, 1307, 1242, 1176, 1140, 1063, 1022, 983, 860, 762, 698 cm^{-1}.

Butyl (*E*)-3-(2-((dimethylcarbamoyl)oxy)-5-(*p*-tolyl)furan-3-yl)acrylate (2h)

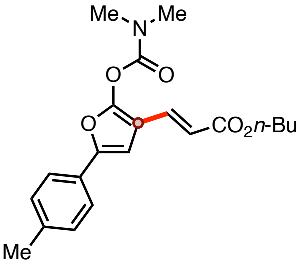

MW (g/mol): 371.43 **Molecular formula:** $C_{21}H_{25}NO_5$

Synthesized according to ***general procedure F***. The desired product was obtained as a yellow oil (48.3 mg, 0.13 mmol, 65% yield) after purification by flash column chromatography over silica gel (*n*-Hexane/Ethyl acetate = 90:10).

^{1}H NMR (400 MHz, CDCl$_3$) δ 7.49 (d, *J* = 7.7 Hz, 2H), 7.43 (d, *J* = 15.8 Hz, 1H), 7.17 (d, *J* = 7.7 Hz, 2H), 6.69 (s, 1H), 6.13 (d, *J* = 15.8 Hz, 1H), 4.18 (t, *J* = 6.7 Hz, 2H), 3.15 (s, 3H), 3.05 (s, 3H), 2.35 (s, 3H), 1.70-1.64 (m, 2H), 1.46–1.39 (m, 2H), 0.96 (t, *J* = 7.4 Hz, 3H).

^{13}C NMR (101 MHz, CDCl$_3$) δ 167.3, 152.3, 150.7, 148.9, 137.9, 133.1, 129.5, 127.0, 123.7, 117.3, 107.9, 102.3, 64.4, 37.3, 36.9, 30.9, 21.4, 19.3, 13.9.

HRMS (ESI): Calculated for $C_{21}H_{26}NO_5$ [M+H$^+$]: 372.18055 found: 372.18064.

IR (neat): 2958, 2873, 2361, 2342, 1753, 1709, 1644, 1586, 1504, 1457, 1379, 1305, 1240, 1173, 1136, 1064, 1042, 982, 925, 818, 742, 689 cm^{-1}.

Butyl (*E*)-3-(2-((dimethylcarbamoyl)oxy)-5-(4-(trifluoromethyl)phenyl)furan-3-yl)acrylate (2i)

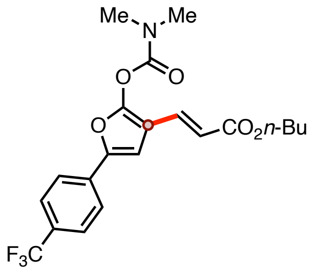

MW (g/mol): 425.40 **Molecular formula:** $C_{21}H_{22}F_3NO_5$

Synthesized according to ***general procedure F***. The desired product was obtained as a brown oil (77.4 mg, 0.18 mmol, 91% yield) after purification by flash column chromatography over silica gel (*n*-Hexane/Ethyl acetate = 90:10).

^{1}H NMR (400 MHz, CDCl$_3$) δ 7.66 (d, *J* = 8.2 Hz, 2H), 7.59 (d, *J* = 8.1 Hz, 2H), 7.39 (d, *J* = 15.9 Hz, 1), 6.87 (s, 1H), 6.15 (d, *J* = 15.9 Hz, 1H), 4.18 (t, *J* = 6.7 Hz, 2H), 3.15 (s, 3H), 3.04 (s, 3H), 1.70-1.62 (m, 2H), 1.44-1.38 (m, 2H), 0.95 (t, *J* = 7.4 Hz, 3H, H_{19}).

^{13}C NMR (101 MHz, CDCl$_3$) δ 167.1, 152.0, 151.5, 147.0, 132.8, 132.5, 129.5 (q, *J* = 32.7 Hz), 125.8 (q, *J* = 3.8 Hz), 124.1 (q, *J* = 272.1 Hz), 123.7, 122.8, 118.0, 108.3, 64.5, 37.3, 36.9, 30.8, 19.3, 13.8.

HRMS (ESI): Calculated for $C_{21}H_{22}F_3NO_5Na$ [M+Na$^+$]: 448.13423 found: 448.13433.

IR (neat): 2961, 2875, 1753, 1709, 1645, 1619, 1567, 1497, 1567, 1497, 1460, 1380, 1322, 1267, 1240, 1166, 1120, 1068, 1015, 980, 910, 843, 826, 731, 674, 664, 648 cm^{-1}.

Butyl (*E*)-3-(5-cyclohexyl-2-((dimethylcarbamoyl)oxy)furan-3-yl)acrylate (2j)

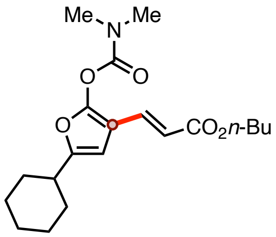

MW (g/mol): 363.45 **Molecular formula:** $C_{20}H_{29}NO_5$

Synthesized according to ***general procedure F***. The desired product was obtained as a brown oil (66.1 mg, 0.18 mmol, 91% yield) after purification by flash column chromatography over silica gel (*n*-Hexane/Ethyl acetate = 95:5).

^{1}H NMR (400 MHz, CDCl$_3$) δ 7.36 (d, *J* = 15.8, 1H), 6.07-6.06 (m, 1H), 6.00 (d, *J* = 15.8 Hz, 1H), 4.15 (t, *J* = 6.7 Hz, 2H), 3.10 (s, 3H), 3.00 (s, 3H), 2.00-1.96 (m, 1H), 1.79-1.73 (m, 2H), 1.73-1.56 (m, 4H), 1.45-1.21 (m, 8H), 0.93 (t, *J* = 7.4 Hz, 3H).

^{13}C NMR (101 MHz, CDCl$_3$) δ 167.5, 155.7, 152.4, 149.8, 133.6, 116.4, 106.1, 101.5, 64.2, 37.2, 37.0, 36.8, 31.0, 30.9, 26.1, 25.8, 19.3, 13.8.

HRMS (ESI): Calculated for $C_{20}H_{29}NO_5Na$ [M+Na$^+$]: 386.1938 found: 386.1933.

IR (neat): 2930, 2855, 2361, 1751, 1707, 1643, 1450, 1381, 1301, 1256, 1236, 1136, 1063, 984, 913, 829, 799, 730, 649 cm^{-1}.

Butyl (*E*)-3-(5-cyclopropyl-2-((dimethylcarbamoyl)oxy)furan-3-yl)acrylate (2k)

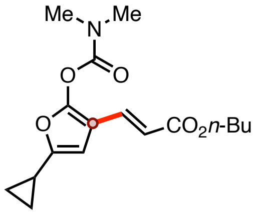

MW (g/mol): 321.37 **Molecular formula:** $C_{17}H_{23}NO_5$

Synthesized according to ***general procedure F***. The desired product was obtained as a brown oil (41.8 mg, 0.13 mmol, 65% yield) after purification by flash column chromatography over silica gel (*n*-Hexane/Ethyl acetate = 99:1 to 95:5).

^{1}H NMR (400 MHz, CDCl$_3$) δ 7.33 (d, *J* = 15.8 Hz, 1H), 6.06 (s, 1H), 5.97 (d, *J* = 15.8 Hz, 1H), 4.14 (t, *J* = 6.7 Hz, 2H), 3.08 (s, 3H), 2.99 (s, 3H), 1.80-1.75 (m, 1H), 1.66-1.61 (m, 2H), 1.41-1.36 (m, 2H), 0.93 (t, *J* = 7.4 Hz, 3H), 0.85-0.81 (m, 2H), 0.74-0.70 (m, 2H).

^{13}C NMR (101 MHz, CDCl$_3$) δ 167.4, 152.42, 152.37, 149.5, 133.3, 116.5, 106.5, 102.2, 64.2, 37.1, 36.8, 30.9, 19.2, 13.8, 8.6, 6.4.

HRMS (ESI): Calculated for $C_{17}H_{24}NO_5$ [M+H$^+$]: 322.16490 found: 322.16509.

IR (neat): 2959, 2874, 1751, 1708, 1643, 1593, 1457, 1378, 1304, 1252, 1233, 1173, 1134, 1062, 1025, 982, 954, 856, 826, 741, 693, 652 cm^{-1}.

Butyl (*E*)-3-(2-((dimethylcarbamoyl)oxy)-5-phenethylfuran-3-yl)acrylate (2l)

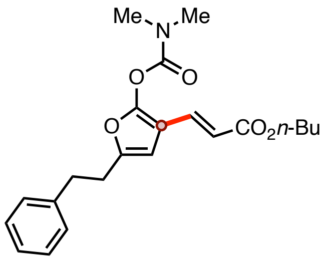

MW (g/mol): 385.46 **Molecular formula:** $C_{22}H_{27}NO_5$

Synthesized according to ***general procedure F***. The desired product was obtained as a yellow oil (60.1 mg, 0.16 mmol, 78% yield) after purification by flash column chromatography over silica gel (*n*-Hexane/Ethyl acetaet = 95:5 to 80:20).

^{1}H NMR (400 MHz, CDCl$_3$) δ 7.44 (d, *J* = 15.8 Hz, 1H), 7.37-7.32 (m, 2H), 7.28-7.21 (m, 3H), 6.17 (s, 1H), 6.05 (d, *J* = 15.8 Hz, 1H), 4.23 (t, *J* = 6.7 Hz, 2H), 3.18 (s, 3H), 3.09 (s, 3H), 3.02-2.97 (m, 2H), 2.94-2.88 (m, 2H), 1.75-1.68 (m, 2H), 1.51-1.44 (m, 2H), 1.01 (t, *J* = 7.4 Hz, 3H).

^{13}C NMR (101 MHz, CDCl$_3$) δ 167.4, 152.5, 150.4, 150.1, 140.8, 133.4, 128.6, 128.5, 126.4, 116.8, 106.5, 104.2, 64.4, 37.3, 36.9, 33.9, 30.9, 30.1, 19.3, 13.9.

HRMS (ESI): Calculated for $C_{22}H_{28}NO_5$ [M+H$^+$]: 386.19620 found: 386.19651.

IR (neat): 2959, 2873, 2360, 2341, 2253, 1751, 1706, 1644, 1591, 1382, 1308, 1258, 1224, 1176, 1136, 1064, 977, 908, 859, 829, 728, 699, 648 cm^{-1}.

Butyl (*E*)-3-(2-((dimethylcarbamoyl)oxy)-4-methylfuran-3-yl)acrylate (2m)

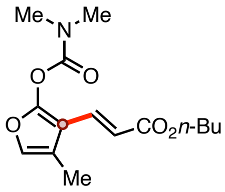

MW (g/mol): 295.34 **Molecular formula:** $C_{15}H_{21}NO_5$

Synthesized according to ***general procedure F***. The desired product was obtained as a yellow oil (36.0 mg, 0.12 mmol, 61% yield) after purification by flash column chromatography over silica gel (*n*-Hexane/Ethyl acetate = 95:5).

^{1}H NMR (400 MHz, CDCl$_3$) δ 7.35 (d, *J* = 16.3 Hz, 1H), 6.84 (q, *J* = 1.4 Hz, 1H), 6.09 (d, *J* = 16.2 Hz, 1H), 4.11 (t, *J* = 6.7 Hz, 2H), 3.06 (s, 3H), 2.95 (s, 3H), 2.06 (d, *J* = 1.3 Hz, 3H), 1.62-1.57 (m, 2H), 1.37-1.32 (m, 2H), 0.89 (t, *J* = 7.4 Hz, 3H).

^{13}C NMR (101 MHz, CDCl$_3$) δ 167.6, 152.3, 151.9, 133.9, 133.7, 120.5, 116.9, 105.6, 64.4, 37.2, 36.9, 30.9, 19.3, 13.9, 10.6.

HRMS (ESI): Calculated for $C_{15}H_{21}NO_5Na$ [M+Na$^+$]: 318.1312 found: 318.1310.

IR (neat): 2928, 1752, 1710, 1644, 1619, 1459, 1377, 1306, 1175, 1138, 1099, 1046, 857, 831, 741 cm^{-1}.

Butyl (*E*)-3-(2-((dimethylcarbamoyl)oxy)-5-(*o*-tolyl)furan-3-yl)acrylate (2n)

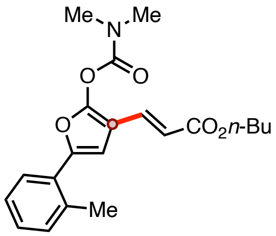

MW (g/mol): 371.43 **Molecular formula:** $C_{21}H_{25}NO_5$

Synthesized according to ***general procedure F***. The desired product was obtained as a yellow oil (49.8 mg, 0.13 mmol, 67% yield) after purification by flash column chromatography over silica gel (*n*-Hexane/Ethyl acetate = 90:10).

^{1}H NMR (400 MHz, $CDCl_3$) δ 7.64-7.58 (m, 1H), 7.43 (d, *J* = 15.8 Hz, 1H), 7.23-7.18 (m, 3H), 6.63 (s, 1H), 6.15 (d, *J* = 15.8 Hz, 1H), 4.19 (t, *J* = 6.7 Hz, 2H), 3.15 (s, 3H), 3.04 (s, 3H), 2.48 (s, 3H), 1.71-1.64 (m, 2H), 1.45-1.40 (m, 2H), 0.96 (t, *J* = 7.4 Hz, 3H).

^{13}C NMR (101 MHz, $CDCl_3$) δ 167.3, 152.2, 150.8, 148.3, 134.9, 133.1, 131.2, 128.9, 128.0, 127.1, 126.1, 117.2, 107.5, 106.9, 64.4, 37.2, 36.9, 30.9, 21.8, 19.3, 13.8.

HRMS (ESI): Calculated for $C_{21}H_{25}NO_5Na$ [M+Na$^+$]: 394.16249 found: 394.16249.

IR (neat): 3064, 2958, 2873, 2360, 2342, 1752, 1708, 1644, 1459, 1378, 1308, 1258, 1235, 1173, 1134, 1063, 1026, 980, 924, 859, 826, 759, 720, 692, 652 cm^{-1}.

Butyl (*E*)-3-(5-(4-bromophenyl)-2-((dimethylcarbamoyl)oxy)furan-3-yl)acrylate (2o)

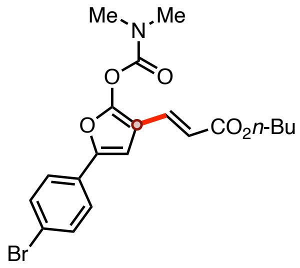

MW (g/mol): 436.30 **Molecular formula:** $C_{20}H_{22}BrNO_5$

Synthesized according to ***general procedure F***. The desired product was obtained as a brown oil (80.3 mg, 0.18 mmol, 92% yield) after purification by flash column chromatography over silica gel (*n*-Hexane/Ethyl acetate = 90:10).

^{1}H NMR (400 MHz, $CDCl_3$) δ 7.50-7.42 (m, 4H), 7.33 (d, *J* = 15.8 Hz, 1H), 6.74 (s, 1H), 6.11 (d, *J* = 15.8 Hz, 1H), 4.17 (t, *J* = 6.7 Hz, 2H), 3.13 (s, 3H), 3.03 (s, 3H), 1.68-1.62 (m, 2H), 1.44-1.38 (m, 2H), 0.94 (t, *J* = 7.4 Hz, 3H).

^{13}C NMR (101 MHz, $CDCl_3$) δ 167.1, 152.0, 151.0, 147.5, 132.7, 131.9, 128.5, 125.1, 121.7, 117.6, 108.1, 103.7, 64.4, 37.2, 36.8, 30.8, 19.2, 13.8.

HRMS (ESI): Calculated for $C_{20}H_{23}BrNO_5$ [M+H$^+$]: 437.07877 found: 437.07902.

IR (neat): 2958, 2361, 1751, 1707, 1644, 1481, 1378, 1313, 1301, 1267, 1239, 1174, 1135, 1071, 980, 910, 859, 823, 802, 730, 678 cm^{-1}.

Butyl (*E*)-3-(2-((dimethylcarbamoyl)oxy)-5-(4-nitrophenyl)furan-3-yl)acrylate (2p)

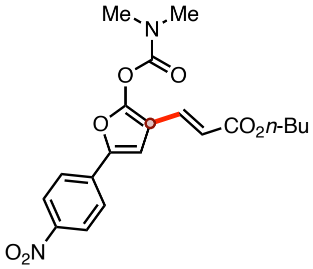

MW (g/mol): 402.40 **Molecular formula:** $C_{20}H_{22}N_2O_7$

Synthesized according to ***general procedure F***. The desired product was obtained as a brown oil (74.0 mg, 0.18 mmol, 92% yield) after purification by flash column chromatography over silica gel (*n*-Hexane/Ethyl acetate = 90:10 to 80:20).

^{1}H NMR (400 MHz, $CDCl_3$) δ 8.18 (d, *J* = 8.9 Hz, 2H), 7.67 (d, *J* = 8.9 Hz, 2H), 7.38 (d, *J* = 15.9 Hz, 1H), 6.97 (s, 1H), 6.14 (d, *J* = 15.9 Hz, 1H), 4.17 (t, *J* = 6.7 Hz, 2H), 3.15 (s, 3H), 3.05 (s, 3H), 1.68-1.62 (m, 2H), 1.42-1.38 (m, 2H), 0.94 (t, *J* = 7.4 Hz, 3H).

^{13}C NMR (101 MHz, $CDCl_3$) δ 166.9, 152.1, 151.7, 146.7, 146.1, 135.2, 132.1, 124.3, 123.8, 118.4, 108.7, 107.2, 64.5, 37.3, 36.9, 30.8, 19.2, 13.8.

HRMS (ESI): Calculated for $C_{20}H_{23}N_2O_7$ [M+H$^+$]: 403.14998 found: 403.14992.

IR (neat): 3118, 2961, 2874, 1751, 1704, 1644, 1594, 1564, 1508, 1465, 1378, 1336, 1272, 1136, 1066, 1045, 974, 926, 857, 820, 753, 693, 660 cm^{-1}.

Methyl (*E*)-4-(4-(3-butoxy-3-oxoprop-1-en-1-yl)-5-((dimethylcarbamoyl)oxy)furan-2-yl)benzoate (2q)

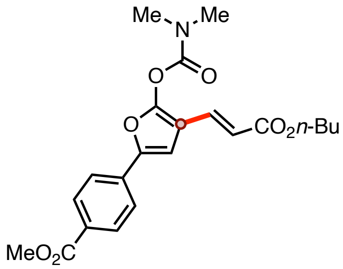

MW (g/mol): 415.44 **Molecular formula:** $C_{22}H_{25}NO_7$

Synthesized according to ***general procedure F***. The desired product was obtained as a dark yellow oil (77.3 mg, 0.19 mmol, 93% yield) after purification by flash column chromatography over silica gel (*n*-Hexane/Ethyl acetate = 90:10 to 80:20).

^{1}H NMR (400 MHz, $CDCl_3$) δ 8.01 (d, *J* = 8.5 Hz, 2H), 7.63 (d, *J* = 8.5 Hz, 2H), 7.40 (d, *J* = 16.0 Hz, 1H), 6.89 (s, 1H), 6.14 (d, *J* = 15.8 Hz, 1H), 4.17 (t, *J* = 6.7 Hz, 2H), 3.90 (s, 3H), 3.15 (s, 3H), 3.04 (s, 3H), 1.69-1.62 (m, 2H), 1.44-1.38 (m, 2H), 0.94 (t, *J* = 7.4 Hz, 3H).

^{13}C NMR (101 MHz, $CDCl_3$) δ 167.1, 166.7, 152.0, 151.5, 147.5, 133.5, 132.6, 130.1, 129.1, 123.3, 117.9, 108.3, 105.4, 64.5, 52.2, 37.3, 36.9, 30.8, 19.3, 13.8.

HRMS (ESI): Calculated for $C_{22}H_{25}NO_7Na$ [M+Na$^+$]: 438.15232 found: 438.15214.

IR (neat): 2956, 2874, 1753, 1711, 1644, 1604, 1581, 1435, 1379, 1273, 1175, 1135, 1109, 1064, 1043, 1016, 979, 916, 858, 823, 770, 731, 698, 679, 648 cm^{-1}.

Butyl (*E*)-3-(2-((dimethylcarbamoyl)oxy)-5-(thiophen-2-yl)furan-3-yl)acrylate (2r)

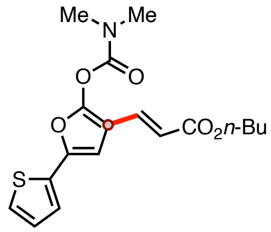

MW (g/mol): 363.43 **Molecular formula:** $C_{18}H_{21}NO_5S$

Synthesized according to ***general procedure F***. The desired product was obtained as a brown oil (21.8 mg, 0.06 mmol, 30% yield) after purification by flash column chromatography over silica gel (*n*-Hexane/Ethyl acetate = 90:10).

^{1}H NMR (400 MHz, CDCl$_3$) δ 7.40 (d, *J* = 15.8 Hz, 1H), 7.25-7.22 (m, 2H), 7.02 (dd, *J* = 4.8, 3.9 Hz, 1H), 6.60 (s, 1H), 6.11 (d, *J* = 15.9 Hz, 1H), 4.18 (t, *J* = 6.7 Hz, 2H), 3.15 (s, 3H), 3.04 (s, 3H), 1.69-1.64 (m, 2H), 1.45-1.41 (m, 2H), 0.96 (t, *J* = 7.4 Hz, 3H).

^{13}C NMR (101 MHz, CDCl$_3$) δ 167.2, 152.1, 150.4, 144.4, 132.8, 132.4, 127.8, 124.9, 123.5, 117.7, 108.0, 103.1, 64.5, 37.3, 36.9, 30.9, 19.3, 13.9.

HRMS (ESI): Calculated for $C_{18}H_{21}NO_5SNa$ [M+Na$^+$]: 386.1033 found: 386.1038.

IR (neat): 2958, 2361, 1753, 1707, 1644, 1459, 1380, 1307, 1262, 1234, 1173, 1135, 979, 908, 857, 824, 731, 698 cm^{-1}.

Butyl (*E*)-3-(2-((dimethylcarbamoyl)oxy)thiophen-3-yl)acrylate (2t)

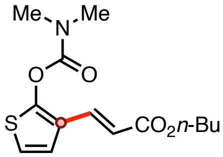

MW (g/mol): 297.37 **Molecular formula:** $C_{14}H_{19}NO_4S$

Synthesized according to ***general procedure F***. The desired product was obtained as a yellow oil (51.7 mg, 0.17 mmol, 87% yield) after purification by flash column chromatography over silica gel (*n*-Hexane/Ethyl acetate = 90:10).

¹H NMR (400 MHz, CDCl₃) δ 7.70 (d, *J* = 15.9 Hz, 1H), 7.00 (d, *J* = 6.0 Hz, 1H), 6.84 (d, *J* = 6.0 Hz, 1H), 6.21 (d, *J* = 15.9 Hz, 1H), 4.19 (t, *J* = 6.6 Hz, 2H), 3.17 (s, 3H), 3.06 (s, 3H), 1.77-1.56 (m, 2H), 1.47-1.38 (m, 2H), 0.95 (t, *J* = 7.4 Hz, 3H).

¹³C NMR (101 MHz, CDCl₃) δ 167.7, 153.3, 152.5, 134.5, 121.7, 121.2, 118.3, 117.0, 64.4, 37.2, 36.6, 30.9, 19.4, 13.9.

HRMS (ESI): Calculated for $C_{14}H_{19}NO_4SNa$ [M+Na$^+$]: 320.0927 found: 320.0922.

IR (neat): 2929, 2360, 1735, 1706, 1627, 1455, 1385, 1297, 1269, 1246, 1160, 1065, 981, 911, 732 cm^{-1}.

Benzyl (*E*)-3-(1-benzyl-2-((dimethylcarbamoyl)oxy)-1*H*-pyrrol-3-yl)acrylate (2u)

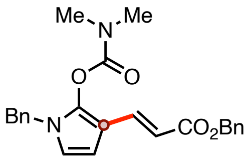

MW (g/mol): 404.47 **Molecular formula:** $C_{21}H_{26}N_2O_4$

Synthesized according to ***general procedure F***. The desired product was obtained as a brown amorphous solid (32.4 mg, 0.08 mmol, 40% yield) after purification by flash column chromatography over silica gel (*n*-Hexane/Ethyl acetate = 80:20 to 70:30).

¹H NMR (400 MHz, CDCl₃) δ 7.46 (d, *J* = 15.8 Hz, 1H), 7.30-7.18 (m, 8H), 7.06 (d, *J* = 6.5 Hz, 2H), 6.24 (dd, *J* = 22.8, 3.5 Hz, 2H), 5.99 (d, *J* = 15.7 Hz, 1H), 5.11 (s, 2H), 4.78 (s, 2H), 2.88 (s, 3H), 2.87 (s, 3H).

¹³C NMR (101 MHz, CDCl₃) δ 167.9, 153.1, 138.0, 136.65, 136.58, 136.4, 128.8, 128.5, 128.1, 128.0, 127.9, 127.4, 117.6, 112.3, 107.8, 104.7, 65.8, 49.5, 37.1, 36.6.

HRMS (ESI): Calculated for $C_{24}H_{24}N_2O_4Na$ [M+Na$^+$]: 427.1628 found: 427.1624.

IR (neat): 2934, 1739, 1703, 1626, 1561, 1510, 1455, 1381, 1296, 1250, 1141, 1065, 1028, 992, 852, 834, 732, 698, 653 cm^{-1}.

Methyl (*E*)-3-(1-benzyl-2-((dimethylcarbamoyl)oxy)-1*H*-pyrrol-3-yl)acrylate (2v)

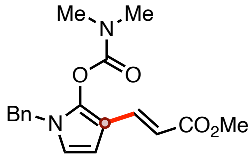

MW (g/mol): 328.37 **Molecular formula:** $C_{18}H_{20}N_2O_4$

Synthesized according to ***general procedure F***. The desired product was obtained as a brown amorphous solid (63.7 mg, 0.19 mmol, 97% yield) after purification by flash column chromatography over silica gel (*n*-Hexane/Ethyl acetate = 80:20 to 70:30).

^{1}H NMR (400 MHz, CDCl$_3$) δ 7.41 (d, *J* = 15.7 Hz, 1H), 7.29-7.17 (m, 3H), 7.07 (d, *J* = 6.5 Hz, 2H), 6.28 (d, *J* = 3.6 Hz, 1H), 6.22 (d, *J* = 3.5 Hz, 1H), 5.94 (d, *J* = 15.8 Hz, 1H), 4.79 (s, 2H), 3.65 (s, 3H), 2.89 (s, 3H).

^{13}C NMR (101 MHz, CDCl$_3$) δ 168.5, 153.0, 137.8, 136.4, 136.2, 128.8, 128.0, 127.4, 117.5, 112.3, 107.8, 104.7, 51.3, 49.5, 37.1, 36.6.

HRMS (ESI): Calculated for $C_{18}H_{20}N_2O_4Na$ [M+Na$^+$]: 351.13153 found: 351.13124.

IR (neat): 2947, 1736, 1699, 1625, 1561, 1509, 1433, 1382, 1303, 1248, 1171, 1140, 1065, 1030, 990, 853, 833, 724, 653 cm^{-1}.

Butyl (*E*)-3-(2-((dimethylcarbamoyl)oxy)-1-tosyl-1*H*-pyrrol-3-yl)acrylate (2w)

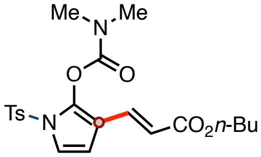

MW (g/mol): 434.51 **Molecular formula:** $C_{21}H_{26}N_2O_6S$

Synthesized according to ***general procedure F***. The desired product was obtained as a brown amorphous solid (79.1 mg, 0.18 mmol, 91% yield) after purification by flash column chromatography over silica gel (*n*-Hexane/Ethyl acetate = 80:20 to 60:40).

^{1}H NMR (400 MHz, CDCl$_3$) δ 7.73 (d, *J* = 8.4 Hz, 2H), 7.29 (dd, *J* = 12.1, 3.8 Hz, 3H), 6.97 (d, *J* = 4.1 Hz, 1H), 6.36 (d, *J* = 4.0 Hz, 1H), 6.05 (d, *J* = 15.8 Hz, 1H), 4.12 (t, *J* = 6.7 Hz, 2H), 3.12 (s, 3H), 2.97 (s, 3H), 2.39 (s, 3H), 1.64-1.59 (m, 2H), 1.41-1.34 (m, 2H), 0.91 (t, *J* = 7.4 Hz, 3H).

^{13}C NMR (101 MHz, $CDCl_3$) δ 167.2, 152.3, 145.7, 136.8, 135.5, 133.7, 130.0, 127.3, 118.1, 117.3, 112.9, 107.9, 64.3, 37.3, 36.8, 30.8, 21.7, 19.2, 13.8.

HRMS (ESI): Calculated for $C_{21}H_{26}N_2O_6SNa$ [M+Na$^+$]: 457.14038 found: 457.14033.

IR (neat): 2959, 2361, 1752, 1707, 1637, 1595, 1493, 1379, 1319, 1267, 1173, 1143, 1089, 1063, 1021, 980, 860, 816, 742, 695, 671 cm^{-1}.

Butyl (*E*)-3-(1-benzyl-2-((dimethylcarbamoyl)oxy)-1*H*-pyrrol-3-yl)acrylate (2x)

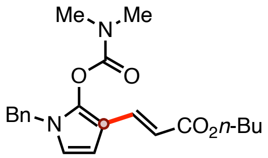

MW (g/mol): 370.45 **Molecular formula:** $C_{21}H_{26}N_2O_4$

Synthesized according to ***general procedure F***. The desired product was obtained as a brown amorphous solid (63.0 mg, 0.17 mmol, 85% yield) after purification by flash column chromatography over silica gel (*n*-Hexane/Ethyl acetate = 80:20 to 70:30).

^{1}H NMR (400 MHz, $CDCl_3$) δ 7.48 (d, *J* = 15.7 Hz, 1H), 7.35-7.28 (m, 3H), 7.16 (d, *J* = 7.1 Hz, 2H), 6.33 (dd, *J* = 22.4, 3.5 Hz, 2H), 6.03 (d, *J* = 15.7 Hz, 1H), 4.88 (s, 2H), 4.15 (t, *J* = 6.7 Hz, 2H), 2.99 (s, 3H), 2.98 (s, 3H), 1.67-1.63 (m, 2H), 1.43-1.40 (m, 2H), 0.94 (t, *J* = 7.4 Hz, 3H).

^{13}C NMR (101 MHz, $CDCl_3$) δ 168.3, 153.2, 137.8, 136.5, 135.9, 128.9, 128.0, 127.5, 117.6, 112.9, 107.9, 104.7, 64.0, 49.6, 37.2, 36.7, 31.0, 19.3, 13.9.

HRMS (ESI): Calculated for $C_{21}H_{26}N_2O_4Na$ [M+Na$^+$]: 393.17848 found: 393.17852.

IR (neat): 2958, 1738, 1699, 1626, 1562, 1510, 1455, 1383, 1299, 1247, 1172, 1141, 1065, 1029, 991, 853, 833, 777, 728, 699, 653 cm^{-1}.

5. C5-olefination

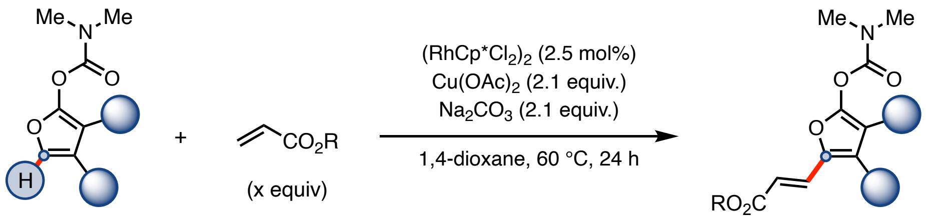

a. General procedure G

An oven-dried microwave tube under an argon atmosphere was charged with the desired carbamate (1.0 equiv.), $[RhCp^*Cl_2]_2$ (0.025 equiv.), Na_2CO_3 (2.0 equiv.), and $Cu(OAc)_2$ (2.0 equiv.). The tube is then sealed and purged with argon. 1,4-dioxane (c = 0.1 M) was then added and the reaction was stirred at 60 ˚C for 24 h. The reaction mixture is then cooled down and filtered through a pad of celite. The filtrate was concentrated under reduced pressure, and the resulting crude residue was purified by flash column chromatography over silica gel to afford the desired product.

Failed coupling partners

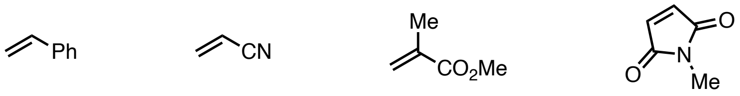

b. Characterization data

Methyl (*E*)-3-(5-((dimethylcarbamoyl)oxy)furan-2-yl)acrylate (3a)

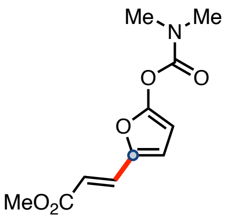

MW (g/mol): 239.29 **Molecular formula:** $C_{11}H_{13}NO_5$

Synthesized according to ***general procedure G***. The desired product was obtained as a yellow oil (24.9 mg, 0.1 mmol, 52% yield) after purification by flash column chromatography over silica gel (*n*-Hexane/Ethyl acetate = 80:20 to 70:30).

^{1}H NMR (400 MHz, CDCl$_3$) δ 7.30 (d, *J* = 15.7 Hz, 1H), 6.57 (d, *J* = 3.5 Hz, 1H), 6.18 (d, *J* = 15.7 Hz, 1H), 5.95 (d, *J* = 3.4 Hz, 1H), 3.76 (s, 3H), 3.10 (s, 3H), 3.02 (s, 3H).

^{13}C NMR (101 MHz, CDCl$_3$) δ 167.7, 153.6, 151.7, 143.9, 131.1, 117.0, 114.1, 95.6, 51.7, 37.2, 36.8.

HRMS (ESI): Calculated for $C_{11}H_{12}NO_5Na$ [M+Na$^+$]: 262.0692 found: 262.0687.

IR (neat): 2941, 1748, 1706, 1641, 1367, 1261, 1142, 1015, 963, 731, 681 cm^{-1}.

Ethyl (*E*)-3-(5-((dimethylcarbamoyl)oxy)furan-2-yl)acrylate (3b)

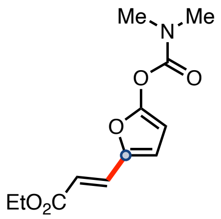

MW (g/mol): 253.25 **Molecular formula:** $C_{12}H_{15}NO_5$

Synthesized according to ***general procedure G***. The desired product was obtained as a brown solid (16.2 mg, 0.064 mmol, 32% yield) after purification by flash column chromatography over silica gel (*n*-Hexane/Ethyl acetate = 75:25).

^{1}H NMR (400 MHz, CDCl$_3$) δ 7.31-7.27 (d, *J* = 15.8 Hz), 6.55 (d, *J* = 2.9 Hz), 6.20-6.16 (d, *J* = 15.7 Hz), 5.94 (d, *J* = 3.0 Hz), 4.20 (q, *J* = 7.0 Hz, 2H), 3.09 (s, 3H), 3.01 (s, 3H), 1.29 (t, *J* = 7.0 Hz, 3H).

^{13}C NMR (101 MHz, CDCl$_3$) δ 167.2, 153.4, 151.7, 143.8, 130.7, 116.7, 114.6, 95.3, 60.3, 37.0, 36.6, 14.3.

HRMS (ESI): Calculated for $C_{12}H_{14}NO_5Na$ [M+Na$^+$]: 276.0848 found: 276.0859.

IR (neat): 2928, 1743, 1701, 1640, 1580, 1537, 1492, 1474, 1437, 1366, 1300, 1246, 1194, 1142, 1019, 961, 835, 783, 730 cm^{-1}.

Butyl (*E*)-3-(5-((dimethylcarbamoyl)oxy)furan-2-yl)acrylate (3c)

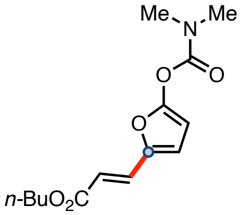

MW (g/mol): 281.30 **Molecular formula:** $C_{14}H_{19}NO_5$

Synthesized according to ***general procedure G***. The desired product was obtained as a brown oil (18.6 mg, 0.066 mmol, 33% yield) after purification by flash column chromatography over silica gel (*n*-Hexane/Ethyl acetate = 75:25).

^{1}H NMR (400 MHz, CDCl$_3$) δ 7.31-7.26 (d, *J* = 15.7 Hz), 6.55 (d, *J* = 3.4 Hz), 6.21-6.17 (d, *J* = 15.7 Hz), 5.95 (d, *J* = 3.4 Hz), 4.18-4.15 (t, 2H), 3.09 (s, 3H), 3.02 (s, 3H), 1.69-1.60 (m, 2H), 1.46-1.36 (m, 2H), 0.96-0.93 (t, 3H).

^{13}C NMR (101 MHz, CDCl$_3$) δ 167.2, 153.4, 151.6, 143.8, 130.6, 116.6, 114.6, 95.3, 64.2, 37.0, 36.6, 30.8, 19.2, 13.7.

HRMS (ESI): Calculated for $C_{14}H_{18}NO_5Na$ [M+Na$^+$]: 304.1161 found: 304.1188.

IR (neat): 2928, 1740, 1699, 1638, 1574, 1533, 1493, 1439, 1411, 1387, 1366, 1297, 1243, 1182, 1144, 1068, 1014, 983, 963, 837, 823, 786, 759, 742, 731, 683, 639 cm^{-1}.

***tert*-Butyl (*E*)-3-(5-((dimethylcarbamoyl)oxy)furan-2-yl)acrylate (3d)**

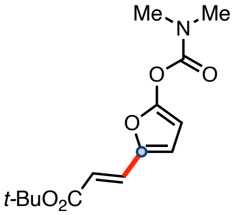

MW (g/mol): 281.30 **Molecular formula:** $C_{14}H_{19}NO_5$

Synthesized according to ***general procedure G***. The desired product was obtained as a brown oil (24.8 mg, 0.088 mmol, 44% yield) after purification by flash column chromatography over silica gel (*n*-Hexane/Ethyl acetate = 75:25).

^{1}H NMR (400 MHz, CDCl$_3$) δ 7.21-7.17 (d, *J* = 15.7 Hz), 6.53 (d, *J* = 3.4 Hz), 6.15-6.11 (d, *J* = 15.7 Hz), 5.93 (d, *J* = 3.4 Hz), 3.09 (s, 3H), 3.02 (s, 3H), 1.50 (s, 9H).

^{13}C NMR (101 MHz, $CDCl_3$) δ 166.4, 153.2, 151.7, 144.0, 129.8, 116.7, 116.0, 95.3, 80.5, 37.0, 36.6, 28.2.

HRMS (ESI): Calculated for $C_{14}H_{18}NO_5Na$ [M+Na$^+$]: 304.1161 found: 304.1158.

IR (neat): 2928, 1750, 1701, 1637, 1597, 1581, 1532, 1454, 1392, 1366, 1309, 1283, 1254, 1207, 1135, 1015, 962, 858, 831, 780, 742, 681, 639 cm^{-1}.

Benzyl (*E*)-3-(5-((dimethylcarbamoyl)oxy)furan-2-yl)acrylate (3e)

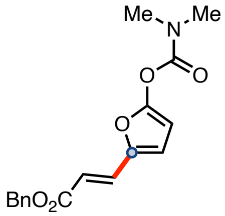

MW (g/mol): 315.32 **Molecular formula:** $C_{17}H_{17}NO_5$

Synthesized according to ***general procedure G***. The desired product was obtained as a yellow sticky solid (25.2 mg, 0.08 mmol, 40% yield) after purification by flash column chromatography over silica gel (*n*-Hexane/Ethyl acetate = 75:25).

^{1}H NMR (400 MHz, $CDCl_3$) δ 7.40-7.30 (m, 5H), 7.36-7.32 (d, *J* = 15.6 Hz), 6.57 (d, *J* = 3.5 Hz), 6.26-6.22 (d, *J* = 15.6 Hz), 5.95 (d, *J* = 3.5 Hz), 5.22 (s, 2H), 3.09 (s, 3H), 3.02 (s, 3H).

^{13}C NMR (101 MHz, $CDCl_3$) δ 166.9, 153.6, 151.6, 143.7, 136.2, 131.2, 128.6, 128.1, 117.0, 114.1, 95.4, 66.2, 37.0, 36.6.

HRMS (ESI): Calculated for $C_{17}H_{16}NO_5Na$ [M+Na$^+$]: 338.1004 found: 338.0996.

IR (neat): 1740, 1700, 1640, 1577, 1533, 1500, 1440, 1366, 1297, 1245, 1194, 1148, 1020, 998, 978, 965, 913, 846, 835, 779, 746, 731, 696, 639 cm^{-1}.

Methyl (*E*)-3-(5-((dimethylcarbamoyl)oxy)-4-methylfuran-2-yl)acrylate (3f)

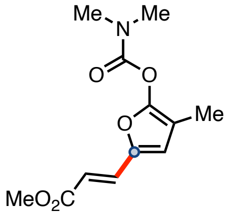

MW (g/mol): 253.25 **Molecular formula:** $C_{12}H_{15}NO_5$

Synthesized according to ***general procedure G***. The desired product was obtained as a red oil (14.7 mg, 0.058 mmol, 29% yield) after purification by flash column chromatography over silica gel (*n*-Hexane/Ethyl acetate = 75:25).

^{1}H NMR (400 MHz, CDCl$_3$) δ 7.26 (d, *J* = 15.7), 6.46 (s), 6.16 (d, *J* = 15.7 Hz), 3.75 (s, 3H), 3.11 (s, 3H), 3.02 (s, 3H), 1.90 (s, 3H).

^{13}C NMR (101 MHz, CDCl$_3$) δ 167.7, 152.4, 149.6, 143.7, 131.0, 119.1, 113.9, 106.1, 51.5, 37.0, 36.7, 8,4.

HRMS (ESI): Calculated for $C_{12}H_{14}NO_5Na$ [M+Na$^+$]: 276.0828 found: 2776.0839.

IR (neat): 2951, 1745, 1709, 1618, 1435, 1387, 1364, 1299, 1263, 1239, 1137, 974, 826, 759, 744 cm^{-1}.

Methyl (*E*)-3-(5-((dimethylcarbamoyl)oxy)-4-phenylfuran-2-yl)acrylate (3g)

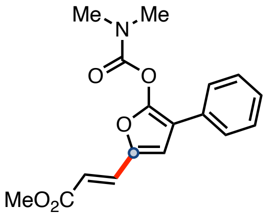

MW (g/mol): 315.32 **Molecular formula:** $C_{17}H_{17}NO_5$

Synthesized according to ***general procedure G***. The desired product was obtained as a yellow oil (20.8 mg, 0.066 mmol, 33% yield) after purification by flash column chromatography over silica gel (pentane/Et$_2$O = 1:0 to 1:1).

^{1}H NMR (400 MHz, CDCl$_3$) δ 7.45 (d, 2H), 7.40-7.27 (m, 3H), 7.35 (d, *J* = 15.7 Hz), 6.85 (s, 1H), 6.27 (d, *J* = 15.7 Hz), 3.78 (s, 3H), 3.16 (s, 3H), 3.04 (s, 3H).

^{13}C NMR (101 MHz, $CDCl_3$) δ 167.5, 152.2, 148.6, 144.4, 130.7, 130.5, 128.8, 127.2, 126.4, 115.7, 115.2, 111.8, 51.7, 37.2, 36.8.

HRMS (ESI): Calculated for $C_{17}H_{16}NO_5Na$ [M+Na$^+$]: 338.1004 found: 338.0993.

IR (neat): 2950, 1748, 1716, 1644, 1621, 1599, 1541, 1493, 1435, 1368, 1305, 1261, 1240, 1196, 1167, 1133, 1010, 967, 795, 759 cm^{-1}.

Methyl (*E*)-3-(5-((dimethylcarbamoyl)oxy)-4-(4-ethylphenyl)furan-2-yl)acrylate (3h)

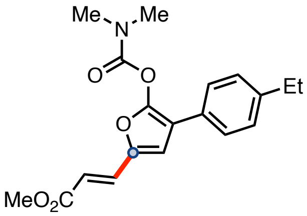

MW (g/mol): 343.37 **Molecular formula:** $C_{19}H_{21}NO_5$

Synthesized according to ***general procedure G***. The desired product was obtained as a yellow oil (19.4 mg, 0.06 mmol, 47% yield) after purification by flash column chromatography over silica gel (*n*-Hexane/Ethyl acetate = 75:25).

^{1}H NMR (400 MHz, $CDCl_3$) δ 7.38-7.36 (d, *J* = 8.2 Hz, 2H), 7.37-7.33 (d, *J* = 15.7 Hz), 7.21 (d, *J* = 8.0 Hz, 2H), 6.83 (s), 6.28-6.24 (d, *J* = 15.7 Hz), 3.78 (s, 3H), 3.16 (s, 3H), 3.04 (s, 3H), 2.68-2.63 (q, *J* = 7.6 Hz, 2H), 1.27-1.23 (t, *J* = 7.6 Hz, 3H).

^{13}C NMR (101 MHz, $CDCl_3$) δ 166.5, 151.2, 147.4, 143.3, 142.3, 129.8, 127.3, 126.8, 125.3, 114.8, 113.9, 110.8, 50.6, 36.2, 35.8, 27.6, 14.5.

HRMS (ESI): Calculated for $C_{19}H_{20}NO_5Na$ [M+Na$^+$]: 366.1317 found: 366.1312.

IR (neat): 2963, 1750, 1716, 1645, 1624, 1608, 1541, 1515, 1435, 1366, 1304, 1261, 1234, 1193, 1164, 1134, 1113, 1008, 967, 818, 758 cm^{-1}.

Methyl (*E*)-3-(5-((dimethylcarbamoyl)oxy)-4-(4-fluorophenyl)furan-2-yl)acrylate (3i)

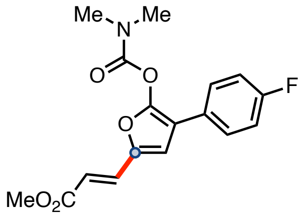

MW (g/mol): 333.31 **Molecular formula:** $C_{17}H_{16}NO_5F$

Synthesized according to ***general procedure G***. The desired product was obtained as a colourless oil (17.0 mg, 0.05 mmol, 51% yield) after purification by flash column chromatography over silica gel (*n*-Hexane/Ethyl acetate = 75:25).

^{1}H NMR (400 MHz, CDCl$_3$) δ 7.43-7.39 (dd, *J* = 8.8, 5.3 Hz, 2H), 7.32 (d, *J* = 15.7 Hz), 7.09-7.05 (t, *J* = 8.7 Hz, 2H), 6.80 (s), 6.29-6.25 (d, *J* = 15.7 Hz), 3.78 (s, 3H), 3.16 (s, 3H), 3.04 (s, 3H).

^{13}C NMR (101 MHz, CDCl$_3$) δ 167.4, 152.1, 148.5, 144.5, 130.6, 128.1, 128.0, 115.9, 115.7, 115.5, 115.3, 111.0, 51.7, 37.2, 36.8.

HRMS (ESI): Calculated for $C_{17}H_{15}NO_5Na$ [M+Na$^+$]: 356.0910 found: 356.09023.

IR (neat): 2948, 1745, 1715, 1644, 1622, 1511, 1366, 1303, 1270, 1195, 1164, 1135, 1009, 965, 820, 801, 755 cm^{-1}.

Methyl 4-((dimethylcarbamoyl)oxy)-5-methyl-7-oxabicyclo[2.2.1]hept-5-ene-2-carboxylate

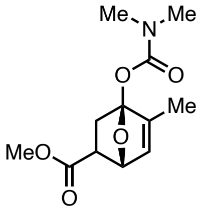

MW (g/mol): 255.27 **Molecular formula:** $C_{12}H_{17}NO_5$

Obtained as a side produce in ***general procedure G*** when using carbamate **1f** and methyl acrylate. The product was obtained as a yellow oil (12.8 mg, 0.05 mmol, 25% yield) after purification by flash column chromatography over silica gel (*n*-Hexane/Ethyl acetate = 80:20).

^{1}H NMR (400 MHz, CDCl$_3$) δ 5.86-5.76 (m, 1H), 5.01 (dd, *J* = 4.7, 2.2 Hz, 1H), 3.65 (s, 3H), 3.39 (dt, *J* = 9.2, 4.3 Hz, 1H), 3.00 (s, 3H), 2.93 (s, 3H), 2.25 (dd, *J* = 11.6, 9.2 Hz, 1H), 1.98 (dd, *J* = 11.4, 4.1 Hz), 1.84 (d, *J* = 1.8 Hz, 3H).

^{13}C NMR (101 MHz, CDCl$_3$) δ 172.2, 153.2, 146.8, 125.8, 111.4, 75.1, 52.0, 47.1, 36.5, 31.4, 11.3.

HRMS (ESI): Calculated for $C_{12}H_{17}NO_5Na$ [M+Na$^+$]: 278.1004 found: 278.1016.

IR (neat): 2923, 1721, 1437, 1394, 1318, 1270, 1152, 1058, 993, 732 cm^{-1}.

6. Post-functionalisation

Ethyl (*E*)-3-(2-oxo-2,5-dihydrofuran-3-yl)acrylate

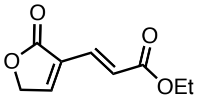

MW (g/mol): 182.18 **Molecular formula:** $C_9H_{10}O_4$

According to the literature procedure.[16] In a microwave tube (10 mL), **2c** (0.2 mmol, 56.2 mg, 1 equiv.) was introduced, then EtOH (0.2 M) and an aqueous solution of HCl (6 M) were introduced and the reaction mixture was heated at 110 °C during 2 h. After cooling at rt, the reaction mixture was quenched with H_2O and extracted three times with EtOAc. The combined organic layers were washed with a saturated aqueous solution of brine, dried over anhydrous $MgSO_4$, filtered and concentrated under reduced pressure. The crude residue was finally purified by flash column chromatography over silica gel (Pentane/EtOAc = 50:50) to afford the title compound (0.11 mmol, 20 mg, 55%) as a yellow liquid.

^{1}H NMR (400 MHz, CDCl$_3$) δ 7.61-7.59 (m, 1H), 7.36 (d, *J* = 15.9 Hz, 1H), 7.03 (d, *J* = 15.9 Hz, 1H), 4.91 (m, 2H), 4.26-4.21 (m, 2H), 1.31 (t, *J* = 7.1 Hz, 3H).
^{13}C NMR (101 MHz, CDCl$_3$) δ 170.9, 166.5, 150.6, 131.3, 128.0, 124.9, 70.0, 60.9, 14.2.
HRMS (ESI): Calculated for $C_9H_{10}O_4Na$ [M+Na$^+$]: 205.0471 found: 205.0476.
IR (neat): 2925, 2852, 1756, 1713, 1655, 1445, 1369, 1342, 1309, 1289, 1267, 1205, 1178, 1089, 1054, 911, 824, 731 cm^{-1}.

Butyl (*E*)-3-(2-hexylfuran-3-yl)acrylate (5)

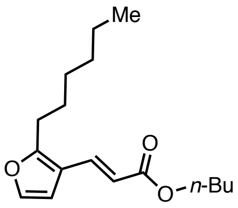

MW (g/mol): 278.39 **Molecular formula:** $C_{17}H_{26}O_3$

According to the literature procedure.[17] A microwave tube (10 mL) equipped with a stirring bar and LiCl (50.9 mg, 1.20 mmol, 6 equiv.) was flame-dried under *vacuum* and cooled under argon.

A solution of butyl (*E*)-3-(2-((dimethylcarbamoyl)oxy)furan-3-yl)acrylate **2c** (56.3 mg, 0.2 mmol, 1 equiv.) and $FeCl_2$ (1.3 mg, 0.010 mmol, 0.05 equiv.) in anhydrous THF (1 mL, 0.2 M) was added. The vial was then evacuated and backfilled with argon five times and the vial was sealed. The reaction mixture was cooled down to 0 °C and a solution of hexylmagnesium bromide (0.25 mL, 2 M in Et_2O) was added dropwise. After 1 h, the reaction mixture was quenched with a saturated aqueous solution of NH_4Cl and the resulting mixture was extracted three times with EtOAc. The combined organic layers were washed with a saturated aqueous solution of brine, dried over anhydrous $MgSO_4$, filtered and concentrated under reduced pressure. The crude residue was finally purified by flash column chromatography over silica gel (Pentane/Diethyl ether = 100:0 to 80:20) to afford the desired product **5** (30 mg, 0.108 mmol, 54%) as a yellow oil.

^{1}H NMR (400 MHz, $CDCl_3$) δ 7.55 (d, *J* = 15.7 Hz, 1H), 7.27 (d, *J* = 2.1 Hz, 1H), 6.48 (d, *J* = 2.1 Hz, 1H), 6.06 (d, *J* = 15.7 Hz, 1H), 4.18 (t, *J* = 6.7 Hz, 2H), 2.72 (t, *J* = 7.5 Hz, 2H), 1.67-1.63 (m, 2H), 1.45-1.40 (m, 2H), 1.33-1.22 (m, 8H), 0.97-0.85 (m, 6H).
^{13}C NMR (101 MHz, $CDCl_3$) δ 167.7, 158.8, 141.8, 135.2, 117.2, 116.2, 107.7, 64.3, 31.6, 31.0, 28.9, 28.6, 26.4, 22.6, 19.3, 14.2, 13.9.
HRMS (ESI): Calculated for $C_{17}H_{26}O_3Na$ [$M+Na^+$]: 301.17742 found: 301.17725.
IR (neat): 2958, 2930, 2872, 2860, 1711, 1638, 1524, 1466, 1381, 1311, 1266, 1230, 1171, 1136, 1064, 1024, 975, 895, 857, 800, 747 cm^{-1}.

Butyl (*E*)-3-(furan-3-yl)acrylate (6)

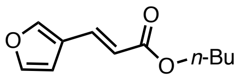

MW (g/mol): 194.23 **Molecular formula:** $C_{11}H_{14}O_3$

According to the literature procedure.[18] A microwave tube (10 mL) equipped with a stirring bar and K_3PO_4 (318 mg, 1.50 ımmol, 7.5 equiv.) was flame-dried under vacuum and cooled under argon. $NiCl_2(PCy_3)_2$ (28 mg, 0.041 mmol, 0.20 equiv.) and a solution of butyl (*E*)-3-(2-((dimethylcarbamoyl)oxy)furan-3-yl)acrylate **2c** (56 mg, 0.20 mmol, 1 equiv.) in anhydrous toluene (0.67 mL, 0.3 M) then 1,1,3,3-tetramethyldisiloxane (92 µL, 0.52 mmol,

2.5 equiv.) were added. The vial was then evacuated and backfilled with argon five times and the vial was sealed. The heterogeneous mixture was allowed to stir at 135 °C for 24 h. The reaction vessel was cooled to rt, diluted with CH_2Cl_2 and the resulting solution was filtered through a short pad of Celite® and concentrated under reduced pressure. The crude residue was purified by flash chromatography over silica gel (Pentane/Et_2O = 100:0 to 75:25) to afford the desired product **6** (30 mg, 0.16 mmol, 77%) as a colorless oil.

^{1}H NMR (400 MHz, $CDCl_3$) δ 7.64 (s, 1H), 7.56 (d, *J* = 15.8 Hz, 1H), 7.42 (d, *J* = 2.1 Hz, 1H), 6.59 (d, *J* = 2.1 Hz, 1H), 6.16 (d, *J* = 15.8 Hz, 1H), 4.19 (t, *J* = 6.7 Hz, 2H), 1.69-1.65 (m, 2H), 1.45-1.40 (m, 2H), 0.95 (t, *J* = 7.4 Hz, 3H).
^{13}C NMR (101 MHz, $CDCl_3$) δ 167.2, 144.6, 144.5, 134.6, 122.8, 118.2, 107.6, 64.5, 30.9, 19.3, 13.9.
HRMS (ESI): Calculated for $C_{11}H_{14}O_3Na$ [M+Na$^+$]: 217.08352 found: 217.08361.
IR (neat): 3126, 2960, 2933, 2874, 1710, 1643, 1511, 1465, 1390, 1465, 1390, 1313, 1267, 1219, 1176, 1154, 1081, 1065, 1021, 977, 871, 795, 737, 670 cm^{-1}.

Butyl (*E*)-3-(2-(4-methoxyphenyl)furan-3-yl)acrylate (7)

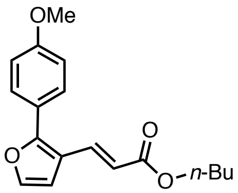

MW (g/mol): 300.35 **Molecular formula:** $C_{18}H_{20}O_4$

According to the literature procedure.[19] A microwave tube (10 mL) equipped with a stirring bar and K_3PO_4 (318 mg, 1.50 mmol, 7.5 equiv.) was flame-dried under *vacuum* and cooled under argon. 4-methoxyphenylboronic acid (121 mg, 0.80 mmol, 4 equiv.), $NiCl_2(PCy_3)_2$ (28.5 mg, 0.041 mmol, 0.20 equiv.) and a solution of butyl (*E*)-3-(2-((dimethylcarbamoyl)oxy)furan-3-yl)acrylate **2c** (56 mg, 0.20 mmol, 1 equiv.) in anhydrous toluene (0.67 mL, 0.3 M) were added. The vial was then evacuated and backfilled with argon five times and the vial was sealed. The heterogeneous mixture was allowed to stir at rt for 1 h, then heated to 135 °C during 24 h. The reaction vessel was cooled to rt, diluted with CH_2Cl_2 and the resulting solution was filtered

through a short pad of Celite® and concentrated under reduced pressure. The crude residue was finally purified by flash column chromatography over silica gel (Pentane/Diethyl ether = 100:0 to 75:25) to afford the desired product **7** (45 mg, 0.15 mmol, 76%) as a colorless oil.

^{1}H NMR (400 MHz, CDCl$_3$) δ 7.83 (d, *J* = 15.6 Hz, 1H), 7.56 (d, *J* = 8.9 Hz, 2H), 7.41 (d, *J* = 2.0, 1H), 6.99 (d, *J* = 8.9 Hz, 2H), 6.65 (d, *J* = 2.0 Hz, 1H), 6.21 (d, *J* = 15.6 Hz, 1H), 4.19 (t, *J* = 6.7 Hz, 2H), 3.86 (s, 3H), 1.71-1.64 (m, 2H), 1.46-1.40 (m, 2H), 0.96 (t, *J* = 7.4 Hz, 3H).
^{13}C NMR (101 MHz, CDCl$_3$) δ 167.5, 160.2, 154.9, 142.2, 136.0, 128.9, 122.9, 118.0, 117.0, 114.5, 109.4, 64.4, 55.5, 30.9, 19.4, 13.9.
HRMS (ESI): Calculated for $C_{18}H_{20}O_4Na$ [M+Na$^+$]: 323.12538 found: 323.12537.
IR (neat): 2959, 2934, 1708, 1631, 1612, 1527, 1498, 1464, 1317, 1255, 1175, 1148, 1118, 1073, 1033, 982, 888, 834, 804, 756, 726, 678, 609 cm^{-1}.

(*E*)-3-(3-Hydroxyprop-1-en-1-yl)furan-2-yl dimethylcarbamate (8)

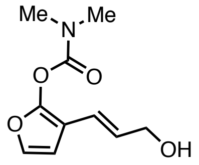

MW (g/mol): 281.31 **Molecular formula:** $C_{14}H_{19}NO_5$

According to the literature procedure.[20] A microwave tube (10 mL) equipped with a stirring bar was flame-dried under *vacuum* and cooled under argon. $Cp_2Zr(H)Cl$ (162 mg, 0.63 mmol, 4 equiv.) was added followed by a solution of butyl (*E*)-3-(2-((dimethylcarbamoyl)oxy)furan-3-yl)acrylate **2c** (56 mg, 0.20 mmol, 1 equiv.) in anhydrous THF (2 mL, 0.1 M). The vial was then evacuated and backfilled with argon five times and the vial was sealed. The heterogeneous mixture was allowed to stir at rt for 4 h. The reaction mixture was quenched with an aqueous solution of HCl (1 M) and the resulting mixture was extracted three times with EtOAc. The combined organic layers were washed with a saturated aqueous solution of brine, dried over anhydrous $MgSO_4$, filtered and concentrated under reduced pressure. The crude residue was finally purified by flash column chromatography over silica gel (Pentane/Diethyl ether = 100:0 to 60:40) to afford the desired product **8** (32 mg, 0.152 mmol, 76%) as a brown oil.

^{1}H NMR (400 MHz, $CDCl_3$) δ 7.03 (d, *J* = 2.3 Hz, 1H), 6.47 (d, *J* = 2.3 Hz, 1H), 6.28 (d, *J* = 15.9 Hz, 1H), 6.04-5.97 (m, 1H), 4.22 (dd, *J* = 5.8, 1.5 Hz, 2H), 3.10 (s, 3H), 3.00 (s, 3H), 1.86 (br s, 1H).

^{13}C NMR (101 MHz, $CDCl_3$) δ 152.8, 148.1, 136.6, 128.1, 119.4, 109.2, 106.4, 63.7, 37.1, 36.8.

HRMS (ESI): Calculated for $C_{10}H_{13}NO_4Na$ [$M+Na^+$]: 234.07368 found: 234.07375.

IR (neat): 3413, 2929, 2859, 1738, 1673, 1618, 1499, 1454, 1382, 1265, 1233, 1142, 1103, 1067, 991, 966, 896, 835, 746, 660 cm^{-1}.

7. DFT calculations

DFT calculations were performed with Gaussian 16 using the ωB97X-D functional, the SMD solvation model, the SDD basis set/effective core potential for Rh, and 6-31G(d,p) for all other atoms. Geometry optimizations, frequency calculations, and IRC calculations were carried out at this level of theory. Unless otherwise noted, all energies reported in the paper are Gibbs free energies derived from unscaled harmonic frequency calculations at 298 °C and 1 atm. Transition states were confirmed by the presence of a single imaginary frequency corresponding to the expected reaction coordinate and by IRC calculations connecting them to the appropriate reactant and product minima.

Cationic path

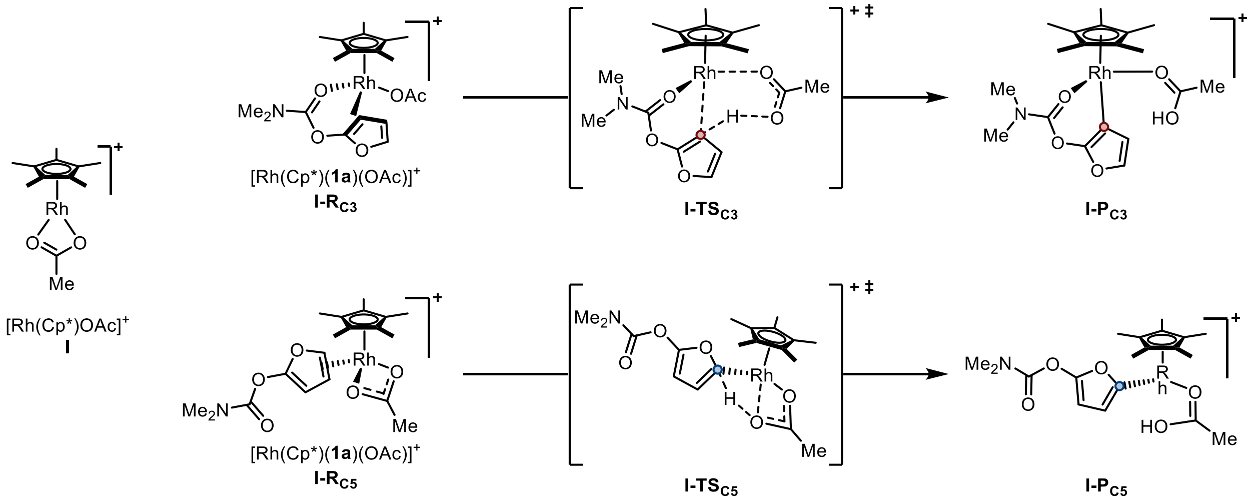

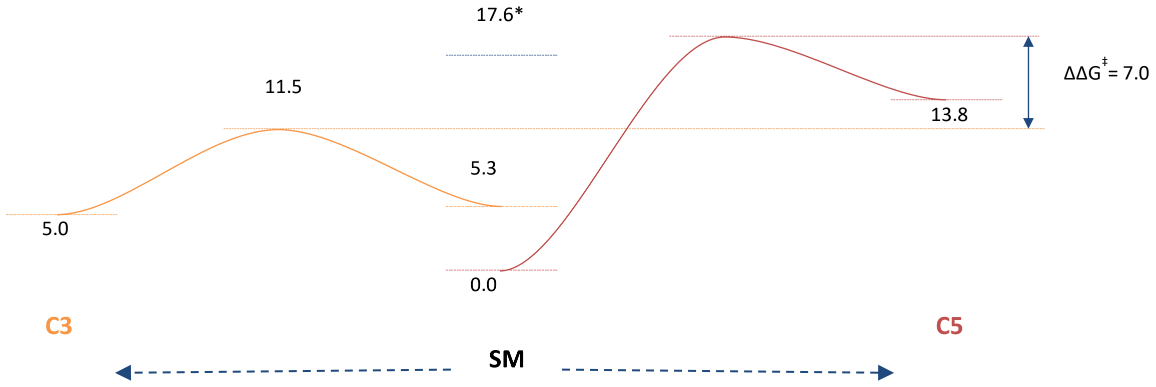

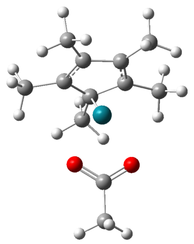

I
E = -728.916761899 Eh

Rh	0.2786	-0.1460	-0.3908
C	-1.6704	-1.0813	-0.3753
C	-1.7845	0.2723	-0.8915
C	-1.1560	-1.0122	0.9497
C	-1.3383	1.1732	0.1161
C	-0.9032	0.3841	1.2537
O	2.2017	-0.9712	0.1626
C	2.7816	0.1146	-0.1625
O	2.0531	1.0794	-0.5625
C	-0.3969	0.9171	2.5477
H	0.0412	1.9086	2.4239
H	-1.2347	0.9957	3.2498
H	0.3517	0.2529	2.9840
C	-1.2907	2.6596	0.0366
H	-1.2955	3.0070	-0.9973
H	-2.1716	3.0696	0.5424
H	-0.4017	3.0532	0.5336
C	-2.2967	0.6257	-2.2452
H	-2.0223	-0.1322	-2.9819
H	-3.3906	0.6775	-2.2092
H	-1.9200	1.5937	-2.5793
C	-2.0460	-2.3114	-1.1280
H	-1.6454	-3.2097	-0.6567
H	-3.1378	-2.3942	-1.1582
H	-1.6877	-2.2659	-2.1596
C	-0.8896	-2.1582	1.8628
H	-0.0911	-1.9249	2.5690
H	-1.7987	-2.3742	2.4344
H	-0.6135	-3.0556	1.3068
C	4.2672	0.2450	-0.1056
H	4.6738	-0.0038	-1.0916
H	4.6843	-0.4461	0.6277
H	4.5479	1.2725	0.1311

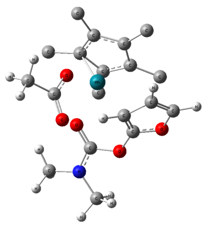

I-R$_{C3}$
E = -1281.35020371 Eh

C	0.31654	-2.700106	-2.405797
C	-0.226397	-1.520491	-2.76781
C	0.494616	-0.484759	-2.058446
C	1.42923	-1.182759	-1.329096
O	1.34404	-2.496474	-1.510858
H	0.121928	-3.730418	-2.657101
H	0.533798	0.584982	-2.26521
O	2.485904	-0.834216	-0.569791
C	2.433968	0.310766	0.172052
O	1.379497	0.931093	0.360889
N	3.616836	0.646061	0.66322
H	-1.045769	-1.372016	-3.455262
C	3.719658	1.752848	1.60635
H	4.418892	2.496918	1.217118
H	2.743492	2.212532	1.744404
H	4.088703	1.378887	2.565529
C	4.829059	-0.126154	0.400394
H	4.9019	-0.977526	1.083709
H	4.845833	-0.480617	-0.629209
H	5.683567	0.532267	0.558528
Rh	-0.615556	0.08722	0.049595
O	-1.210362	1.883403	-0.844087
C	-0.563673	2.7146	-1.599832
O	0.516625	2.501272	-2.155363
C	-1.272249	4.04674	-1.760554
H	-2.314263	3.888465	-2.051204
H	-1.274007	4.572943	-0.800658
H	-0.76816	4.662072	-2.506973
C	-2.568435	-0.004495	0.972555
C	-1.545279	0.292672	1.939248
C	-2.251669	-1.263016	0.368571
C	-0.643378	-0.843984	1.992286
C	-1.083184	-1.807196	1.043042

C	-0.522348	-3.174526	0.855633
H	-0.740387	-3.567598	-0.137654
H	0.557253	-3.197522	1.0133
H	-0.987216	-3.842179	1.58963
C	0.543879	-0.963604	2.886442
H	0.986302	0.013599	3.089596
H	0.233051	-1.401063	3.841294
H	1.308243	-1.608983	2.448347
C	-1.485459	1.512424	2.793958
H	-1.982321	2.352615	2.305375
H	-1.99271	1.317394	3.745271
H	-0.453005	1.794743	3.01021
C	-3.698863	0.902697	0.631423
H	-4.367274	0.452821	-0.103665
H	-4.270392	1.126564	1.537103
H	-3.308887	1.841384	0.224864
C	-3.051556	-1.956038	-0.682362
H	-2.432876	-2.646472	-1.257306
H	-3.856294	-2.527471	-0.20706
H	-3.499618	-1.237969	-1.37196

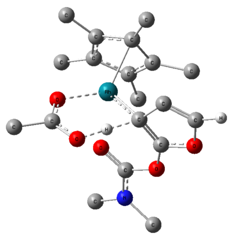

I-TS$_{C3}$
E = -1281.33399280 Eh

C	0.887447	3.382559	1.600592
C	-0.074619	2.475249	1.858833
C	0.371919	1.180985	1.359248
C	1.605147	1.493374	0.839768
O	1.944706	2.772842	0.954948
H	1.014777	4.435617	1.795885
H	0.264963	0.139209	2.181251
O	2.569763	0.790824	0.20856
C	2.383727	-0.514269	-0.123648
O	1.302107	-1.107449	0.007243

N	3.488337	-1.068981	-0.604954
H	-1.009456	2.668869	2.365835
C	3.443111	-2.420073	-1.149554
H	4.058047	-3.085584	-0.537591
H	2.417644	-2.782695	-1.159301
H	3.836042	-2.406043	-2.169691
C	4.766614	-0.368066	-0.685955
H	4.865013	0.143672	-1.648032
H	4.864423	0.353699	0.12272
H	5.56171	-1.109375	-0.593376
Rh	-0.680057	-0.151928	-0.028597
O	-1.161687	-1.611783	1.443902
C	-0.634511	-1.658872	2.591377
O	0.167931	-0.781983	3.034793
C	-0.955422	-2.837961	3.465415
H	-1.978542	-3.174091	3.290375
H	-0.276967	-3.654978	3.198209
H	-0.805968	-2.592151	4.517176
C	-2.645124	-0.238178	-0.917145
C	-1.714334	-0.914192	-1.774492
C	-2.22141	1.128856	-0.767767
C	-0.719072	0.052482	-2.17309
C	-1.026462	1.312171	-1.564921
C	-0.295327	2.596511	-1.77717
H	-0.348784	3.233758	-0.892026
H	0.756155	2.420988	-2.013517
H	-0.745485	3.139346	-2.615074
C	0.453772	-0.22742	-3.051169
H	0.771884	-1.268448	-2.969452
H	0.174974	-0.038138	-4.093482
H	1.300069	0.417967	-2.804871
C	-1.78631	-2.33771	-2.220358
H	-2.321779	-2.952434	-1.494185
H	-2.314639	-2.402805	-3.177674
H	-0.787975	-2.75989	-2.35551
C	-3.842081	-0.849607	-0.273808
H	-4.127516	-0.305771	0.628255
H	-4.682184	-0.814896	-0.976
H	-3.660068	-1.892672	-0.009464
C	-2.956903	2.193957	-0.025279
H	-2.305062	3.036521	0.208716
H	-3.782587	2.560178	-0.645369
H	-3.378033	1.811387	0.906903

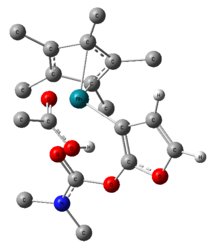

I-P$_{C3}$
E = -1281.35020371 Eh

C	0.554845	3.002032	-2.187792
C	-0.547534	2.425223	-1.649689
C	-0.123372	1.222689	-0.960691
C	1.218642	1.223384	-1.171494
O	1.667387	2.260567	-1.898906
H	0.728182	3.896799	-2.765141
H	0.174083	2.048162	0.860497
O	2.212025	0.341214	-0.859561
C	2.02764	-0.50971	0.170601
O	0.986046	-0.539839	0.855237
N	3.079274	-1.293417	0.381357
H	-1.557668	2.804857	-1.718069
C	3.010946	-2.358265	1.373123
H	3.792896	-2.211893	2.122869
H	2.038092	-2.347519	1.859984
H	3.164316	-3.323543	0.881617
C	4.294839	-1.204255	-0.422648
H	4.190087	-1.775159	-1.350309
H	4.525861	-0.166561	-0.659943
H	5.115457	-1.621978	0.161573
Rh	-1.017585	-0.353069	-0.030879
O	-1.522894	0.786879	1.792533
C	-0.960471	1.745111	2.329314
O	0.013419	2.416168	1.759177
C	-1.3343	2.235741	3.687304
H	-2.17228	1.660641	4.077545
H	-0.470987	2.135292	4.351615
H	-1.5928	3.296523	3.635355
C	-2.658127	-1.84643	0.28789
C	-1.502481	-2.53095	-0.114113
C	-2.96491	-0.840294	-0.731424
C	-1.060228	-1.96577	-1.393083
C	-2.034407	-0.997276	-1.810924

C	-2.074641	-0.293765	-3.12599
H	-2.638423	-0.906332	-3.838145
H	-2.569436	0.675527	-3.045159
H	-1.072375	-0.13635	-3.528342
C	-4.158101	0.056961	-0.708491
H	-5.035538	-0.482975	-1.081622
H	-4.375239	0.391199	0.308515
H	-4.005167	0.936588	-1.337136
C	-3.449132	-2.027612	1.540989
H	-4.430928	-2.449837	1.302038
H	-2.947367	-2.697168	2.241432
H	-3.611019	-1.067084	2.038084
C	-0.774554	-3.610645	0.615806
H	-0.970695	-4.57701	0.139003
H	0.304795	-3.439947	0.588562
H	-1.085814	-3.670816	1.660005
C	0.104985	-2.453471	-2.191533
H	0.924825	-2.766863	-1.539738
H	-0.190887	-3.316363	-2.798242
H	0.476698	-1.675491	-2.861486

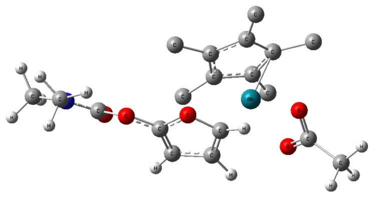

I-R$_{C5}$
E = -1281.35732280 Eh

C	2.088953	-1.690996	0.713171
C	1.372746	-2.225183	1.747043
C	0.024901	-2.061367	1.358044
C	-0.009827	-1.421587	0.121571
O	1.31875	-1.219878	-0.257983
H	-0.848932	-2.389786	1.906712
H	1.775453	-2.677149	2.640488
O	3.416434	-1.66554	0.508194
C	4.030894	-0.423215	0.606898
O	3.419165	0.558436	0.986731
N	5.317061	-0.488943	0.241891

C	6.000166	-1.72419	-0.116908
H	6.751863	-1.492705	-0.874923
H	6.501337	-2.159566	0.754549
H	5.300468	-2.447934	-0.529456
C	6.151531	0.692055	0.400555
H	5.528172	1.548307	0.650747
H	6.884912	0.532712	1.198291
H	6.683514	0.890764	-0.534032
H	-0.816867	-1.883277	-0.774477
Rh	-1.328444	0.218754	0.144934
C	-2.46117	1.960107	-0.554903
C	-1.415451	1.554713	-1.471827
C	-1.843319	2.273522	0.68051
C	-0.132307	1.736365	-0.812746
C	-0.39661	2.126558	0.526952
O	-2.94841	-1.137573	-0.07837
C	-2.873311	-2.093599	-0.91456
O	-1.791086	-2.482037	-1.440518
C	-4.151626	-2.768981	-1.317013
H	-4.854959	-2.780861	-0.48288
H	-4.599466	-2.191181	-2.132526
H	-3.957035	-3.781112	-1.672006
C	0.615412	2.413112	1.583229
H	0.8912	3.473073	1.542568
H	0.213973	2.209677	2.578484
H	1.517052	1.814535	1.437628
C	1.200565	1.590396	-1.461714
H	1.982449	1.384061	-0.730096
H	1.196144	0.791311	-2.204896
H	1.436115	2.529787	-1.974985
C	-1.614989	1.133738	-2.886316
H	-2.588039	0.657593	-3.022176
H	-1.572017	2.016289	-3.534753
H	-0.835038	0.439017	-3.204666
C	-3.917551	1.959045	-0.875362
H	-4.528301	1.998913	0.027469
H	-4.155182	2.830534	-1.494196
H	-4.191399	1.06245	-1.436455
C	-2.511521	2.684811	1.947109
H	-2.35921	3.758507	2.103057
H	-3.583885	2.487623	1.921376
H	-2.078929	2.161473	2.80385

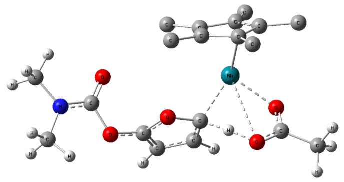

I-TS$_{C5}$
E = -1281.32207771 Eh

C	1.845001	-1.344081	0.647511
C	1.129499	-1.878216	1.680121
C	-0.219836	-1.713496	1.290775
C	-0.253754	-1.071228	0.056837
O	1.074156	-0.872017	-0.324144
H	-1.093456	-2.040168	1.840936
H	1.531818	-2.330886	2.573435
O	3.172923	-1.318142	0.441501
C	3.78646	-0.076167	0.540399
O	3.175179	0.90593	0.920201
N	5.073046	-0.141483	0.17533
C	5.756085	-1.376729	-0.183421
H	6.507892	-1.145348	-0.941418
H	6.257297	-1.812271	0.687987
H	5.056358	-2.100445	-0.595909
C	5.907439	1.039415	0.334025
H	5.284013	1.895633	0.584231
H	6.640909	0.880262	1.131779
H	6.439532	1.238298	-0.600512
H	-1.113193	-1.567337	-0.882367
Rh	-1.572281	0.565967	0.078352
C	-2.705225	2.307849	-0.621118
C	-1.65939	1.901955	-1.538283
C	-2.087716	2.621081	0.613884
C	-0.376434	2.083658	-0.879467
C	-0.640862	2.473856	0.460368
O	-3.192529	-0.791316	-0.14558
C	-3.12022	-1.744403	-0.979057
O	-2.032107	-2.133925	-1.505857
C	-4.394992	-2.421759	-1.384004
H	-5.097472	-2.43291	-0.54931
H	-4.842861	-1.84367	-2.199289
H	-4.20056	-3.433916	-1.738817
C	0.371285	2.760426	1.516669

H	0.646916	3.820447	1.47584
H	-0.030156	2.5571	2.511945
H	1.272956	2.161843	1.371077
C	0.95652	1.937852	-1.528272
H	1.738279	1.731192	-0.796592
H	0.952168	1.138693	-2.271339
H	1.192241	2.877231	-2.041505
C	-1.859076	1.481199	-2.952907
H	-2.832156	1.00503	-3.088772
H	-1.816269	2.363604	-3.601529
H	-1.079145	0.786464	-3.271295
C	-4.161638	2.306466	-0.941867
H	-4.772497	2.346341	-0.039085
H	-4.399435	3.177854	-1.560773
H	-4.435627	1.409977	-1.503145
C	-2.755628	3.03237	1.88067
H	-2.603669	4.106036	2.037005
H	-3.828008	2.835033	1.855
H	-2.323203	2.508836	2.737376

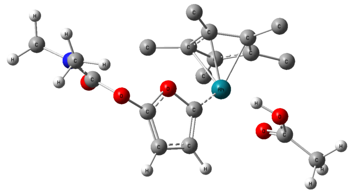

I-P$_{C5}$
E = -1281.33533897 Eh

C	1.9847	1.25389	-0.312458
C	1.393585	2.202421	-1.081282
C	0.003562	1.86878	-1.054257
C	-0.151412	0.733658	-0.287371
O	1.099197	0.382001	0.185817
H	-0.794885	2.397883	-1.557614
H	1.884654	3.017103	-1.591664
O	3.265521	1.107079	0.085316
C	3.90824	-0.041326	-0.35352
O	3.399474	-0.793539	-1.161855
N	5.109726	-0.173867	0.234891
C	5.693707	0.820661	1.122448

H	6.310826	0.303966	1.861598
H	6.327073	1.521369	0.566365
H	4.917236	1.375929	1.644398
C	5.986656	-1.247891	-0.200615
H	5.434534	-1.927992	-0.84649
H	6.842882	-0.84277	-0.751503
H	6.356791	-1.796459	0.670544
H	-1.700906	1.609659	1.271849
Rh	-1.6733	-0.556426	-0.215189
C	-3.124696	-2.144656	0.398602
C	-2.271303	-1.679736	1.474986
C	-2.288385	-2.647396	-0.615989
C	-0.894873	-2.005204	1.148919
C	-0.893096	-2.534968	-0.173476
O	-3.11802	1.049871	-0.456822
C	-3.286607	1.980752	0.339517
O	-2.47222	2.211603	1.343862
C	-4.421854	2.936098	0.223338
H	-4.029168	3.899736	-0.116239
H	-5.151969	2.565392	-0.494147
H	-4.883991	3.089206	1.201015
C	-2.689026	-3.17181	-1.952097
H	-2.499751	-4.250054	-1.990768
H	-3.746139	-2.997076	-2.156045
H	-2.096698	-2.706226	-2.745215
C	0.297353	-3.016041	-0.934626
H	1.180954	-2.414627	-0.709156
H	0.507764	-4.057897	-0.668246
H	0.118059	-2.972756	-2.010966
C	0.273374	-1.872344	2.065379
H	1.209158	-1.812172	1.510291
H	0.187829	-0.987506	2.699037
H	0.307486	-2.754154	2.714961
C	-2.748892	-1.102217	2.762832
H	-3.653815	-0.507994	2.618729
H	-2.98861	-1.918955	3.453416
H	-1.985073	-0.477282	3.229124
C	-4.613574	-2.019533	0.386125
H	-5.019701	-2.178352	-0.614082
H	-5.056499	-2.760185	1.059766
H	-4.92537	-1.028536	0.727213

Neutral dichloride manifold

Preliminary exploration in this manifold was performed with the simplified Cp model

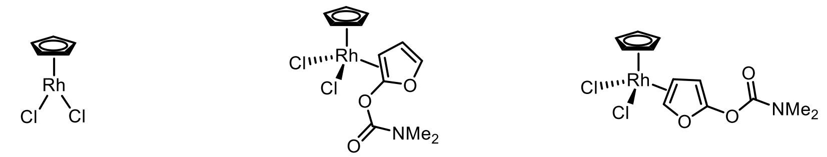

Rh(Cp*)Cl_2 **II** — [Rh(Cp*)(**1a**)(OAc)]$^+$ **II-R$_{C3}$** — [Rh(Cp*)(**1a**)(OAc)]$^+$ **II-R$_{C5}$**

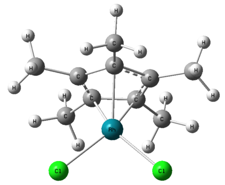

II

E = -1421.06327820 Eh

Rh	-1.0616	-0.4706	0.0240
C	-1.6791	-2.4665	0.4664
C	-0.6391	-2.1109	1.3739
C	-1.1482	-2.4008	-0.8919
C	0.5174	-1.7668	0.5821
C	0.2144	-1.9976	-0.8157
Cl	-2.0284	1.0176	1.5431
Cl	-0.9989	1.0915	-1.7127
C	-0.7239	-2.0829	2.8634
H	-0.4172	-3.0527	3.2704
H	-0.0732	-1.3136	3.2829
H	-1.7407	-1.8732	3.1986
C	1.8292	-1.3051	1.1173
H	2.4551	-2.1746	1.3489
H	2.3586	-0.6878	0.3893
H	1.7006	-0.7226	2.0314
C	1.1656	-1.8240	-1.9518
H	1.7768	-2.7261	-2.0646
H	0.6365	-1.6457	-2.8885
H	1.8323	-0.9763	-1.7820
C	-1.9193	-2.7265	-2.1262
H	-1.9433	-3.8122	-2.2716
H	-2.9492	-2.3708	-2.0515

H	-1.4701	-2.2678	-3.0081
C	-3.0689	-2.8749	0.8192
H	-3.7882	-2.4995	0.0883
H	-3.1373	-3.9686	0.8316
H	-3.3571	-2.4989	1.8017

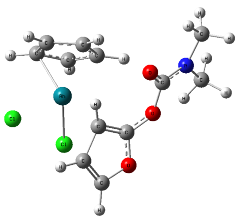

II-R$_{C3}$
E = -1776.90555745 Eh

C	-2.408592	-1.267479	0.34358
C	-2.556232	0.055794	0.173028
C	-1.282415	0.56506	-0.296646
C	-0.458844	-0.548957	-0.277696
O	-1.110341	-1.643745	0.072229
H	-3.075687	-2.072547	0.60211
H	-0.941951	1.587018	-0.259145
O	0.84099	-0.797315	-0.494688
C	1.765798	0.224691	-0.429416
O	1.487878	1.346071	-0.057338
N	2.968266	-0.21016	-0.856684
H	-3.462569	0.627075	0.288207
Rh	-1.435825	0.697342	-2.784188
C	-1.100977	1.526359	-4.705951
C	-0.21123	0.415478	-4.537895
C	-0.739487	2.525511	-3.732353
H	-1.892592	1.604845	-5.437386
C	0.642711	0.689923	-3.43912
H	-0.249747	-0.5095	-5.095837
C	0.314039	2.007019	-2.947419
H	-1.240304	3.471358	-3.583322
H	1.414707	0.037716	-3.05682
H	0.769681	2.487345	-2.092317
Cl	-2.305252	-1.499811	-2.98703
C	4.107781	0.684489	-0.743475
H	4.630281	0.739657	-1.703226

H	3.763531	1.677577	-0.46192
H	4.805684	0.317355	0.017099
C	3.265095	-1.592406	-1.206506
H	2.372088	-2.109269	-1.551323
H	4.004449	-1.593653	-2.011904
H	3.680783	-2.133331	-0.348885
Cl	-3.615396	1.638354	-2.455663

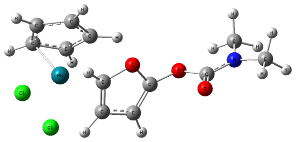

II-R$_{C5}$
E = -1776.90827476 Eh

C	0.600526	0.909221	-0.142739
C	0.457126	-0.41454	-0.347989
C	-0.931074	-0.596404	-0.691882
C	-1.49764	0.663717	-0.666435
O	-0.559257	1.589442	-0.33113
H	-1.471499	-1.529795	-0.733191
H	1.220525	-1.172306	-0.276335
Rh	-1.367225	-0.072584	-3.041565
C	-2.023346	1.413213	-4.499382
C	-1.187351	0.427379	-5.110953
C	-1.279664	2.062656	-3.481181
H	-3.073858	1.561607	-4.706825
C	0.117881	0.527243	-4.504442
H	-1.475403	-0.257994	-5.895047
C	0.056913	1.513326	-3.496478
H	-1.641628	2.839636	-2.823178
H	0.968164	-0.099486	-4.732117
H	0.862578	1.787738	-2.829753
Cl	-0.637273	-2.358213	-3.244955
H	-2.523606	0.996571	-0.65819
O	1.688716	1.663029	0.095777
C	1.843049	2.091487	1.41431
O	1.050327	1.790044	2.276409
N	2.960596	2.831521	1.547875
C	3.829175	3.215859	0.449144
H	3.727477	2.525711	-0.385109

H	3.602185	4.23205	0.10463
H	4.866433	3.190867	0.794971
C	3.236967	3.441192	2.836284
H	4.247052	3.176261	3.16465
H	3.164301	4.53258	2.765774
H	2.515976	3.081366	3.567814
Cl	-3.60523	-0.784822	-2.632764

Attempt at finding σ-bond metathesis transition states:

Relaxed scan of H-Cl bond from **II-R$_{C3}$**

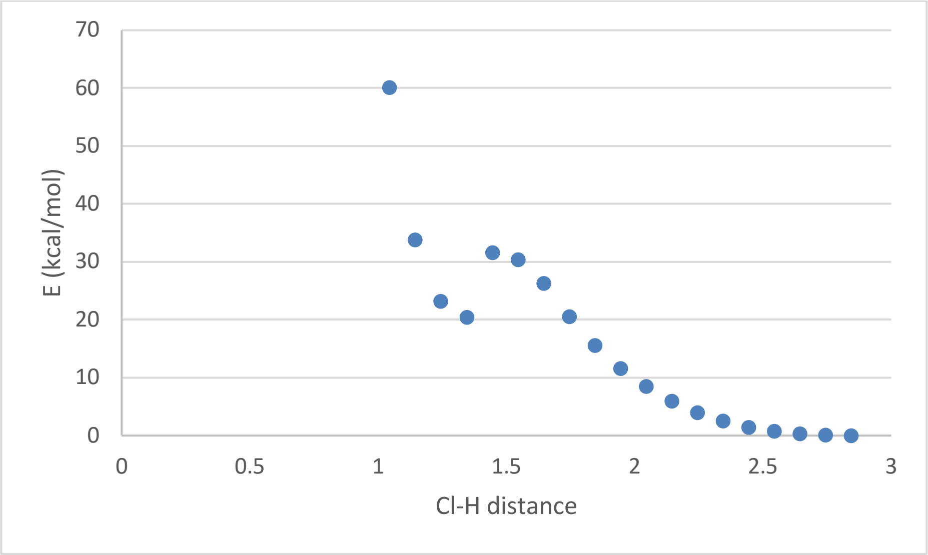

Relaxed scan of H-Cl bond from **II-R$_{C5}$**

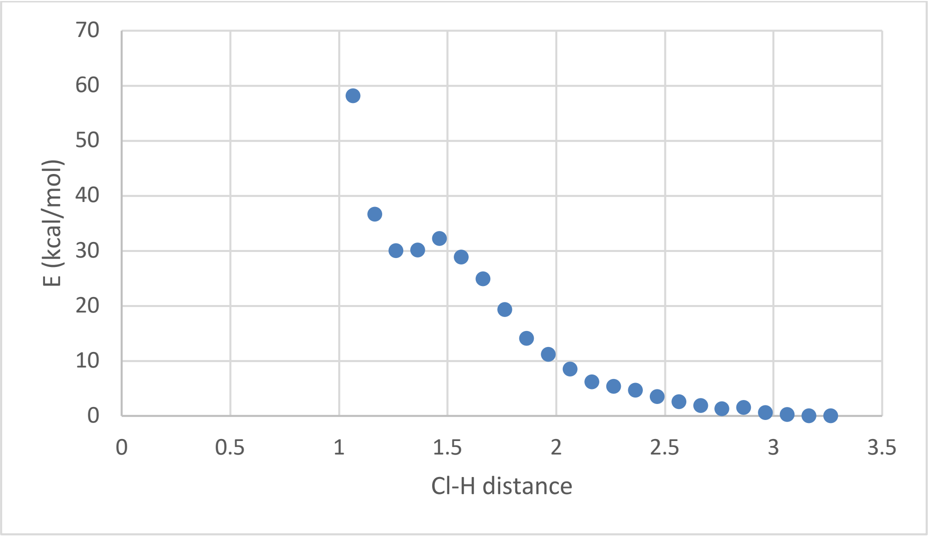

Neutral chloride-acetate-manifold

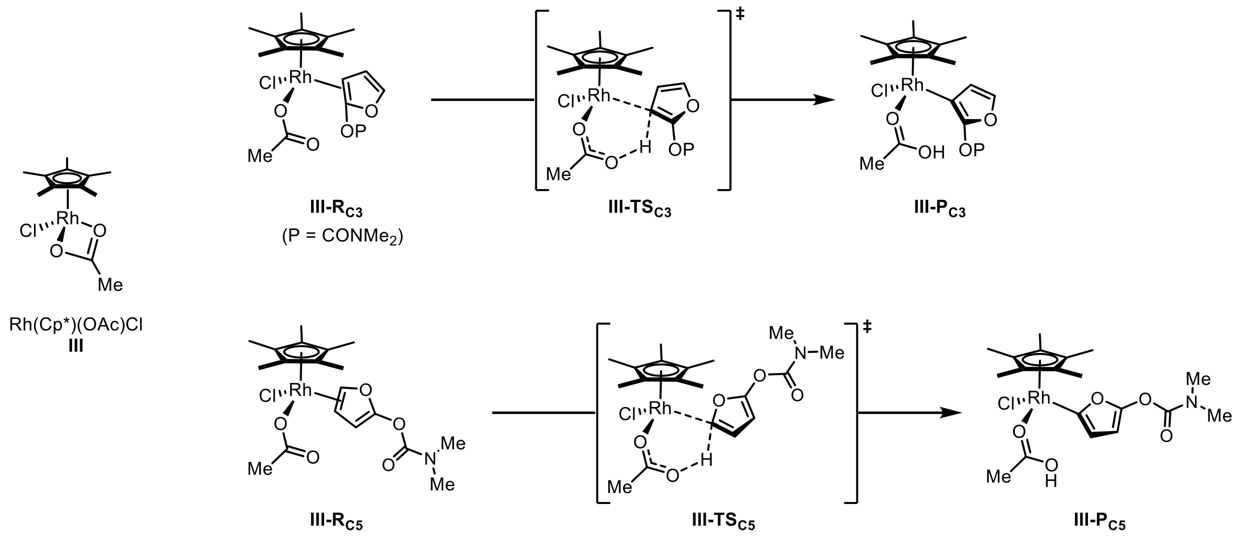

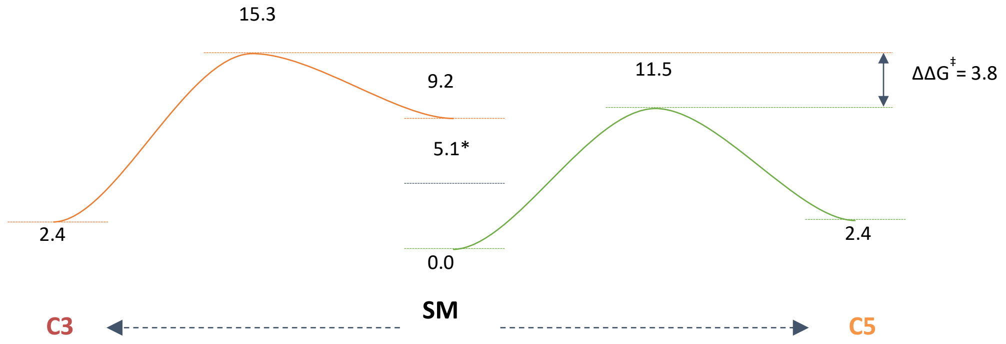

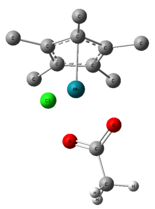

III
E = -1189.28810719 Eh

Rh	0.1726	-0.1846	-0.0567
C	-1.1963	0.9685	1.1283
C	-1.8269	-0.2492	0.7052
C	-0.8171	1.7195	-0.0395
C	-1.8603	-0.2492	-0.7409
C	-1.2411	0.9614	-1.1922
Cl	0.5964	-2.5409	-0.1445
O	2.0240	0.0601	1.0488
C	2.6769	0.1274	-0.0369
O	2.0322	0.1491	-1.1288
C	4.1746	0.1293	-0.0310
H	4.5155	-0.9100	-0.0759
H	4.5621	0.6566	-0.9042
H	4.5558	0.5775	0.8878
C	-0.1623	3.0627	-0.0580
H	-0.9127	3.8560	0.0313
H	0.5451	3.1697	0.7671
H	0.3866	3.2191	-0.9888
C	-0.9457	1.3654	2.5449
H	-1.8541	1.8062	2.9700
H	-0.6687	0.5013	3.1524
H	-0.1423	2.0999	2.6177
C	-2.4060	-1.3066	1.5859
H	-3.4927	-1.1839	1.6534
H	-2.1924	-2.3014	1.1898
H	-1.9922	-1.2527	2.5945
C	-2.4746	-1.3096	-1.5928
H	-3.5355	-1.0888	-1.7553
H	-1.9831	-1.3739	-2.5652
H	-2.3921	-2.2874	-1.1156
C	-1.0427	1.3696	-2.6136
H	-1.8552	2.0368	-2.9221
H	-0.0973	1.8996	-2.7448
H	-1.0388	0.5045	-3.2787

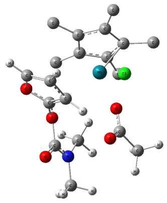

III-R$_{C3}$
E = -1741.69352597 Eh

C	-0.744742	3.339641	-1.111046
C	-0.9216	2.256416	-1.893715
C	-0.036304	1.230363	-1.395251
C	0.622199	1.831467	-0.350729
O	0.203845	3.086714	-0.146779
H	-1.18698	4.322301	-1.076744
H	0.300401	0.317171	-1.883354
O	1.584837	1.40956	0.478053
C	2.796709	1.036804	-0.115696
O	3.168589	1.511086	-1.160911
N	3.434123	0.153496	0.675833
H	-1.598298	2.160088	-2.729279
Rh	-1.294597	-0.33164	0.092356
C	-1.552376	-0.821402	2.198987
C	-2.626436	-1.334421	1.382674
C	-1.526678	0.59507	2.061999
C	-3.338716	-0.198691	0.826943
C	-2.651632	0.978086	1.220736
Cl	-2.051058	-1.262496	-1.996711
O	0.387828	-1.564066	0.132106
C	1.264902	-1.862996	-0.781054
O	1.403452	-1.32444	-1.873457
C	4.773856	-0.266058	0.310543
H	4.822407	-1.358421	0.26354
H	5.018516	0.141036	-0.668925
H	5.506078	0.09151	1.043527
C	2.87909	-0.321625	1.934178
H	3.442432	-1.206276	2.238655
H	2.96668	0.430427	2.727237
H	1.834596	-0.606294	1.799871
C	2.162529	-3.020612	-0.371368
H	2.431473	-2.960173	0.686315
H	1.614254	-3.957079	-0.519556
H	3.060016	-3.041567	-0.991446

C	-3.089335	2.376307	0.94119
H	-2.26558	3.08366	1.042022
H	-3.502092	2.475607	-0.064155
H	-3.865751	2.660534	1.660806
C	-0.610485	1.530897	2.780602
H	-1.023064	1.769756	3.767451
H	0.378318	1.089983	2.917002
H	-0.481685	2.466544	2.234508
C	-0.600308	-1.6684	2.972879
H	-1.150245	-2.382336	3.593034
H	0.038146	-2.22452	2.277078
H	0.033769	-1.063138	3.623664
C	-3.01407	-2.769499	1.255907
H	-3.742594	-3.034797	2.030781
H	-3.457523	-2.966671	0.278389
H	-2.141303	-3.416688	1.364768
C	-4.594875	-0.268179	0.02704
H	-4.607552	-1.155693	-0.606971
H	-5.455348	-0.306435	0.704963
H	-4.703826	0.604164	-0.619759

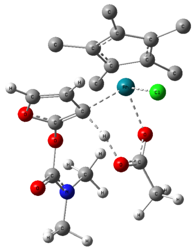

III-TS$_{C3}$
E = -1741.67891918 Eh

C	-0.3665	3.2017	-1.4365
C	-0.9752	2.0199	-1.6744
C	-0.2704	1.0030	-0.9157
C	0.7302	1.7245	-0.3114
O	0.7032	3.0270	-0.5880
H	-0.5271	4.2146	-1.7705
H	0.2565	-0.0806	-1.4100
O	1.7122	1.2786	0.4963
C	2.9276	0.9972	-0.1341
O	3.2739	1.5659	-1.1414

N	3.6028	0.0552	0.5554
H	-1.8334	1.8501	-2.3086
Rh	-1.4071	-0.4847	0.2221
C	-2.1334	-1.2787	2.1214
C	-3.2479	-0.9388	1.3085
C	-1.3537	-0.0766	2.3474
C	-3.1607	0.4780	0.9973
C	-2.0261	1.0068	1.6980
Cl	-2.3143	-1.3109	-1.8659
O	0.1815	-1.9035	0.1251
C	1.0212	-1.9122	-0.8234
O	1.0562	-1.0601	-1.7521
C	4.9704	-0.2416	0.1718
H	5.0767	-1.3074	-0.0559
H	5.2264	0.3371	-0.7140
H	5.6606	0.0156	0.9833
C	3.0673	-0.6177	1.7274
H	3.6002	-1.5638	1.8482
H	3.2050	-0.0243	2.6392
H	2.0080	-0.8348	1.5932
C	2.0439	-3.0185	-0.8435
H	2.1070	-3.5124	0.1267
H	1.7429	-3.7547	-1.5953
H	3.0164	-2.6188	-1.1386
C	-0.1541	0.0278	3.2323
H	0.3585	-0.9336	3.3088
H	0.5583	0.7623	2.8511
H	-0.4526	0.3301	4.2428
C	-1.7775	-2.6263	2.6572
H	-1.9190	-2.6501	3.7431
H	-2.3931	-3.4097	2.2127
H	-0.7315	-2.8594	2.4406
C	-1.6490	2.4487	1.7785
H	-0.6118	2.5791	2.0915
H	-1.7884	2.9561	0.8215
H	-2.2889	2.9428	2.5182
C	-4.1616	1.2605	0.2112
H	-4.5007	0.6918	-0.6575
H	-5.0338	1.5010	0.8297
H	-3.7299	2.1969	-0.1490
C	-4.3406	-1.8467	0.8538
H	-5.1840	-1.7835	1.5510
H	-4.6886	-1.5671	-0.1417
H	-4.0067	-2.8845	0.8060

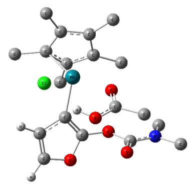

III-P$_{C3}$
E = -1741.70520009 Eh

C	-0.13356	-2.340109	2.747987
C	0.875375	-1.928665	1.93987
C	0.384718	-0.813232	1.164315
C	-0.890861	-0.676243	1.601347
O	-1.23828	-1.573381	2.55523
H	-0.230378	-3.130368	3.476722
H	0.735829	-2.079785	-1.505488
O	-1.852168	0.251402	1.319488
C	-2.777292	-0.105656	0.37283
O	-2.773595	-1.185686	-0.182771
N	-3.669992	0.88973	0.154063
H	1.861668	-2.365339	1.872461
Rh	1.386174	0.365846	-0.144663
C	1.919386	2.421593	-0.915841
C	3.094378	1.812774	-0.461529
C	0.959215	2.4399	0.189708
C	2.883808	1.405732	0.931075
C	1.603469	1.909032	1.350334
Cl	2.647202	-1.5863	-0.924058
O	-0.219577	0.060451	-1.615165
C	-0.662501	-0.970586	-2.133558
O	-0.118091	-2.14941	-2.003663
C	-4.695949	0.701914	-0.851499
H	-4.647358	1.505026	-1.596134
H	-4.546732	-0.257217	-1.343796
H	-5.69131	0.715426	-0.392046
C	-3.734841	2.125008	0.909994
H	-3.48096	2.984144	0.277599
H	-4.753275	2.266523	1.289096
H	-3.053746	2.092056	1.756477
C	-1.850887	-0.939049	-3.041165
H	-2.373108	0.011005	-2.938219
H	-1.504957	-1.057345	-4.073197
H	-2.519608	-1.766445	-2.803591
C	1.057305	1.877256	2.740342

H	1.385227	2.771399	3.282631
H	-0.033797	1.853657	2.740434
H	1.40591	0.997426	3.28415
C	3.925041	0.782928	1.803718
H	4.526612	0.068496	1.237312
H	4.594927	1.548928	2.211697
H	3.468846	0.246634	2.638513
C	4.353066	1.555587	-1.222058
H	4.23733	1.785783	-2.28277
H	5.164895	2.173799	-0.823046
H	4.650028	0.506882	-1.13657
C	1.616653	2.946714	-2.281761
H	0.62498	2.62087	-2.60764
H	1.627193	4.042473	-2.283
H	2.343062	2.596972	-3.017716
C	-0.395751	3.064839	0.111012
H	-0.965396	2.64739	-0.724095
H	-0.961754	2.887817	1.025187
H	-0.310255	4.147122	-0.039439

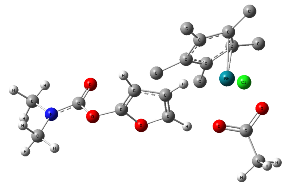

III-R$_{C5}$
E = -1741.70872364 Eh

C	2.723908	0.585586	1.56868
C	2.082293	-0.147187	2.509134
C	0.695093	0.187828	2.356182
C	0.624782	1.085952	1.342913
O	1.872883	1.346461	0.85699
H	-0.148946	-0.194799	2.910258
H	2.538124	-0.826963	3.212569
Rh	-2.273227	-0.498217	-0.560868
C	-2.700651	-2.044525	-1.972132
C	-2.555153	-2.603731	-0.654385
C	-1.468324	-1.400905	-2.329147
C	-1.203605	-2.34183	-0.211098
C	-0.540475	-1.600083	-1.23784

Cl	-2.938898	-0.089986	1.710297
O	-3.746688	0.968176	-1.184698
C	-2.913817	1.910887	-1.030111
O	-1.712913	1.615443	-0.748365
H	-0.192598	1.595077	0.855607
O	4.03984	0.759182	1.303119
C	4.566273	0.023897	0.261011
O	3.93047	-0.821609	-0.332816
N	5.847176	0.37956	0.02178
C	6.585383	1.358886	0.79872
H	7.248371	0.868664	1.52213
H	7.197905	1.960247	0.120035
H	5.90633	2.019753	1.331875
C	6.604802	-0.36395	-0.965841
H	7.034881	0.322282	-1.70307
H	7.420691	-0.918007	-0.486552
H	5.944962	-1.06638	-1.471606
C	-3.349322	3.340684	-1.128144
H	-2.52026	3.974973	-1.445638
H	-4.192638	3.440508	-1.813252
H	-3.672648	3.665475	-0.133876
C	0.866064	-1.105579	-1.22565
H	0.915205	-0.046681	-1.489055
H	1.347811	-1.231722	-0.257221
H	1.452365	-1.660969	-1.965341
C	-1.157306	-0.708754	-3.615744
H	-0.776398	-1.425787	-4.351571
H	-2.045053	-0.230467	-4.035329
H	-0.397189	0.06139	-3.469217
C	-3.940109	-2.092372	-2.800881
H	-3.976518	-3.039462	-3.350495
H	-4.834529	-2.024724	-2.17834
H	-3.969254	-1.275091	-3.523094
C	-3.574515	-3.39464	0.096578
H	-3.455175	-4.462422	-0.119503
H	-3.465559	-3.244851	1.172241
H	-4.588975	-3.100345	-0.179804
C	-0.624792	-2.817716	1.079221
H	-0.455803	-3.89909	1.026947
H	0.324778	-2.32793	1.2959
H	-1.308993	-2.614072	1.90618

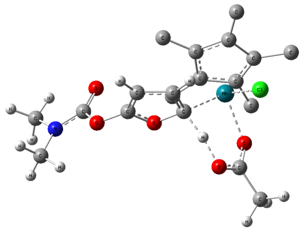

III-TS$_{C5}$
E = -1741.68466456 Eh

C	2.2232	0.7759	0.9555
C	1.8457	0.6323	2.2599
C	0.4356	0.5633	2.2089
C	0.0232	0.6547	0.8890
O	1.1988	0.7893	0.1200
H	-0.2496	0.4687	3.0403
H	2.5014	0.6055	3.1166
Rh	-1.5441	-0.6383	0.1070
C	-1.8765	-2.0787	-1.4959
C	-2.5834	-2.4986	-0.3297
C	-0.4728	-1.9902	-1.1806
C	-1.6029	-2.7109	0.7194
C	-0.3099	-2.4260	0.1888
Cl	-2.9903	0.1258	1.9047
O	-2.3464	0.9047	-1.1148
C	-2.2152	2.1384	-0.8623
O	-1.4435	2.6117	0.0151
H	-0.7725	1.5911	0.5235
O	3.4435	1.0518	0.4474
C	4.0311	0.1074	-0.3680
O	3.5860	-1.0175	-0.4884
N	5.1297	0.6038	-0.9662
C	5.6426	1.9460	-0.7482
H	6.4487	1.9437	-0.0052
H	6.0411	2.3256	-1.6931
H	4.8511	2.6123	-0.4127
C	5.9580	-0.2861	-1.7590
H	6.0972	0.1298	-2.7619
H	6.9408	-0.4097	-1.2900
H	5.4739	-1.2575	-1.8374
C	-3.0670	3.0963	-1.6550
H	-2.5804	4.0692	-1.7354
H	-3.2855	2.6919	-2.6444

H	-4.0136	3.2264	-1.1203
C	-4.0517	-2.7364	-0.1940
H	-4.2732	-3.8073	-0.2667
H	-4.4123	-2.3735	0.7712
H	-4.6130	-2.2175	-0.9734
C	-1.9080	-3.2238	2.0874
H	-1.9661	-4.3182	2.0680
H	-1.1353	-2.9345	2.8023
H	-2.8599	-2.8301	2.4479
C	0.9887	-2.6147	0.8992
H	1.3393	-3.6408	0.7376
H	1.7604	-1.9388	0.5278
H	0.8804	-2.4593	1.9740
C	0.6232	-1.6661	-2.1437
H	0.8716	-2.5495	-2.7438
H	0.3214	-0.8681	-2.8265
H	1.5260	-1.3435	-1.6224
C	-2.4757	-1.7367	-2.8185
H	-1.9256	-0.9260	-3.2995
H	-2.4397	-2.6130	-3.4753
H	-3.5157	-1.4230	-2.7183

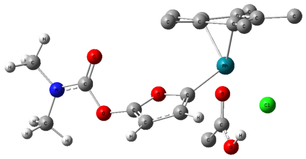

III-P$_{C5}$
E = -1741.70488097 Eh

C	2.028448	0.54595	1.012849
C	1.678656	0.543359	2.316491
C	0.264452	0.278384	2.315416
C	-0.13345	0.137127	1.017357
O	0.971999	0.326809	0.208297
H	-0.382837	0.190222	3.17615
H	2.333713	0.705539	3.159343
Rh	-1.77284	-0.530152	0.064245
C	-2.493085	-1.882199	-1.588885
C	-3.196334	-2.220719	-0.42617
C	-1.057801	-2.001039	-1.314188

C	-2.210126	-2.53829	0.611139
C	-0.905494	-2.512793	0.013876
Cl	-3.226909	0.642088	1.655579
O	-1.626709	1.244669	-1.225877
C	-1.475116	2.424573	-0.894895
O	-1.71645	2.89009	0.30126
H	-2.107431	2.178048	0.873663
O	3.208151	0.824722	0.397899
C	3.801939	-0.21492	-0.276818
O	3.356475	-1.345847	-0.279277
N	4.915069	0.200309	-0.923454
C	5.46849	1.537685	-0.813049
H	6.281627	1.570501	-0.077159
H	5.870859	1.835126	-1.786179
H	4.699537	2.249333	-0.522162
C	5.739332	-0.786259	-1.593657
H	5.955572	-0.458523	-2.615818
H	6.689182	-0.923346	-1.062504
H	5.208968	-1.736139	-1.62515
C	-0.958031	3.443446	-1.860146
H	0.104702	3.600931	-1.648677
H	-1.06453	3.07955	-2.881332
H	-1.473917	4.396358	-1.732022
C	0.37483	-2.934529	0.657754
H	1.225582	-2.348256	0.302951
H	0.324874	-2.829893	1.742905
H	0.562654	-3.989016	0.423955
C	-2.546011	-3.005841	1.989585
H	-3.443116	-2.506108	2.360869
H	-2.726982	-4.087046	1.991384
H	-1.732405	-2.792186	2.685721
C	-4.673483	-2.230928	-0.20228
H	-5.213896	-1.830179	-1.06198
H	-5.024027	-3.253641	-0.025041
H	-4.936069	-1.626212	0.670624
C	-3.044132	-1.4161	-2.896805
H	-2.534884	-0.505932	-3.225838
H	-2.892149	-2.181216	-3.666038
H	-4.111986	-1.200131	-2.831808
C	0.036723	-1.799049	-2.311719
H	0.982507	-1.578619	-1.811695
H	0.169329	-2.699009	-2.923714
H	-0.194705	-0.966217	-2.980277

Other diastereoisomer III-TS$_{C3}$ (c3ab-cmdb-tscp)

E = -1741.67263914 Eh

C	-1.111342	-1.727931	0.640318
C	0.197585	-1.356455	0.853795
C	0.720619	-2.506566	1.576109
H	0.200582	-0.104831	1.359471
Rh	1.337223	-0.227175	-0.635256
C	2.988914	1.058814	-0.102486
C	2.938478	0.941299	-1.546863
C	3.223973	-0.233509	0.451198
C	3.13662	-0.431411	-1.868957
C	3.257668	-1.182477	-0.640786
Cl	0.000996	-1.174595	-2.415856
O	-0.095621	1.387162	-0.432186
C	-0.392624	1.731736	0.742991
O	-0.061872	1.067868	1.777652
O	-2.096867	-1.074401	0.01083
C	-2.895199	-0.239316	0.772192
O	-3.031996	-0.378216	1.967768
N	-3.465216	0.699707	-0.01299
C	-4.495624	1.546271	0.552918
H	-4.559909	1.368177	1.625235
H	-4.254858	2.600085	0.375174
H	-5.467258	1.330042	0.092728
C	-3.231501	0.810747	-1.444409
H	-2.278096	0.358783	-1.714709
H	-4.038318	0.33056	-2.01142
H	-3.20463	1.871973	-1.710071
O	-1.417428	-2.934027	1.114477
C	-0.271094	-3.412755	1.693063
H	-0.347321	-4.394202	2.133578
H	1.720778	-2.629313	1.962862
C	-1.169595	3.002692	0.952223
H	-1.967937	2.831357	1.676997
H	-0.498765	3.760371	1.370577
H	-1.575867	3.367576	0.00867
C	3.22954	-1.012341	-3.238607
H	2.658434	-0.43003	-3.962457
H	4.281176	-1.020579	-3.548887
H	2.853088	-2.035513	-3.262427
C	2.753691	2.071346	-2.506197
H	2.089106	2.83158	-2.089566

H	3.714981	2.545631	-2.733765
H	2.311444	1.72356	-3.441782
C	2.888178	2.331859	0.671534
H	2.517402	2.151363	1.682193
H	3.875429	2.802225	0.743447
H	2.21625	3.039121	0.179781
C	3.453007	-0.505283	1.904924
H	3.681511	-1.556921	2.084459
H	4.307039	0.082122	2.257659
H	2.585106	-0.230418	2.510144
C	3.556545	-2.644973	-0.559426
H	4.610546	-2.833404	-0.793062
H	3.352552	-3.041127	0.436282
H	2.941999	-3.205727	-1.267003

Other diastereoisomer III-TS$_{C5}$ (c5ab-cmdb-tscp)

E = -1741.69385538 Eh

C	-1.912557	0.874405	0.23833
C	0.042972	1.516722	-0.476865
C	0.09666	1.494252	0.901077
H	0.634366	2.057248	-1.197843
Rh	1.641958	-0.295684	-0.350675
C	2.394148	-1.932812	0.885207
C	2.627205	-2.167615	-0.516337
C	0.988652	-1.814239	1.091494
C	1.333243	-2.296187	-1.15989
C	0.332508	-2.05161	-0.185415
Cl	2.196482	0.499402	-2.582871
O	3.194696	0.86593	0.409717
C	3.377747	2.143612	0.248027
O	2.533814	2.95739	-0.111877
H	0.916712	1.875298	1.488626
O	-3.183405	0.522853	-0.02629
C	-3.875452	-0.269073	0.867919
O	-3.372666	-0.743392	1.86788
N	-5.142513	-0.42158	0.443906
C	-5.641805	0.205776	-0.773688
H	-6.680643	-0.097867	-0.90677
H	-5.604116	1.296297	-0.707039

H	-5.071791	-0.113461	-1.649534
C	-6.058828	-1.221846	1.236887
H	-6.43645	-2.062717	0.646224
H	-5.533671	-1.606573	2.109231
H	-6.90534	-0.612137	1.568871
O	-1.222756	1.139019	-0.875351
C	-1.181718	1.061547	1.368761
H	-1.511184	0.910434	2.381649
C	4.802575	2.573741	0.55512
H	5.175172	2.084075	1.45854
H	5.444762	2.268196	-0.277423
H	4.858566	3.657696	0.665232
C	-1.137612	-2.167035	-0.425443
H	-1.711644	-1.855362	0.447418
H	-1.447068	-1.570036	-1.286802
H	-1.390863	-3.21231	-0.635193
C	0.315209	-1.657477	2.416221
H	-0.729032	-1.363642	2.305511
H	0.342333	-2.61193	2.954286
H	0.818833	-0.907882	3.031072
C	3.465838	-1.763396	1.907392
H	4.156081	-2.611598	1.874772
H	4.026082	-0.845448	1.697556
H	3.048946	-1.693664	2.913802
C	3.959045	-2.370888	-1.157302
H	4.211317	-3.437252	-1.181315
H	3.958065	-1.988439	-2.179812
H	4.737909	-1.842842	-0.603305
C	1.08867	-2.673812	-2.580675
H	1.90974	-2.356598	-3.223863
H	0.986715	-3.763442	-2.649877
H	0.171251	-2.216841	-2.956687

Neutral chloride-carbonate manifold

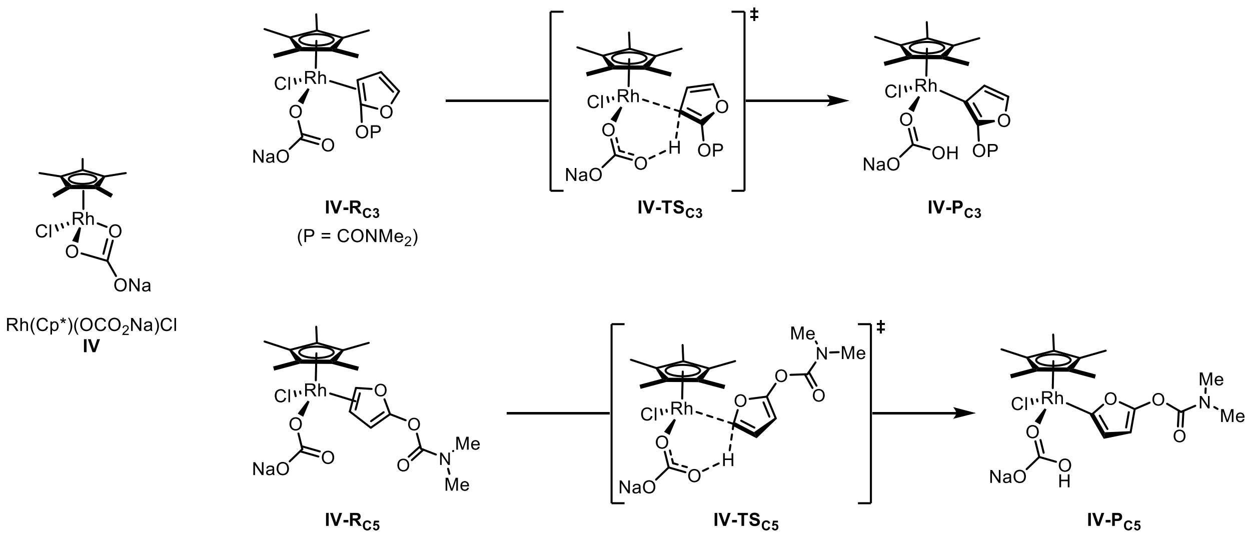

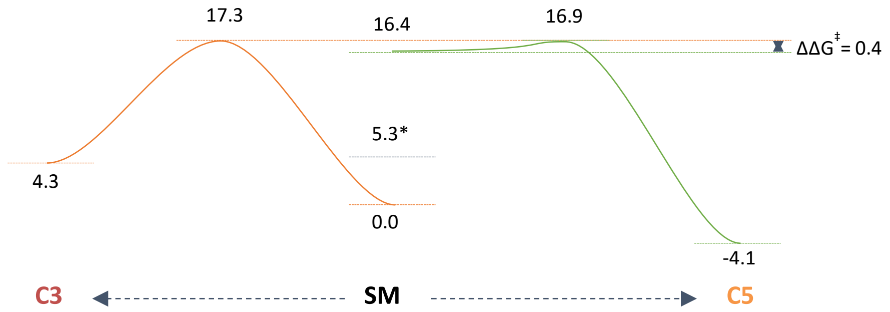

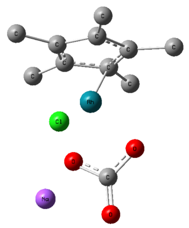

IV

E = -1386.94672175 Eh

Rh	1.476902	-0.586135	0.434161
C	1.16656	-1.584155	-1.453172
C	2.487984	-1.852602	-0.997608
C	1.018494	-0.150674	-1.586567
C	3.193602	-0.583649	-0.88785
C	2.296891	0.451555	-1.277121
Cl	2.689865	-1.340135	2.495453
O	-0.357909	-0.897956	1.39332
C	-0.20577	0.170013	2.11918
O	0.780172	0.946329	1.668223
O	-0.790615	0.426651	3.182375
Na	1.476499	0.683345	3.87132
C	2.584872	1.915969	-1.324458
H	3.433809	2.177619	-0.690356
H	2.819606	2.216611	-2.351584
H	1.722657	2.496506	-0.989522
C	4.624837	-0.435363	-0.486312
H	4.874539	-1.126022	0.321866
H	5.280682	-0.648033	-1.338024
H	4.837772	0.576116	-0.134914
C	3.08874	-3.191881	-0.720283
H	2.323813	-3.924691	-0.456519
H	3.623488	-3.555051	-1.605202
H	3.795667	-3.137908	0.109842
C	0.086389	-2.582848	-1.708919
H	-0.87885	-2.21529	-1.354295
H	0.005521	-2.776879	-2.78416
H	0.289697	-3.528498	-1.20385
C	-0.194419	0.565975	-2.083115
H	-0.268487	1.563647	-1.645106
H	-0.15513	0.672948	-3.173163
H	-1.105039	0.021104	-1.82648

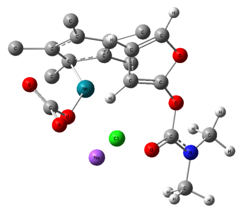

IV-R$_{C3}$
E= -1939.36808189 Eh

C	-2.251435	-0.36719	0.072976
C	-2.085377	-1.196376	-0.987488
C	-2.841234	-0.583234	-2.043098
H	-1.531373	-2.121227	-1.054025
Rh	1.831667	-0.759393	-0.685149
C	2.801416	-0.000446	-2.442849
C	3.452733	0.496066	-1.238992
C	1.453856	0.457378	-2.428403
C	2.515469	1.298129	-0.513503
C	1.270538	1.238817	-1.215393
Cl	1.832499	-1.341173	1.657746
O	1.197518	-2.589934	-0.933246
C	0.601652	-3.167459	-2.046027
O	1.014678	-2.879955	-3.17984
O	-0.329174	-3.958586	-1.717412
Na	-0.053558	-3.64817	0.554405
O	-1.776377	-0.275028	1.340336
C	-1.547168	-1.39223	2.081802
O	-1.687994	-2.526332	1.638205
N	-1.192213	-1.080106	3.338154
C	-0.731254	-2.150553	4.205744
H	-1.169203	-3.094611	3.886023
H	0.361949	-2.228028	4.177393
H	-1.050297	-1.942451	5.229982
C	-0.857392	0.278016	3.739694
H	-1.536287	0.993763	3.279645
H	-0.960384	0.350364	4.824395
H	0.17495	0.519758	3.46142
O	-3.029359	0.689579	-0.224488
C	-3.381395	0.550143	-1.535579
H	-4.020061	1.322222	-1.933597
H	-2.952676	-0.961384	-3.04858
C	0.014715	1.925886	-0.797637
H	-0.179111	1.782211	0.267843

H	0.110058	3.002018	-0.982484
H	-0.845569	1.560151	-1.359027
C	2.770897	2.041219	0.756808
H	3.500319	1.523739	1.382492
H	3.156435	3.042247	0.533695
H	1.853658	2.151214	1.33853
C	4.872228	0.236104	-0.861588
H	5.171036	-0.77778	-1.136977
H	5.529805	0.939296	-1.384973
H	5.025339	0.354189	0.212436
C	3.408851	-0.839084	-3.515001
H	2.751244	-1.693584	-3.709833
H	3.513977	-0.247026	-4.431223
H	4.396934	-1.204263	-3.228497
C	0.447055	0.189897	-3.494345
H	0.722943	0.734675	-4.404568
H	0.429879	-0.883241	-3.715679
H	-0.553017	0.505334	-3.193344

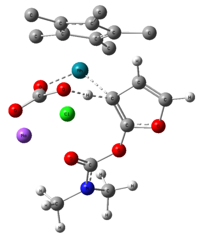

IV-TS$_{C3}$
E = -1742.76504944 Eh

C	0.761913	-1.805271	0.968955
C	0.367279	-1.054601	1.255884
C	1.246339	-2.061991	1.848443
H	0.440434	0.035371	1.819463
Rh	1.270697	0.158591	-0.419765
C	3.270282	0.963396	-0.693057
C	2.704581	0.66818	-1.962981
C	3.327406	-0.241794	0.066013
H	3.511622	1.950017	-0.322299
C	2.467531	-0.756286	-2.016308
H	2.49071	1.378166	-2.748885
C	2.854314	-1.313344	-0.782675

H	3.699777	-0.334911	1.075968
H	2.016032	-1.288769	-2.841473
H	2.759416	-2.351335	-0.49702
Cl	-0.715345	0.373366	-1.87044
O	0.598888	1.891	0.405709
C	0.321582	2.095233	1.711776
O	0.681933	1.209199	2.552872
O	-0.325203	3.138811	1.953407
Na	-1.510069	2.477777	0.010932
O	-1.955082	-1.60487	0.395567
C	-2.585732	-0.392407	0.407156
O	-2.230206	0.540299	1.106461
N	-3.677447	-0.417238	-0.38151
C	-4.388227	0.834855	-0.562309
H	-4.356487	1.415594	0.359898
H	-3.958018	1.418836	-1.389248
H	-5.432626	0.624355	-0.801053
C	-3.901984	-1.451249	-1.382032
H	-3.598351	-2.42415	-1.001673
H	-4.969644	-1.485881	-1.60872
H	-3.343066	-1.23424	-2.299465
O	-0.630402	-3.096943	1.269173
C	0.618846	-3.248619	1.823662
H	0.861782	-4.247532	2.14873
H	2.223641	-1.867481	2.266319

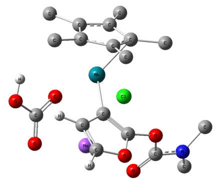

IV-P$_{C3}$

E = -1939.39024441Eh

C	-0.884316	-1.585502	0.921822
C	0.249046	-0.847612	1.049675
C	0.719287	-1.221199	2.36888
H	2.890932	1.740556	2.272129
Rh	1.227196	0.355704	-0.273642
C	3.259409	0.915195	-1.097213

C	2.424648	0.624459	-2.183718
C	3.282957	-0.247823	-0.209907
C	1.88684	-0.724423	-1.98658
C	2.507781	-1.285661	-0.825573
Cl	-0.83822	1.262131	-1.293448
O	1.017408	1.99551	1.144048
C	1.037573	1.905357	2.427591
O	2.257781	1.750917	2.99891
O	0.055364	1.970988	3.167429
Na	-1.387361	1.394155	1.415502
O	-1.775063	-1.714238	-0.100074
C	-2.832498	-0.850258	-0.07561
O	-3.062502	-0.11751	0.880351
N	-3.5822	-0.949829	-1.185805
C	-4.627734	0.037105	-1.390767
H	-4.976701	0.402493	-0.426716
H	-4.254006	0.883774	-1.979402
H	-5.462765	-0.427385	-1.92158
C	-3.105485	-1.65741	-2.364381
H	-2.65332	-2.607561	-2.085065
H	-3.960489	-1.857946	-3.013502
H	-2.371513	-1.0546	-2.911913
O	-1.153373	-2.365601	1.996443
C	-0.153208	-2.122949	2.881212
H	-0.210163	-2.656499	3.81742
H	1.590109	-0.830111	2.876957
C	4.175153	-0.418495	0.97889
H	5.081219	-0.967304	0.697179
H	4.498868	0.54443	1.3826
H	3.679261	-0.978297	1.775246
C	2.398988	-2.698959	-0.354918
H	3.180456	-3.30198	-0.831379
H	2.521246	-2.769687	0.727508
H	1.429215	-3.130799	-0.60939
C	0.9972	-1.437789	-2.952487
H	1.593084	-1.919737	-3.73652
H	0.405514	-2.204924	-2.448687
H	0.305571	-0.738158	-3.426259
C	2.076701	1.496691	-3.344828
H	2.463971	1.061471	-4.272596
H	0.991418	1.594879	-3.43909
H	2.495148	2.49883	-3.236816
C	3.980394	2.193254	-0.81377
H	3.752511	2.556245	0.192807

H	5.063649	2.046899	-0.883067
H	3.692755	2.980084	-1.512563

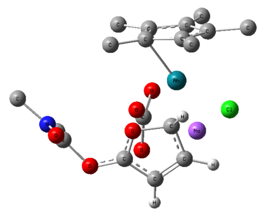

IV-R$_{C5}$
E= -1939.34046141 Eh

C	-2.33039	-0.706768	0.142133
C	-1.429546	-1.345352	0.916079
C	-0.26694	-1.510501	0.08299
C	-0.576274	-0.930408	-1.126361
O	-1.849346	-0.44258	-1.095703
H	0.605016	-2.116771	0.275188
H	-1.559224	-1.661978	1.938579
Rh	1.198301	0.515374	-0.077177
C	1.975623	2.220808	-1.157783
C	2.247549	2.339019	0.24478
C	0.554299	2.140853	-1.32485
C	0.973357	2.442029	0.943993
C	-0.059101	2.322421	-0.013025
Cl	2.03026	-0.544478	2.05405
O	2.178329	-0.826445	-1.286077
C	3.364834	-1.240672	-0.82703
O	4.268456	-0.374348	-0.563919
O	3.520869	-2.477613	-0.623502
Na	4.441964	-1.662497	1.291349
H	-0.094038	-0.969673	-2.089552
O	-3.549807	-0.20268	0.415947
C	-4.631365	-0.927227	-0.072595
O	-4.490133	-1.979466	-0.651932
N	-5.790819	-0.299234	0.214125
C	-5.878098	1.019022	0.817065
H	-4.993492	1.232432	1.412673
H	-5.991538	1.797442	0.052342
H	-6.753147	1.047636	1.472359
C	-7.026275	-0.859461	-0.301782
H	-7.75758	-0.955695	0.506946
H	-7.446611	-0.212675	-1.08103
H	-6.826762	-1.842537	-0.724133
C	-1.521274	2.44071	0.265554
H	-2.123047	2.024711	-0.54214
H	-1.792321	1.930091	1.192401
H	-1.785541	3.498789	0.37355
C	0.807084	2.682681	2.406678

H	1.554099	2.130653	2.979609
H	0.922487	3.751615	2.619631
H	-0.179791	2.368192	2.751922
C	3.611086	2.437405	0.839831
H	4.239177	1.650728	0.410284
H	4.048517	3.416999	0.614339
H	3.578838	2.312687	1.923458
C	3.02541	2.116267	-2.209851
H	3.539106	3.07933	-2.308744
H	3.748528	1.347764	-1.915108
H	2.600514	1.848685	-3.178193
C	-0.170628	2.062246	-2.628962
H	-1.156723	1.609435	-2.512301
H	-0.304888	3.067592	-3.043556
H	0.391201	1.470071	-3.354419

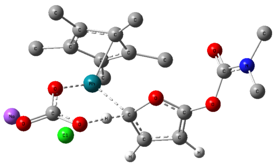

IV-TS$_{C5}$
E = -1939.33684578 Eh

C	2.59387	-0.853766	-1.088302
C	2.003028	-0.928689	-2.31946
C	0.642807	-1.173782	-2.037677
C	0.460585	-1.18917	-0.66106
O	1.735087	-0.982665	-0.093902
H	-0.15408	-1.3368	-2.749738
H	2.500767	-0.854154	-3.273762
Rh	-1.235497	0.159837	-0.064496
C	-1.567206	1.602034	1.529597
C	-2.401204	1.904852	0.406563
C	-0.19916	1.630808	1.094197
C	-1.535934	2.214037	-0.717025
C	-0.187354	2.053894	-0.298628
Cl	-2.74495	-0.760611	-1.848201
O	-2.071563	-1.305179	1.144862
C	-1.888031	-2.64506	1.062774
O	-0.742552	-3.088485	0.76069
O	-2.915077	-3.342234	1.280868
Na	-4.117391	-1.737665	0.271112
H	-0.114684	-2.006864	-0.018632
O	3.907918	-0.852964	-0.792219

C	4.419259	0.152046	0.00401
O	3.831168	1.200154	0.186681
N	5.62546	-0.187219	0.490247
C	6.284536	-1.458298	0.238641
H	7.024219	-1.364396	-0.564819
H	6.798526	-1.771283	1.151615
H	5.560262	-2.223532	-0.030519
C	6.386165	0.802358	1.231571
H	6.603474	0.430852	2.238237
H	7.332684	1.009305	0.720244
H	5.808881	1.721846	1.305661
C	1.022855	2.349818	-1.119121
H	1.270547	3.41422	-1.030841
H	1.891207	1.782935	-0.781926
H	0.851161	2.128497	-2.174613
C	0.987601	1.41586	1.975672
H	1.151217	2.302717	2.599504
H	0.83006	0.557691	2.633223
H	1.891493	1.238053	1.392895
C	-2.047146	1.211907	2.887714
H	-2.900499	1.826749	3.186477
H	-2.351478	0.157981	2.876609
H	-1.257488	1.33324	3.631907
C	-3.894366	1.970901	0.409926
H	-4.236261	3.004359	0.536451
H	-4.302625	1.593148	-0.531142
H	-4.30789	1.380389	1.232218
C	-1.999116	2.666025	-2.061547
H	-2.954262	2.208259	-2.324312
H	-2.122921	3.75511	-2.060752
H	-1.278927	2.404341	-2.838882

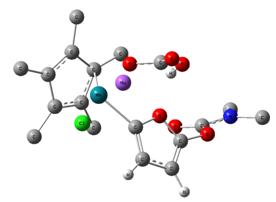

IV-P$_{C5}$

E = -1939.34086665 Eh

C	-1.738122	-0.185854	1.694441
C	0.156605	0.880499	1.959531
C	0.212654	-0.353601	2.672583
H	0.486482	1.813282	2.406812
Rh	1.263863	0.626051	0.056355
C	1.205297	1.462293	-1.960411
C	2.549922	1.158667	-1.620394
C	0.68831	2.388379	-0.979115
C	2.913415	1.962811	-0.459413
C	1.783956	2.741895	-0.092957
Cl	2.803343	-1.173265	0.966052
O	-0.28703	-0.668406	-0.36033
C	0.061679	-1.887004	-0.775054
O	0.881258	-2.007138	-1.745927
O	-0.408553	-2.883132	-0.146285
Na	1.855449	-3.413019	-0.272978
H	1.09027	-0.700427	3.198183
O	-2.92002	-0.449996	1.184146
C	-3.480814	0.500409	0.289758
O	-3.860922	1.570492	0.698867
N	-3.567551	0.004035	-0.95159
C	-3.10664	-1.309483	-1.379827
H	-2.557662	-1.819762	-0.592772
H	-3.967855	-1.913234	-1.687945
H	-2.432631	-1.190072	-2.232905
C	-4.198224	0.819686	-1.973569
H	-3.497684	0.986058	-2.798938
H	-5.084506	0.310391	-2.367428
H	-4.492088	1.777424	-1.548017
O	-1.196613	0.985948	1.516734
C	-0.958705	-1.042113	2.480216
H	-1.21017	-2.05813	2.733994
C	0.449866	0.849825	-3.091949
H	0.888795	1.167112	-4.044657
H	0.505401	-0.2411	-3.009617
H	-0.59849	1.153077	-3.078789
C	-0.6831	2.983754	-0.9904
H	-1.426959	2.231611	-1.262837

H	-0.961104	3.371303	-0.009046
H	-0.740807	3.805988	-1.712826
C	1.730853	3.77096	0.989651
H	2.376157	3.505504	1.83044
H	2.06703	4.737927	0.599597
H	0.714347	3.904841	1.367614
C	4.270314	1.999759	0.16365
H	4.685209	0.992958	0.24742
H	4.949076	2.604669	-0.448266
H	4.23885	2.431493	1.166227
C	3.424713	0.196935	-2.354239
H	3.823452	0.657382	-3.265975
H	4.26451	-0.122936	-1.734452
H	2.842436	-0.68822	-2.623908

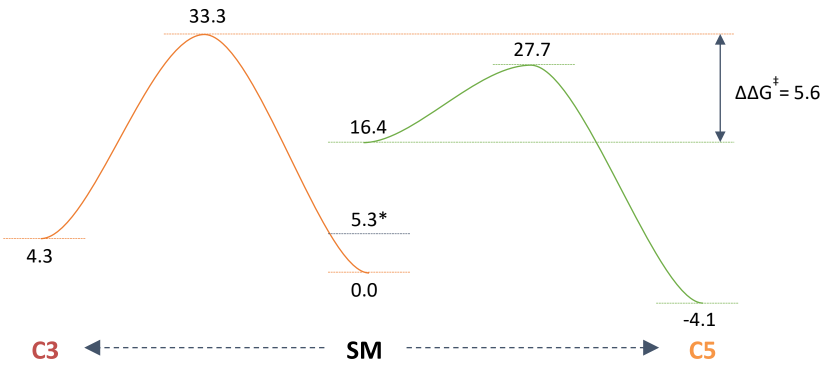

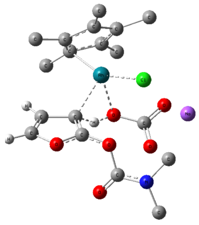

Alternative IV-TS$_{C3}$ (σ-bond metathesis, diastereoisomer A) (c3ab-co3a-cp)

E = -1742.73556608 Eh

C	-1.234839	-2.617116	-2.484009
C	-0.137959	-1.862029	-2.271077
C	-0.41801	-1.011089	-1.130824
C	-1.706892	-1.362764	-0.790318
O	-2.220632	-2.303884	-1.572487
H	-1.4951	-3.385075	-3.194981
H	-0.314099	0.273124	-0.952267
O	-2.495074	-0.859617	0.172294

C	-3.152059	0.328475	-0.194407
O	-3.448346	0.55028	-1.342827
N	-3.408627	1.082993	0.891205
H	0.784141	-1.88783	-2.832495
Rh	1.01677	-0.513486	0.404406
C	1.077328	-0.30988	2.582975
C	2.369078	-0.650471	2.123387
C	0.163852	-1.344874	2.190462
H	0.807211	0.623168	3.05814
C	2.272909	-1.927178	1.454505
H	3.259489	-0.04601	2.216565
C	0.927061	-2.364374	1.524302
H	-0.896697	-1.374427	2.39037
H	3.083961	-2.445841	0.963017
H	0.531924	-3.27329	1.092093
Cl	2.700374	-0.059923	-1.32019
O	-0.077787	1.234188	-0.06613
C	0.709626	2.309692	-0.41223
O	1.556511	2.690361	0.438365
O	0.554128	2.774824	-1.56539
Na	2.846136	2.714208	-1.472257
C	-3.963415	2.407078	0.656277
H	-3.165909	3.154964	0.573491
H	-4.53434	2.399715	-0.270613
H	-4.624665	2.673624	1.484592
C	-2.710436	0.868847	2.150862
H	-1.640217	1.078778	2.044468
H	-3.13597	1.546525	2.893077
H	-2.857185	-0.152775	2.504984

Alternative IV-TS$_{C3}$ (σ-bond metathesis, diastereoisomer B) (c3ab-co3b-cp)

E = -1742.73557633 Eh

C	-0.963579	1.812575	0.319756
C	0.217678	1.553813	-0.347121
C	0.741856	2.88887	-0.597083
H	0.107771	0.60684	-1.19841
Rh	1.626913	-0.073224	0.015048
C	3.537164	-0.807207	-0.756321
C	3.422799	-1.176601	0.600357
C	3.462305	0.625723	-0.846365
H	3.560261	-1.488581	-1.595695
C	3.302503	0.034725	1.381568

H	3.37679	-2.185652	0.983532
C	3.363875	1.135911	0.492786
H	3.519933	1.207798	-1.755254
H	3.174316	0.087405	2.453306
H	3.274146	2.176394	0.772614
Cl	0.233462	-1.220321	1.681913
O	0.493517	-0.581272	-1.715123
C	-0.342163	-1.644786	-1.500033
O	0.186066	-2.785281	-1.442286
O	-1.563083	-1.390499	-1.318992
Na	-1.303465	-2.992648	0.271525
O	-1.919052	0.992989	0.759329
C	-3.025848	0.863106	-0.08
O	-3.1847	1.575691	-1.041982
N	-3.873138	-0.07385	0.390868
C	-5.077444	-0.344188	-0.368513
H	-4.944448	-1.224277	-1.009585
H	-5.907392	-0.526318	0.321268
H	-5.31332	0.513701	-0.99567
C	-3.548205	-1.013712	1.441969
H	-2.514523	-0.888723	1.763
H	-4.209409	-0.877038	2.305369
H	-3.704299	-2.036442	1.069519
O	-1.175691	3.111599	0.515628
C	-0.115226	3.772638	-0.052141
H	-0.145075	4.847972	0.023529
H	1.651104	3.133525	-1.127325

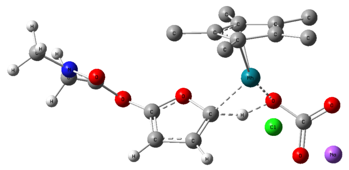

Alternative IV-TS$_{C5}$ (σ-bond metathesis, diastereoisomer A) (c5ab-co3a-cp)

E = -1742.74200211 Eh

C	-2.386981	-0.578592	0.160156
C	-1.996782	-1.446633	1.156263
C	-0.595631	-1.468003	1.063709
C	-0.185724	-0.635195	0.029372
O	-1.369079	-0.079489	-0.511634
H	0.086563	-2.034433	1.681919
H	-2.643813	-1.976449	1.832483
Rh	1.4712	0.694245	0.09147

C	2.291227	2.537597	-0.793421
C	2.700912	2.465316	0.550182
C	0.850048	2.524954	-0.830236
H	2.941503	2.498212	-1.656202
C	1.515366	2.426178	1.379611
H	3.719715	2.384825	0.9011
C	0.384215	2.508765	0.525284
H	0.235854	2.565723	-1.718327
H	1.497524	2.352878	2.457674
H	-0.649935	2.483953	0.839778
Cl	2.66108	-0.793385	1.647011
O	1.708232	-0.59641	-1.595048
C	2.81607	-1.401167	-1.524772
O	3.939718	-0.834696	-1.572138
O	2.615562	-2.631534	-1.359439
Na	4.359239	-2.336968	0.092487
H	0.644429	-0.969461	-0.901315
O	-3.55922	-0.141205	-0.319171
C	-4.747223	-0.460593	0.316258
O	-4.79353	-1.076181	1.35819
N	-5.792047	0.031305	-0.380109
C	-5.695973	0.669988	-1.682353
H	-4.689566	1.0385	-1.863415
H	-5.967859	-0.031265	-2.480029
H	-6.388944	1.515872	-1.711909
C	-7.133608	-0.262292	0.091365
H	-7.716275	0.662642	0.144506
H	-7.636546	-0.956048	-0.592217
H	-7.080724	-0.711802	1.080958

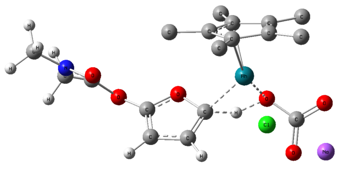

Alternative IV-TS$_{C5}$ (σ-bond metathesis, diastereoisomer B) (c5ab-co3b-CP)

E = -1742.74200207 Eh

C	-2.35561	-0.579768	0.237007
C	-1.91811	-1.399876	1.253857
C	-0.518757	-1.373655	1.138178
C	-0.156103	-0.561096	0.070625
O	-1.367462	-0.066613	-0.467813
H	0.193454	-1.894389	1.762685
H	-2.534474	-1.930147	1.9578

Rh	1.451628	0.828819	0.06176
C	2.189288	2.670776	-0.895561
C	2.622387	2.657361	0.442561
C	0.74915	2.604679	-0.907614
H	2.826951	2.62684	-1.767488
C	1.45225	2.602359	1.292719
H	3.648845	2.625385	0.778637
C	0.305558	2.615929	0.455389
H	0.120015	2.59407	-1.786065
H	1.454038	2.563758	2.372727
H	-0.721919	2.563904	0.787921
Cl	2.71995	-0.563156	1.64367
O	1.709158	-0.506897	-1.586216
C	2.846912	-1.268121	-1.509171
O	3.947998	-0.662988	-1.593701
O	2.694584	-2.498943	-1.301305
Na	4.448955	-2.094086	0.110661
H	0.670999	-0.895374	-0.86283
O	-3.55055	-0.201069	-0.236188
C	-4.715793	-0.541743	0.429605
O	-4.722943	-1.123599	1.491728
N	-5.788872	-0.111192	-0.264426
C	-5.73693	0.486671	-1.58838
H	-6.461093	1.305273	-1.633952
H	-4.747804	0.88538	-1.798403
H	-5.995151	-0.250279	-2.35792
C	-7.111106	-0.437224	0.239248
H	-7.726569	0.467203	0.271411
H	-7.598819	-1.171578	-0.412205
H	-7.026113	-0.850697	1.242229

8. Distortion-interaction analysis

Distortion-interaction analysis was performed on the cationic and neutral acetate transition states by selecting twenty geometries along each IRC, partitioning each structure into Rh catalyst and substrate fragments, and carrying out single-point calculations on the frozen fragments at the same level of theory. E_{int} was defined as the difference between the energy of the parent structure and the sum of the fragment energies, and E_{dis} as the difference between the energies of the distorted fragments and those of the corresponding ground-state optimized fragments.

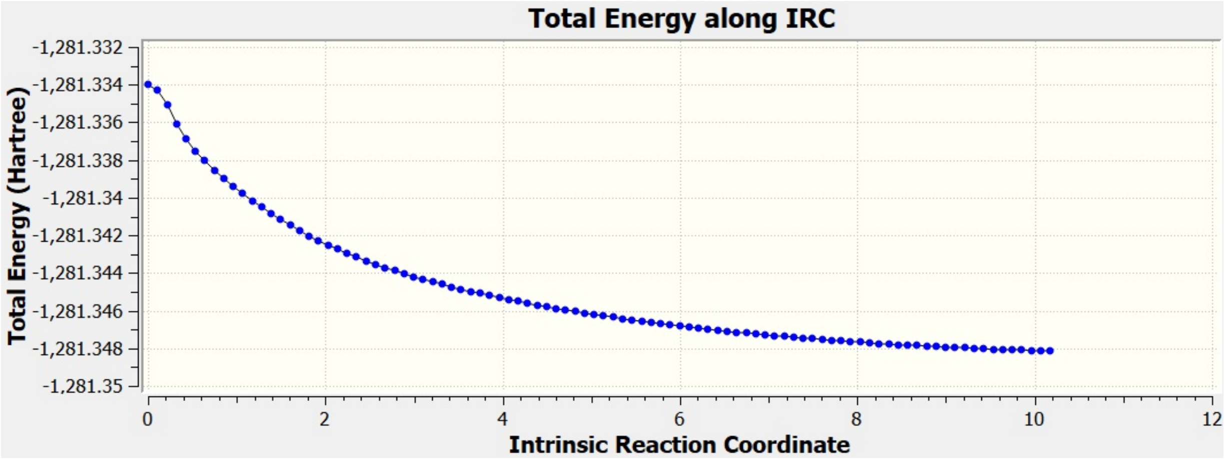

Figure S1. IRC scan along the potenital energy surfuce in one direction connecting a transtion state to a product or starting material, 13 geometries were selected along to be fragmented and submitted to Single Point calculations SPC.

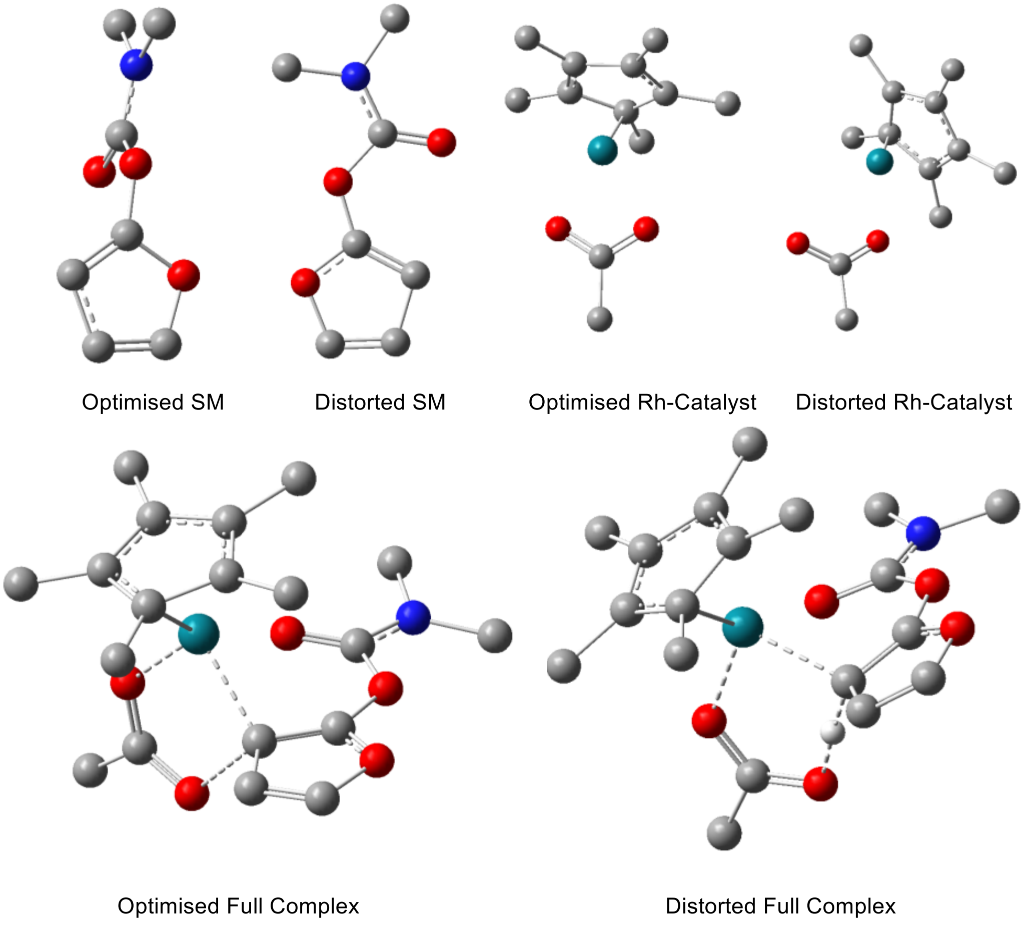

Figure S2. Optimised and single point calculated starting materials, Rh catalyst and Full system.

	E (Hartree)			ΔE (kcal/mol)				
**C–H**	**E**	**E_{dist} SM**	**E_{dist} Rh**	**ΔE**	**E_{dist} SM**	**E_{dist} Rh**	**Total distortion**	**Interaction energy**
**1.09285**	-1281.34879	-552.40932	-728.89131	-13.7	0.6	16.0	16.5	-30.2
**1.09393**	-1281.34856	-552.40925	-728.89178	-13.5	0.6	15.7	16.3	-29.8
**1.0954**	-1281.34794	-552.40912	-728.89284	-13.1	0.7	15.0	15.7	-28.9
**1.09712**	-1281.34734	-552.4086	-728.89344	-12.8	1.0	14.6	15.7	-28.4
**1.10115**	-1281.34605	-552.40661	-728.894	-12.0	2.3	14.3	16.6	-28.5
**1.10495**	-1281.34474	-552.40479	-728.89429	-11.1	3.4	14.1	17.5	-28.7
**1.11148**	-1281.34263	-552.40163	-728.89463	-9.8	5.4	13.9	19.3	-29.1
**1.12088**	-1281.34053	-552.39782	-728.89488	-8.5	7.8	13.7	21.5	-30.0
**1.13646**	-1281.33872	-552.39415	-728.89488	-7.4	10.1	13.7	23.8	-31.2
**1.14328**	-1281.33726	-552.39153	-728.89476	-6.4	11.7	13.8	25.5	-32.0
**1.16542**	-1281.3358	-552.38725	-728.89436	-5.5	14.4	14.1	28.5	-34.0
**1.27771**	-1281.33424	-552.37089	-728.89291	-4.5	24.7	15.0	39.7	-44.2
**1.33133**	-1281.33399	-552.36165	-728.89235	-4.4	30.5	15.3	45.8	-50.2
**1.49454**	-1281.33608	-552.3299	-728.89024	-5.7	50.4	16.6	67.1	-72.8
**1.69468**	-1281.34045	-552.28589	-728.88502	-8.4	78.0	19.9	97.9	-106.4
**1.75422**	-1281.34194	-552.27255	-728.88347	-9.4	86.4	20.9	107.3	-116.7
**1.81081**	-1281.34309	-552.26032	-728.88203	-10.1	94.1	21.8	115.9	-126.0
**1.87276**	-1281.34425	-552.24704	-728.88039	-10.8	102.4	22.8	125.2	-136.1
**1.91359**	-1281.34512	-552.238	-728.87917	-11.4	108.1	23.6	131.7	-143.0
**1.9383**	-1281.34566	-552.23202	-728.8783	-11.7	111.8	24.1	136.0	-147.7
**1.95448**	-1281.34652	-552.22664	-728.87741	-12.2	115.2	24.7	139.9	-152.1
**1.96032**	-1281.3471	-552.22305	-728.87683	-12.6	117.5	25.1	142.5	-155.1
**1.96668**	-1281.34784	-552.21877	-728.87621	-13.1	120.1	25.4	145.6	-158.7
**1.97322**	-1281.34819	-552.21554	-728.87577	-13.3	122.2	25.7	147.9	-161.2

Table S1. Cationic C3 Distortion-Interaction analysis.

	E (Hartree)			ΔE (kcal/mol)				
**C–H**	**E**	**E_{dist} SM**	**E_{dist} Rh**	**ΔE**	**E_{dist} SM**	**E_{dist} Rh**	**Total distortion**	**Interaction energy**
**1.0879**	-1281.3396	-552.41203	-728.89846	-7.9	-1.1	11.5	10.4	-18.3
**1.0899**	-1281.33975	-552.41165	-728.89867	-8.0	-0.9	11.4	10.5	-18.5
**1.0936**	-1281.339	-552.41035	-728.89806	-7.5	-0.1	11.7	11.7	-19.2
**1.09541**	-1281.33829	-552.40969	-728.8976	-7.1	0.3	12.0	12.4	-19.5
**1.09909**	-1281.33706	-552.40816	-728.89683	-6.3	1.3	12.5	13.8	-20.1
**1.10479**	-1281.3352	-552.40475	-728.89585	-5.1	3.4	13.1	16.6	-21.7
**1.11017**	-1281.33353	-552.40186	-728.89524	-4.1	5.3	13.5	18.8	-22.9
**1.1285**	-1281.3293	-552.39667	-728.89457	-1.4	8.5	13.9	22.4	-23.9
**1.13483**	-1281.32758	-552.39573	-728.8944	-0.4	9.1	14.0	23.1	-23.5
**1.16171**	-1281.32547	-552.39255	-728.89385	1.0	11.1	14.4	25.5	-24.5
**1.29127**	-1281.32271	-552.37535	-728.89267	2.7	21.9	15.1	37.0	-34.3
**1.36633**	-1281.32208	-552.36235	-728.89199	3.1	30.0	15.5	45.6	-42.5
**1.43076**	-1281.32256	-552.35025	-728.89133	2.8	37.6	16.0	53.6	-50.8
**1.55947**	-1281.32491	-552.32523	-728.88958	1.3	53.3	17.1	70.4	-69.1
**1.66167**	-1281.327	-552.30542	-728.88678	0.0	65.8	18.8	84.6	-84.6
**1.71277**	-1281.32846	-552.29504	-728.88537	-0.9	72.3	19.7	92.0	-92.9
**1.78173**	-1281.3299	-552.28142	-728.88368	-1.8	80.8	20.8	101.6	-103.4
**1.85682**	-1281.33115	-552.26724	-728.88198	-2.6	89.7	21.8	111.6	-114.2
**1.93955**	-1281.33226	-552.25224	-728.88044	-3.3	99.1	22.8	121.9	-125.2
**2.01622**	-1281.33314	-552.23874	-728.87931	-3.9	107.6	23.5	131.1	-135.0
**2.06017**	-1281.33368	-552.23101	-728.87878	-4.2	112.5	23.8	136.3	-140.5
**2.08259**	-1281.33396	-552.22702	-728.87856	-4.4	115.0	24.0	138.9	-143.3
**2.10126**	-1281.33428	-552.2234	-728.87835	-4.6	117.2	24.1	141.3	-145.9
**2.1126**	-1281.33449	-552.2216	-728.87822	-4.7	118.4	24.2	142.6	-147.3

Table S2. Cationic C5 distortion interaction analysis.

	E (Hartree)			ΔE (kcal/mol)				
**C–H**	**E**	**E_{dist} SM**	**E_{dist} Rh**	**ΔE**	**E_{dist} SM**	**E_{dist} Rh**	**Total distortion**	**Interaction energy**
**1.09072**	-1741.69082	-552.40467	-1189.2487	4.7	3.5	24.7	28.2	-23.5
**1.09179**	-1741.69061	-552.40462	-1189.2496	4.9	3.5	24.2	27.7	-22.8
**1.09275**	-1741.69025	-552.40453	-1189.2506	5.1	3.6	23.5	27.1	-22.1
**1.09496**	-1741.6897	-552.40385	-1189.2514	5.4	4.0	23.1	27.1	-21.6
**1.09749**	-1741.68911	-552.40282	-1189.2518	5.8	4.7	22.8	27.5	-21.7
**1.10104**	-1741.68825	-552.40125	-1189.2519	6.3	5.6	22.7	28.4	-22.0
**1.10727**	-1741.68684	-552.39876	-1189.2519	7.2	7.2	22.7	29.9	-22.7
**1.11579**	-1741.68514	-552.39547	-1189.2517	8.3	9.3	22.9	32.1	-23.8
**1.13958**	-1741.68245	-552.38921	-1189.251	10.0	13.2	23.3	36.5	-26.5
**1.15435**	-1741.68086	-552.38488	-1189.2504	11.0	15.9	23.6	39.6	-28.6
**1.18112**	-1741.6799	-552.37971	-1189.2497	11.6	19.2	24.1	43.2	-31.7
**1.25112**	-1741.67911	-552.36925	-1189.2487	12.1	25.7	24.8	50.5	-38.4
**1.30234**	-1741.67892	-552.36105	-1189.2481	12.2	30.9	25.1	56.0	-43.8
**1.30234**	-1741.67892	-552.36105	-1189.2481	12.2	30.9	25.1	56.0	-43.8
**1.35365**	-1741.67919	-552.35199	-1189.2474	12.0	36.6	25.6	62.1	-50.1
**1.4635**	-1741.68143	-552.33128	-1189.246	10.6	49.5	26.5	76.0	-65.4
**1.59851**	-1741.68487	-552.30457	-1189.2426	8.5	66.3	28.6	94.9	-86.4
**1.68242**	-1741.68743	-552.28711	-1189.2402	6.8	77.3	30.1	107.3	-100.5
**1.77693**	-1741.69022	-552.26763	-1189.2374	5.1	89.5	31.8	121.3	-116.2
**1.95435**	-1741.6941	-552.23383	-1189.2319	2.7	110.7	35.3	146.0	-143.3
**2.13383**	-1741.69726	-552.20378	-1189.2249	0.7	129.6	39.7	169.2	-168.5
**2.3848**	-1741.69852	-552.17578	-1189.2207	-0.1	147.1	42.3	189.4	-189.5
**2.75647**	-1741.70059	-552.14432	-1189.2159	-1.4	166.9	45.3	212.2	-213.6
**3.00028**	-1741.70229	-552.12827	-1189.213	-2.5	176.9	47.2	224.1	-226.6
**3.09314**	-1741.70358	-552.12334	-1189.2127	-3.3	180.0	47.3	227.3	-230.6
**3.11164**	-1741.70452	-552.12189	-1189.2135	-3.9	180.9	46.8	227.8	-231.7

Table S3. Neutral C3 transition state Distortion-Interaction analysis.

	E (Hartree)			ΔE (kcal/mol)				
**C–H**	**E**	**E_{dist} SM**	**E_{dist} Rh**	**ΔE**	**E_{dist} SM**	**E_{dist} Rh**	**Total distortion**	**Interaction energy**
**1.10066**	-1741.69165	-552.40346	-1189.2522	4.2	4.3	22.5	26.8	-22.6
**1.10346**	-1741.69136	-552.40238	-1189.2526	4.4	4.9	22.3	27.2	-22.8
**1.1056**	-1741.69088	-552.40155	-1189.2525	4.7	5.4	22.3	27.8	-23.1
**1.10799**	-1741.6905	-552.40044	-1189.2526	4.9	6.1	22.3	28.4	-23.5
**1.11268**	-1741.68994	-552.3983	-1189.2528	5.3	7.5	22.2	29.6	-24.4
**1.11763**	-1741.68936	-552.39607	-1189.2528	5.6	8.9	22.1	31.0	-25.4
**1.12371**	-1741.68859	-552.39331	-1189.2527	6.1	10.6	22.2	32.8	-26.7
**1.13442**	-1741.68753	-552.38922	-1189.2524	6.8	13.2	22.4	35.6	-28.8
**1.14014**	-1741.68695	-552.38693	-1189.2522	7.1	14.6	22.5	37.2	-30.0
**1.1645**	-1741.68563	-552.38128	-1189.2515	8.0	18.2	23.0	41.1	-33.2
**1.1875**	-1741.68516	-552.37795	-1189.2511	8.3	20.3	23.3	43.5	-35.2
**1.23068**	-1741.68478	-552.37173	-1189.2502	8.5	24.2	23.8	47.9	-39.4
**1.28203**	-1741.68467	-552.36402	-1189.2496	8.6	29.0	24.2	53.2	-44.6
**1.3335**	-1741.68488	-552.35527	-1189.2489	8.4	34.5	24.6	59.1	-50.7
**1.44669**	-1741.68699	-552.33423	-1189.2474	7.1	47.7	25.5	73.2	-66.1
**1.58844**	-1741.69047	-552.3061	-1189.2439	4.9	65.3	27.7	93.1	-88.1
**1.69008**	-1741.69337	-552.28494	-1189.2409	3.1	78.6	29.6	108.3	-105.1
**1.79522**	-1741.69601	-552.26372	-1189.2378	1.5	91.9	31.6	123.5	-122.1
**1.92455**	-1741.69857	-552.23953	-1189.2336	-0.1	107.1	34.2	141.4	-141.5
**1.98545**	-1741.6998	-552.22845	-1189.231	-0.9	114.1	35.9	149.9	-150.8
**2.01464**	-1741.70082	-552.22247	-1189.229	-1.6	117.8	37.1	154.9	-156.4
**2.02367**	-1741.70129	-552.22019	-1189.2282	-1.9	119.3	37.6	156.9	-158.7
**2.03067**	-1741.70171	-552.21822	-1189.2274	-2.1	120.5	38.1	158.6	-160.7
**2.03872**	-1741.70202	-552.21655	-1189.2269	-2.3	121.5	38.4	160.0	-162.3
**2.04613**	-1741.70227	-552.21511	-1189.2265	-2.5	122.4	38.7	161.1	-163.6

Table S4. Neutral C5 transition state Distortion-Interaction analysis.

9. NMR data

^{1}H NMR spectrum furan-2-yl-dimethylcarbamate (1a)

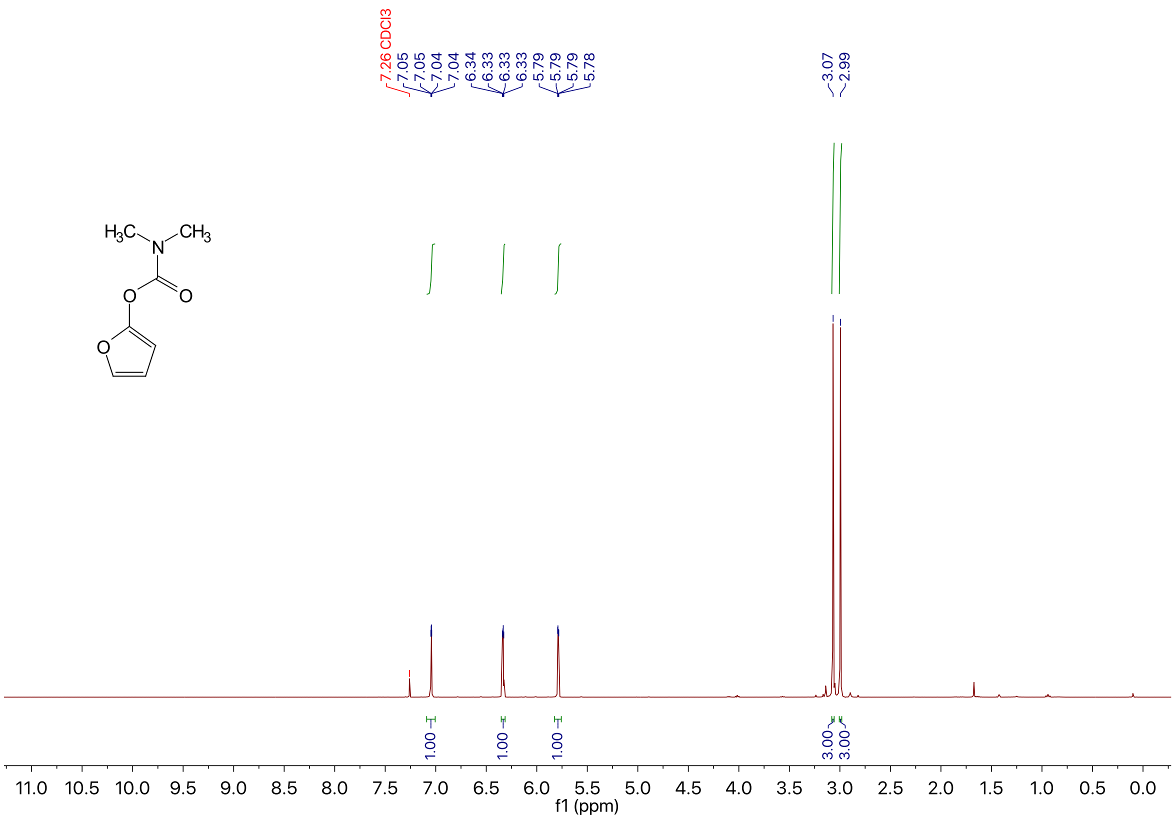

^{13}C NMR spectrum of furan-2-yl-dimethylcarbamate (1a)

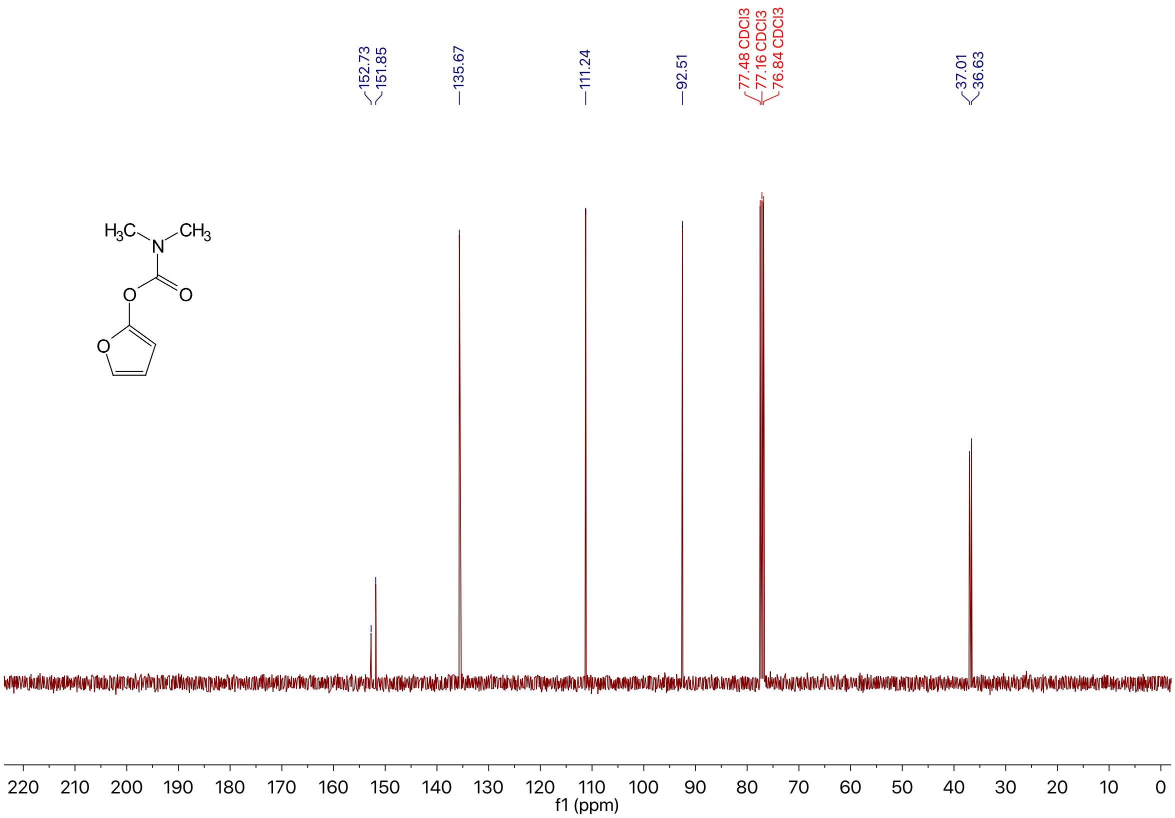

^1H NMR spectrum of furan-2-yl-diethylcarbamate

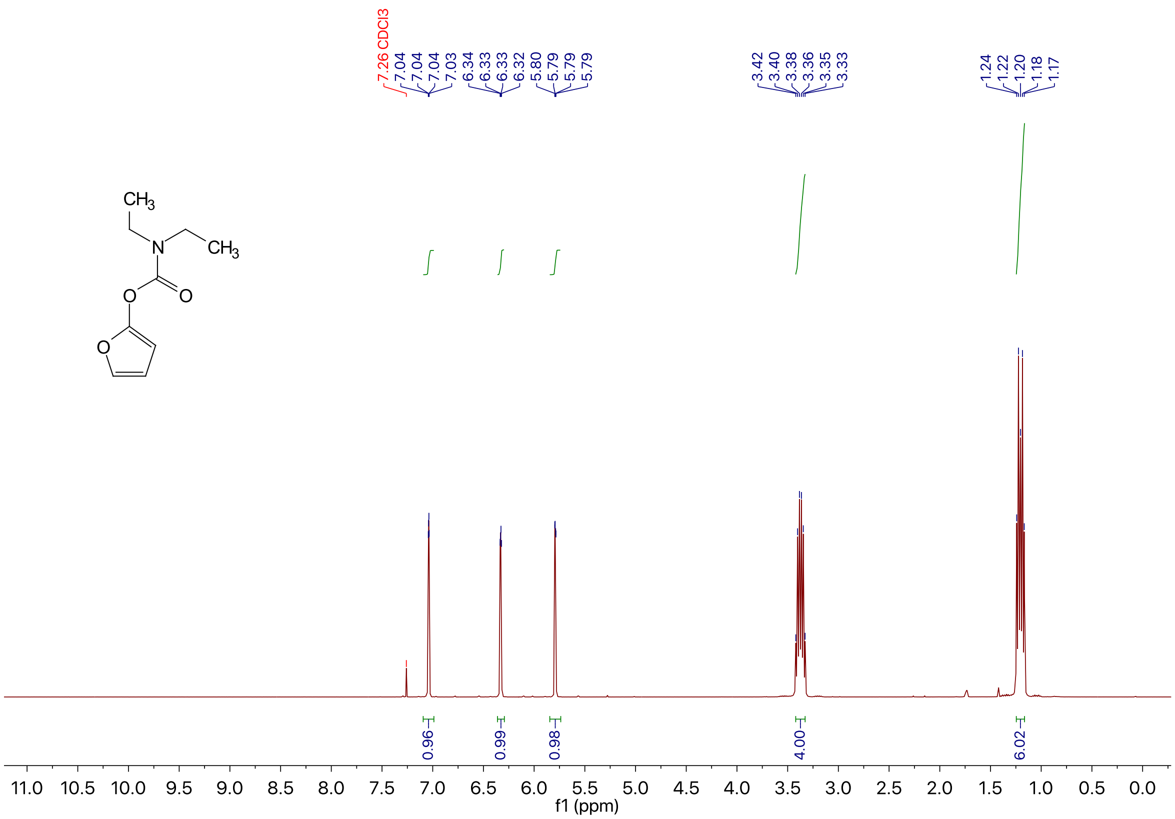

^{13}C NMR spectrum of furan-2-yl-diethylcarbamate

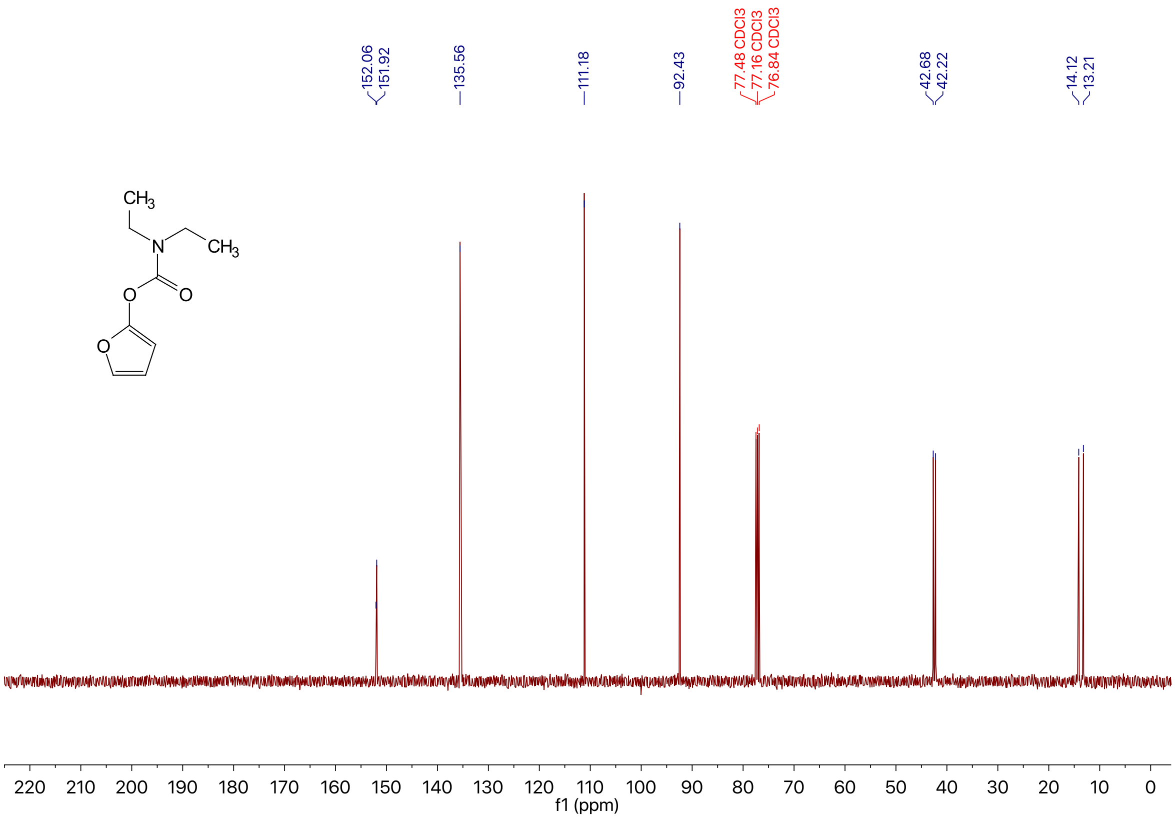

^{1}H NMR spectrum of furan-2-yl-diisopropylcarbamate

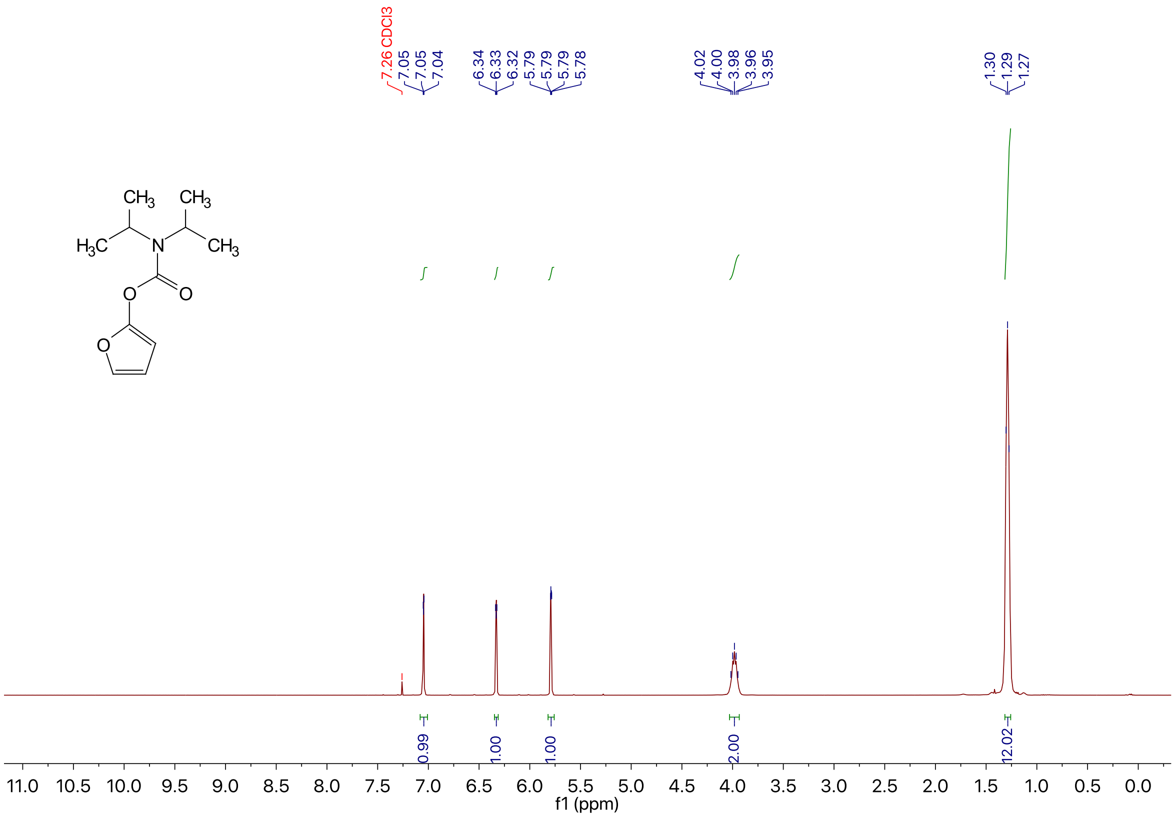

^{13}C NMR spectrum of furan-2-yl-diisopropylcarbamate

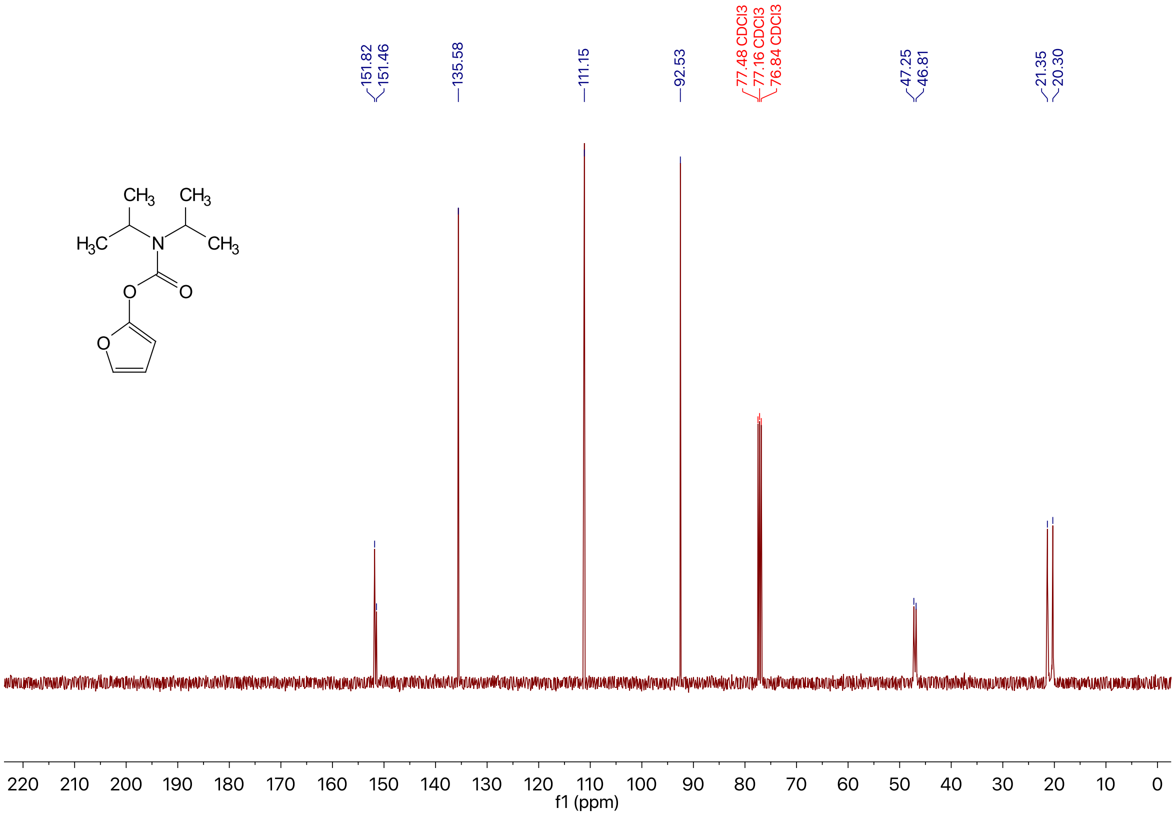

^{1}H NMR spectrum of furan-2-yl-pyrrolidine-1-carboxylate

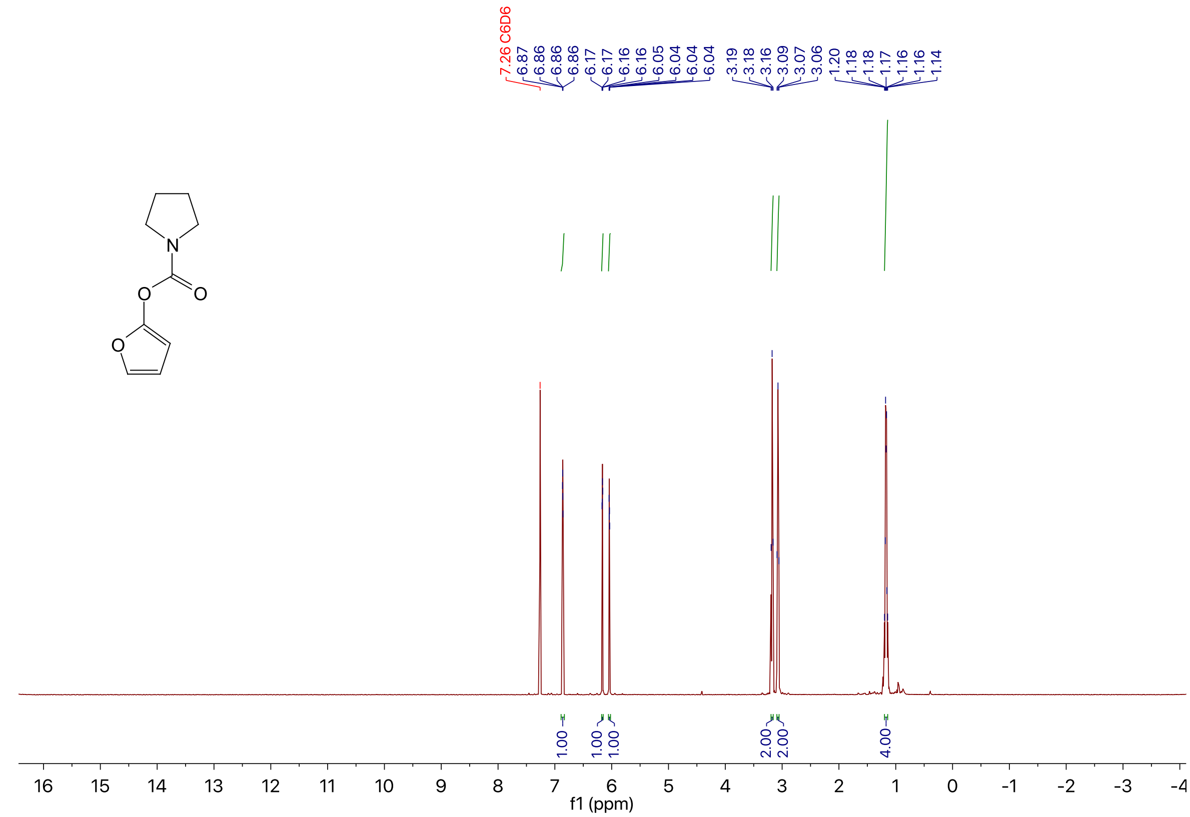

^{13}C NMR spectrum of furan-2-yl-pyrrolidine-1-carboxylate

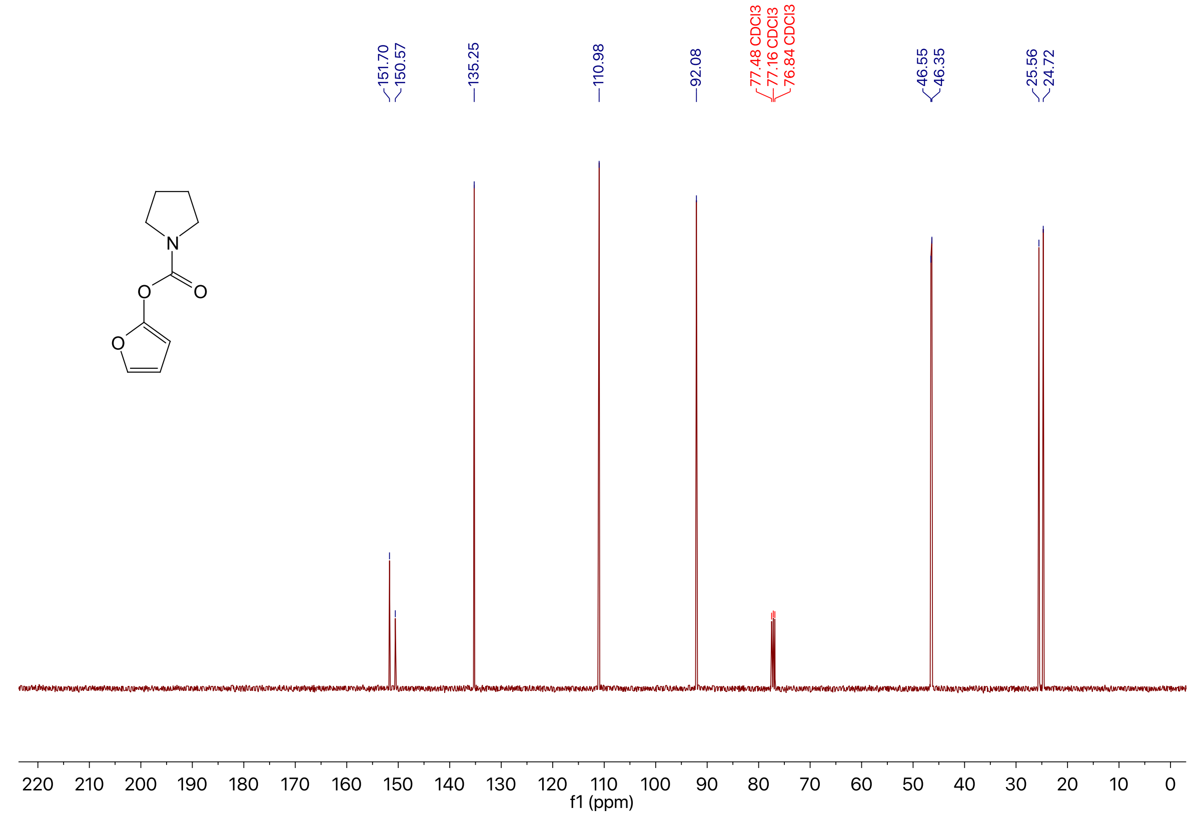

^{1}H NMR spectrum of furan-2-yl-morpholine-4-carboxylate

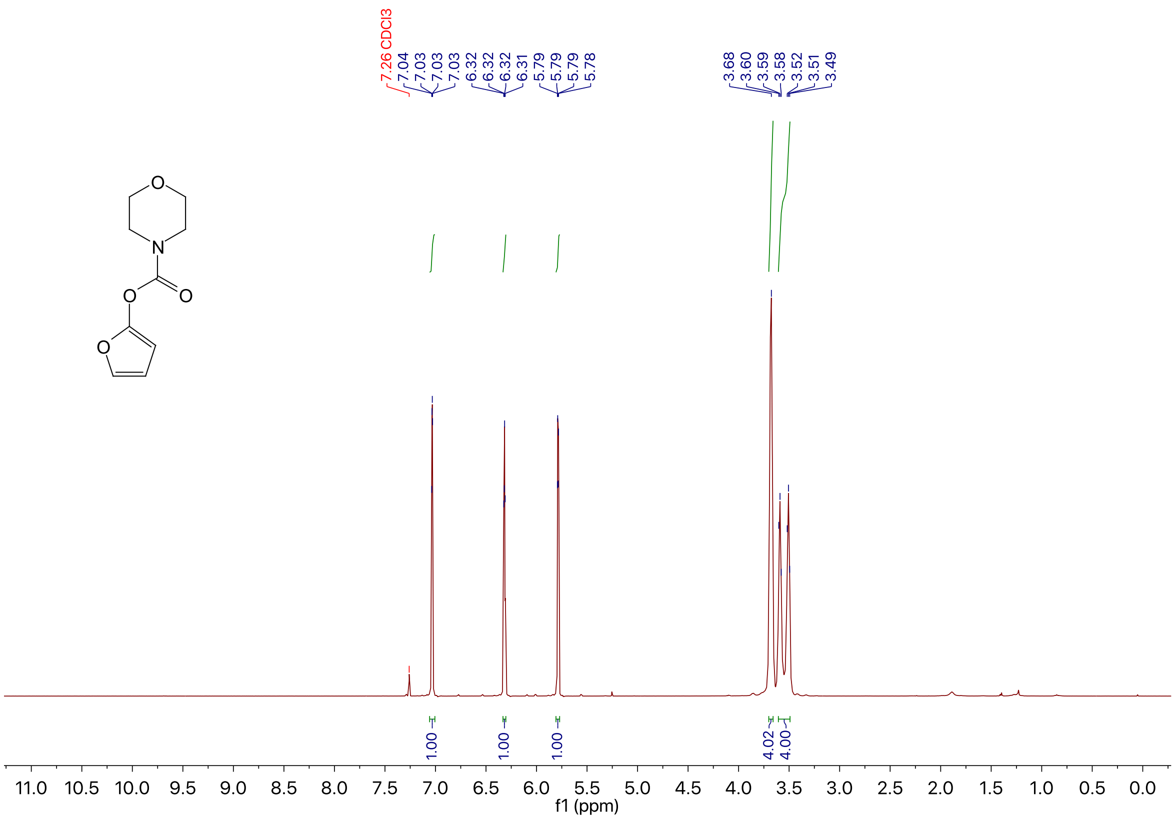

^{13}C NMR spectrum of furan-2-yl-morpholine-4-carboxylate

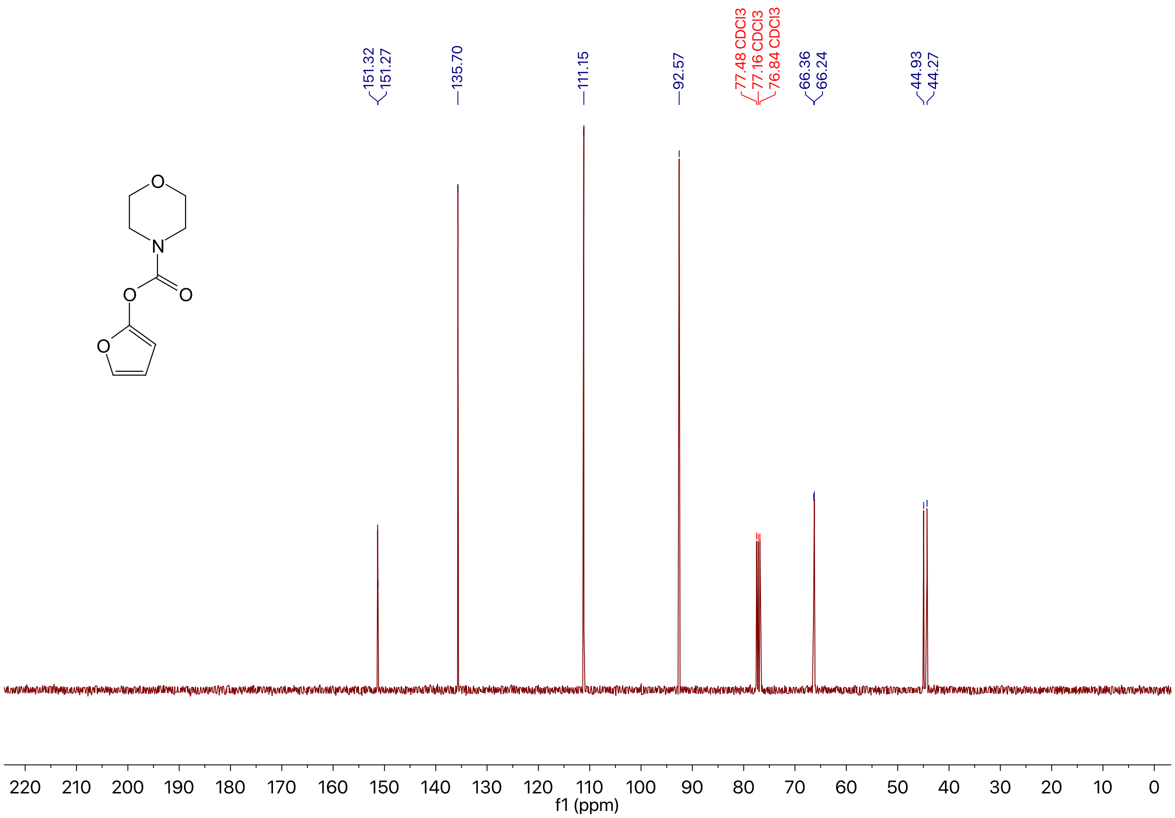

^{1}H NMR spectrum of 5-butylfuran-2-yl-dimethylcarbamate (1b)

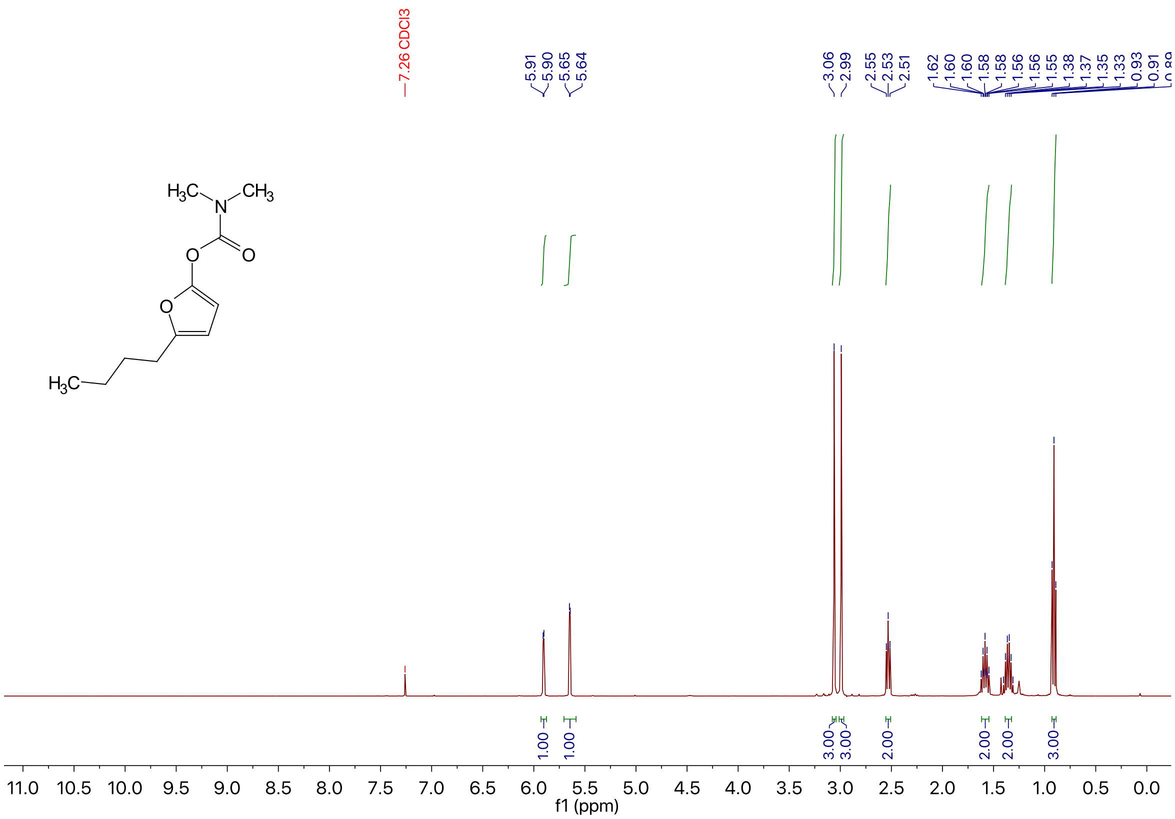

^{13}C NMR spectrum of 5-butylfuran-2-yl-dimethylcarbamate (1b)

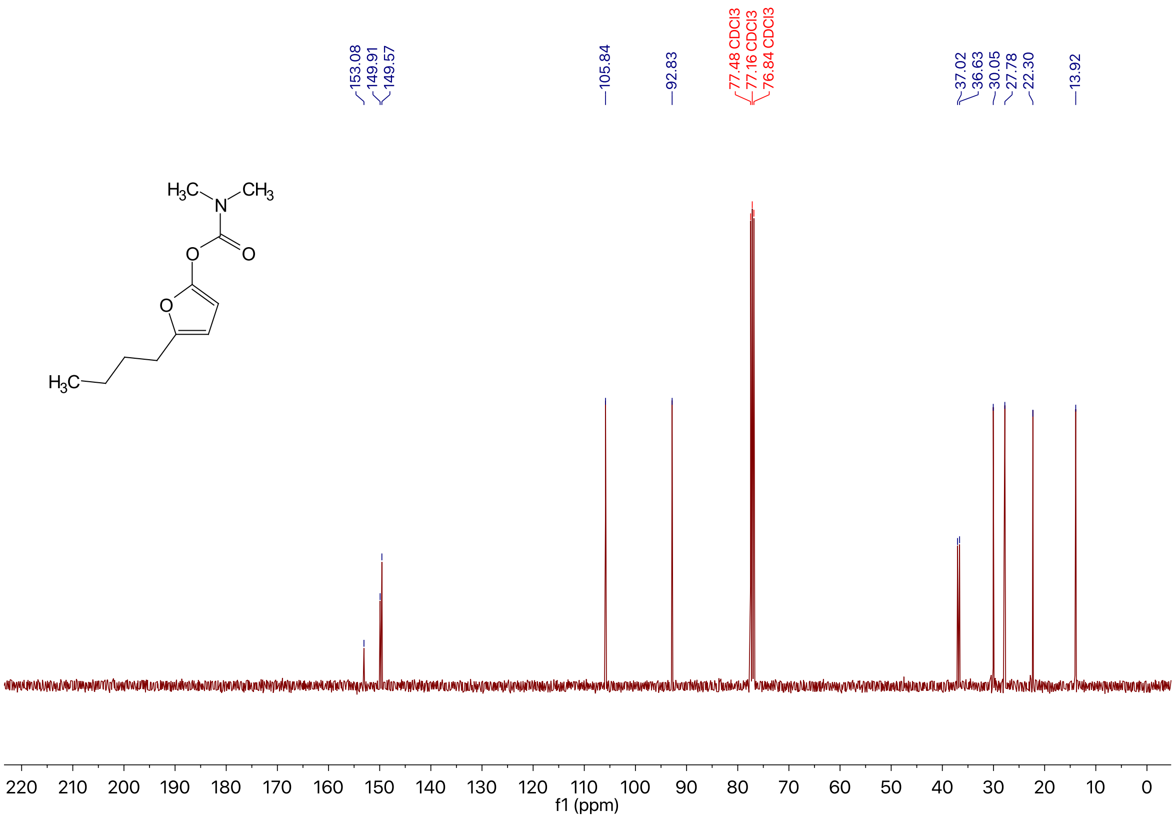

^{1}H NMR spectrum of 5-phenylfuran-2-yl-dimethylcarbamate (1c)

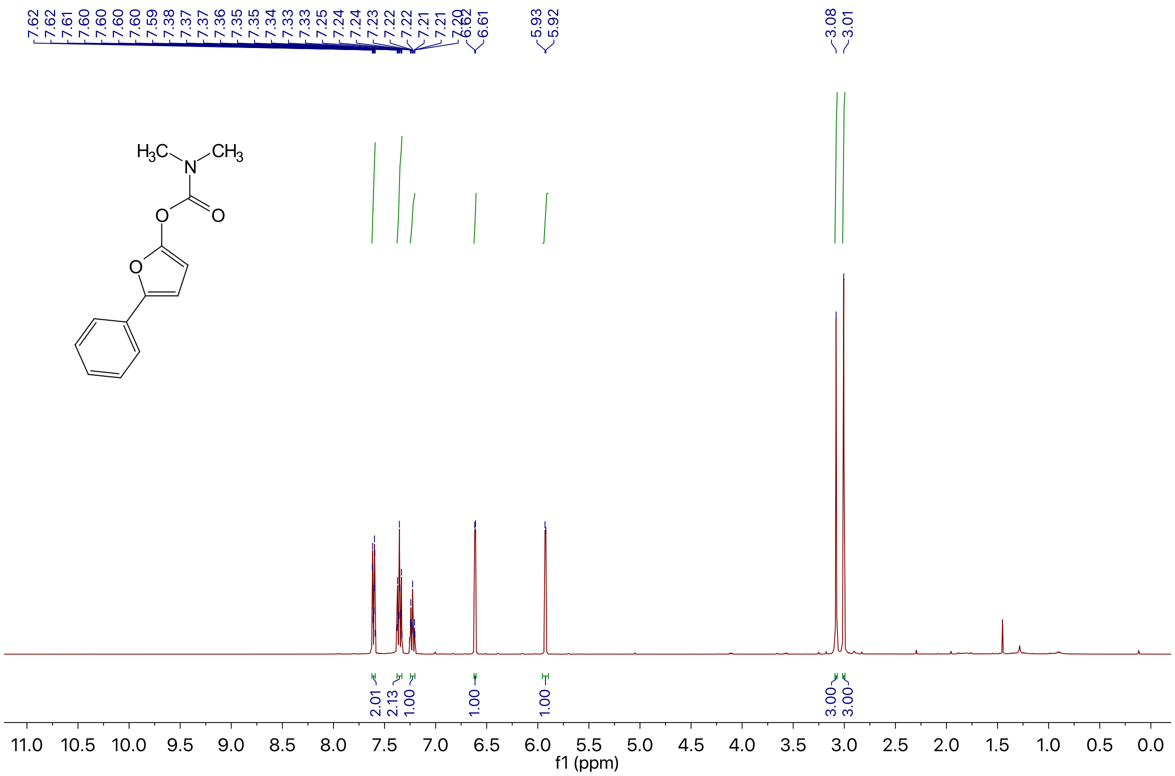

^{13}C NMR spectrum of 5-phenylfuran-2-yl-dimethylcarbamate (1c)

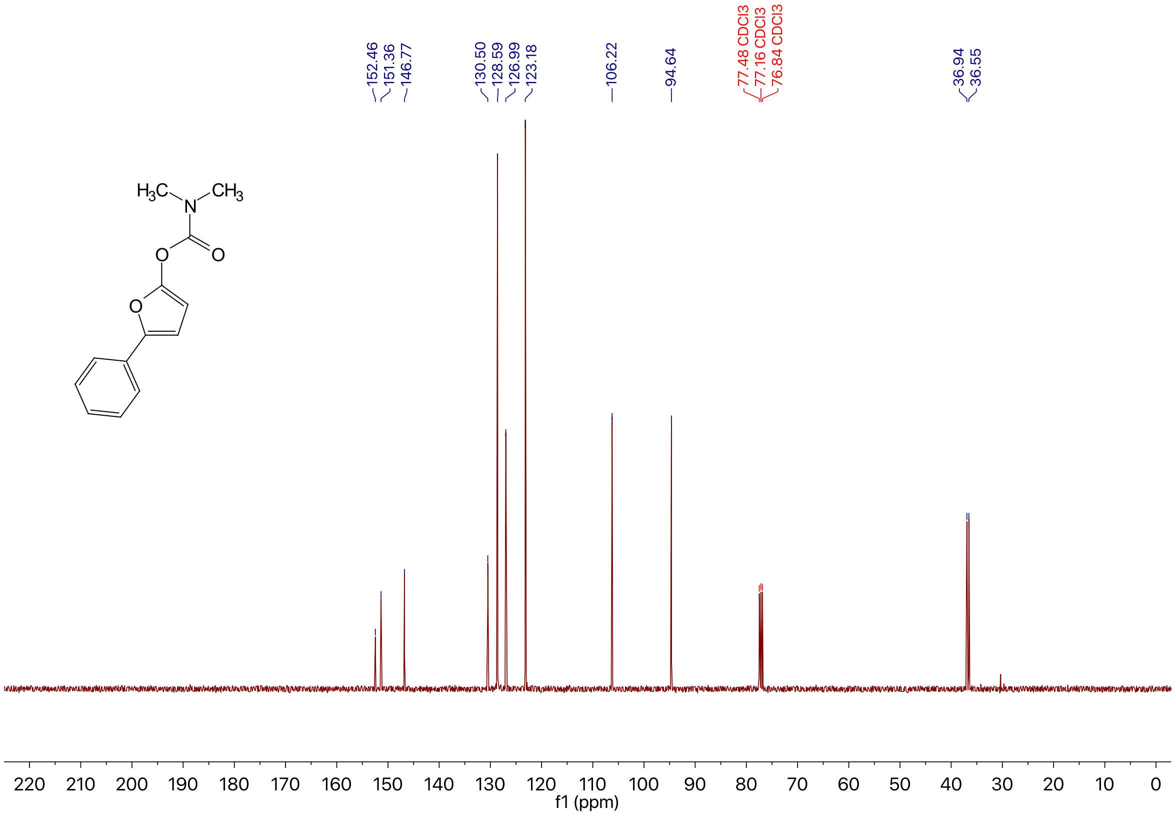

^{1}H NMR spectrum of 5-(*p*-tolyl)furan-2-yl-dimethylcarbamate (1d)

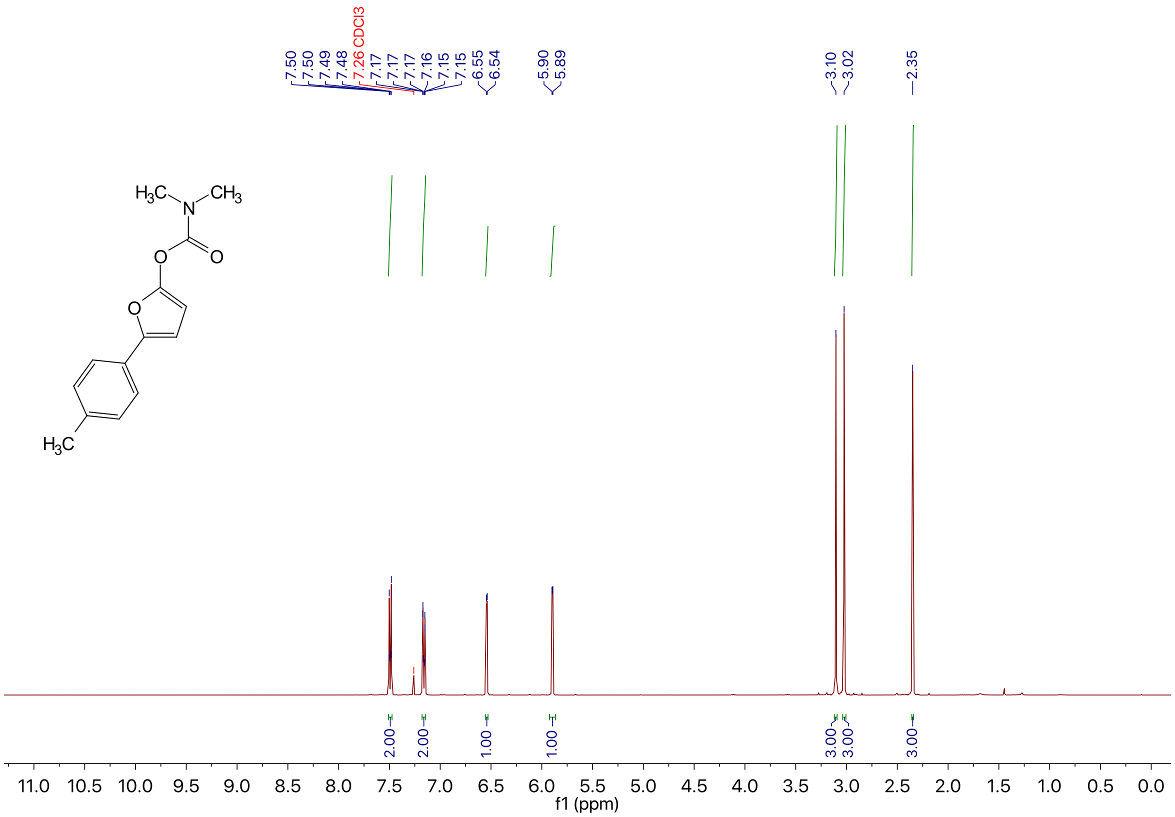

^{13}C NMR spectrum of 5-(*p*-tolyl)furan-2-yl-dimethylcarbamate (1d)

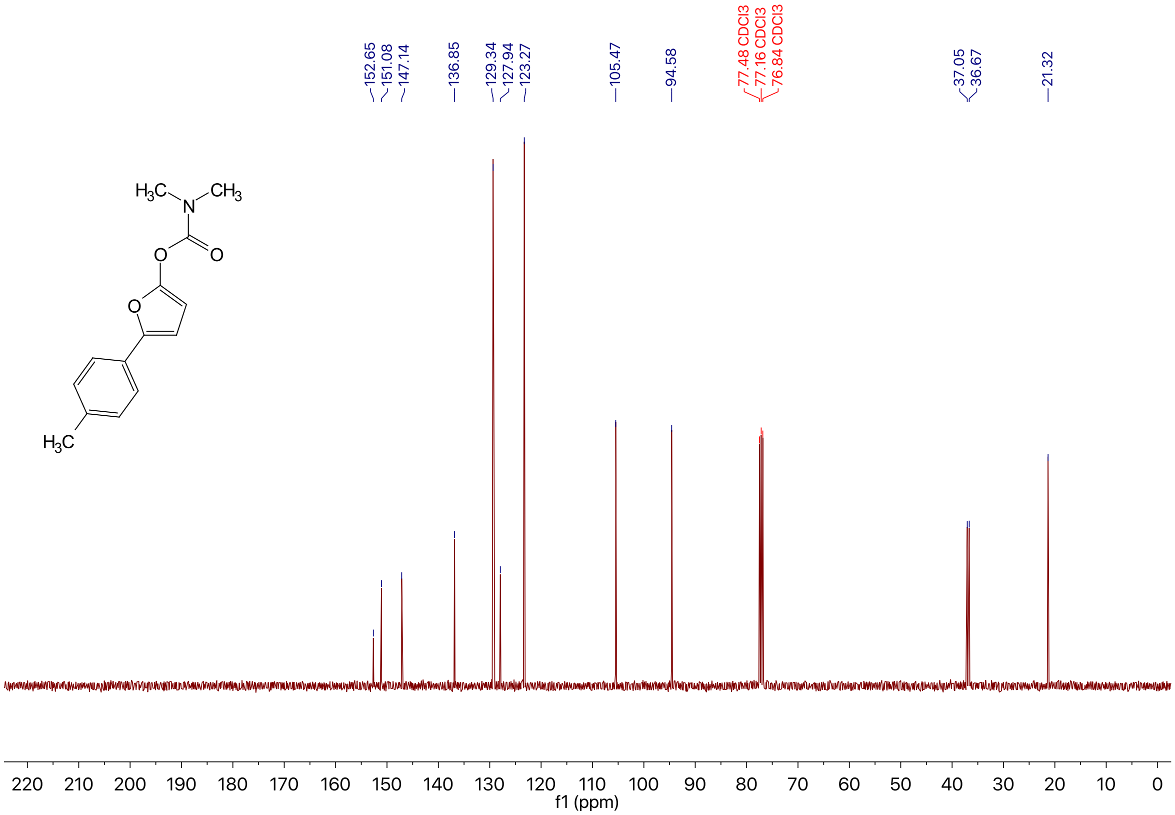

^{1}H NMR spectrum of 5-(4-(trifluoromethyl)phenyl)furan-2-yl-dimethylcarbamate (1e)

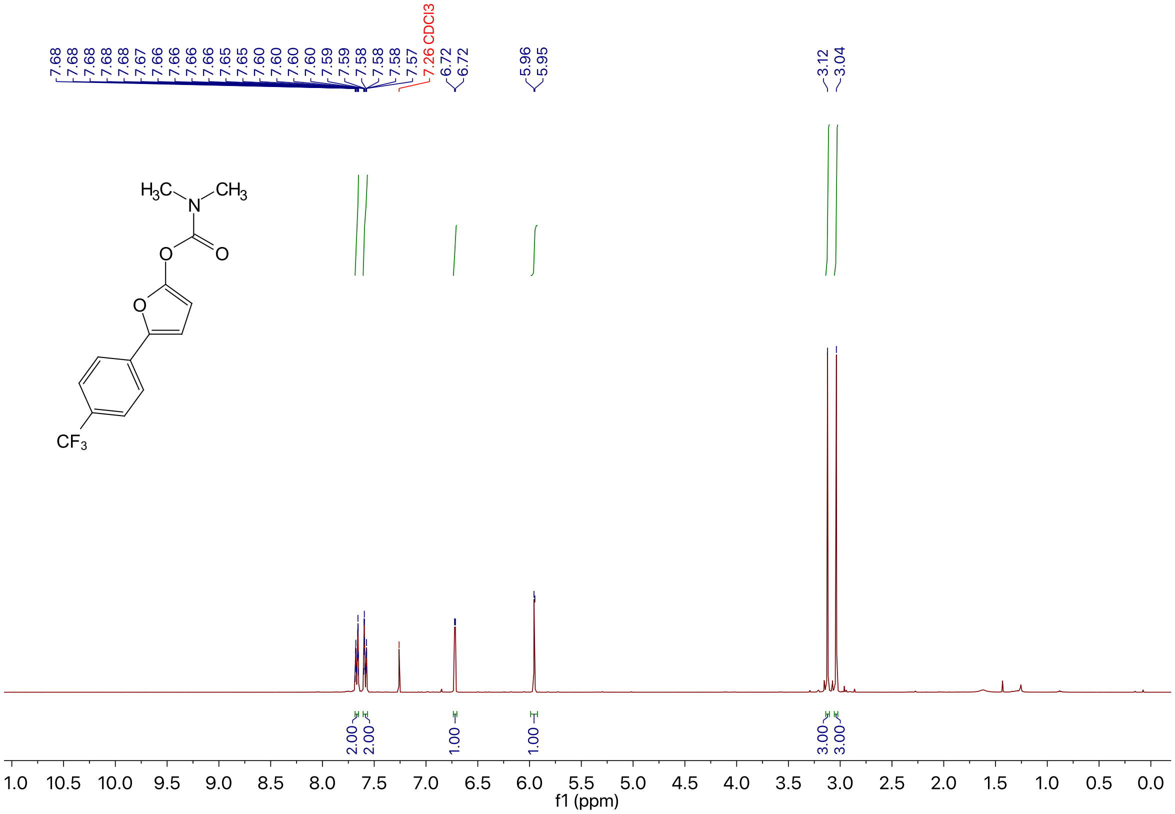

^{13}C NMR Spectrum of 5-(4-(trifluoromethyl)phenyl)furan-2-yl-dimethylcarbamate (1e)

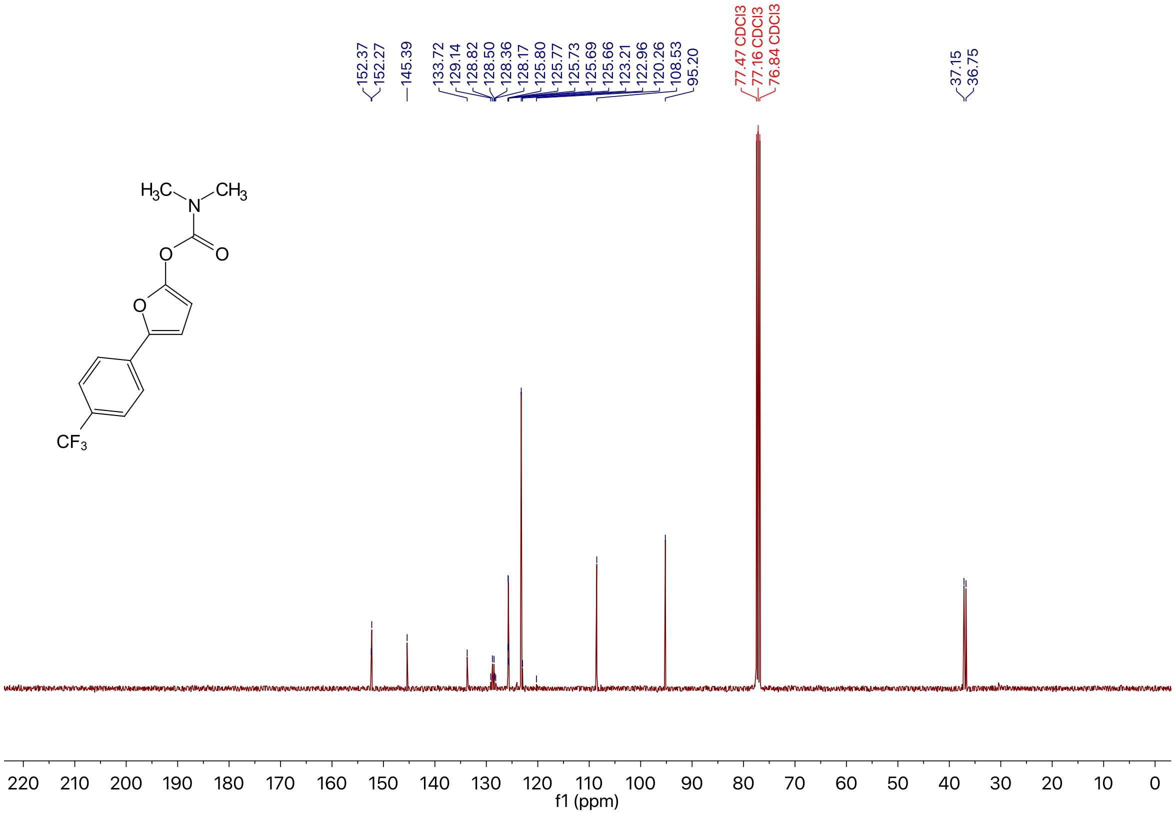

^{1}H NMR spectrum of 3-methylfuran-2-yl-dimethylcarbamate (1f)

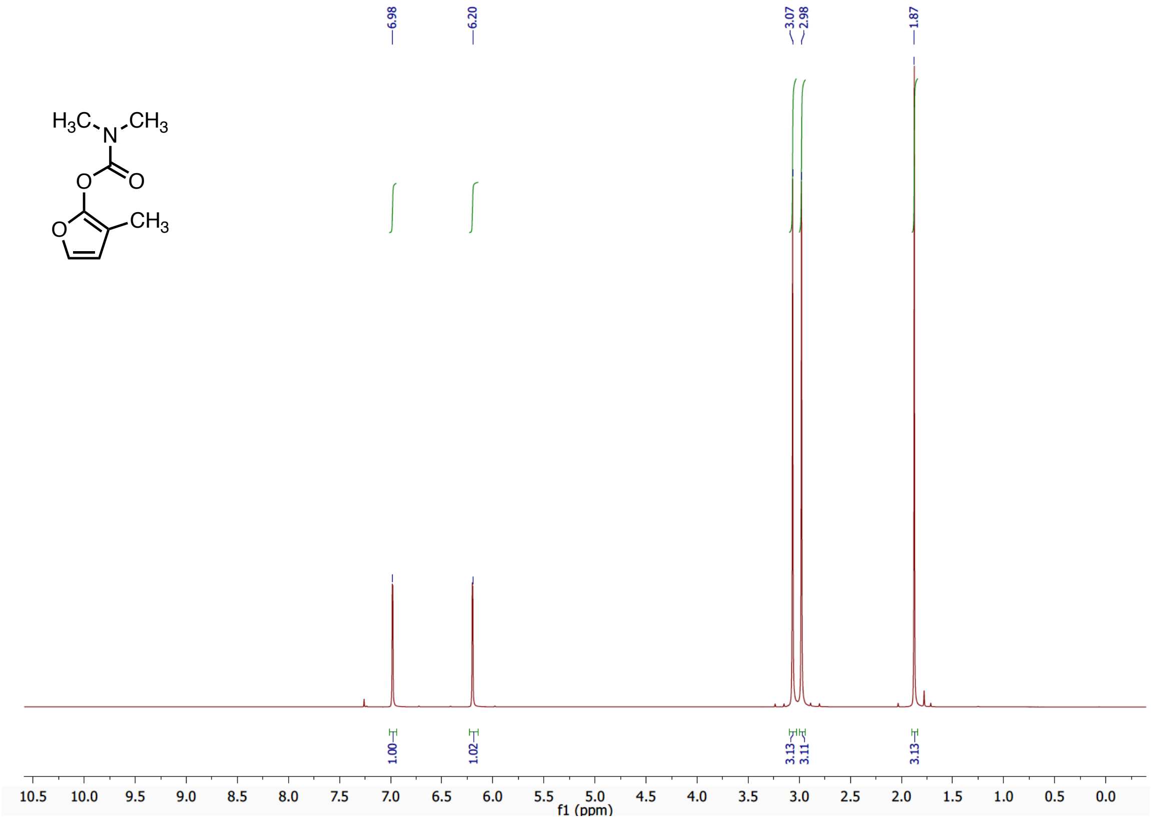

^{13}C NMR spectrum of 3-methylfuran-2-yl-dimethylcarbamate (1f)

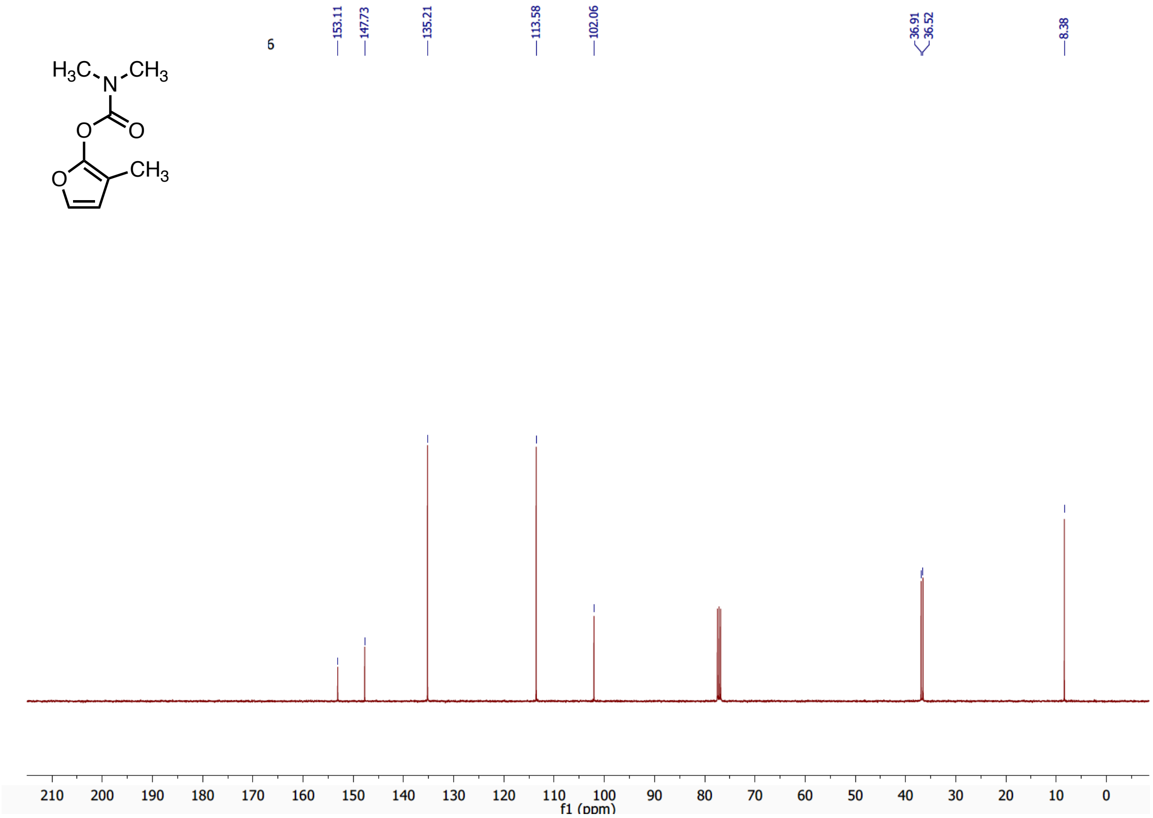

^{1}H NMR spectrum of 3-phenylfuran-2-yl-dimethylcarbamate (1g)

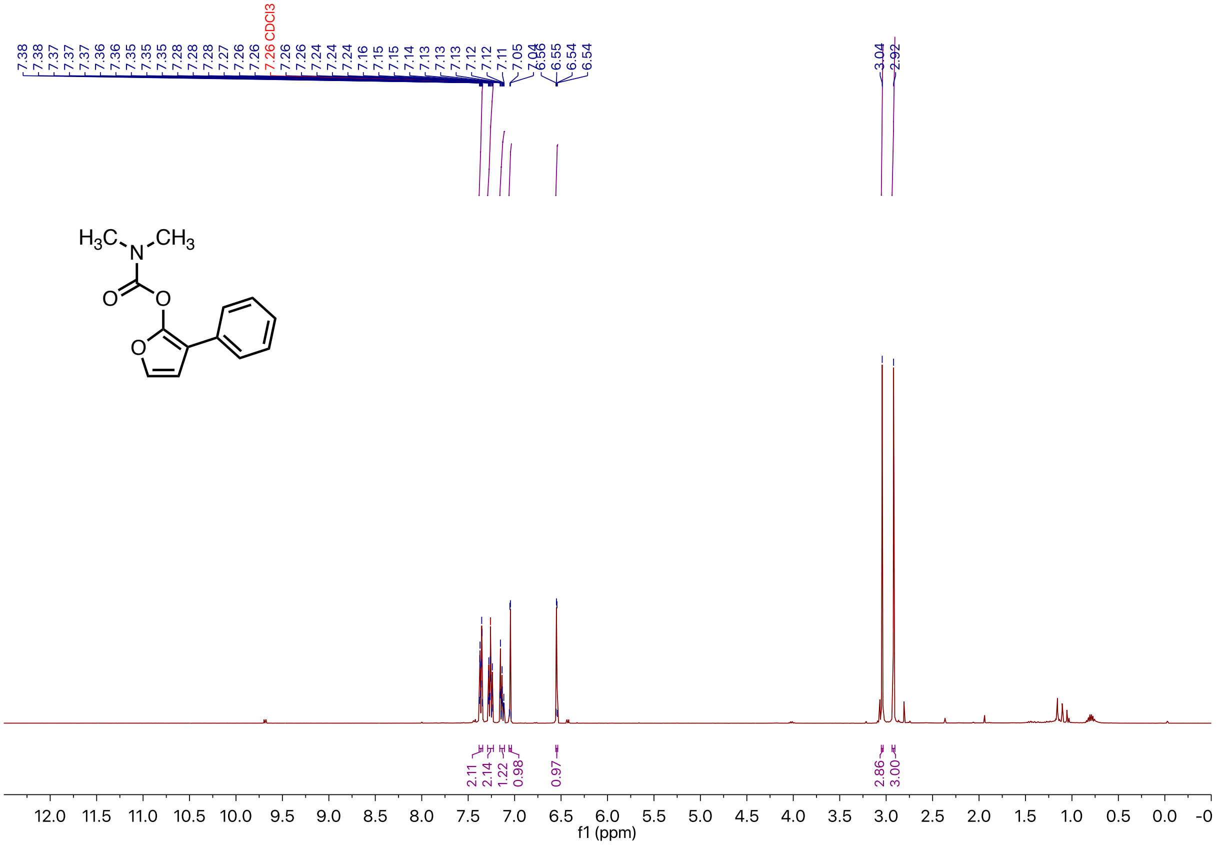

^{13}C NMR spectrum of 3-phenylfuran-2-yl-dimethylcarbamate (1g)

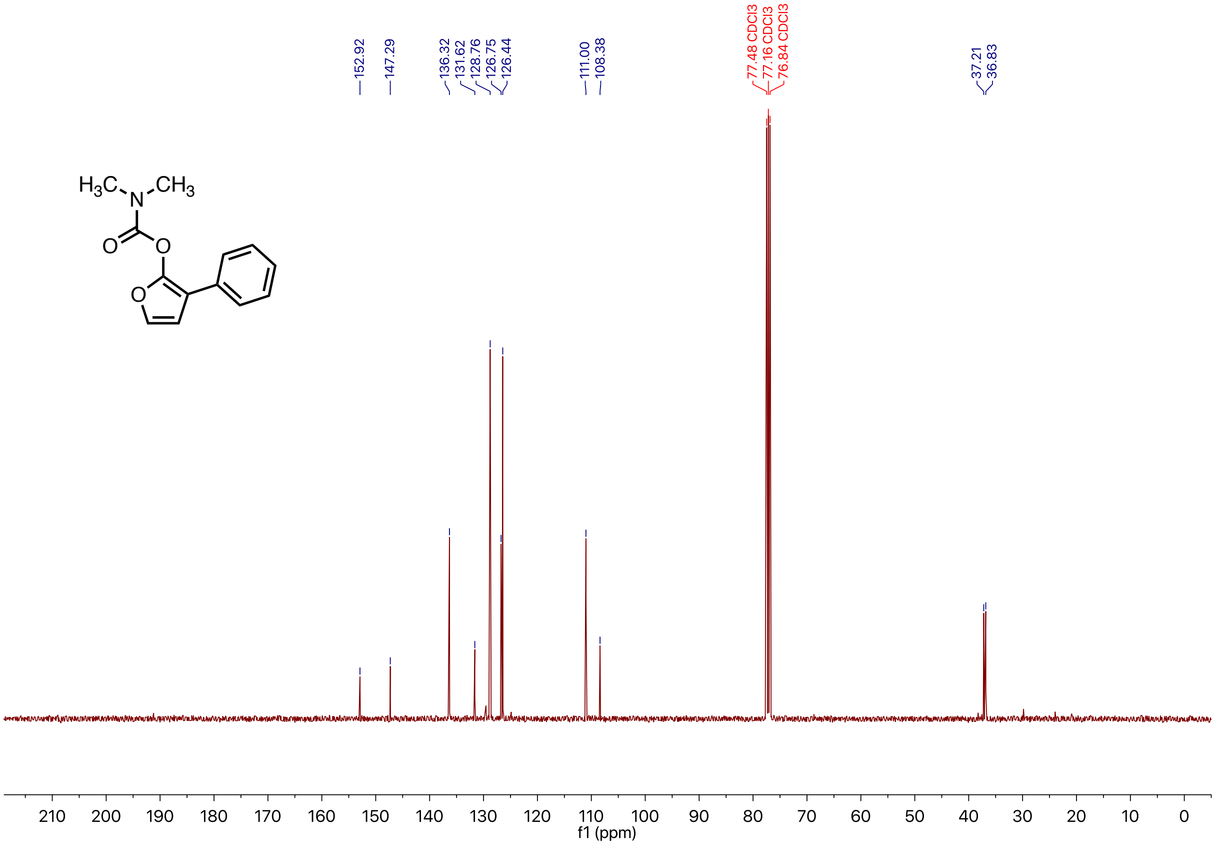

^{1}H NMR spectrum of 3-(4-ethylphenyl)furan-2-yl-dimethylcarbamate (1h)

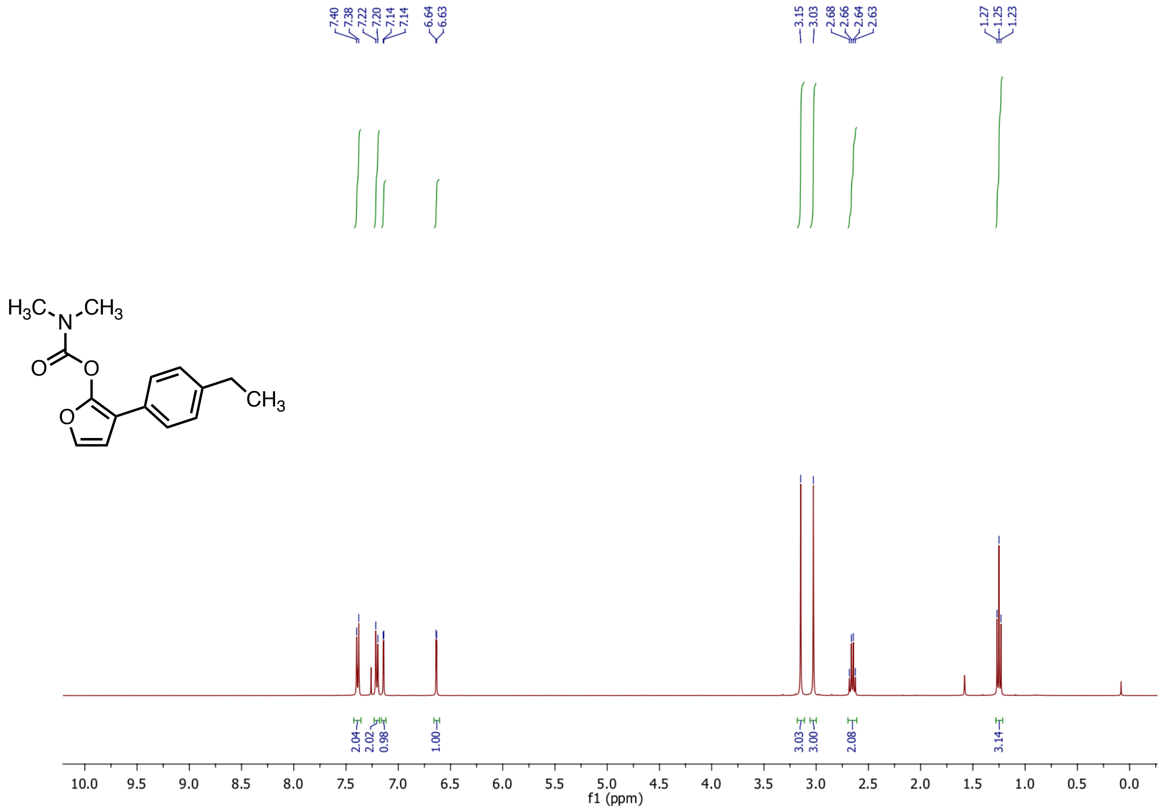

^{13}C NMR spectrum of 3-(4-ethylphenyl)furan-2-yl-dimethylcarbamate (1h)

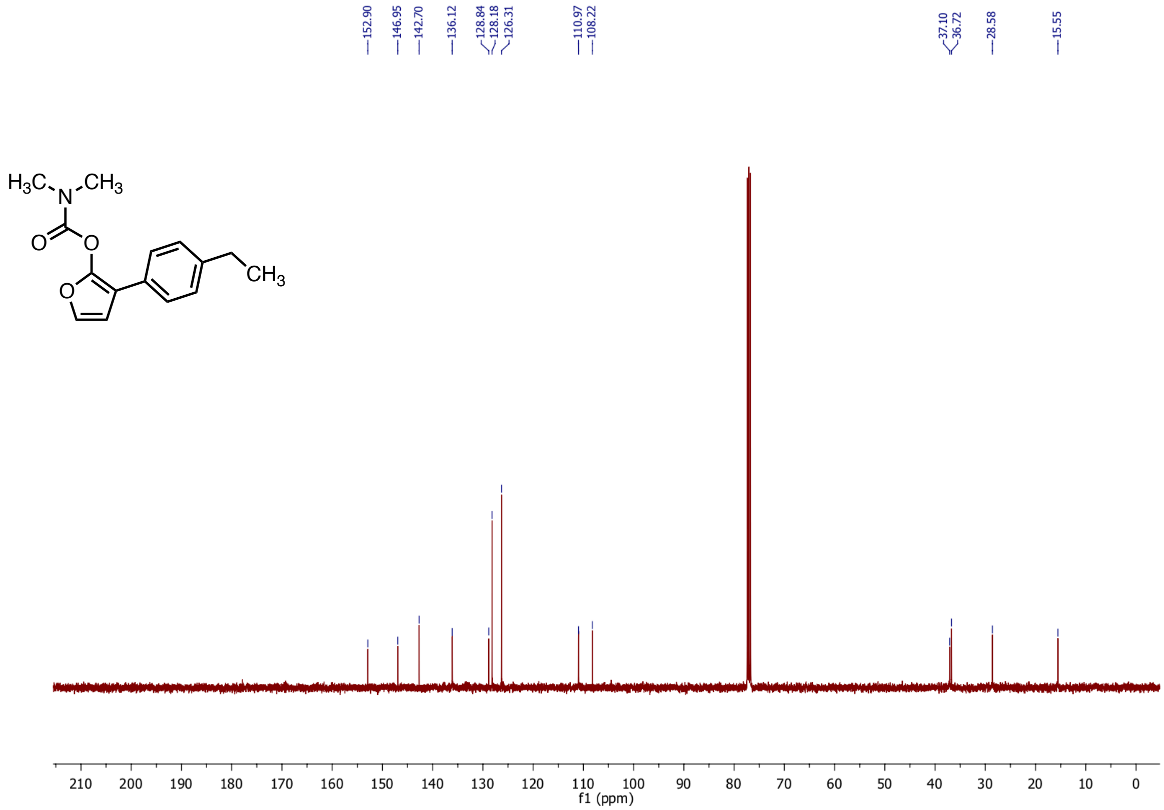

^{1}H NMR spectrum of 3-(4-fluorophenyl)furan-2-yl-dimethylcarbamate (1i)

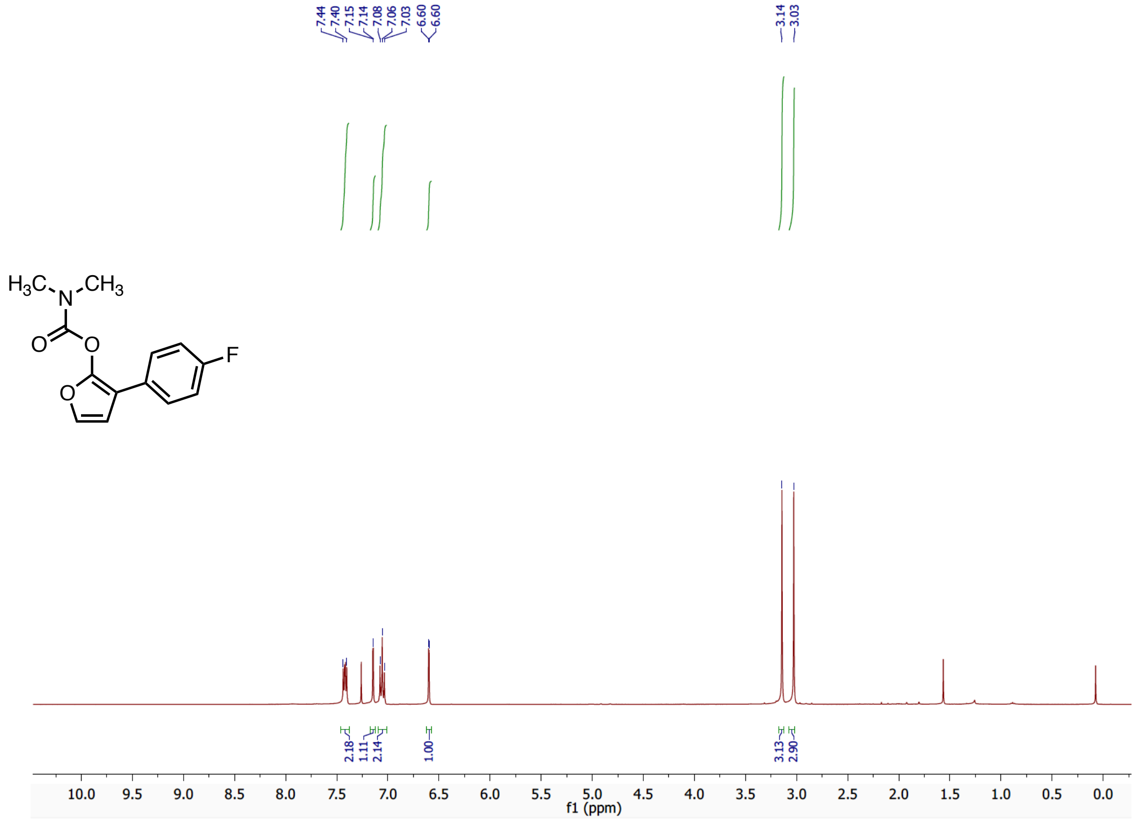

^{13}C NMR spectrum of 3-(4-fluorophenyl)furan-2-yl-dimethylcarbamate (1i)

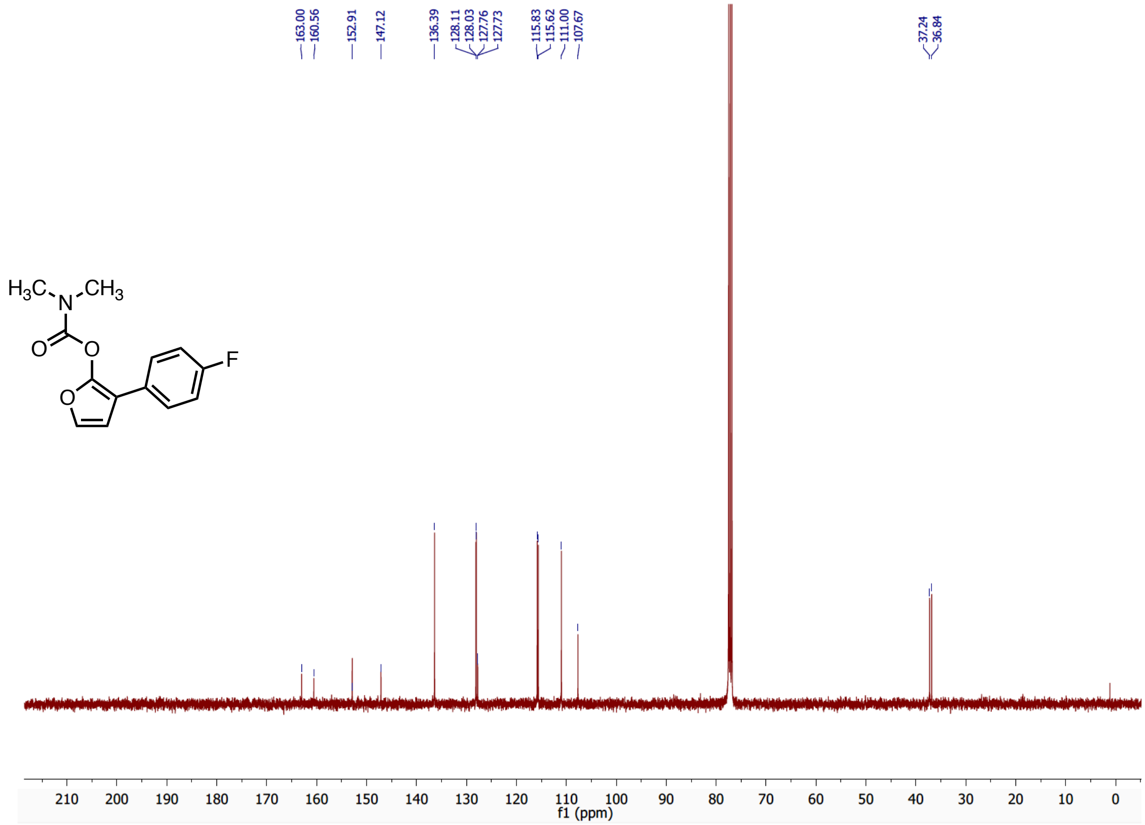

^{1}H NMR spectrum of 5-cyclohexylfuran-2-yl-dimethylcarbamate (1j)

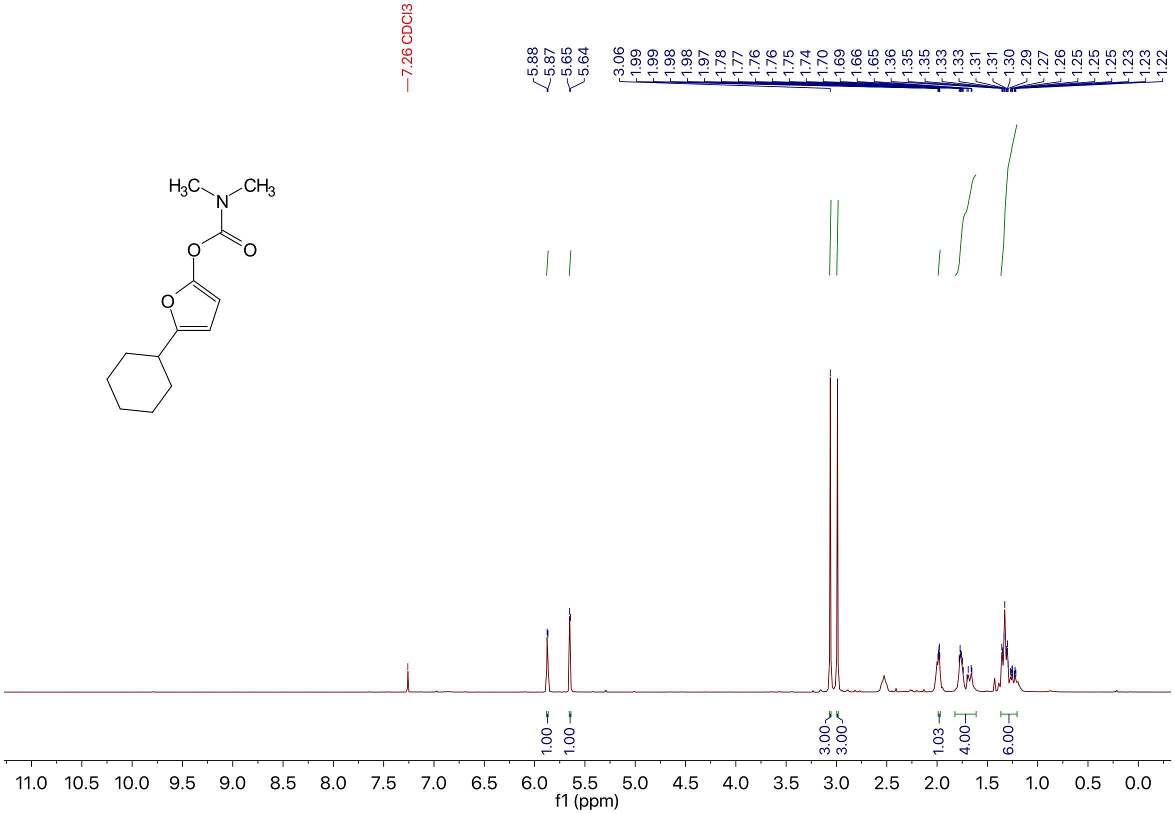

^{13}C NMR spectrum of 5-cyclohexylfuran-2-yl-dimethylcarbamate (1j)

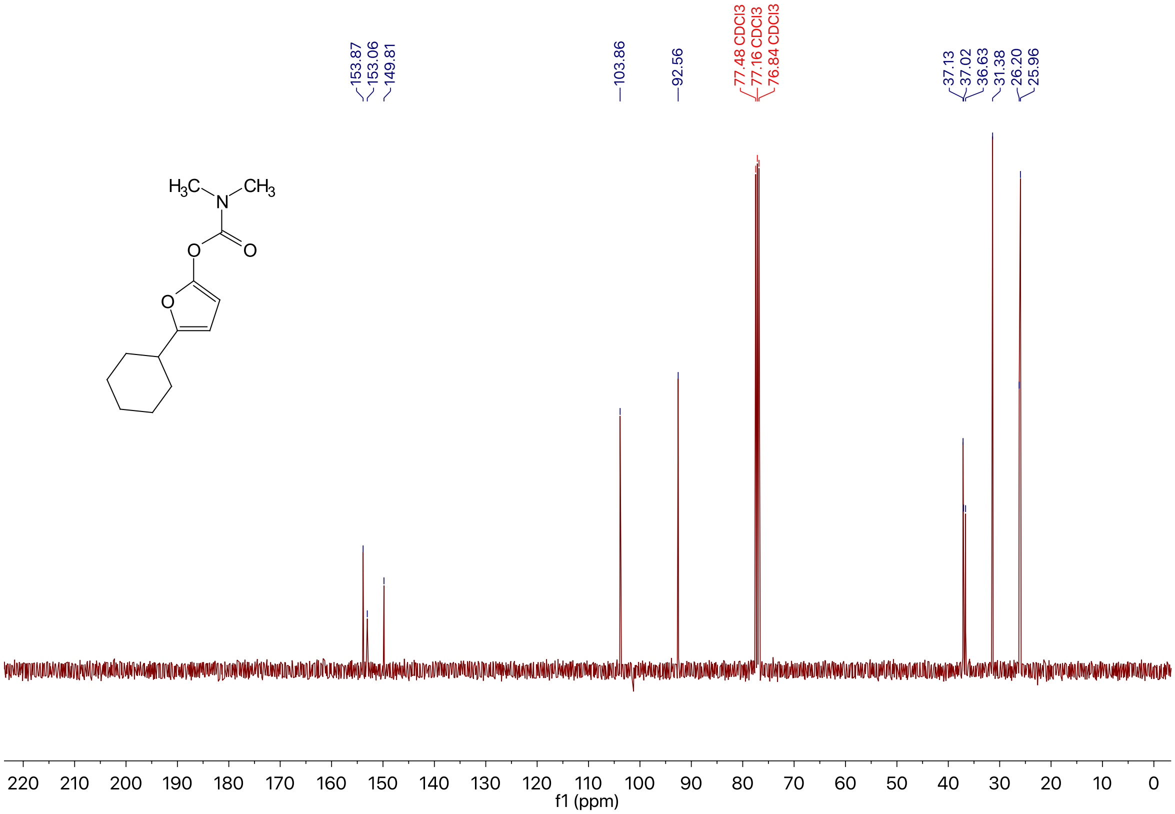

^{1}H NMR spectrum of 5-cyclopropylfuran-2-yl-dimethylcarbamate (1k)

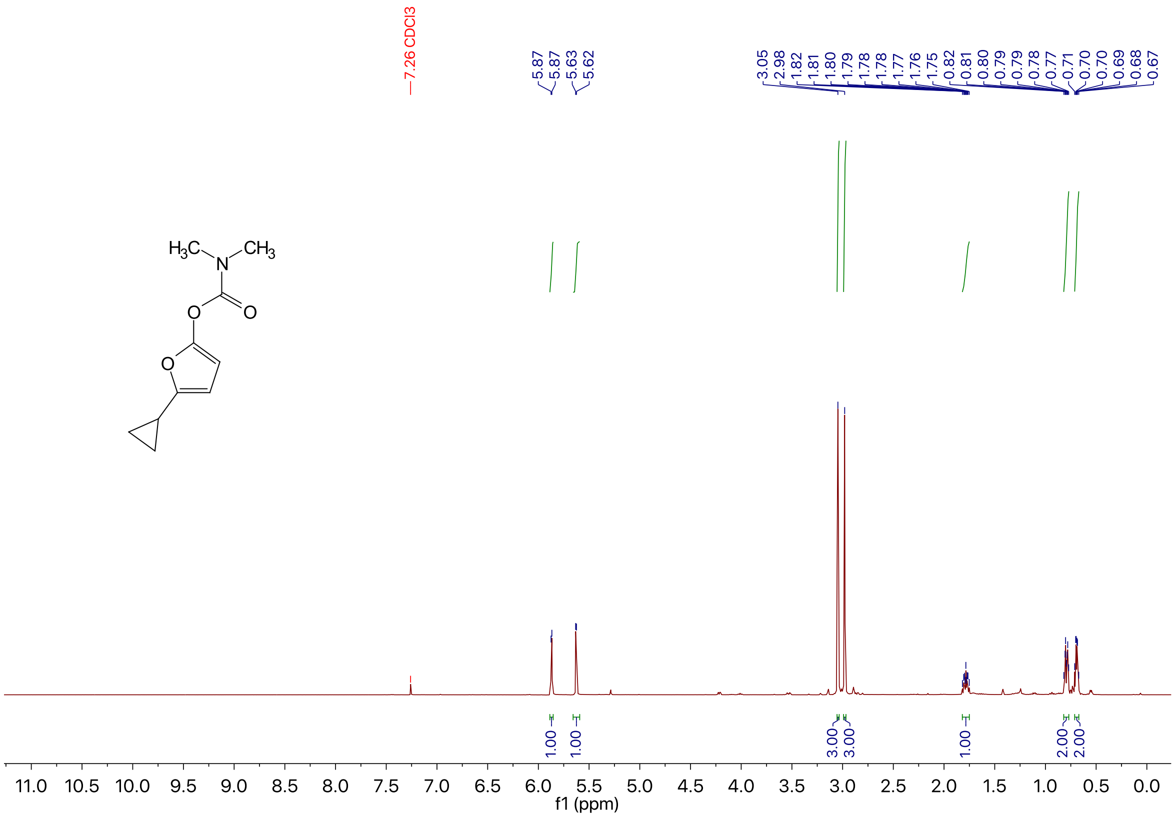

^{13}C NMR spectrum of 5-cyclopropylfuran-2-yl-dimethylcarbamate (1k)

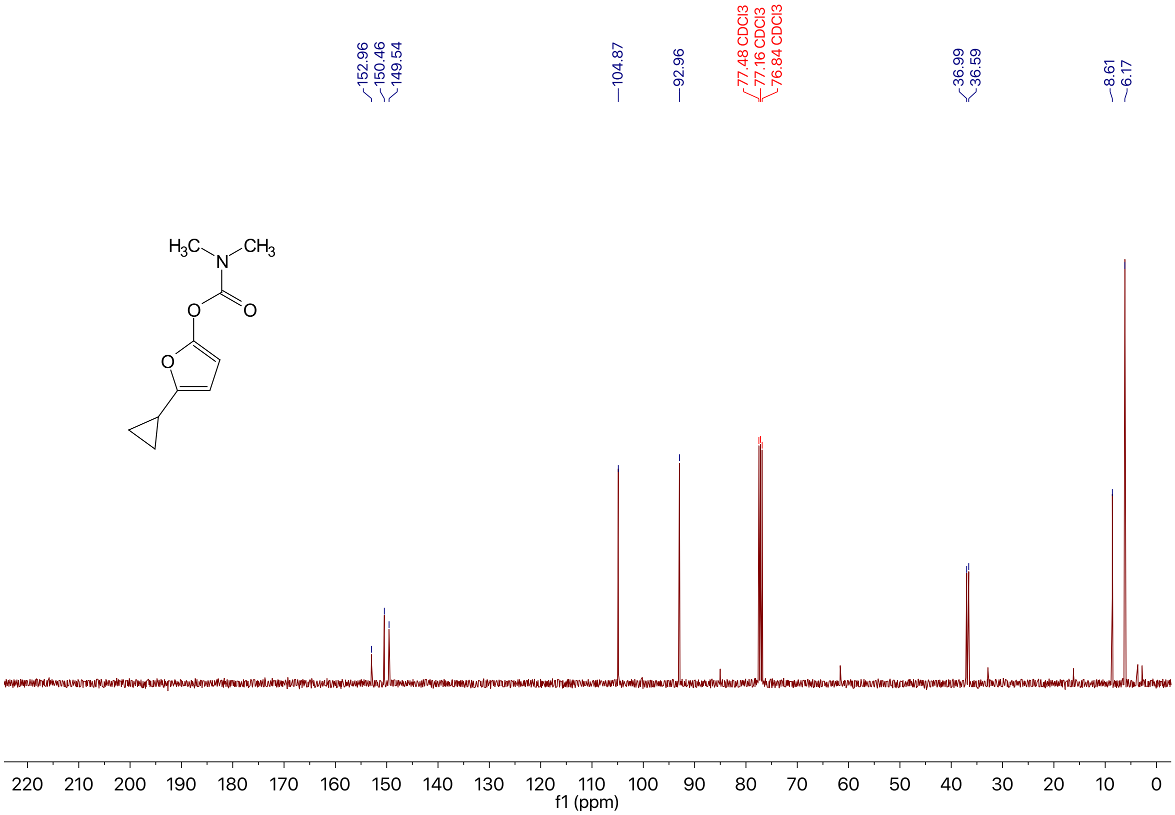

^{1}H NMR spectrum of 5-phenylfuran-2-yl-dimethylcarbamate (1l)

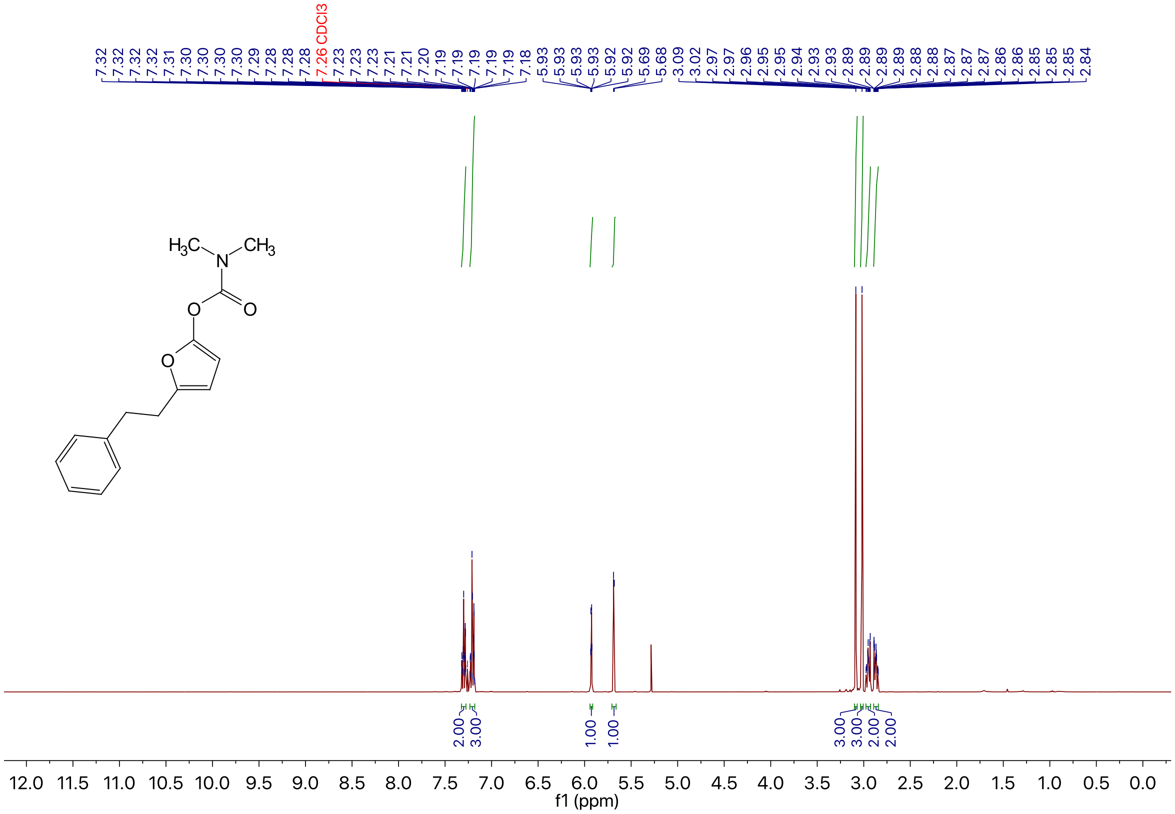

^{13}C NMR spectrum of 5-phenylfuran-2-yl-dimethylcarbamate (1l)

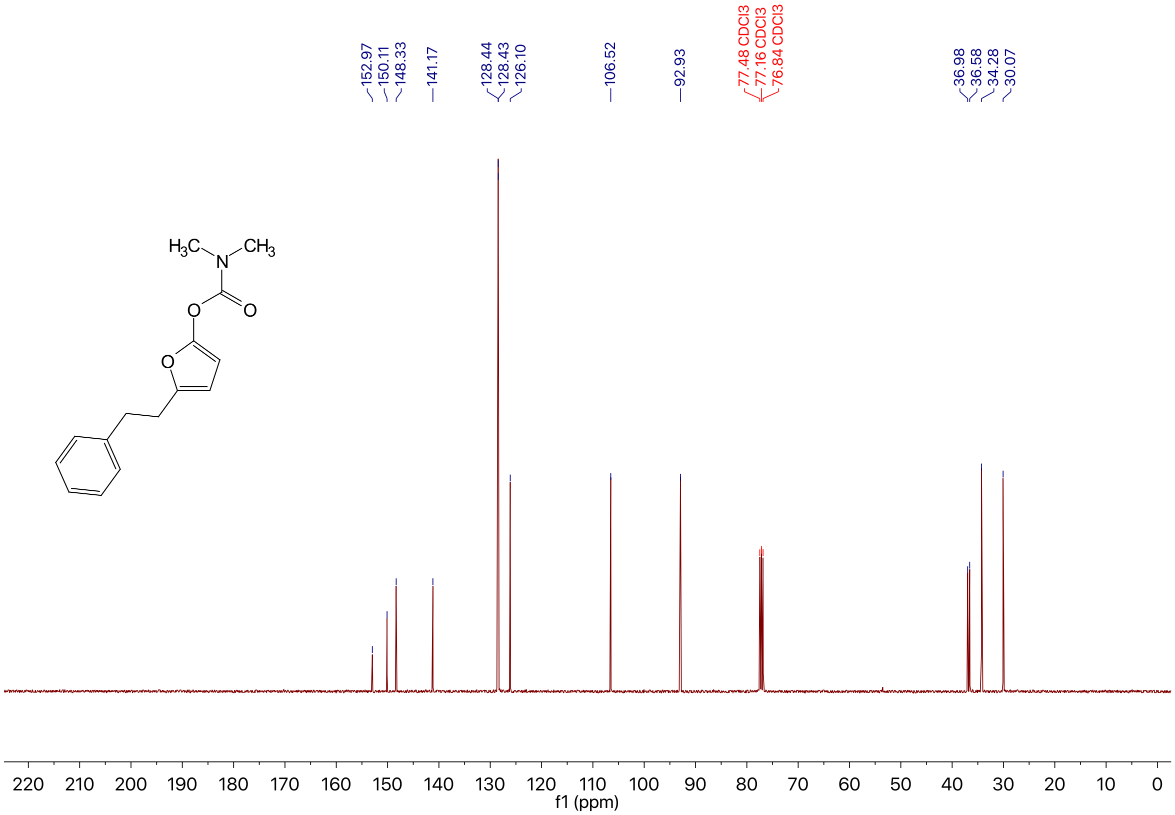

^{1}H NMR spectrum of 4-methylfuran-2-yl-dimethylcarbamate (1m)

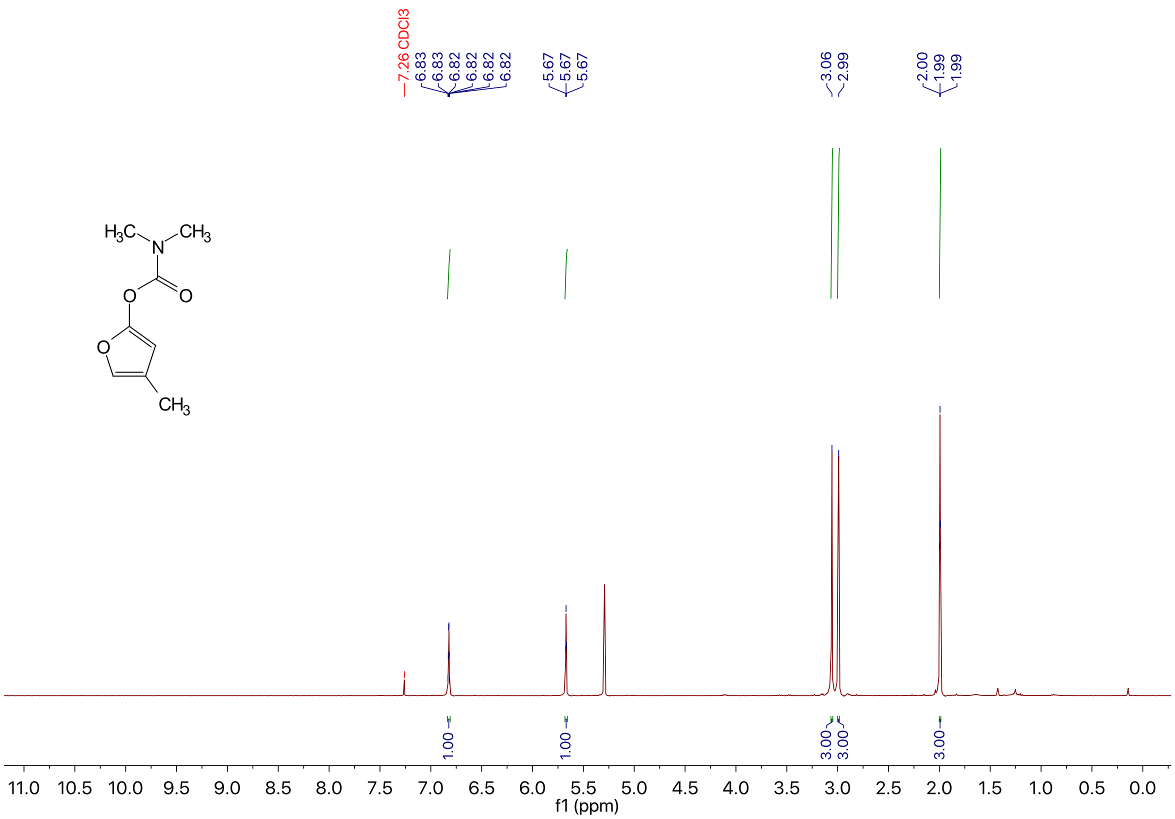

^{13}C NMR spectrum of 4-methylfuran-2-yl-dimethylcarbamate (1m)

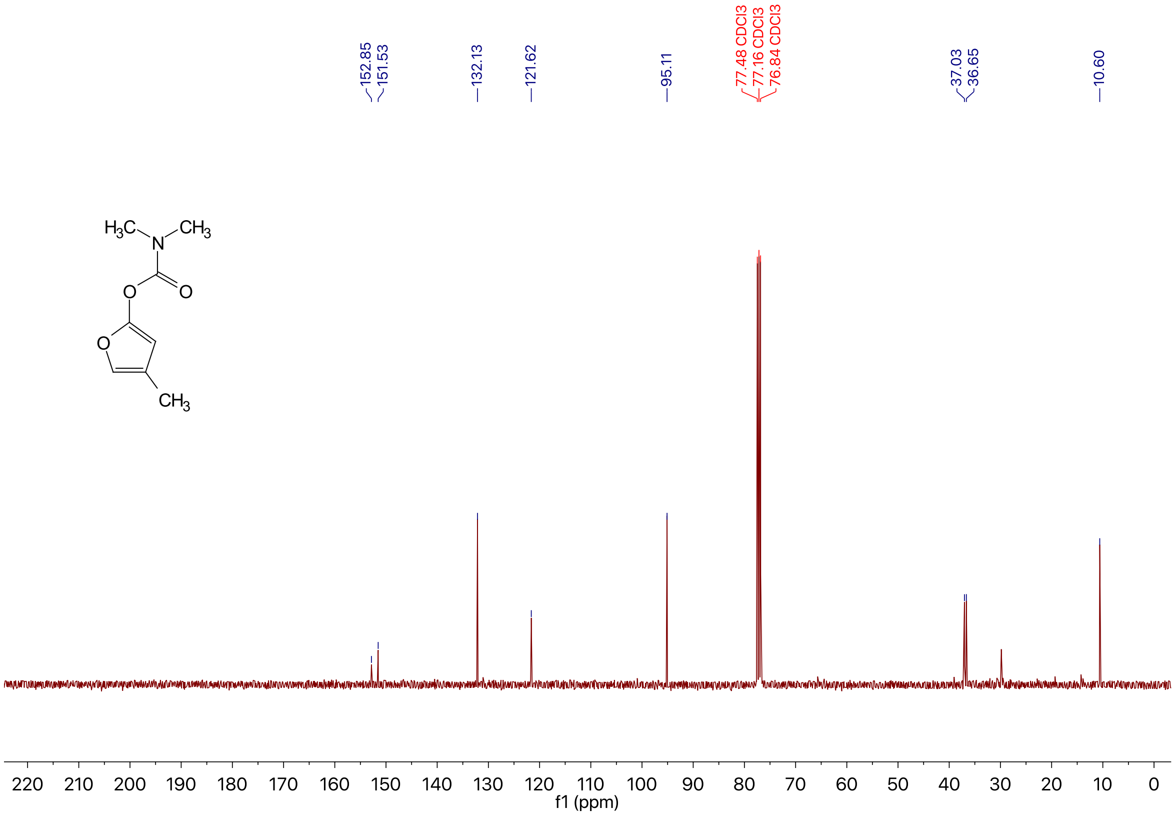

^{1}H NMR spectrum of 5-(*o*-tolyl)furan-2-yl-dimethylcarbamate (1n)

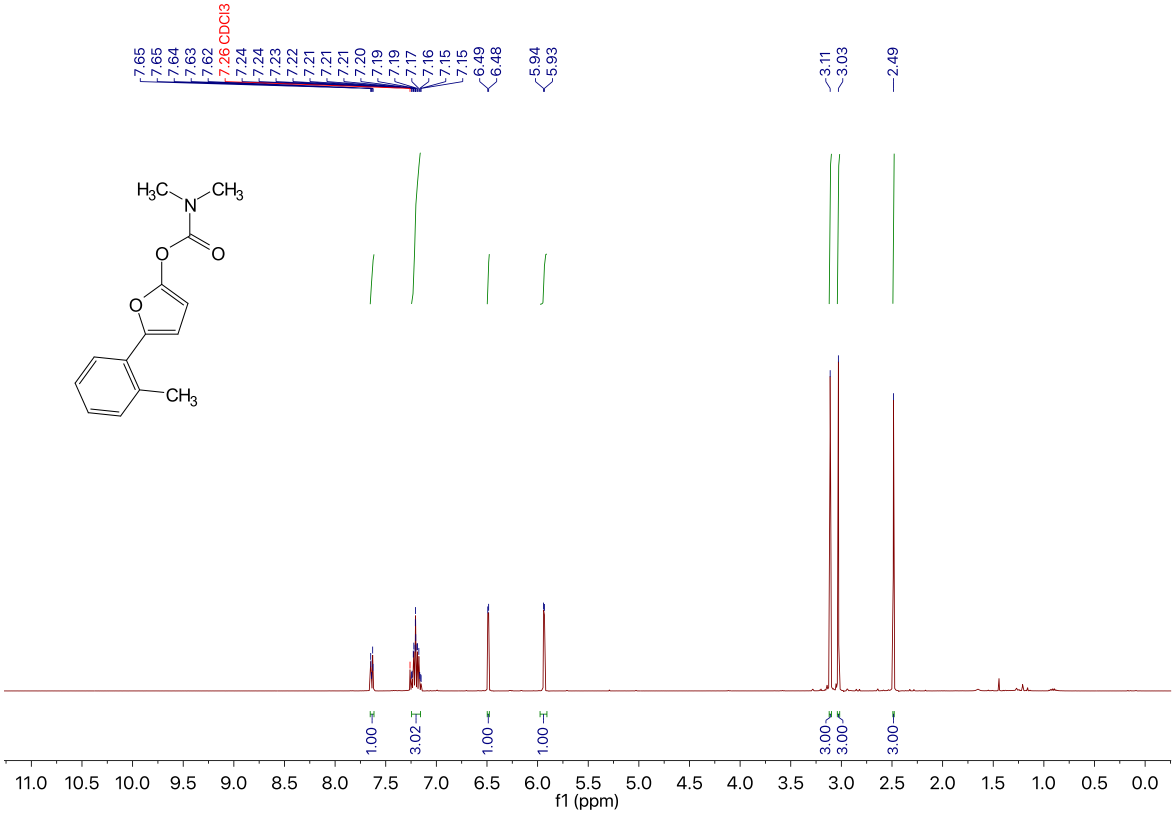

^{13}C NMR spectrum of 5-(*o*-tolyl)furan-2-yl-dimethylcarbamate (1n)

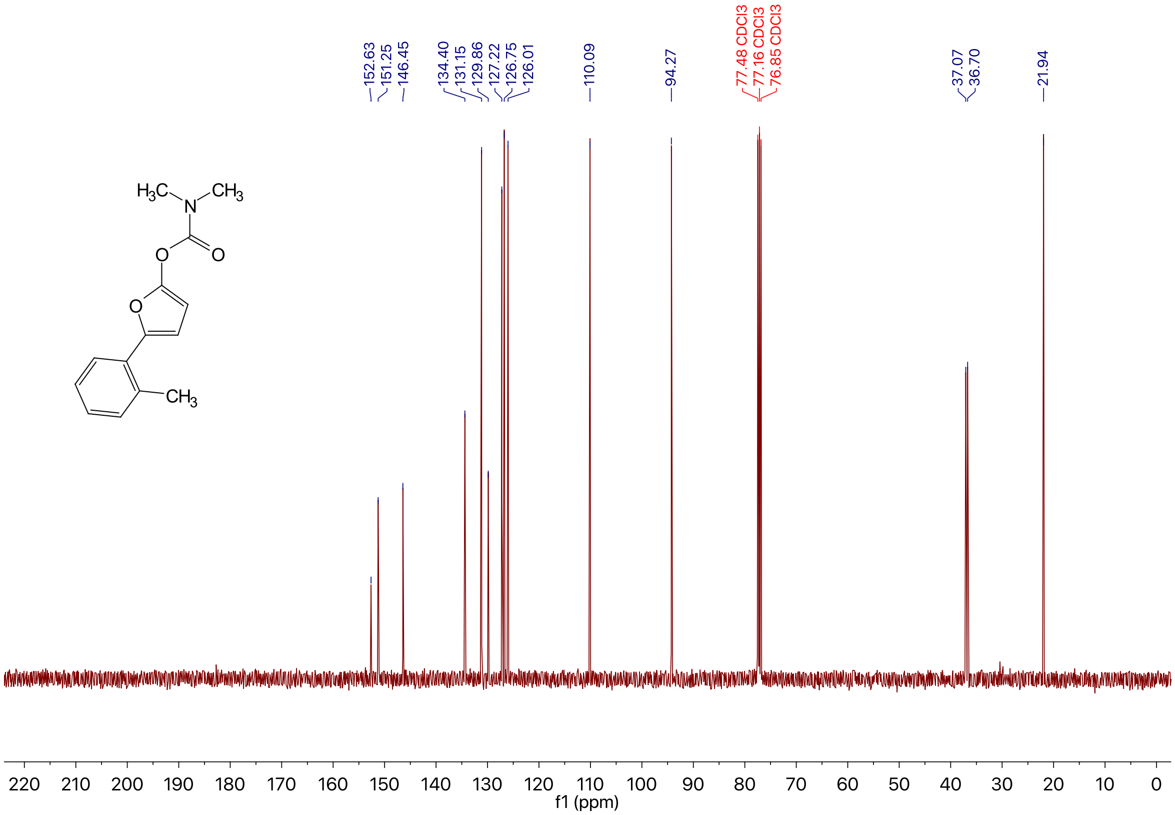

^{1}H NMR spectrum of 5-(4-bromophenyl)furan-2-yl-dimethylcarbamate (1o)

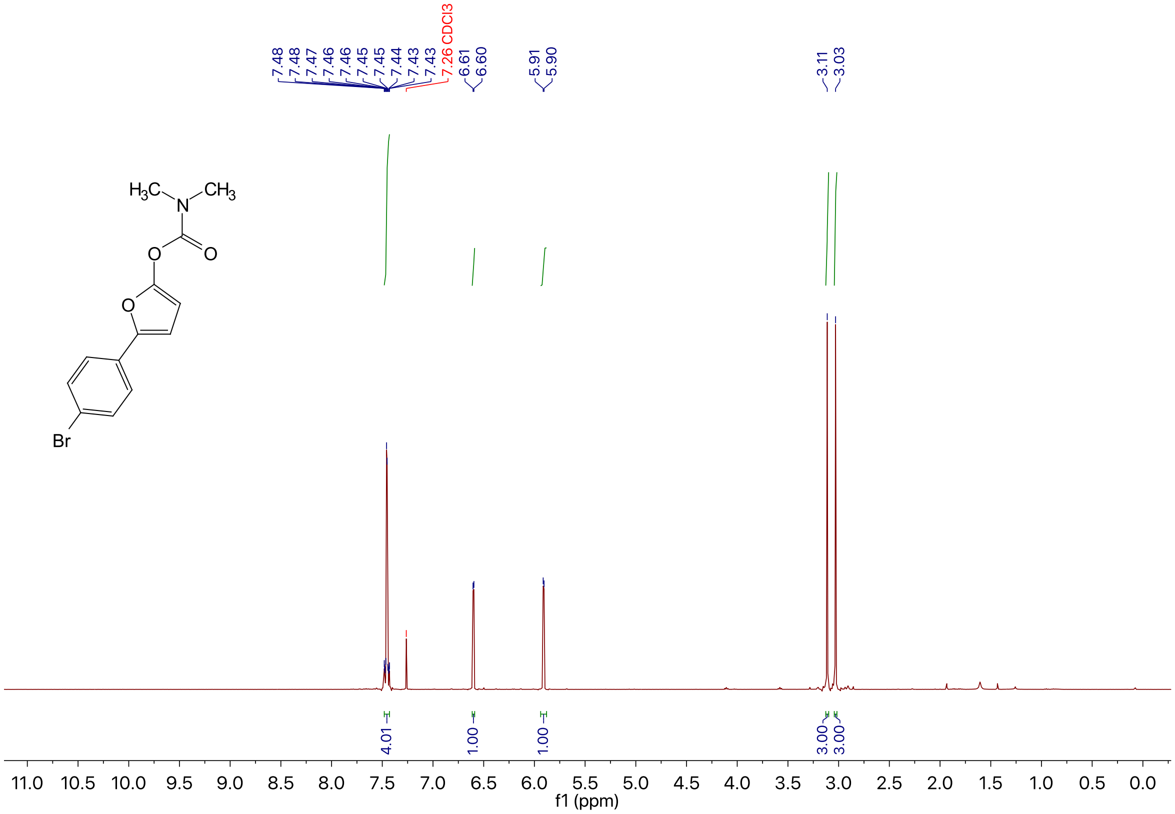

^{13}C NMR spectrum of 5-(4-bromophenyl)furan-2-yl-dimethylcarbamate (1o)

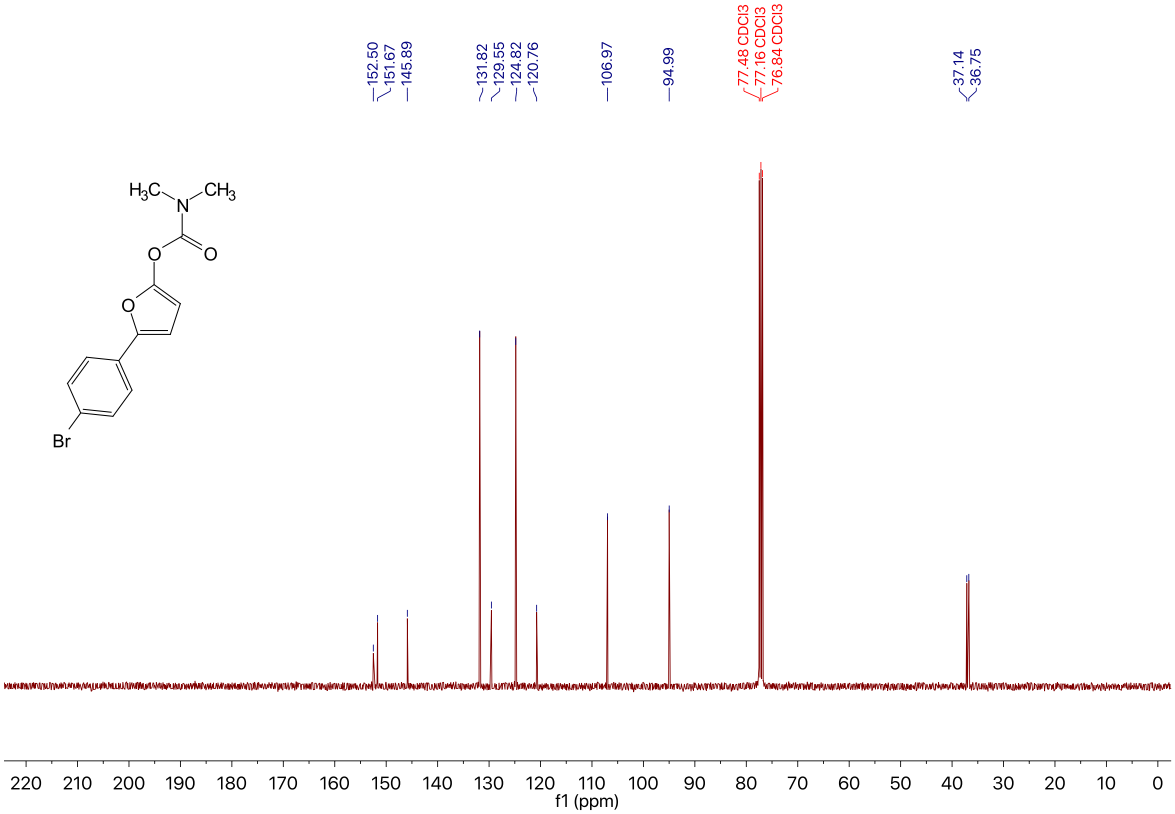

^{1}H NMR spectrum of 5-(4-nitrophenyl)furan-2-yl-dimethylcarbamate (1p)

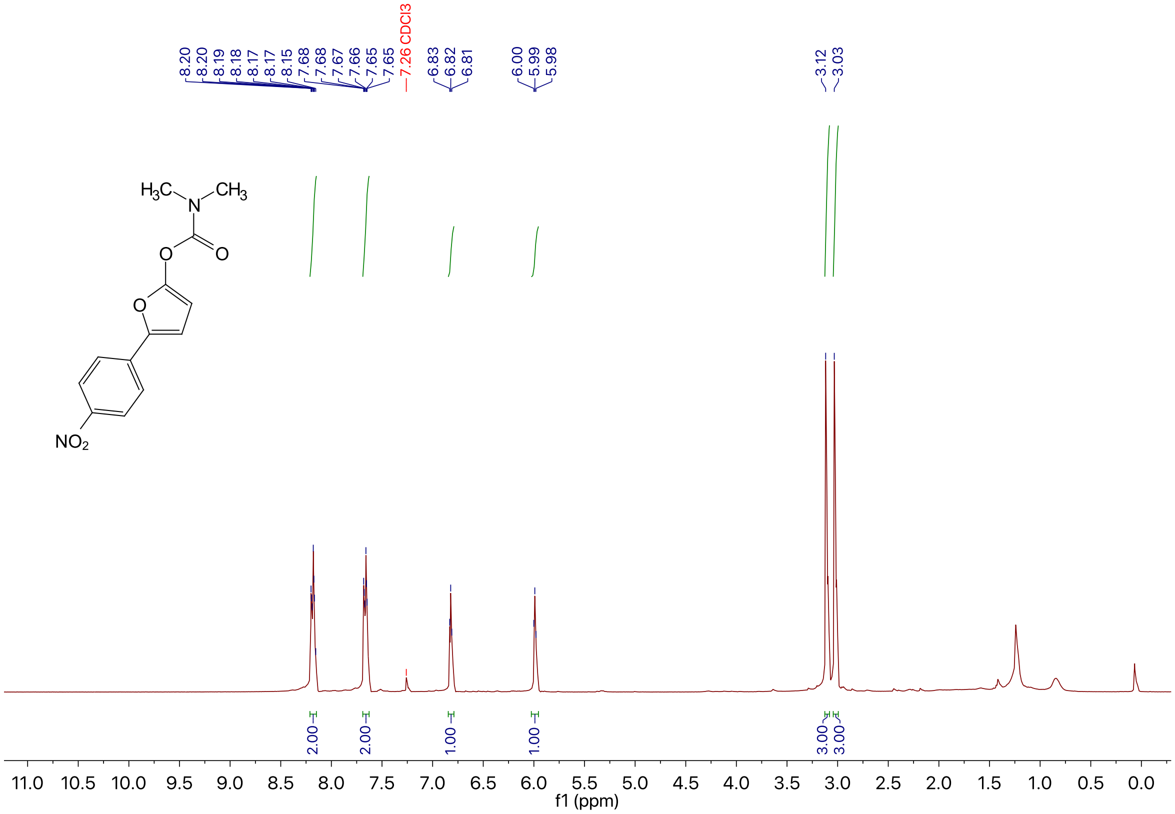

^{13}C NMR spectrum of 5-(4-nitrophenyl)furan-2-yl-dimethylcarbamate (1p)

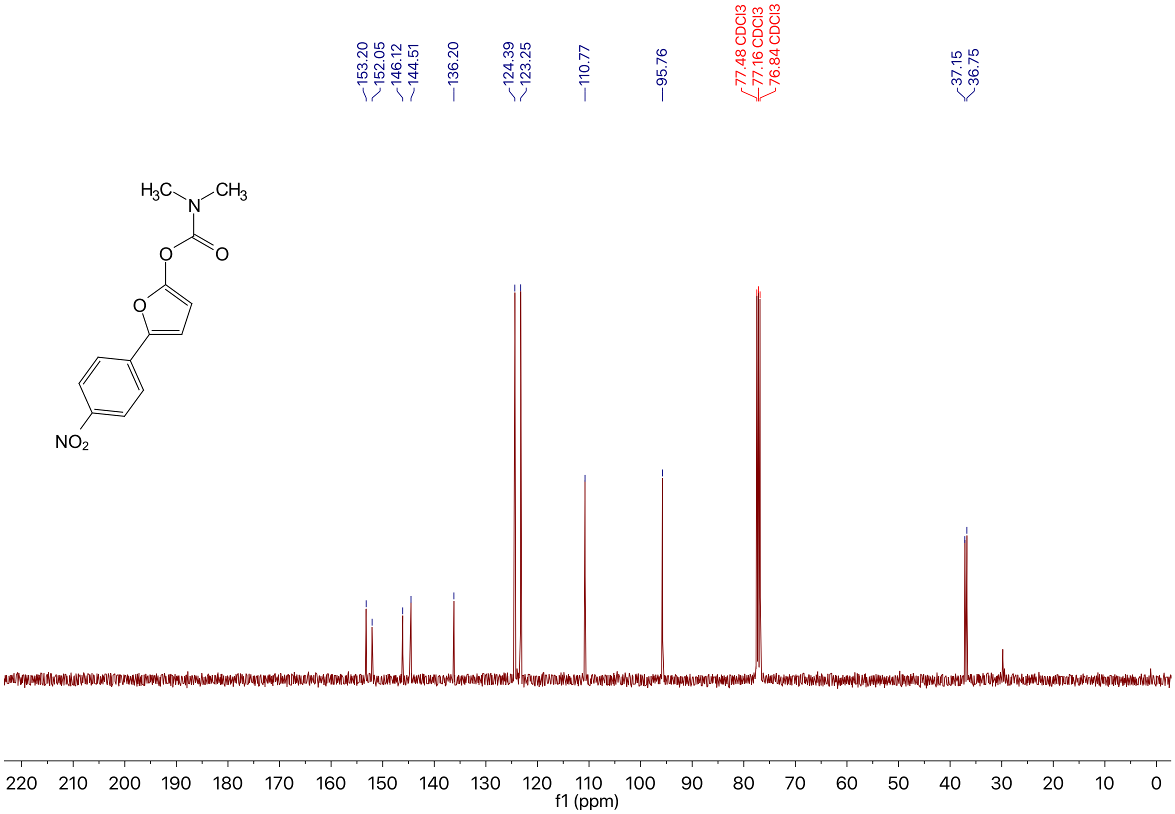

^{1}H NMR spectrum of methyl 4-(5-((dimethylcarbamoyl)oxy)furan-2-yl)benzoate (1q)

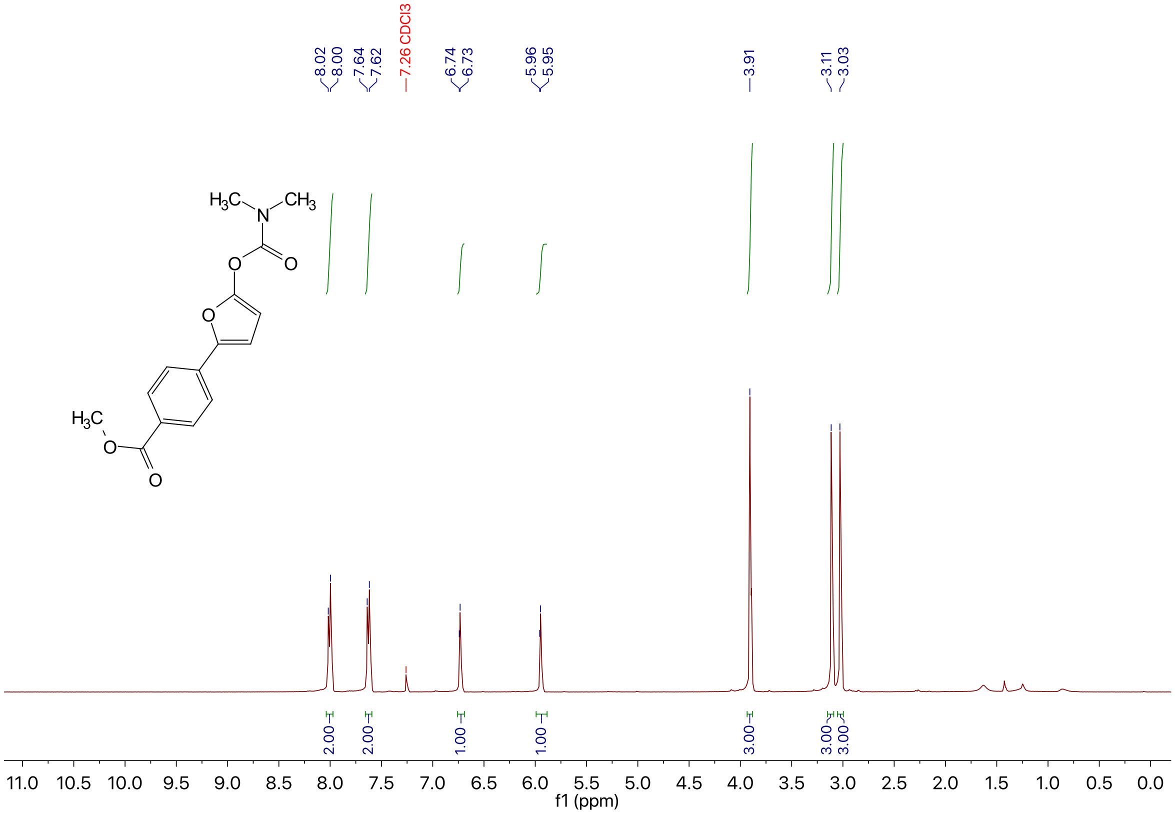

^{13}C NMR spectrum of methyl 4-(5-((dimethylcarbamoyl)oxy)furan-2-yl)benzoate (1q)

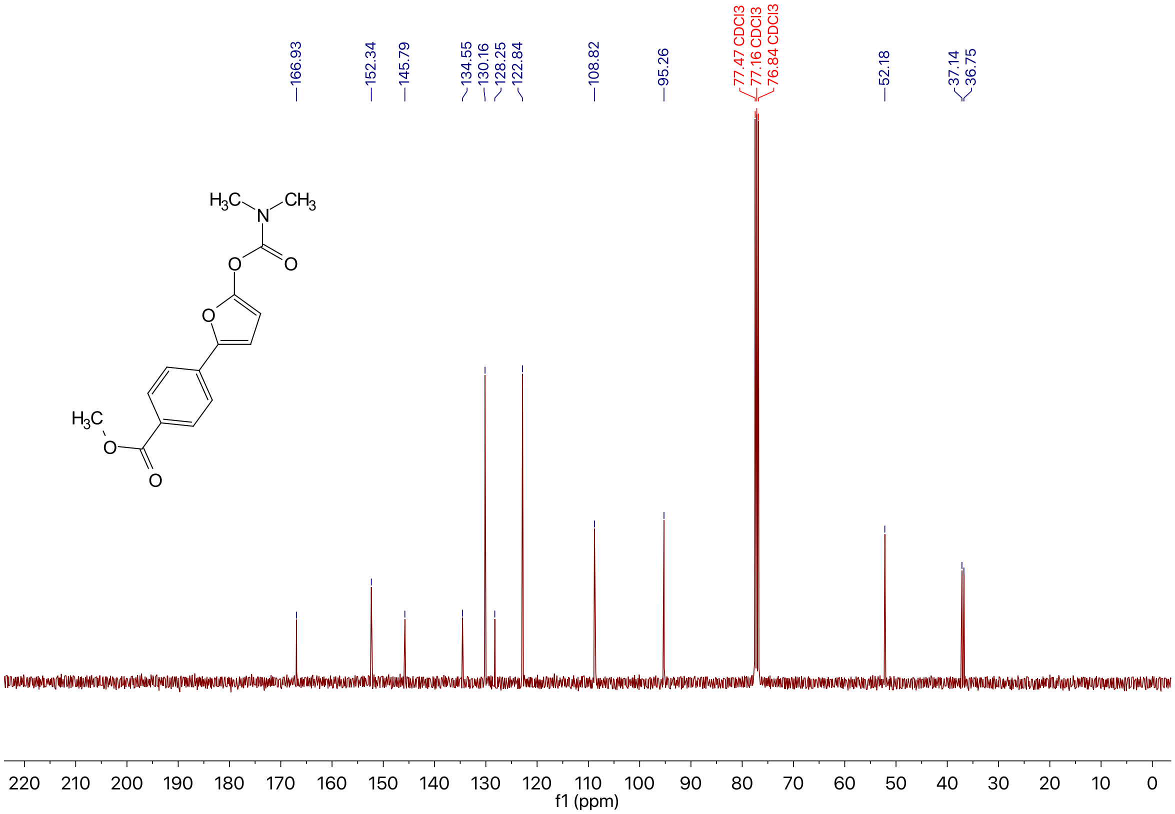

^{1}H NMR spectrum of 5-5-(thiophen-2-yl)furan-2-yl-dimethylcarbamate (1r)

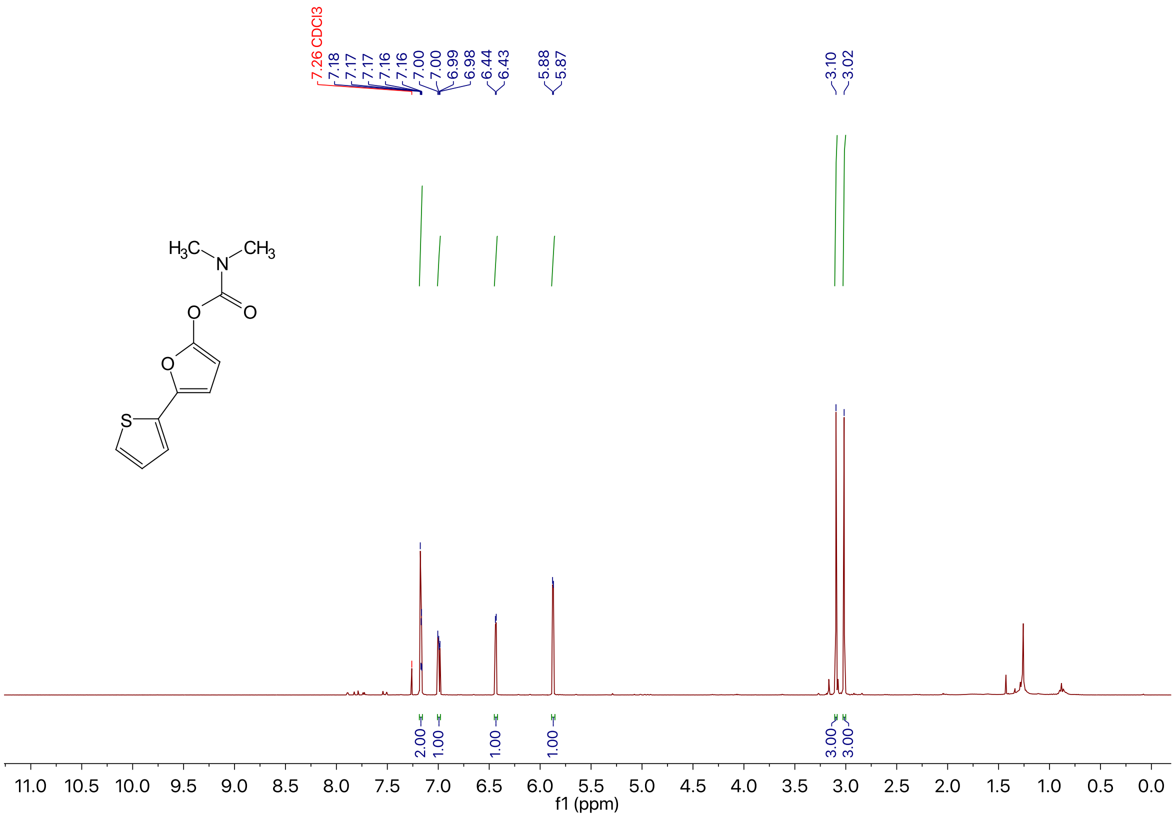

^{13}C NMR spectrum of 5-5-(thiophen-2-yl)furan-2-yl-dimethylcarbamate (1r)

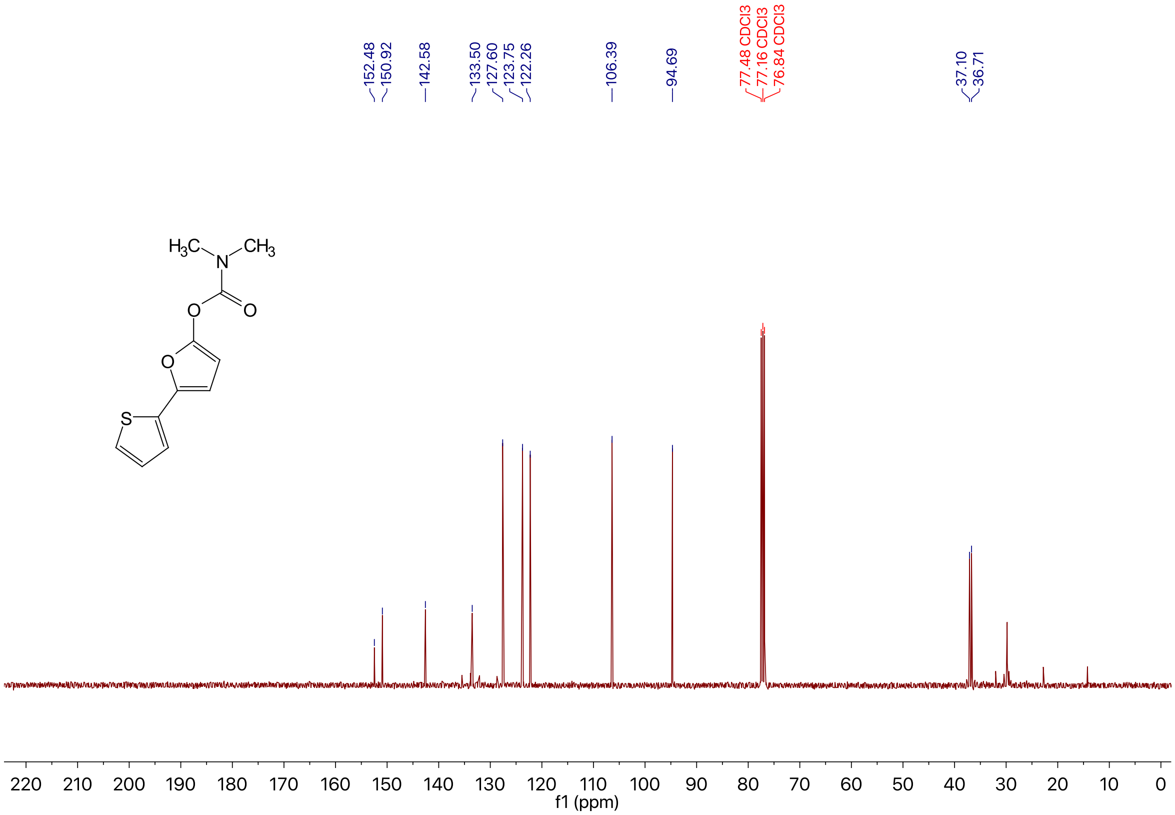

^{1}H NMR spectrum of 5-(oct-1-yn-1-yl)furan-2-yl-dimethylcarbamate (1s)

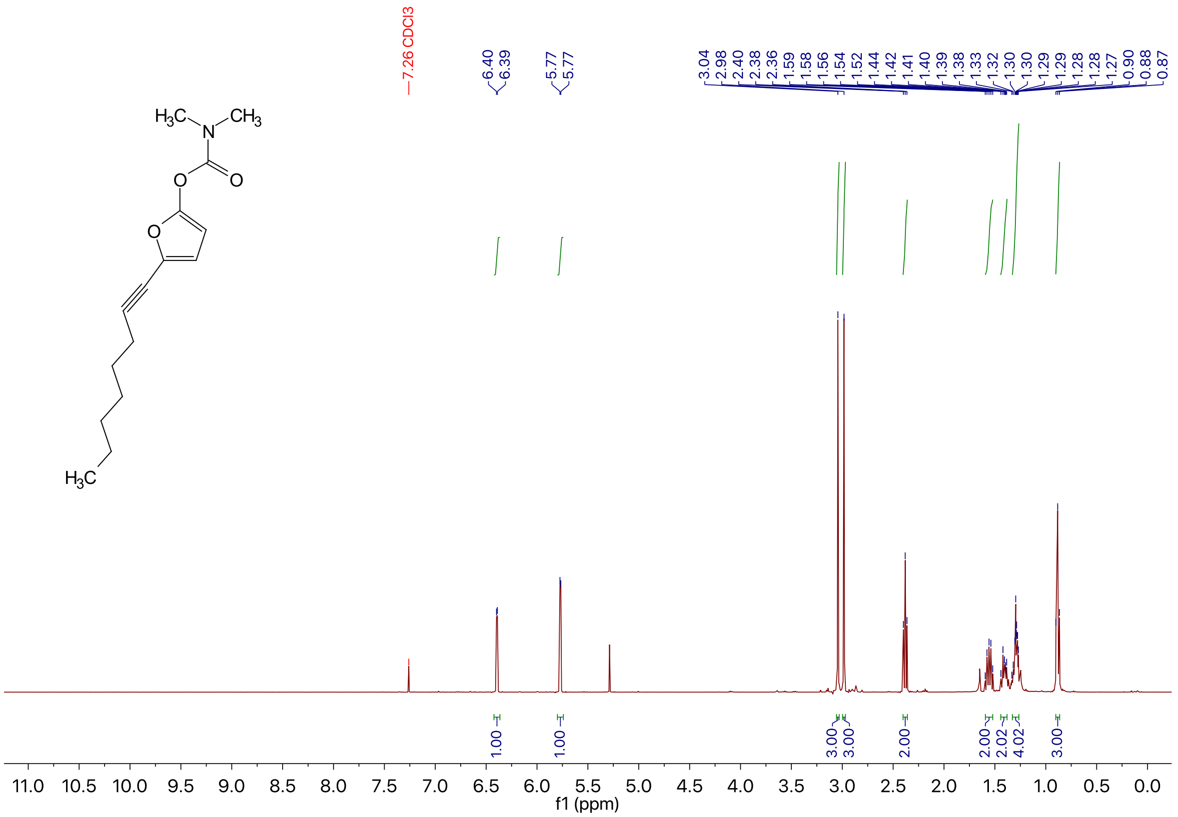

^{13}C NMR spectrum of 5-(oct-1-yn-1-yl)furan-2-yl-dimethylcarbamate (1s)

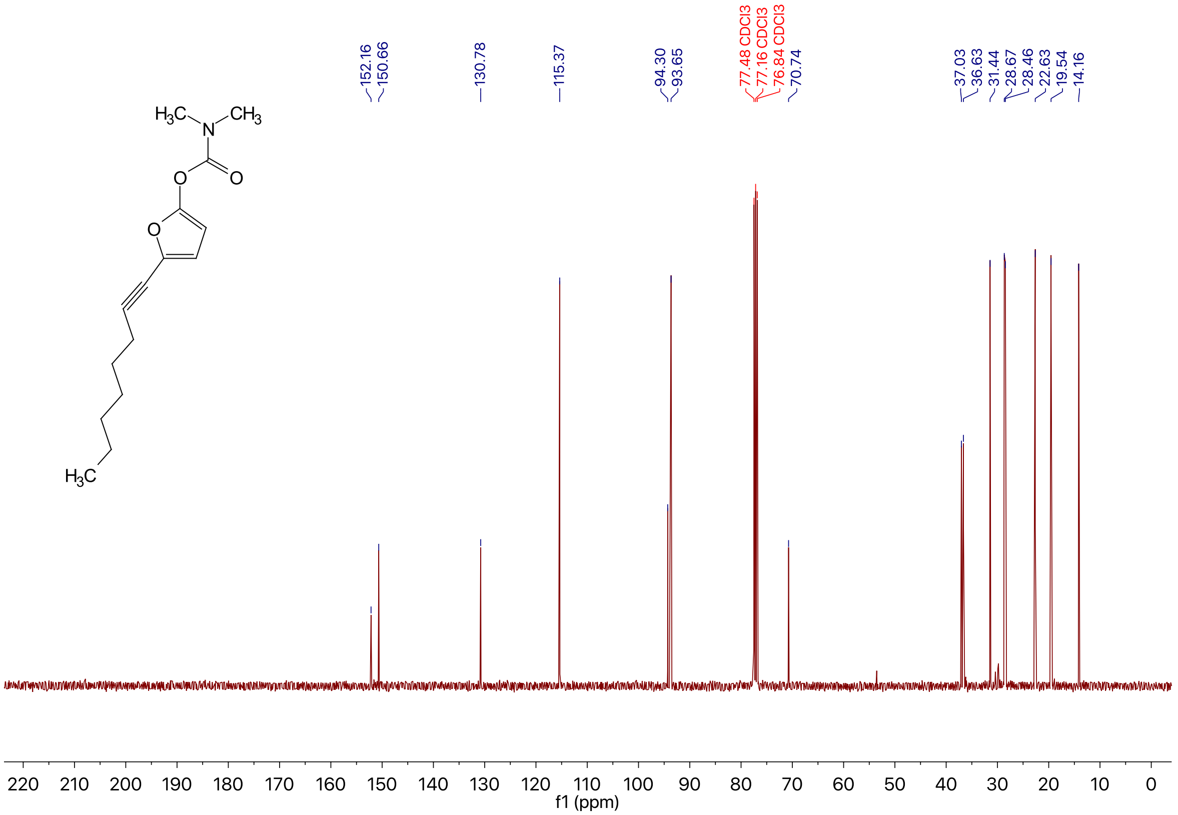

^{1}H NMR spectrum of thiophen-2-yl-dimethylcarbamate (1t)

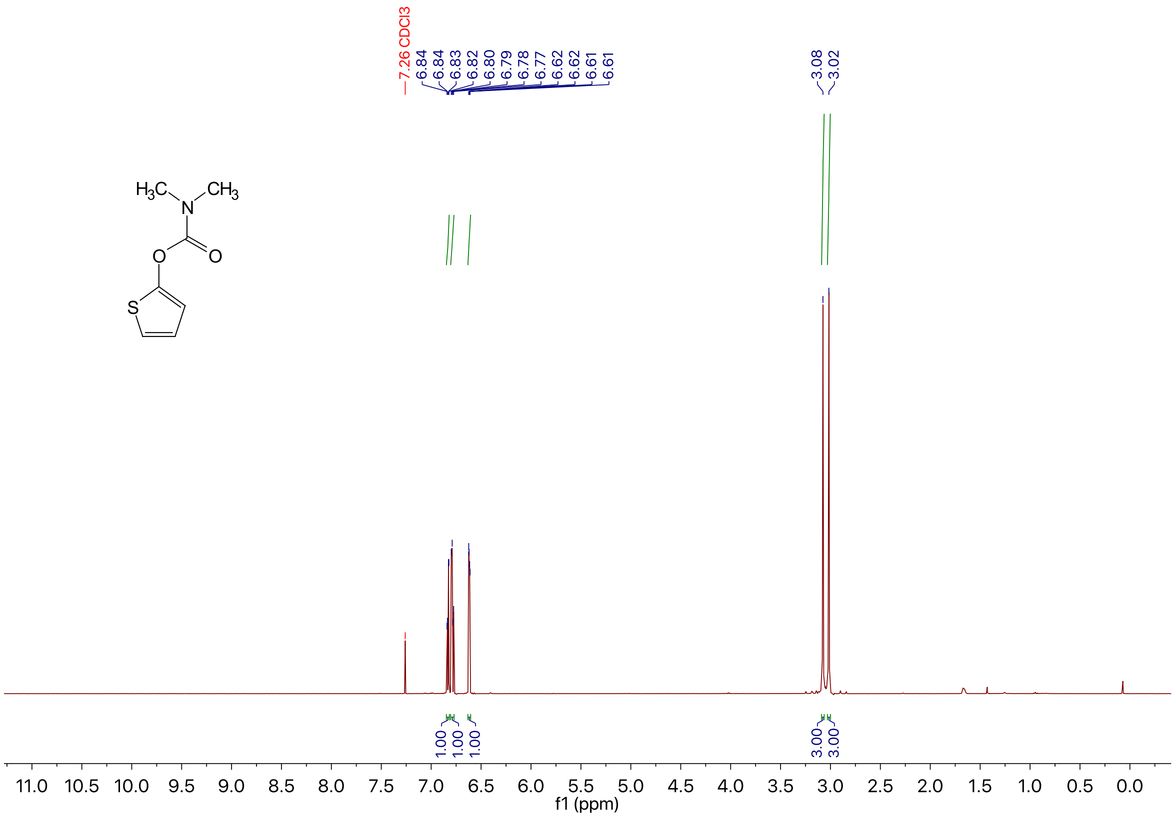

^{13}C NMR spectrum of thiophen-2-yl-dimethylcarbamate (1t)

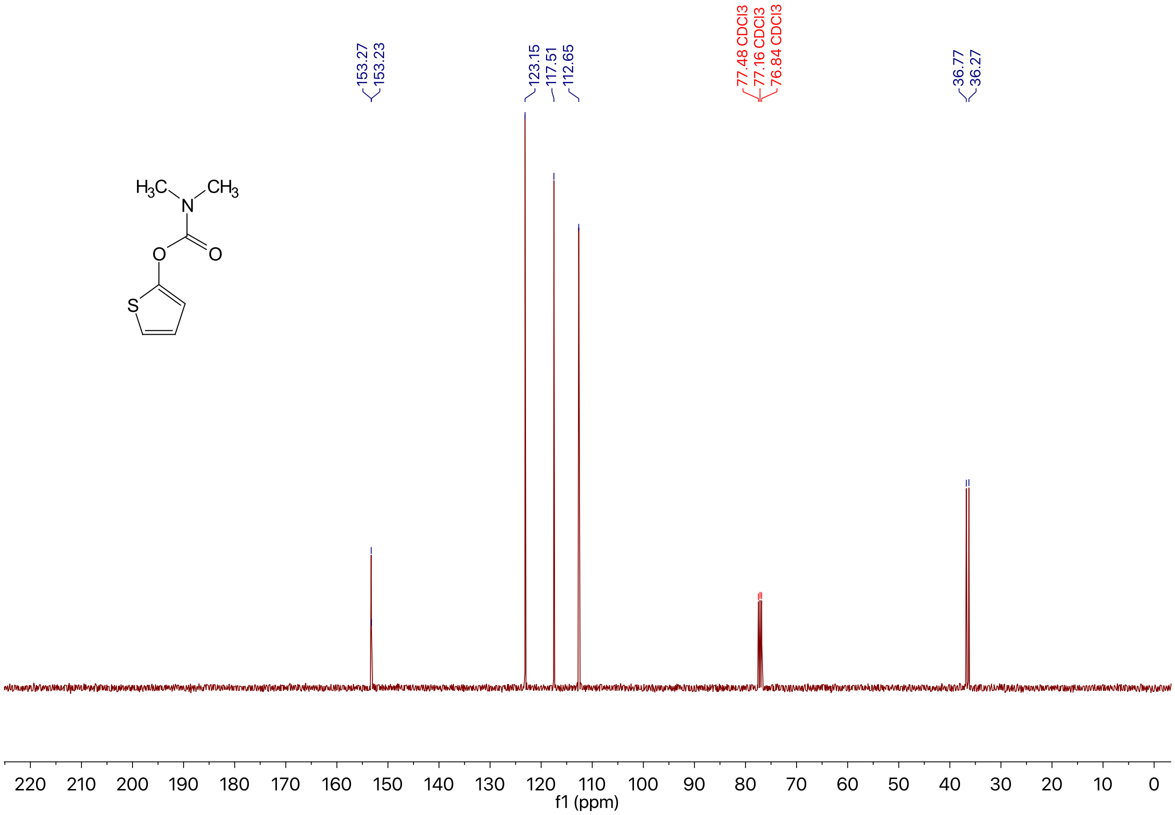

^{1}H NMR spectrum of 1-benzyl-1*H*-pyrrol-2-yl-dimethylcarbamate (1u)

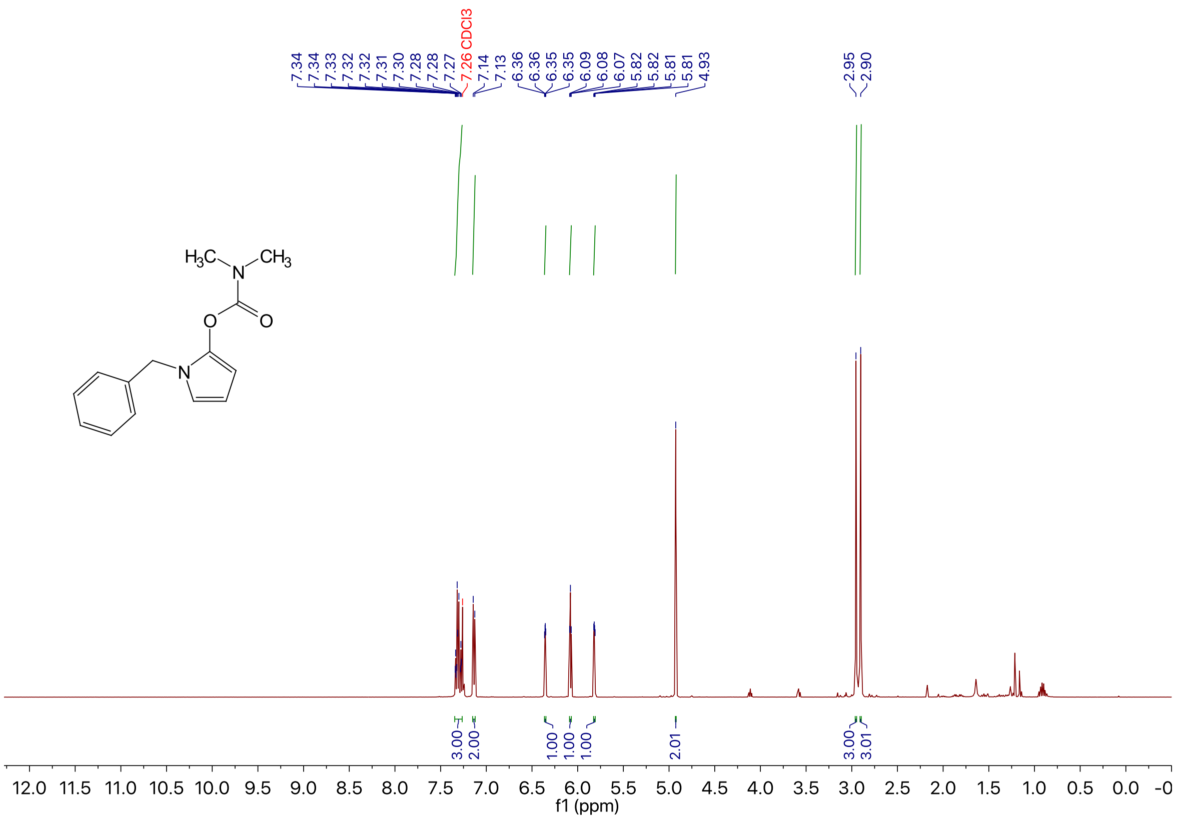

^{13}C NMR spectrum of 1-benzyl-1*H*-pyrrol-2-yl-dimethylcarbamate (1u)

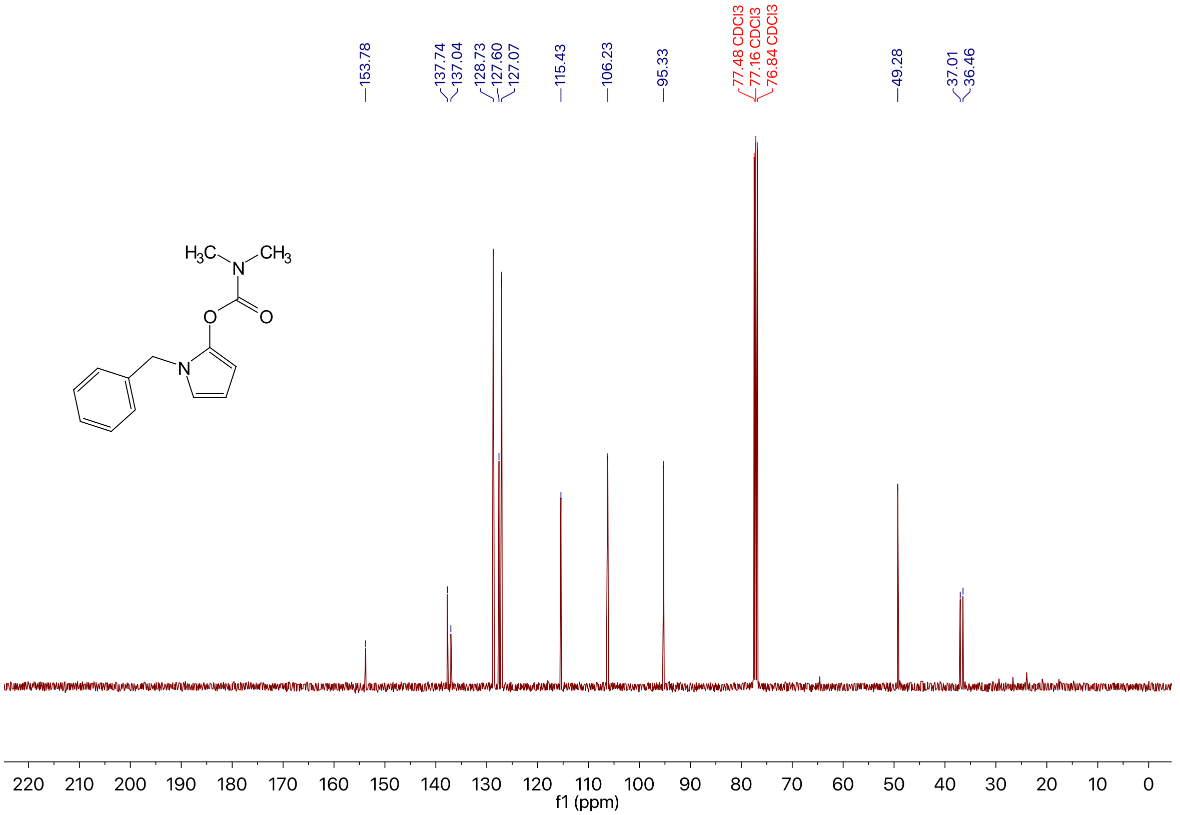

^{1}H NMR spectrum of 1-tosyl-1*H*-pyrrol-2-yl-dimethylcarbamate (1v)

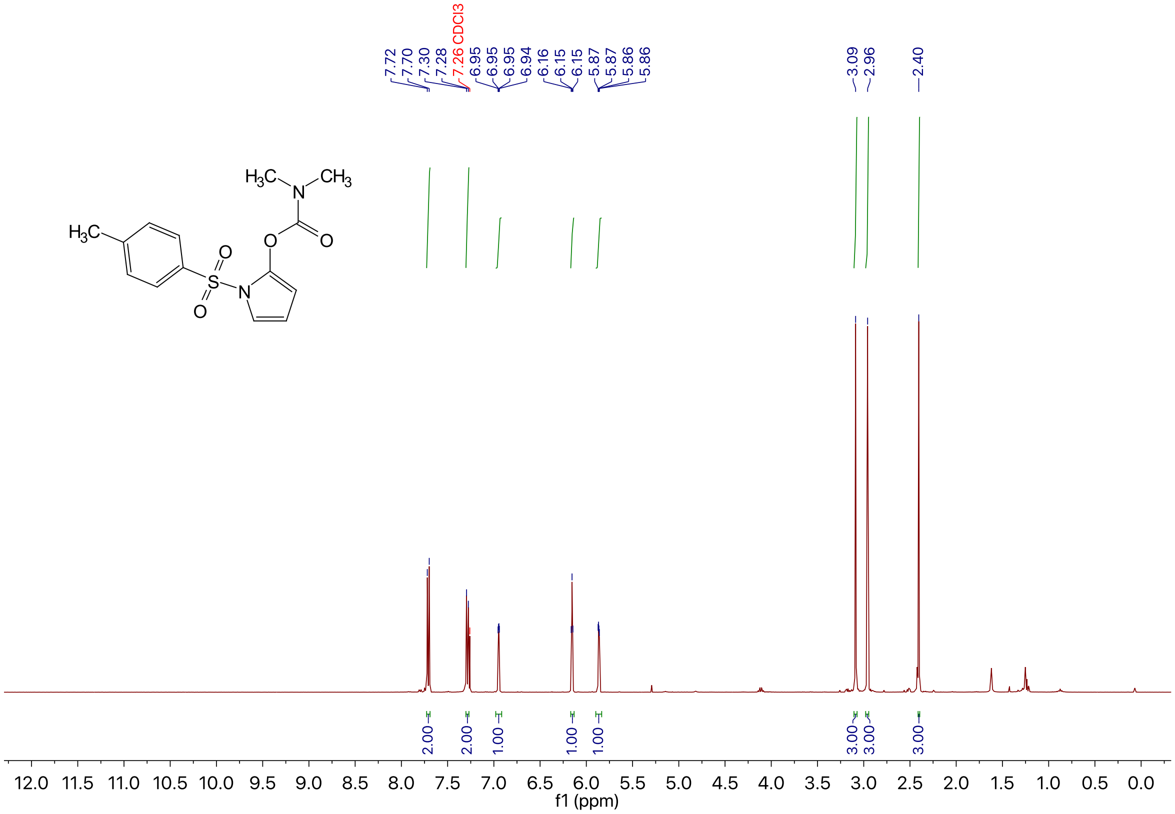

^{13}C NMR spectrum of (1-tosyl-1*H*-pyrrol-2-yl-dimethylcarbamate (1v)

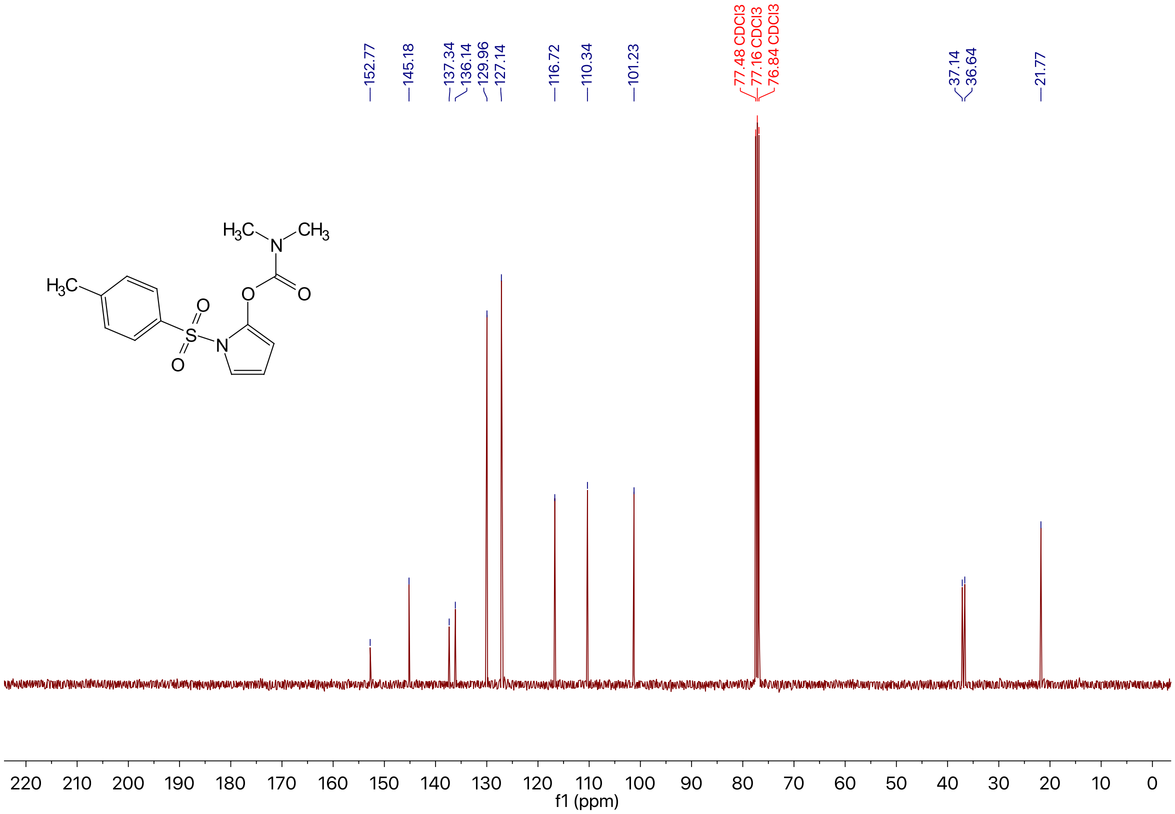

^{1}H NMR spectrum of 5-cyclopropylfuran-2(5*H*)-one

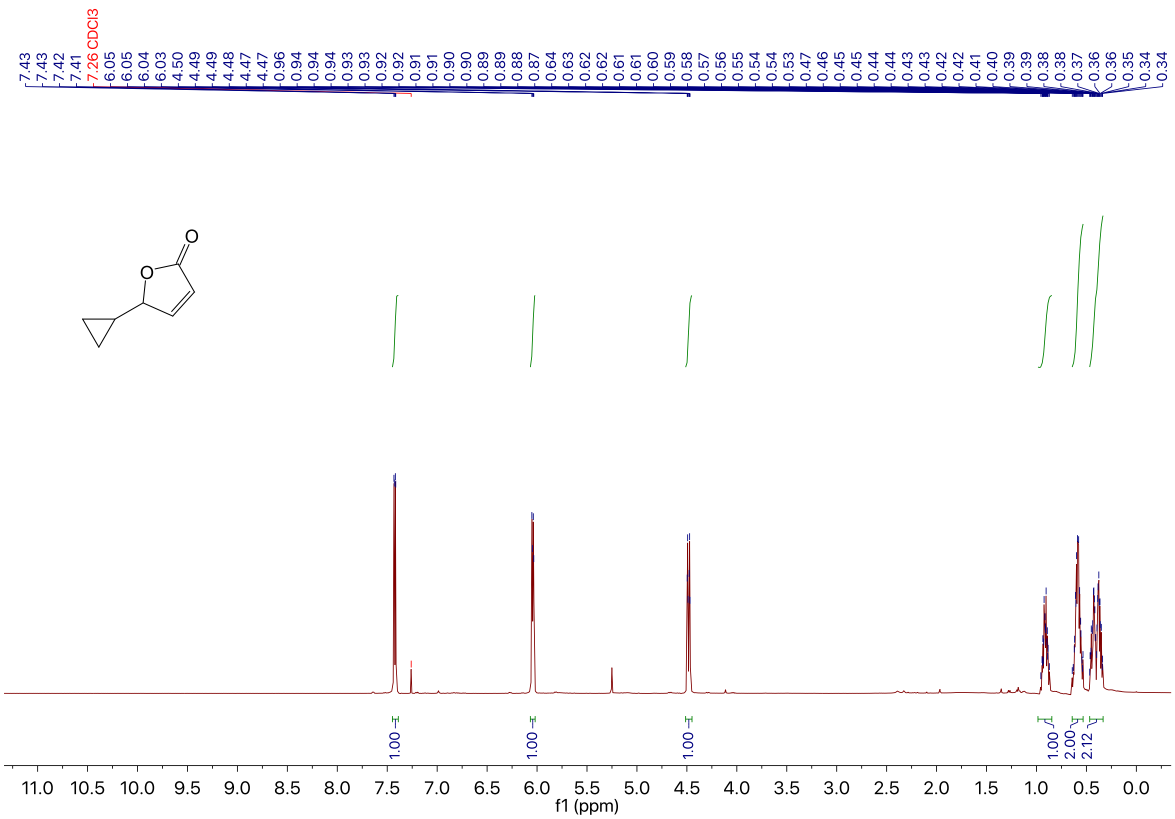

^{13}C NMR spectrum of 5-cyclopropylfuran-2(5*H*)-one

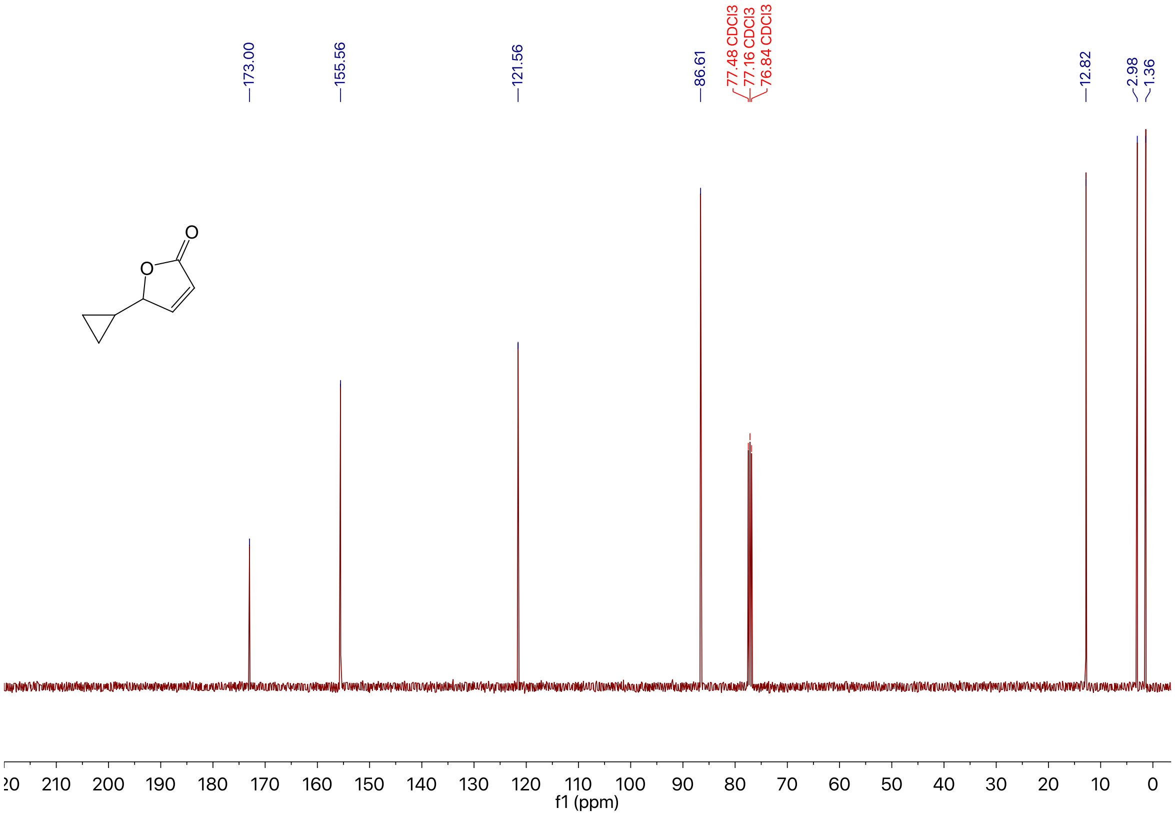

^{1}H NMR spectrum of 5-(*o*-tolyl)furan-2(5*H*)-one

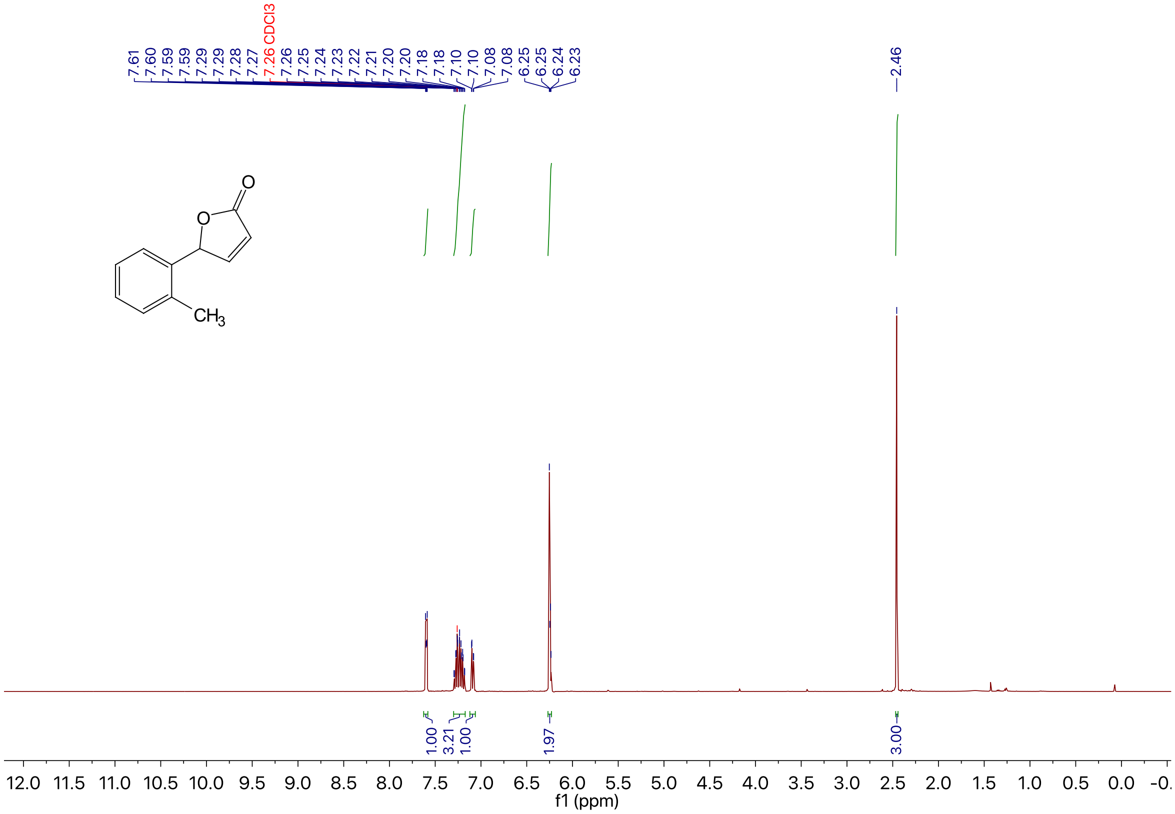

^{13}C NMR spectrum of 5-(*o*-tolyl)furan-2(5*H*)-one

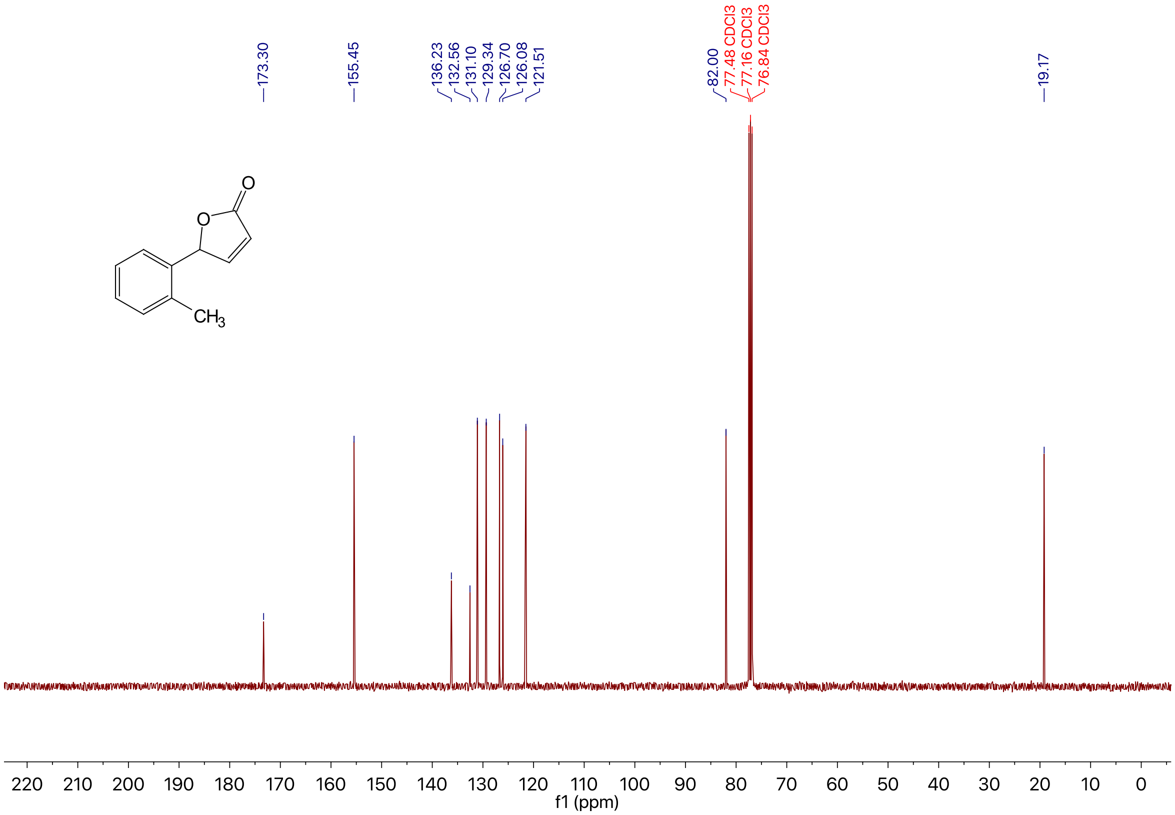

^{1}H NMR spectrum of 5-(4-(trifluoromethyl)phenyl)furan-2(3*H*)-one

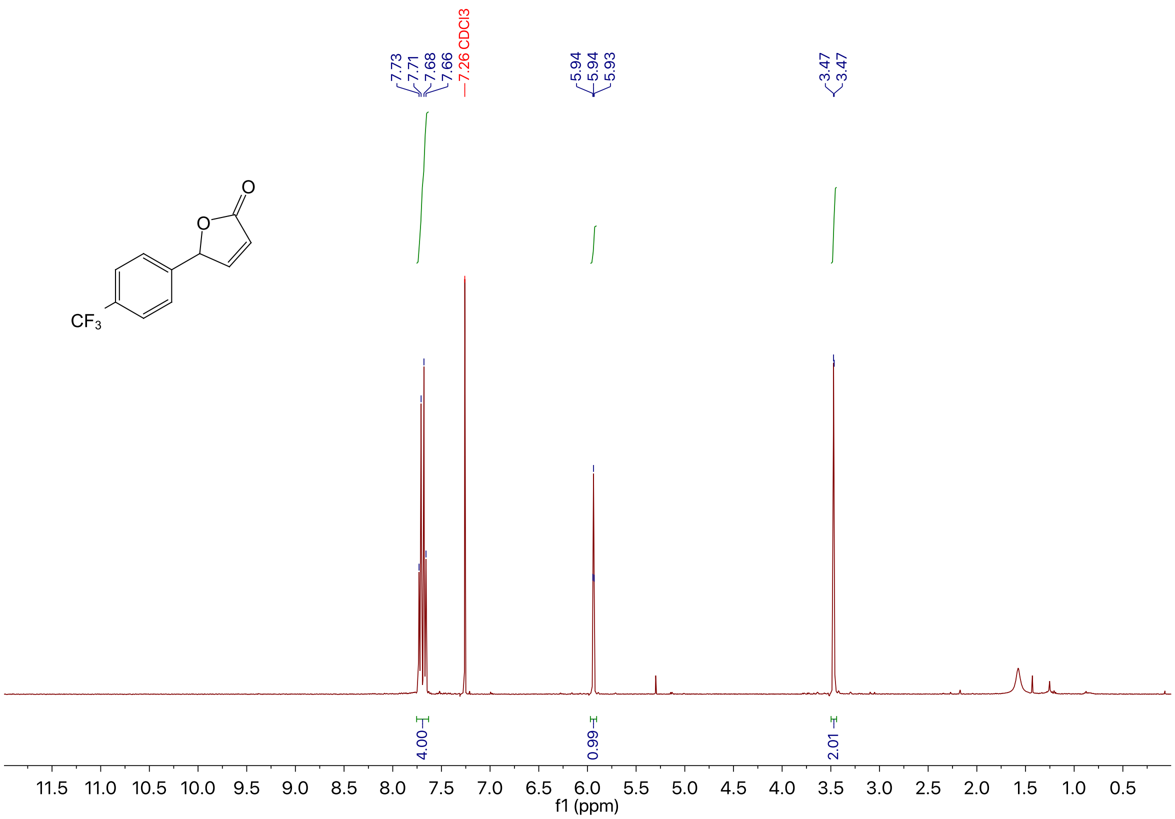

^{13}C NMR spectrum of 5-(4-(trifluoromethyl)phenyl)furan-2(3*H*)-one

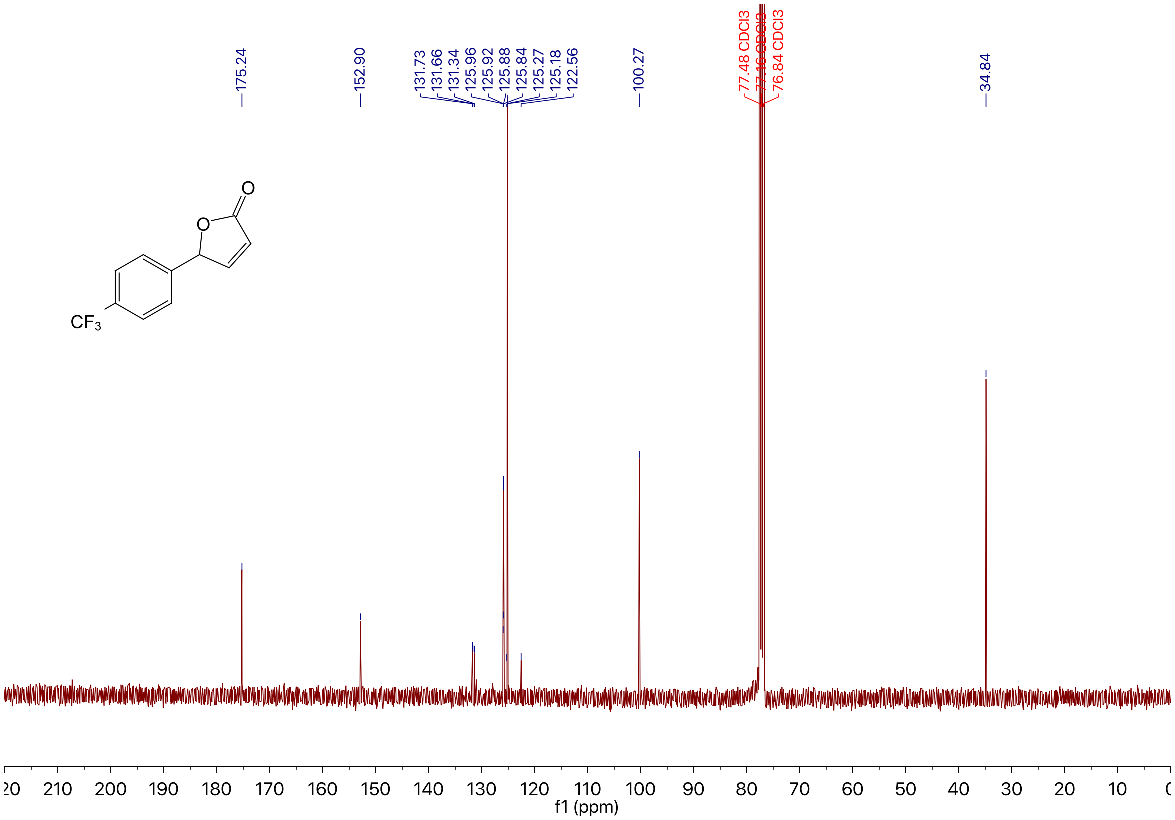

^{1}H NMR spectrum of 5-(4-nitrophenyl)furan-2(3*H*)-one

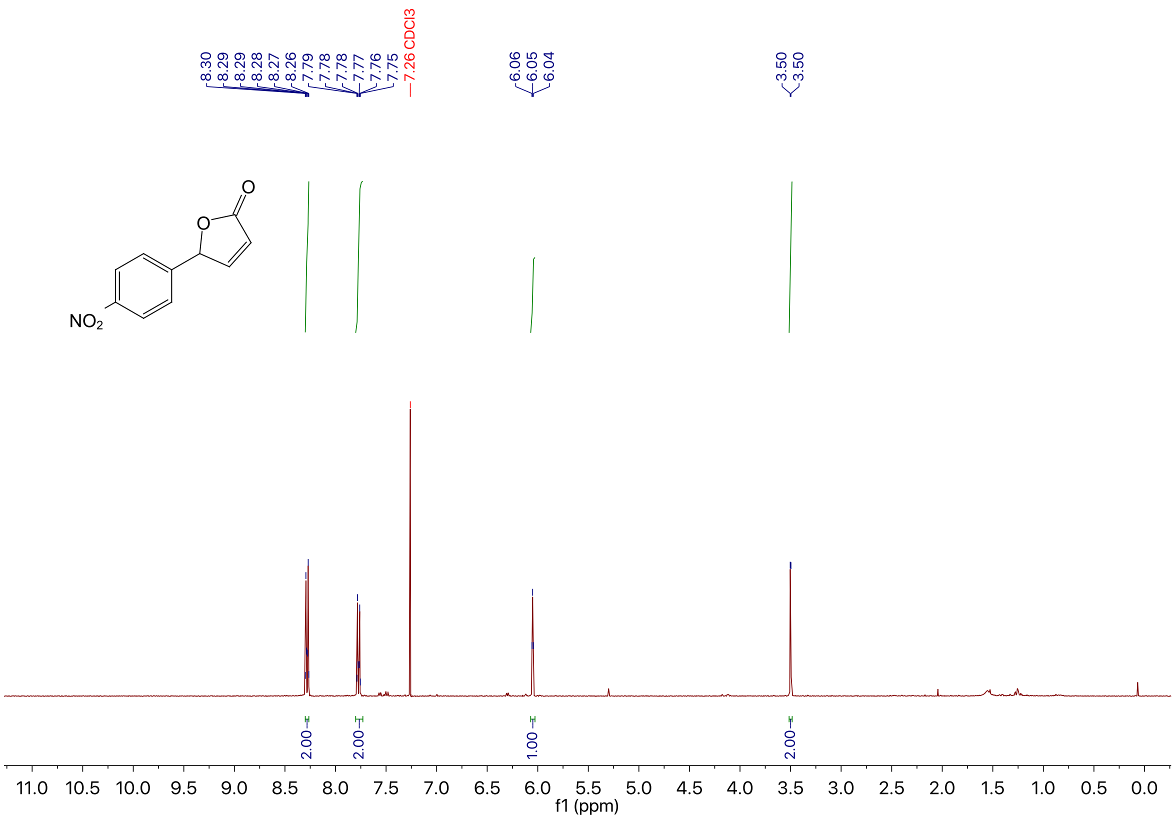

^{13}C NMR spectrum of 5-(4-nitrophenyl)furan-2(3*H*)-one

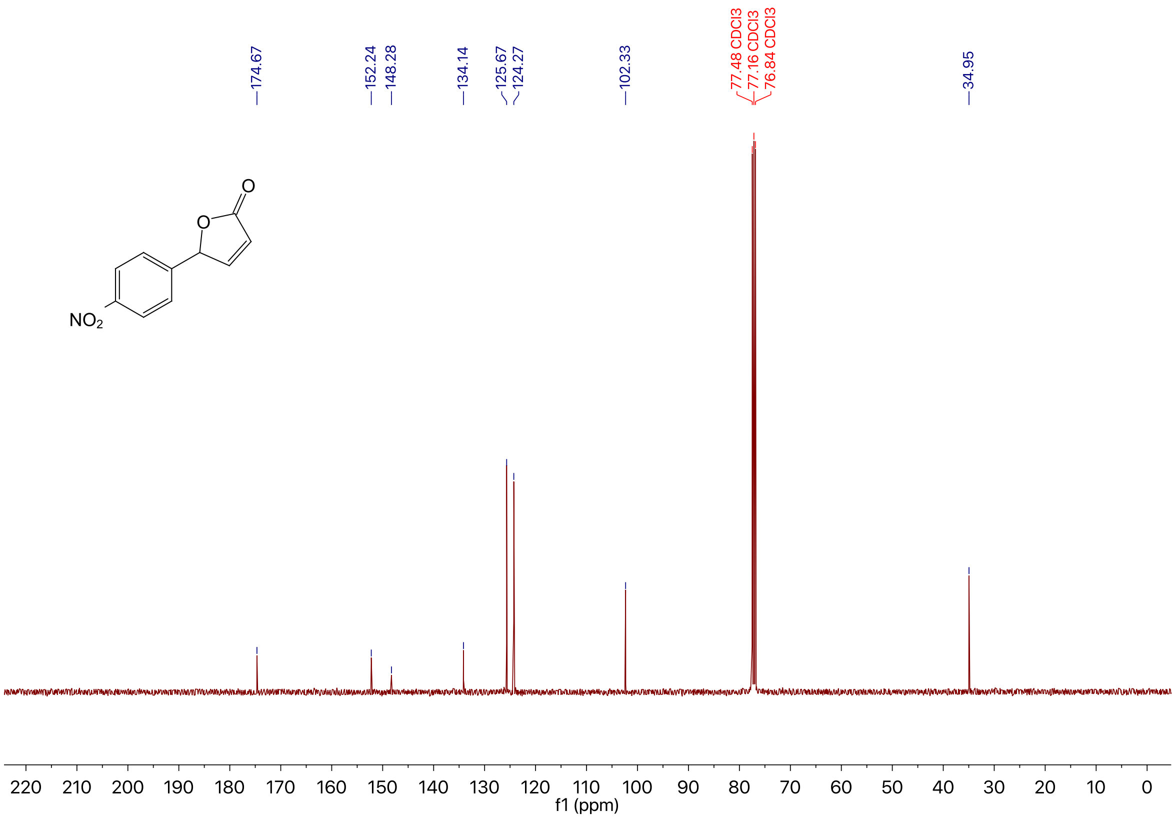

^{1}H NMR spectrum of 5-(oct-1-yn-1-yl)furan-2(5*H*)-one

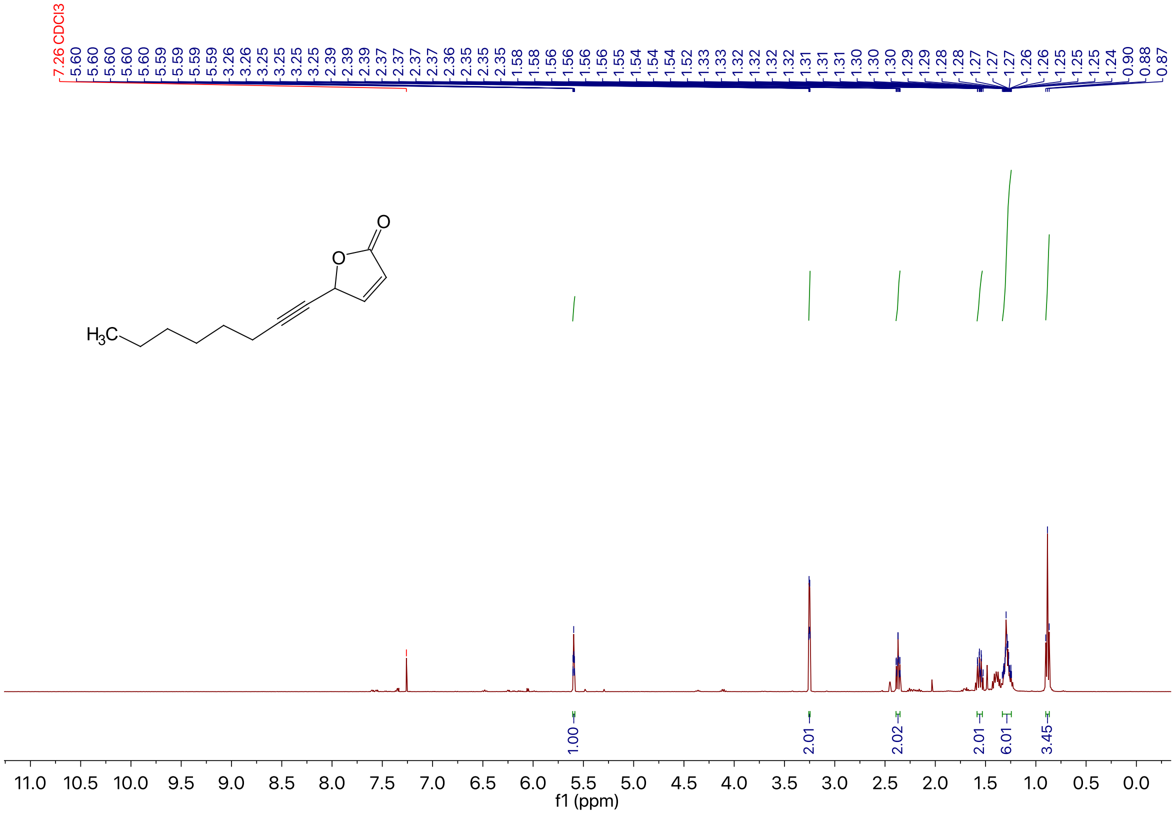

^{13}C NMR spectrum of (5-(oct-1-yn-1-yl)furan-2(5*H*)-one

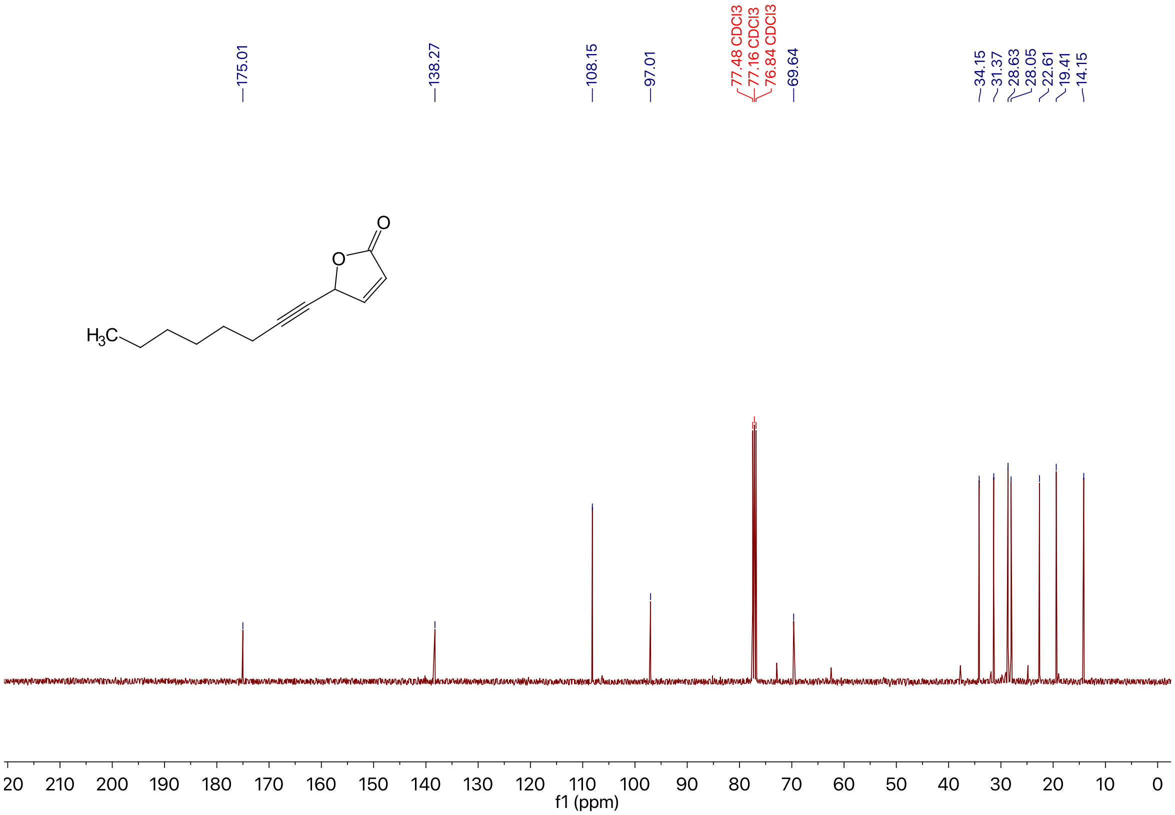

^{1}H NMR spectrum of methyl (*E*)-3-(2-((dimethylcarbamoyl)oxy)furan-3-yl)acrylate (2a)

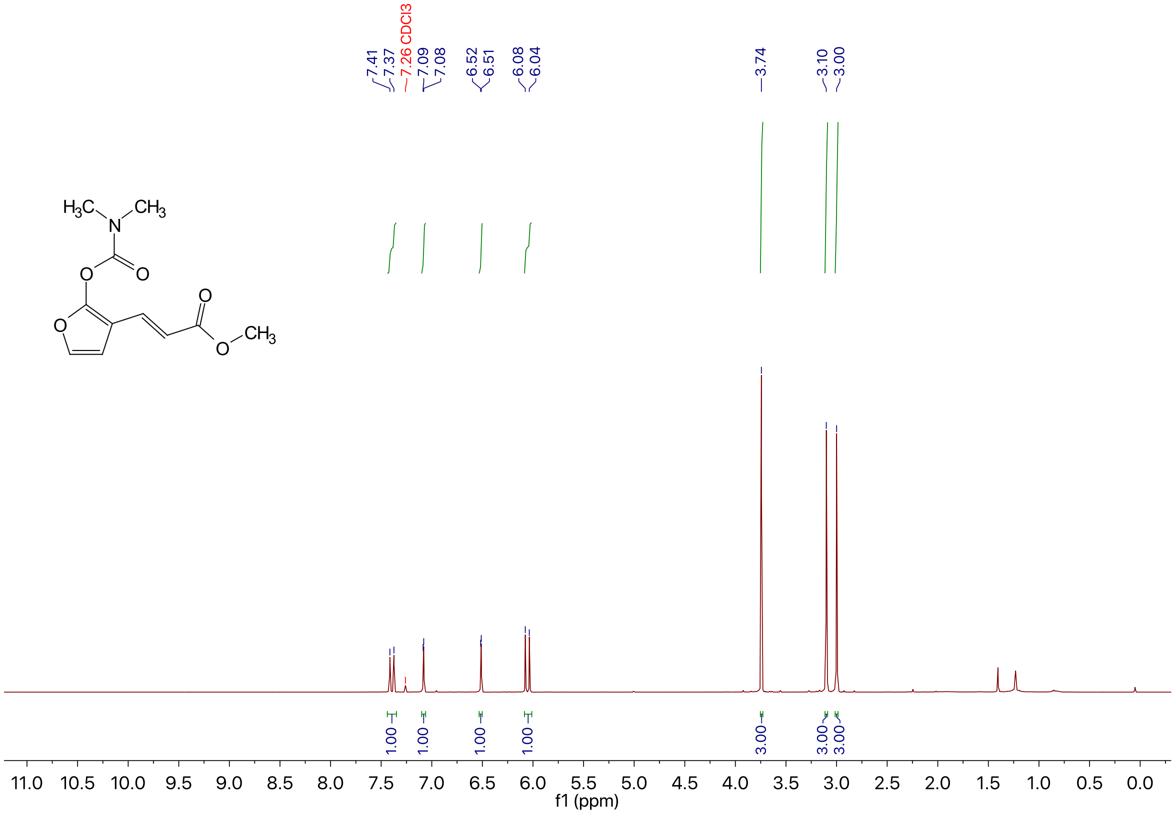

^{13}C NMR spectrum of methyl (*E*)-3-(2-((dimethylcarbamoyl)oxy)furan-3-yl)acrylate (2a)

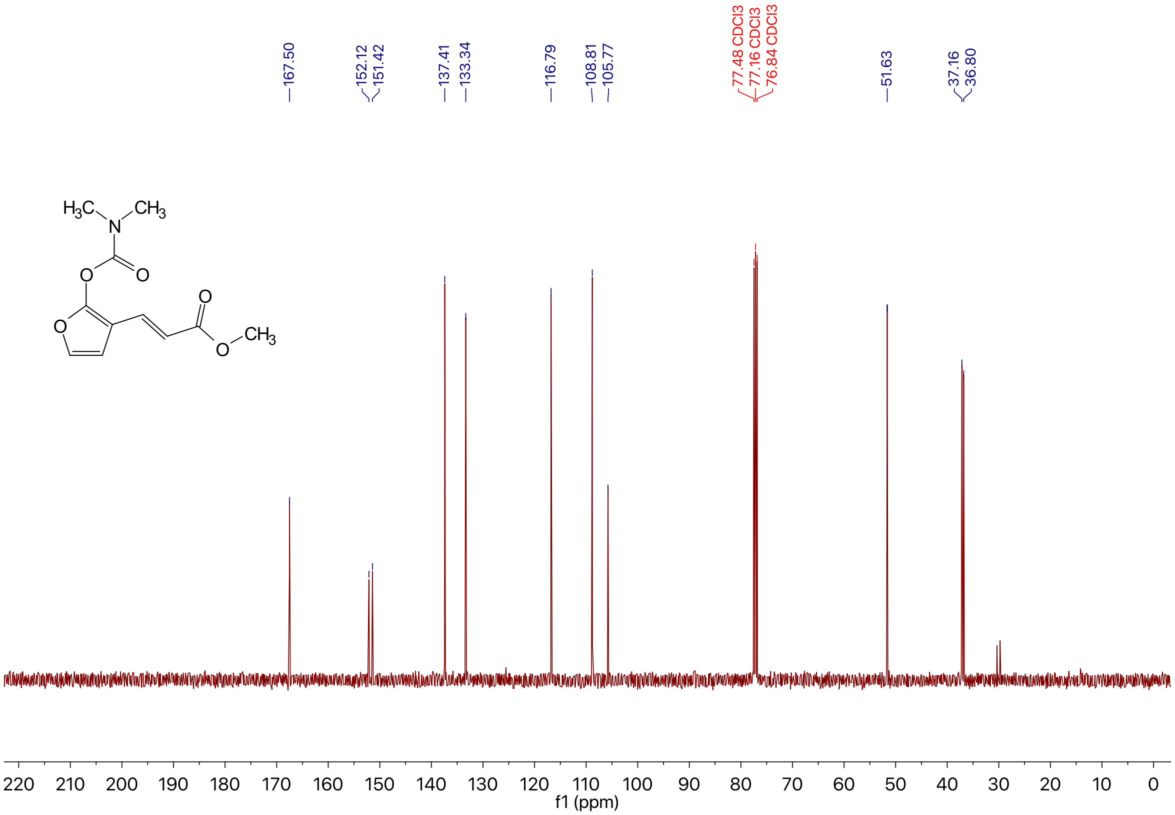

^{1}H NMR spectrum of ethyl (*E*)-3-(2-((dimethylcarbamoyl)oxy)furan-3-yl)acrylate (2b)

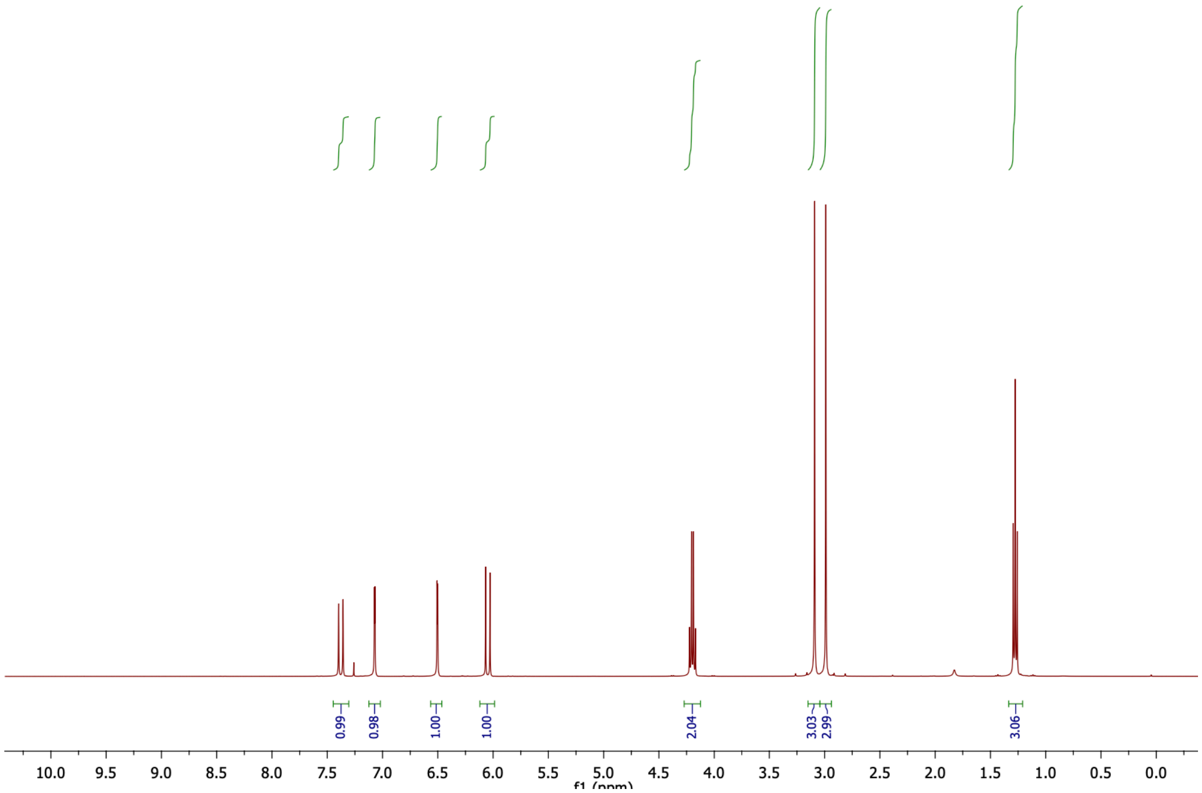

^{13}C NMR spectrum of ethyl (*E*)-3-(2-((dimethylcarbamoyl)oxy)furan-3-yl)acrylate (2b)

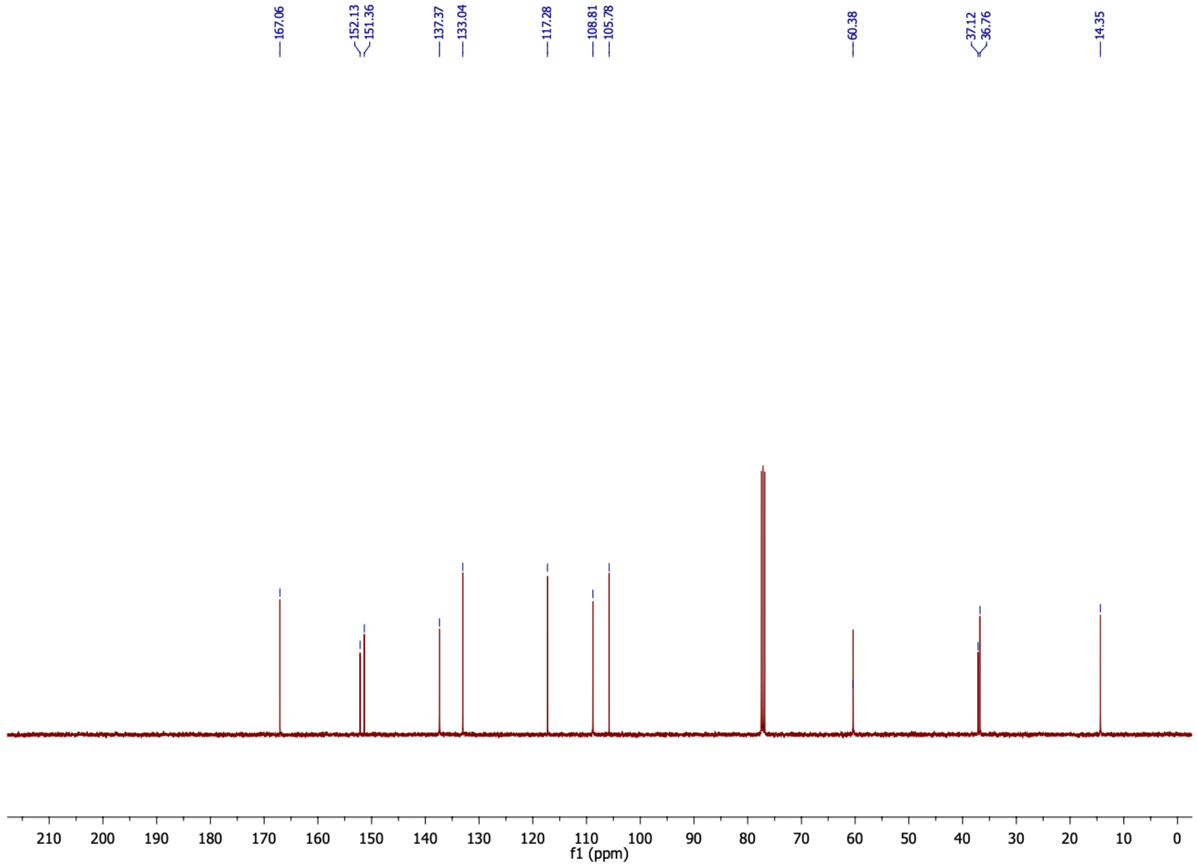

^{1}H NMR spectrum of butyl (*E*)-3-(2-((dimethylcarbamoyl)oxy)furan-3-yl)acrylate (2c)

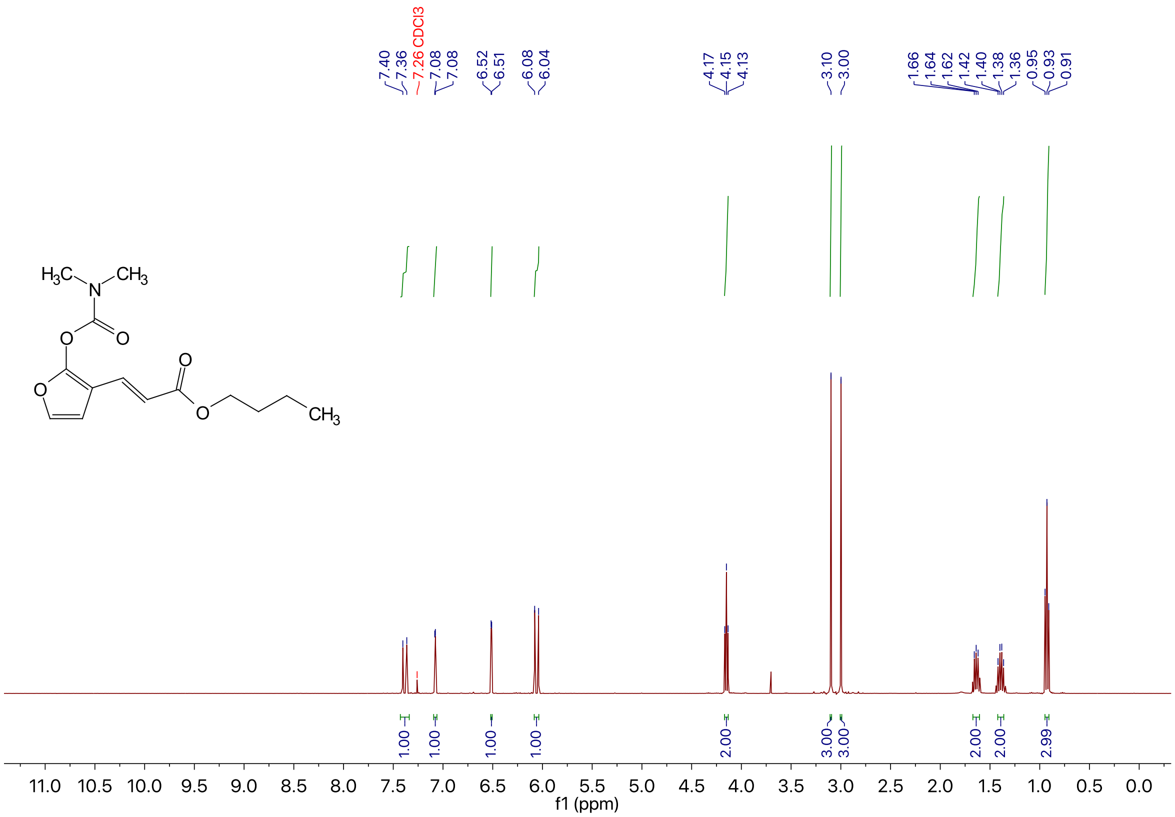

^{13}C NMR spectrum of butyl (*E*)-3-(2-((dimethylcarbamoyl)oxy)furan-3-yl)acrylate (2c)

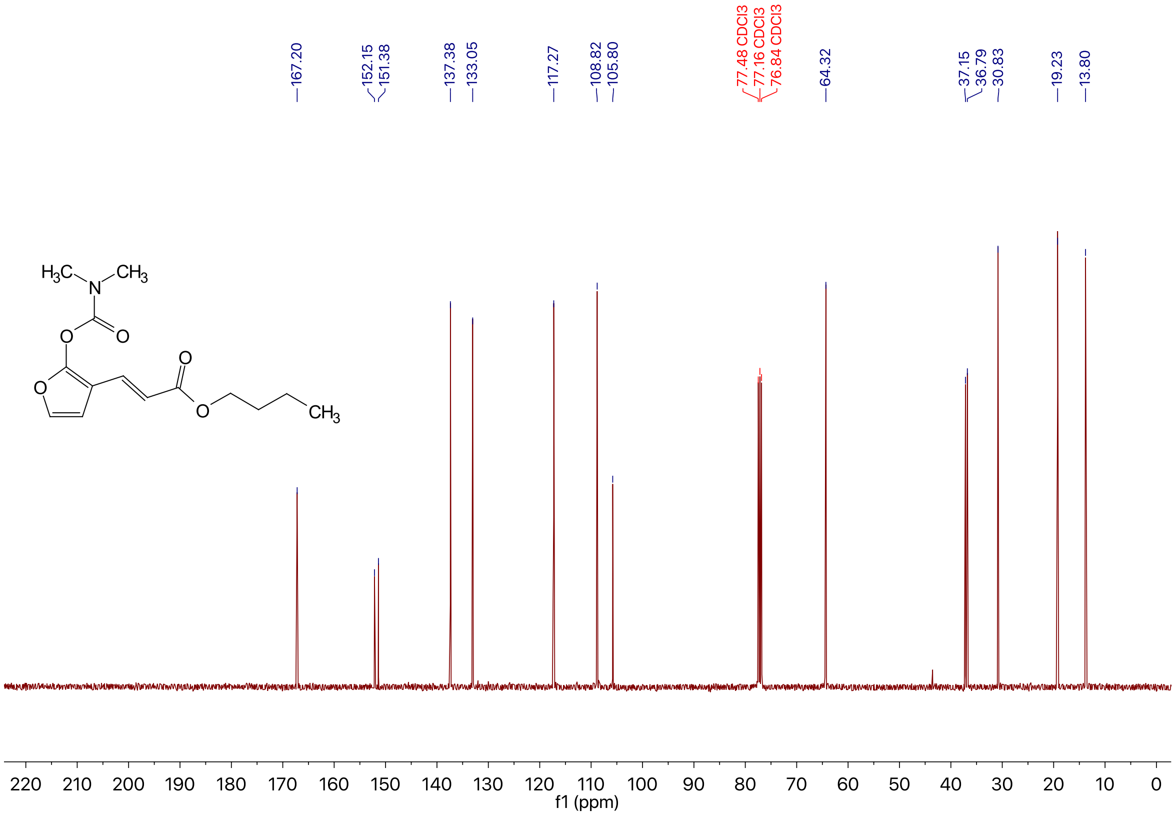

^{1}H NMR spectrum of butyl (*E*)-3-(2-((diethylcarbamoyl)oxy)furan-3-yl)acrylate

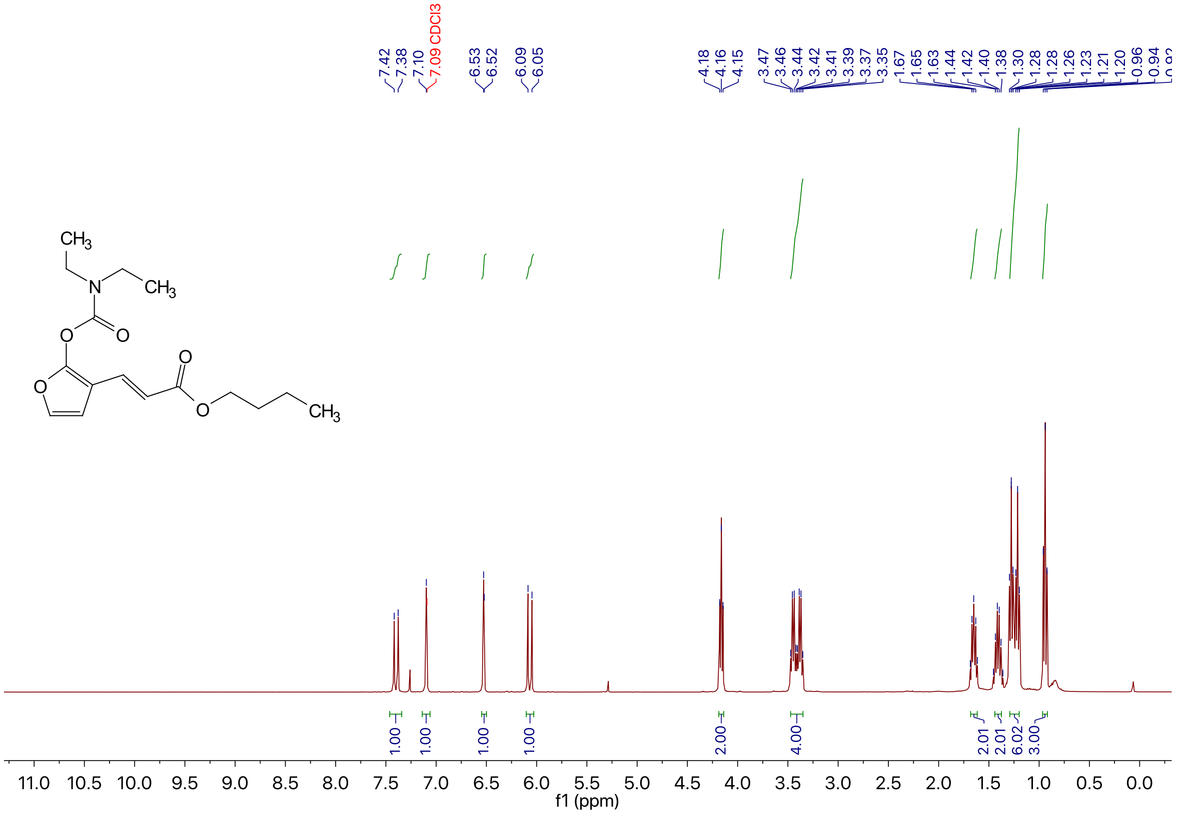

^{13}C NMR spectrum of butyl (*E*)-3-(2-((diethylcarbamoyl)oxy)furan-3-yl)acrylate

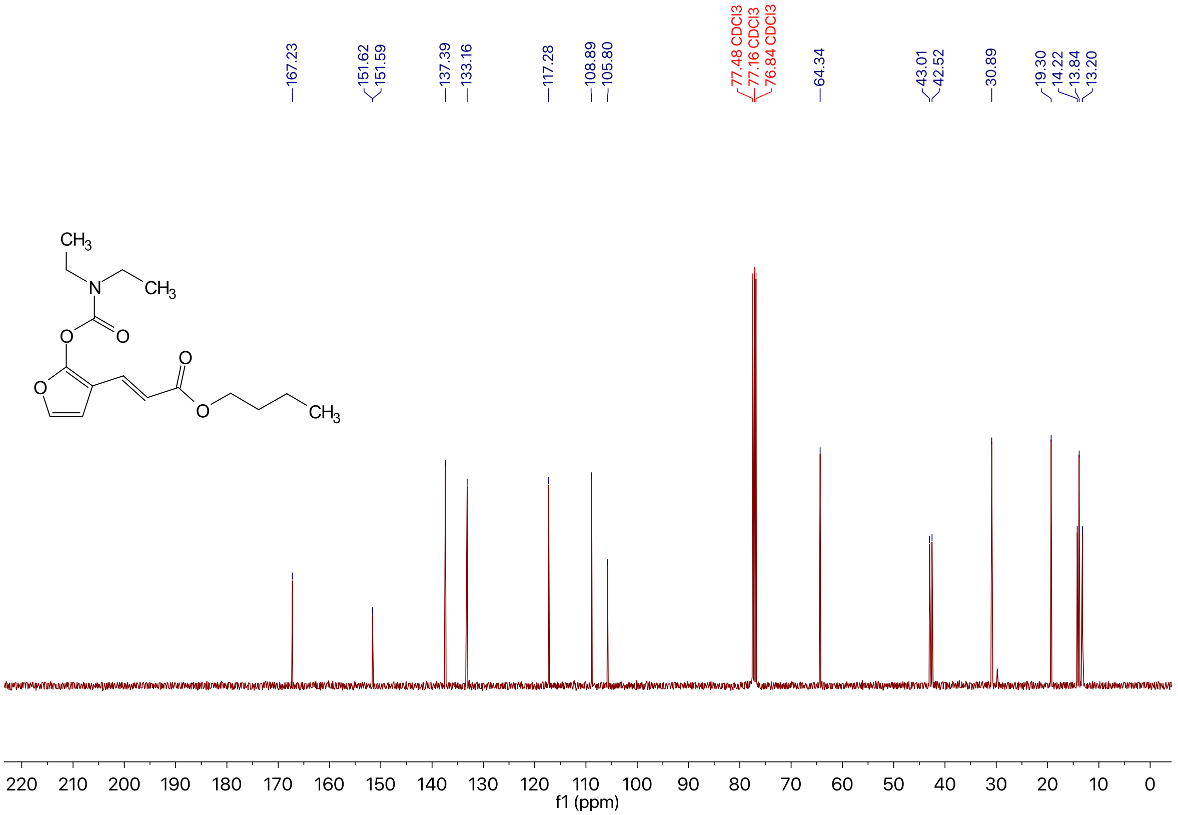

^{1}H NMR spectrum of butyl (*E*)-3-(2-((diisopropylcarbamoyl)oxy)furan-3-yl)acrylate

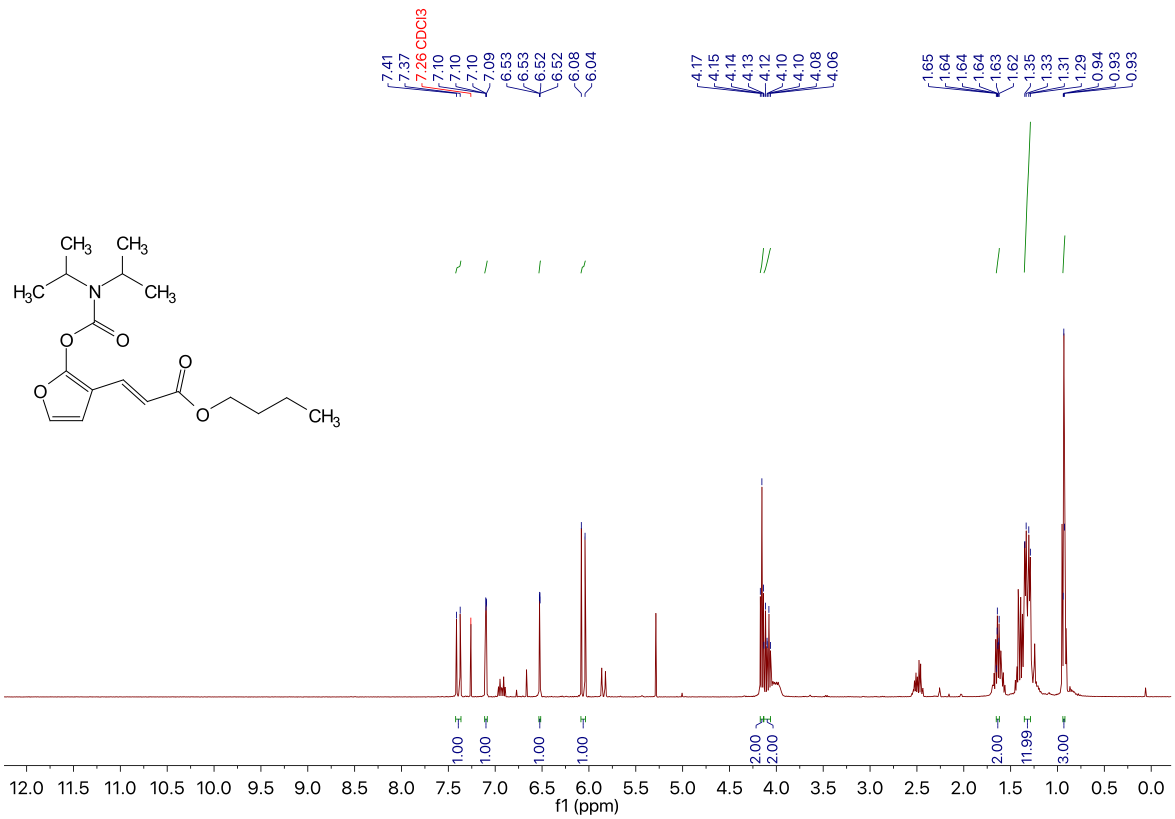

^{13}C NMR spectrum of butyl (*E*)-3-(2-((diisopropylcarbamoyl)oxy)furan-3-yl)acrylate

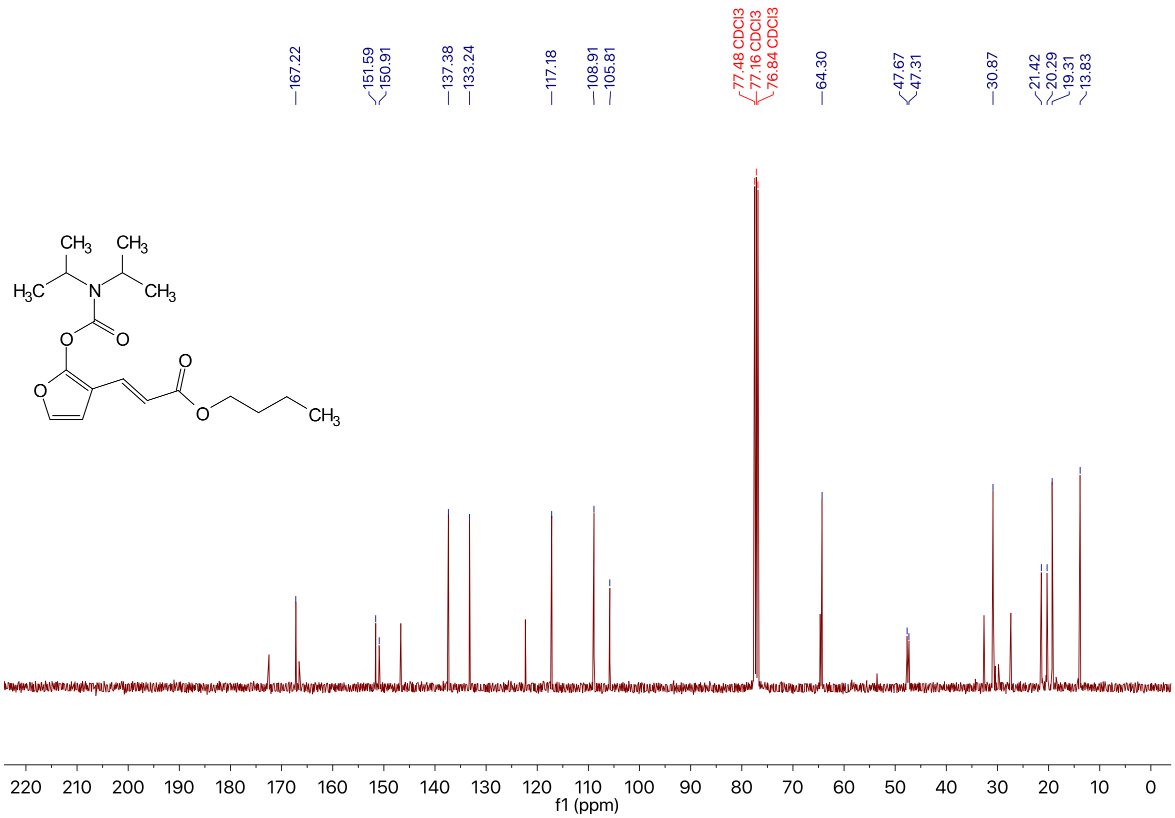

^{1}H NMR spectrum of (*E*)-3-(3-butoxy-3-oxoprop-1-en-1-yl)furan-2-yl-pyrrolidine-1-carboxylate

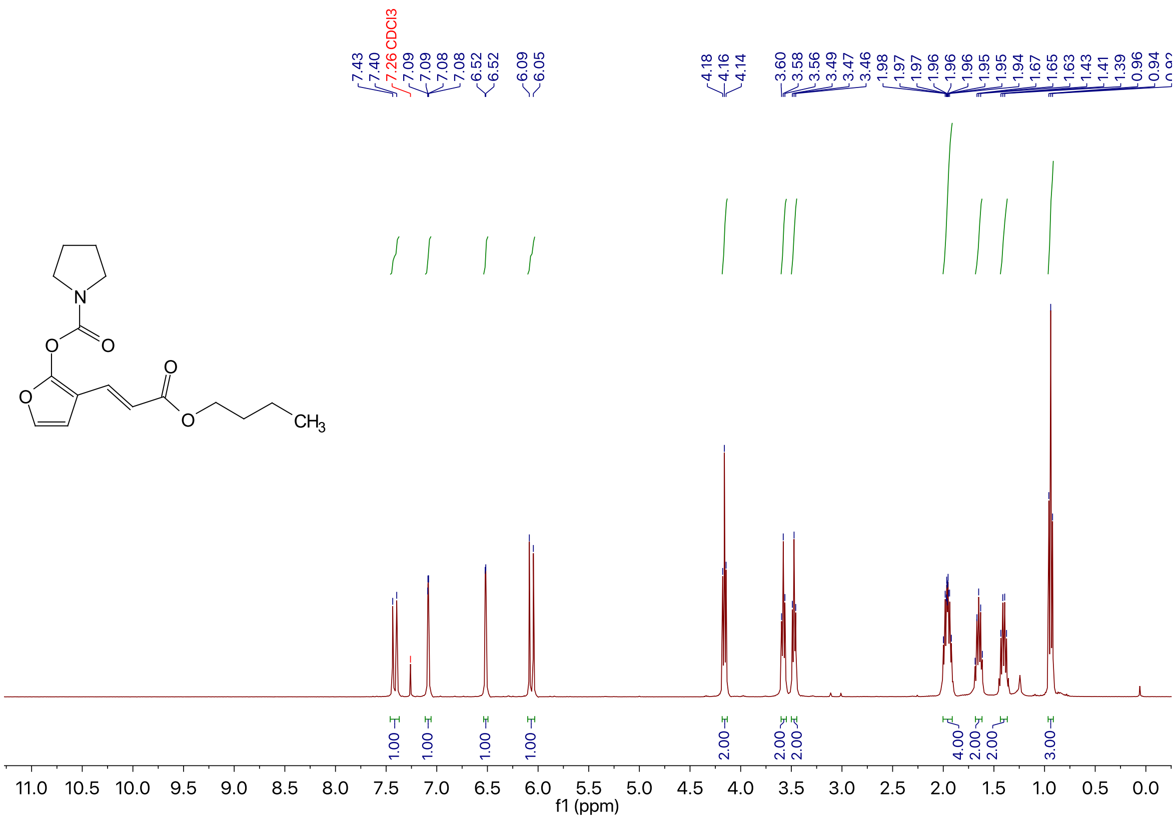

^{13}C NMR spectrum of (*E*)-3-(3-butoxy-3-oxoprop-1-en-1-yl)furan-2-yl pyrrolidine-1-carboxylate

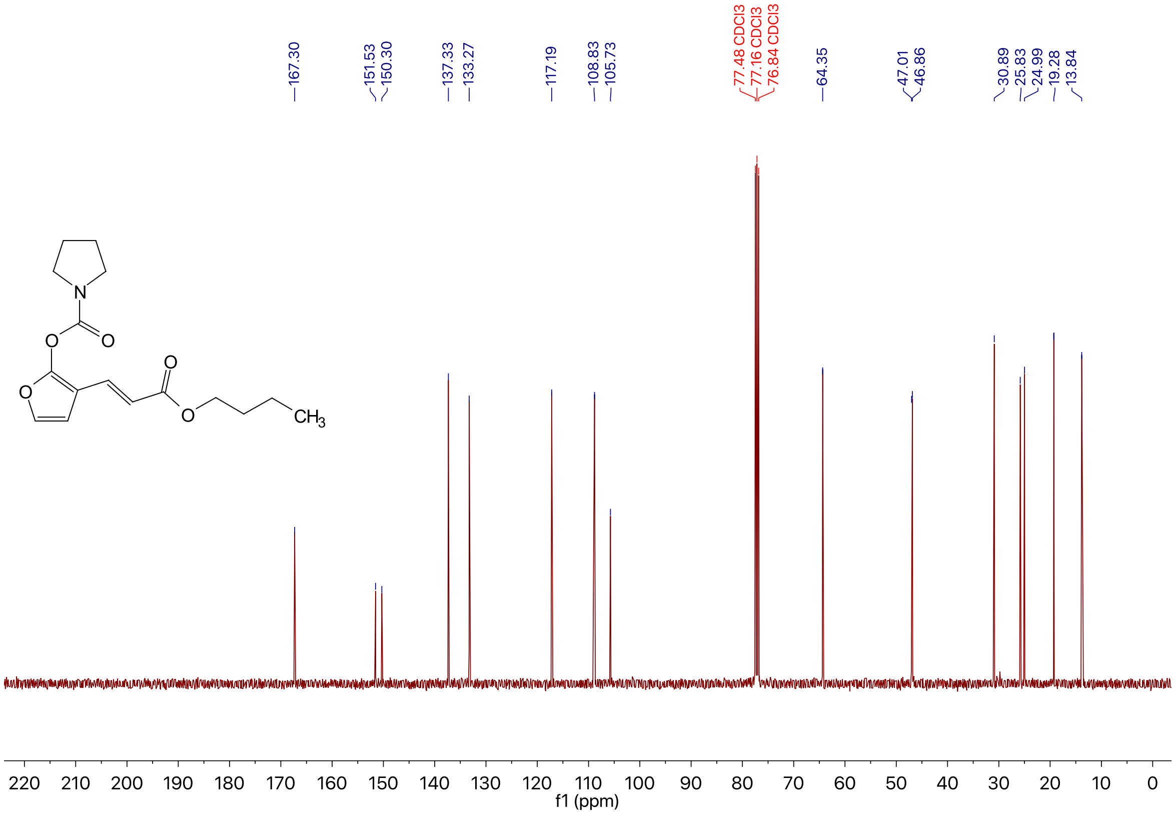

^{1}H NMR spectrum of (*E*)-3-(3-butoxy-3-oxoprop-1-en-1-yl)furan-2-yl-morpholine-4-carboxylate

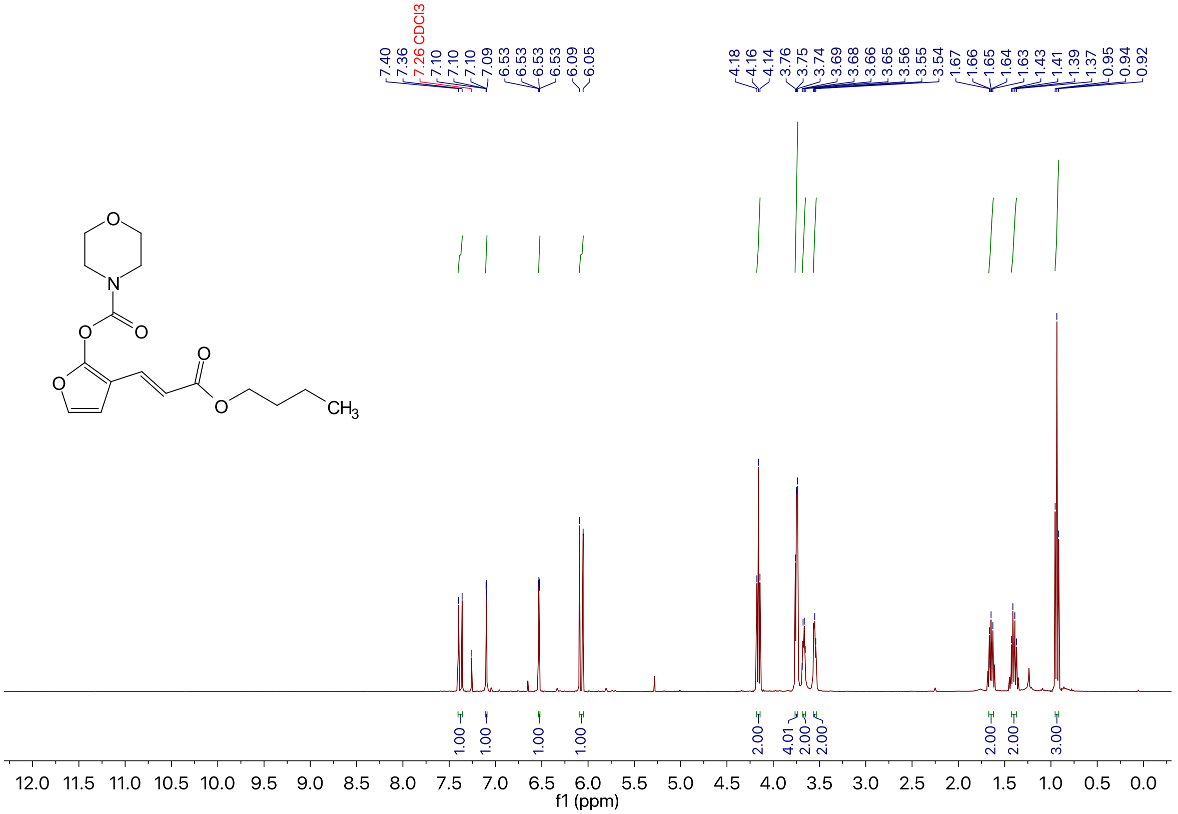

^{13}C NMR spectrum of (*E*)-3-(3-butoxy-3-oxoprop-1-en-1-yl)furan-2-yl-morpholine-4-carboxylate

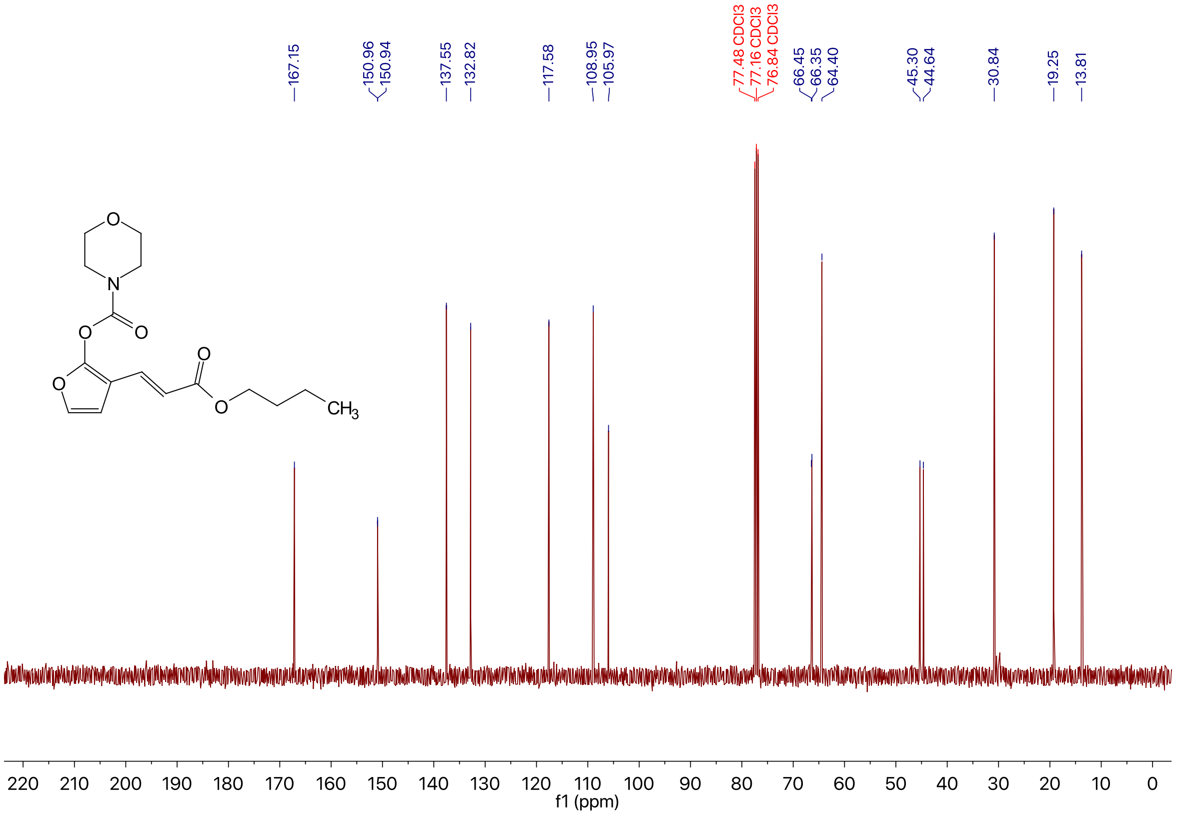

^{1}H NMR spectrum of *tert*-butyl (*E*)-3-(2-((dimethylcarbamoyl)oxy)furan-3-yl)acrylate (2d)

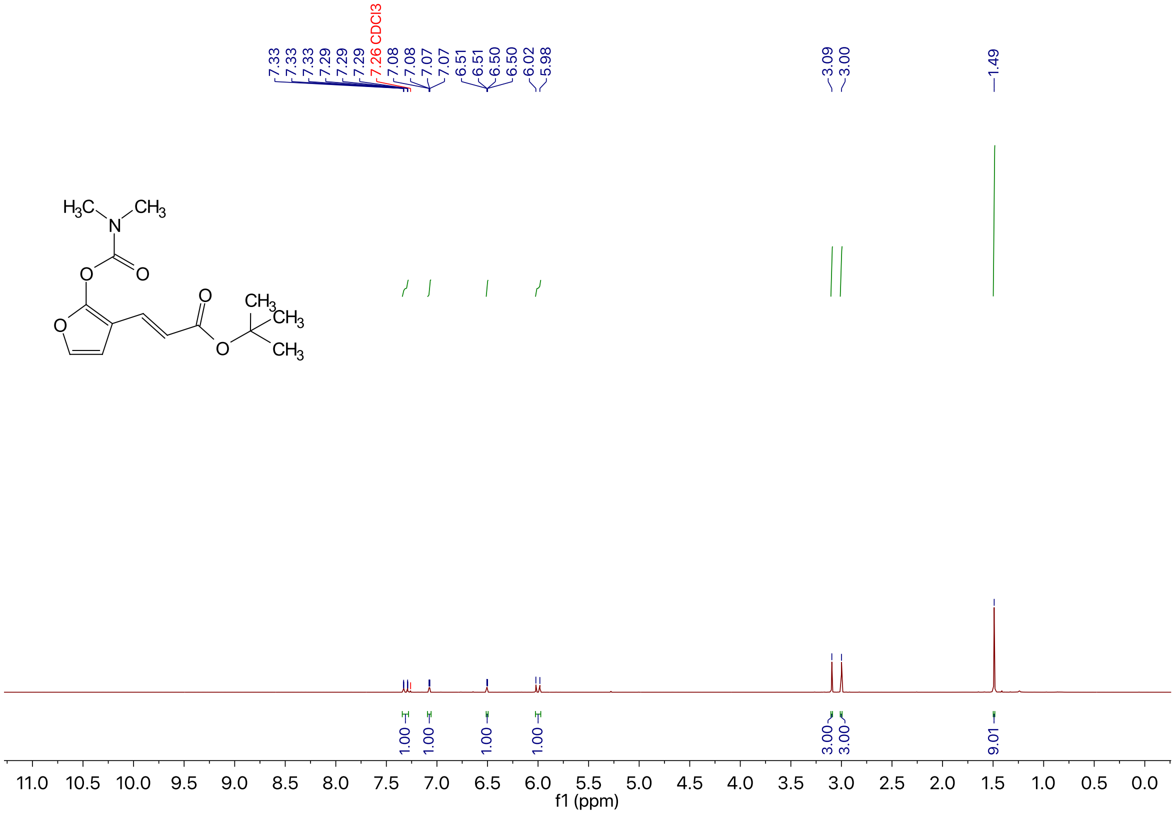

^{13}C NMR spectrum of *tert*-butyl (*E*)-3-(2-((dimethylcarbamoyl)oxy)furan-3-yl)acrylate (2d)

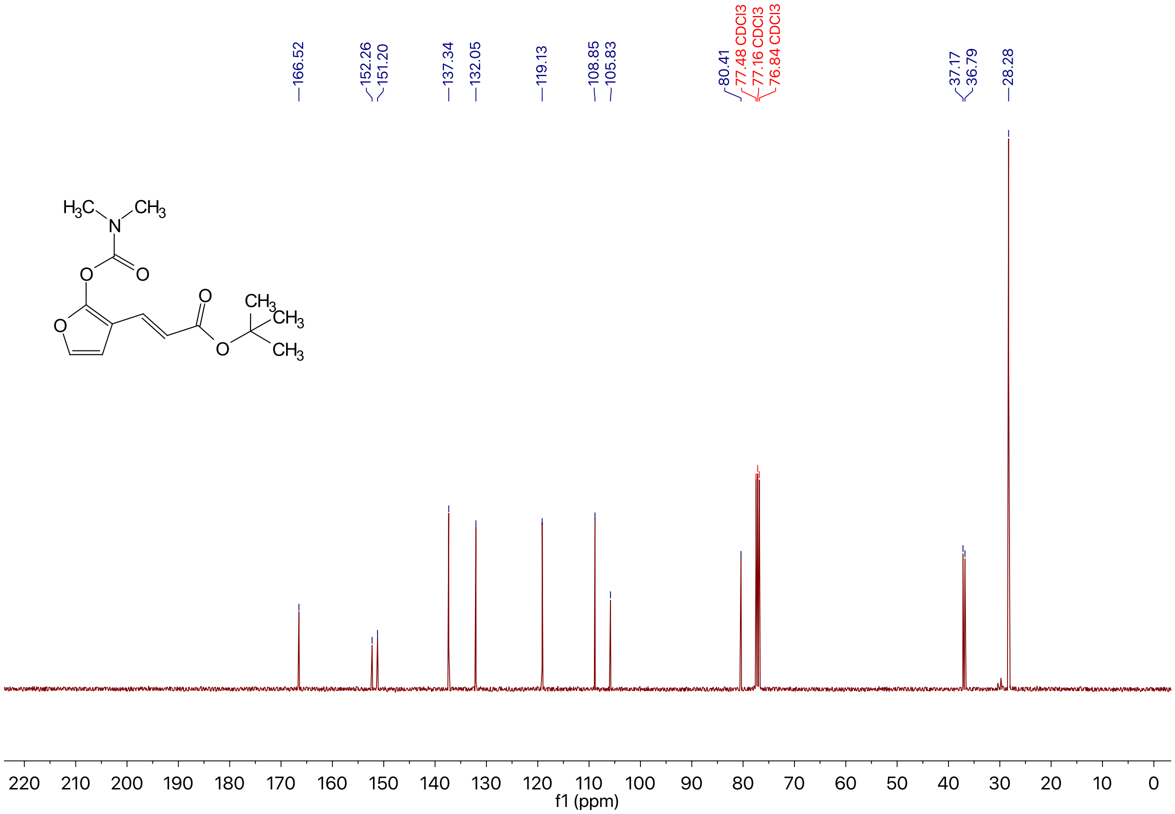

^{1}H NMR spectrum of benzyl (*E*)-3-(2-((dimethylcarbamoyl)oxy)furan-3-yl)acrylate (2e)

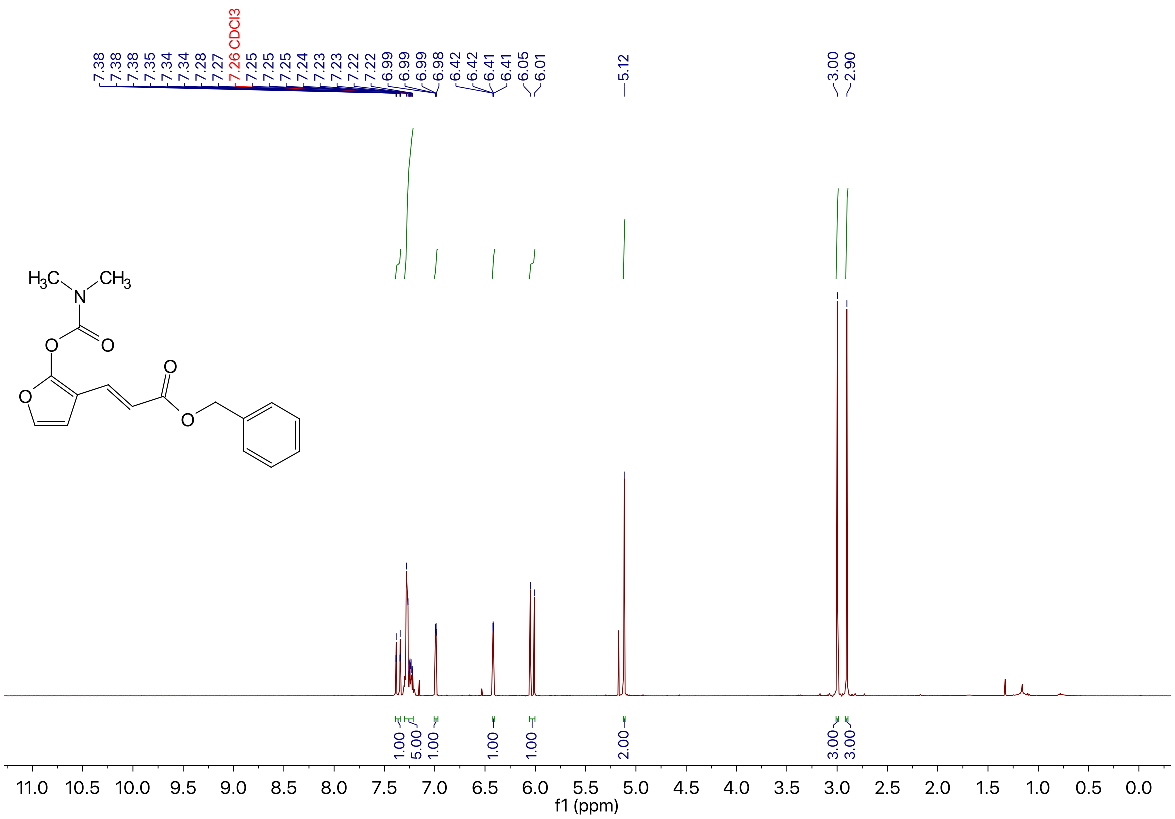

^{13}C NMR spectrum of benzyl (*E*)-3-(2-((dimethylcarbamoyl)oxy)furan-3-yl)acrylate (2e)

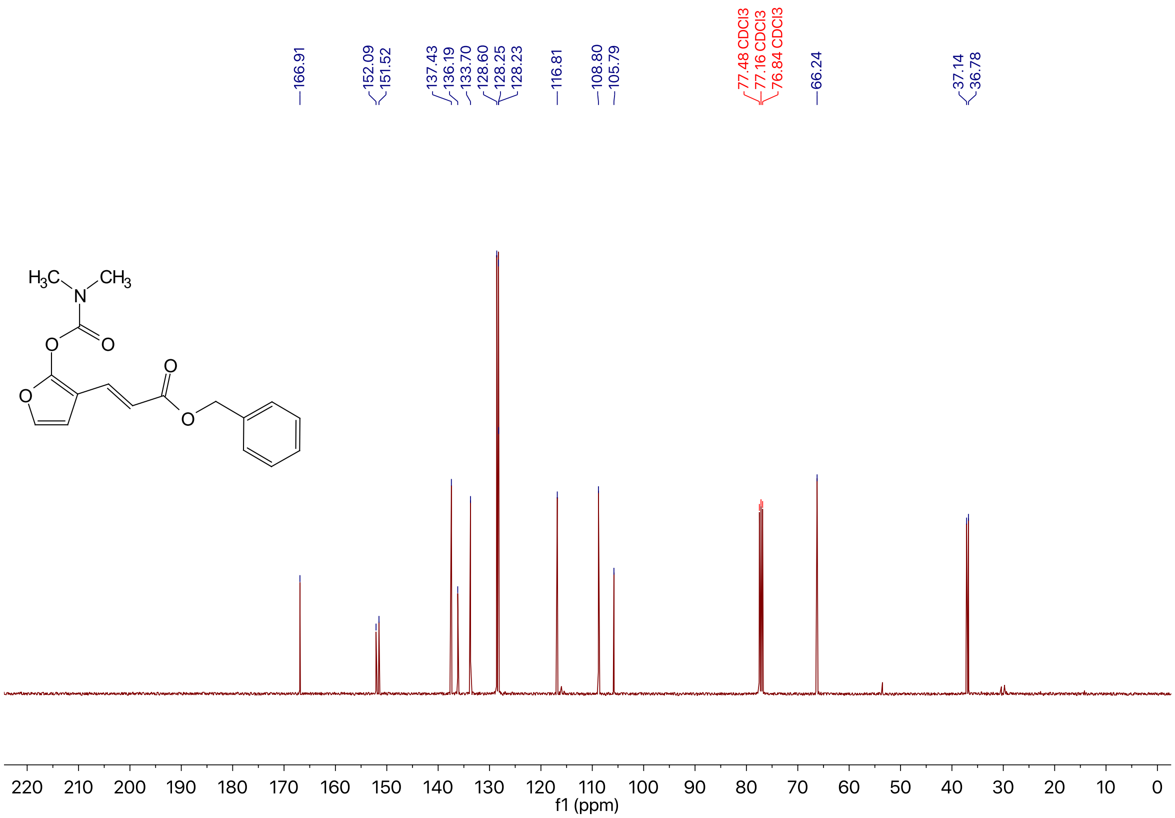

^{1}H NMR spectrum of butyl (*E*)-3-(5-butyl-2-((dimethylcarbamoyl)oxy)furan-3-yl)acrylate (2f)

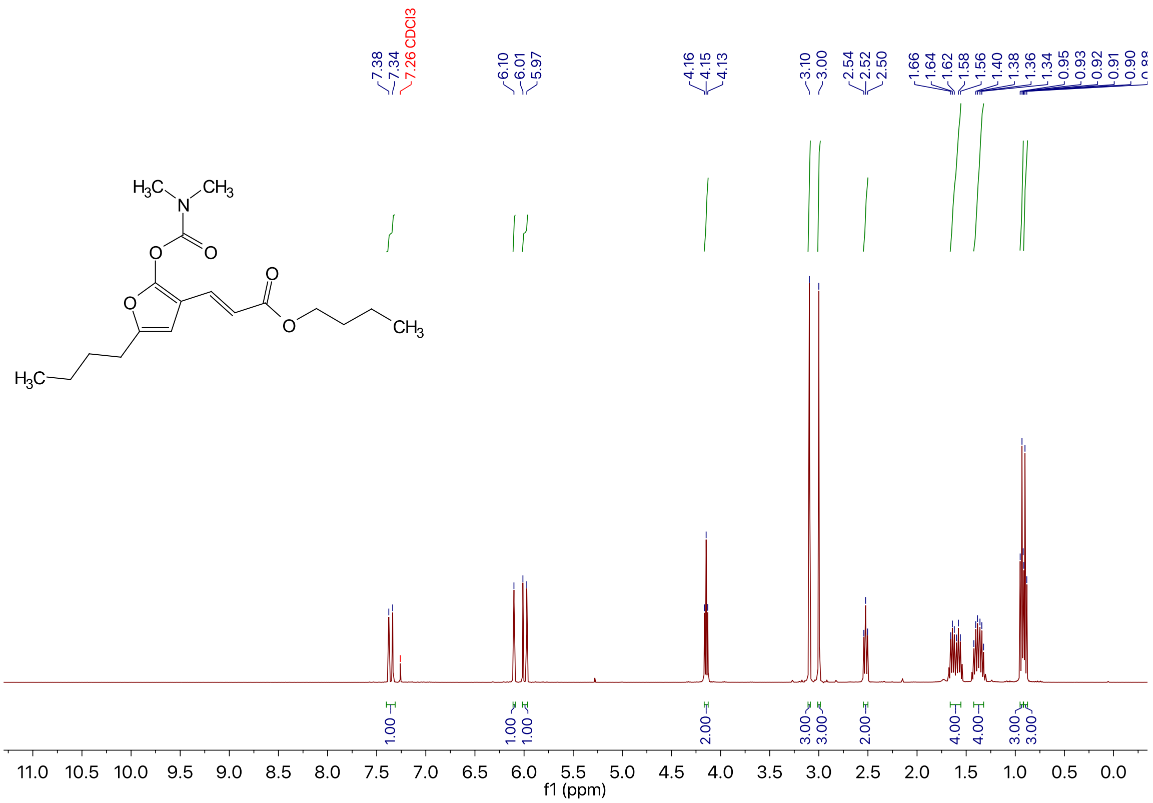

^{13}C NMR spectrum of butyl (*E*)-3-(5-butyl-2-((dimethylcarbamoyl)oxy)furan-3-yl)acrylate (2f)

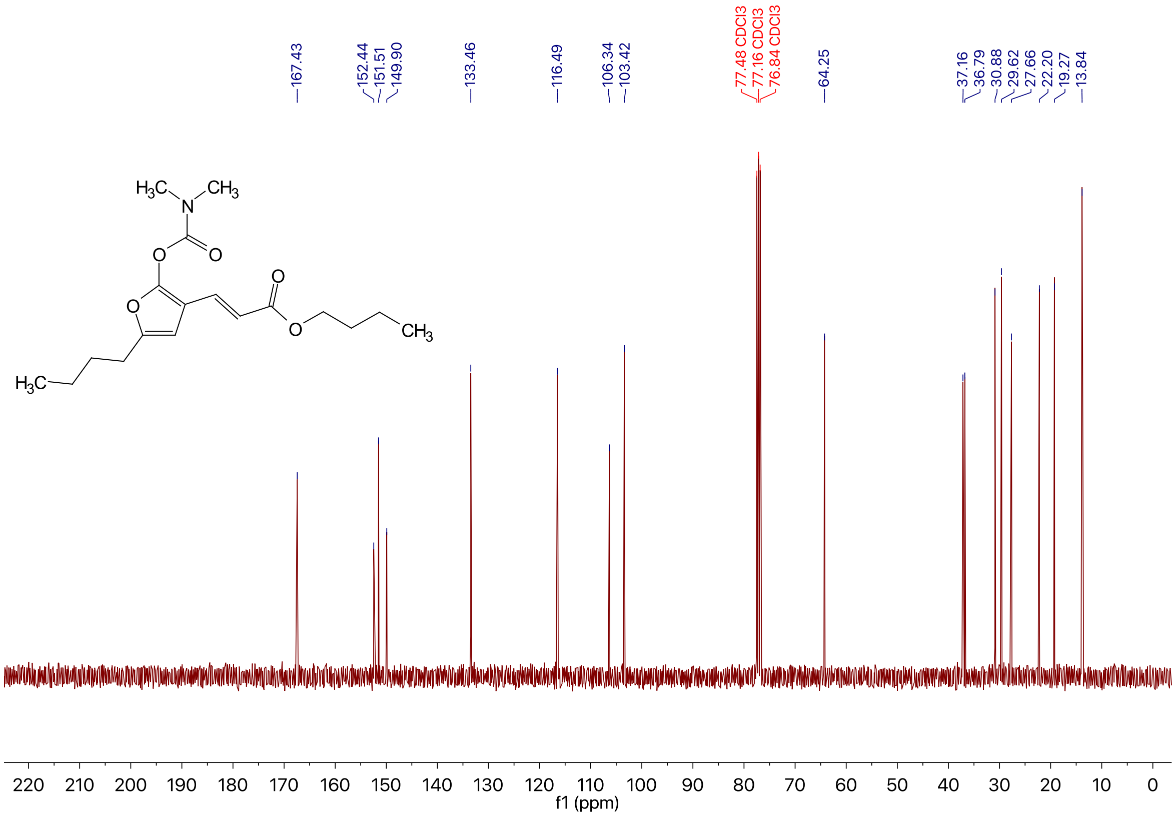

^{1}H NMR spectrum of butyl (*E*)-3-(2-((dimethylcarbamoyl)oxy)-5-phenylfuran-3-yl)acrylate (2g)

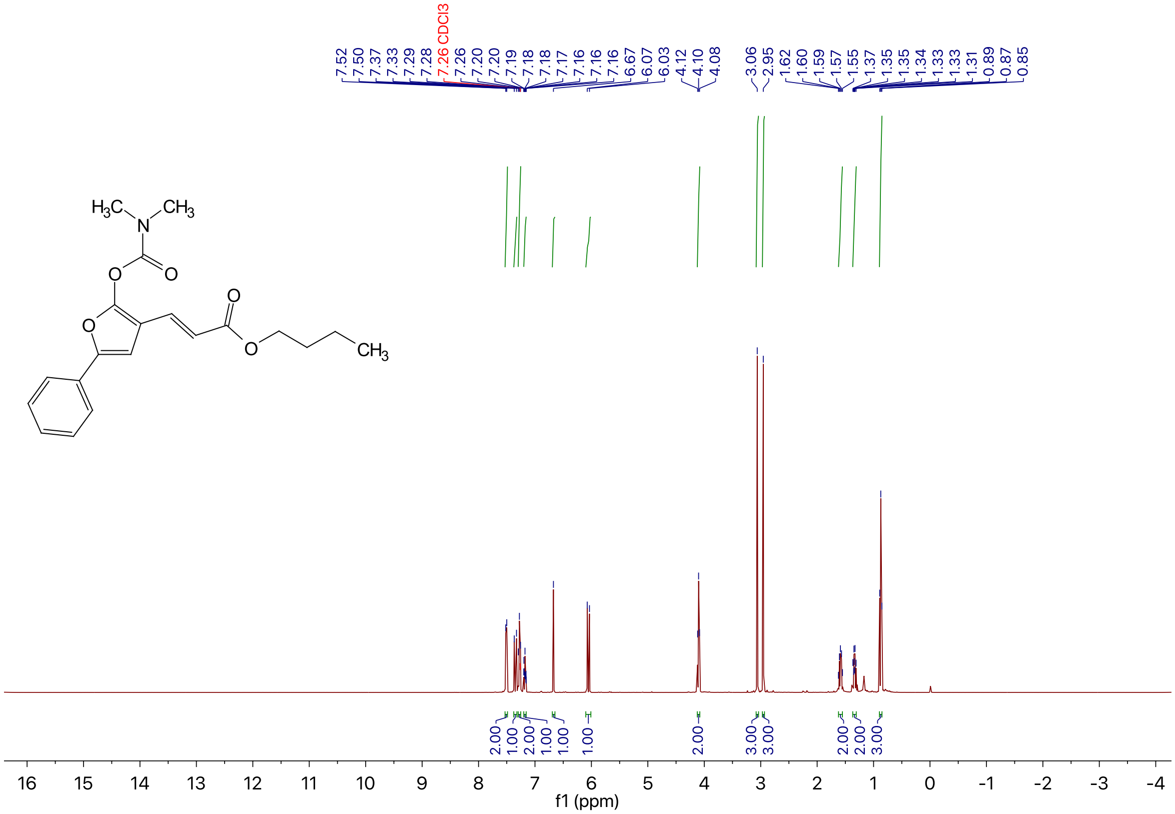

^{13}C NMR spectrum of butyl (*E*)-3-(2-((dimethylcarbamoyl)oxy)-5-phenylfuran-3-yl)acrylate (2g)

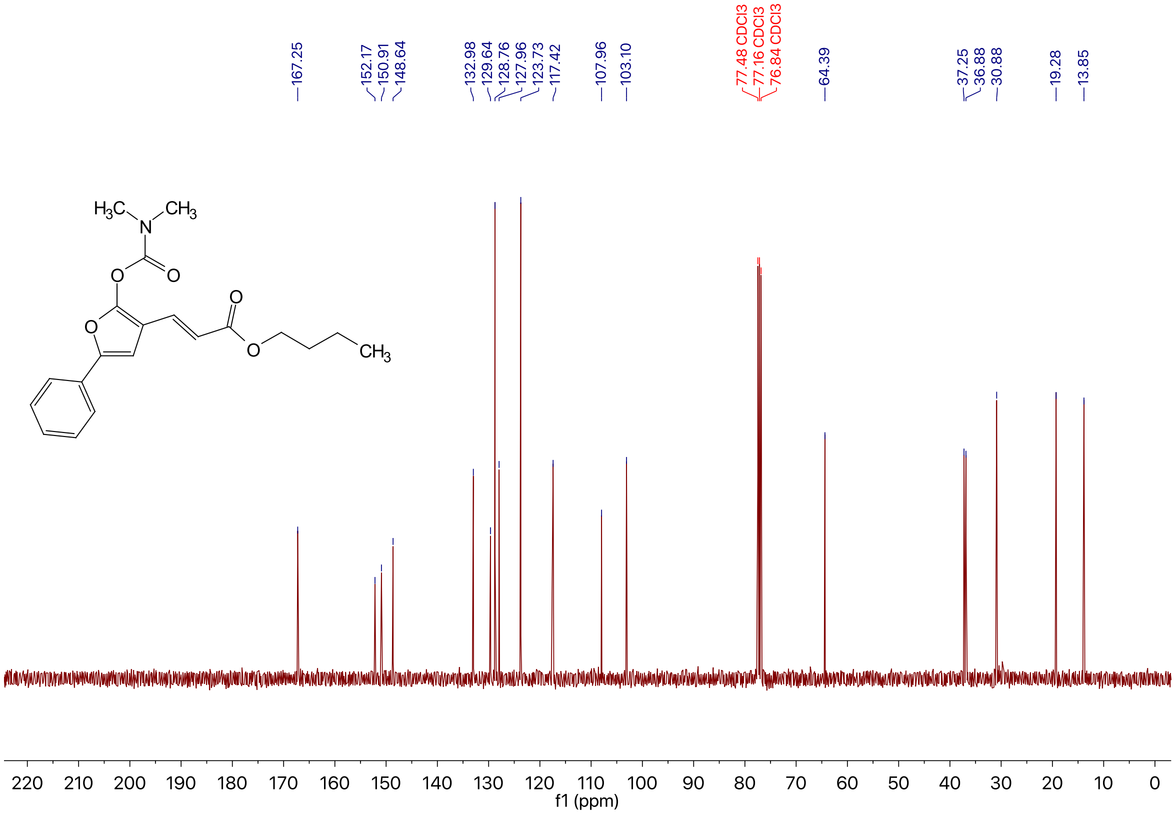

^{1}H NMR spectrum of butyl (*E*)-3-(2-((dimethylcarbamoyl)oxy)-5-(*p*-tolyl)furan-3-yl)acrylate (2h)

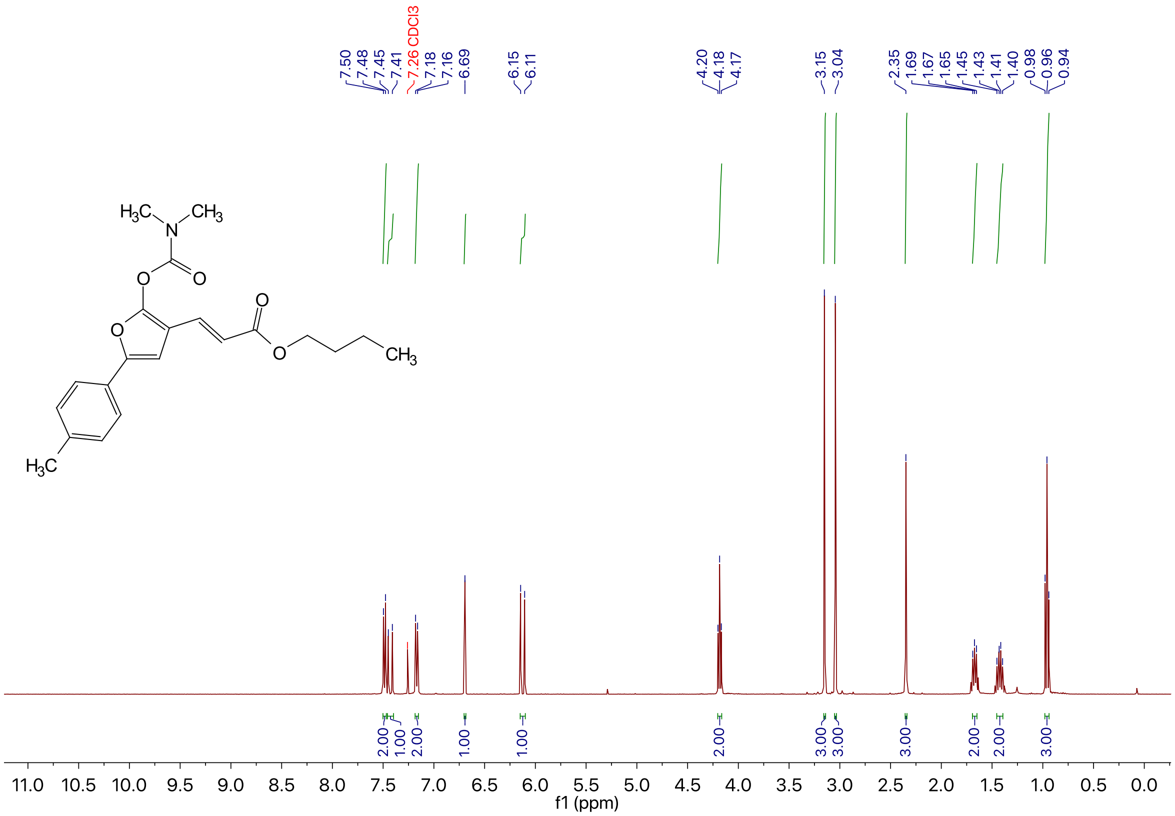

^{13}C NMR spectrum of butyl (*E*)-3-(2-((dimethylcarbamoyl)oxy)-5-(*p*-tolyl)furan-3-yl)acrylate (2h)

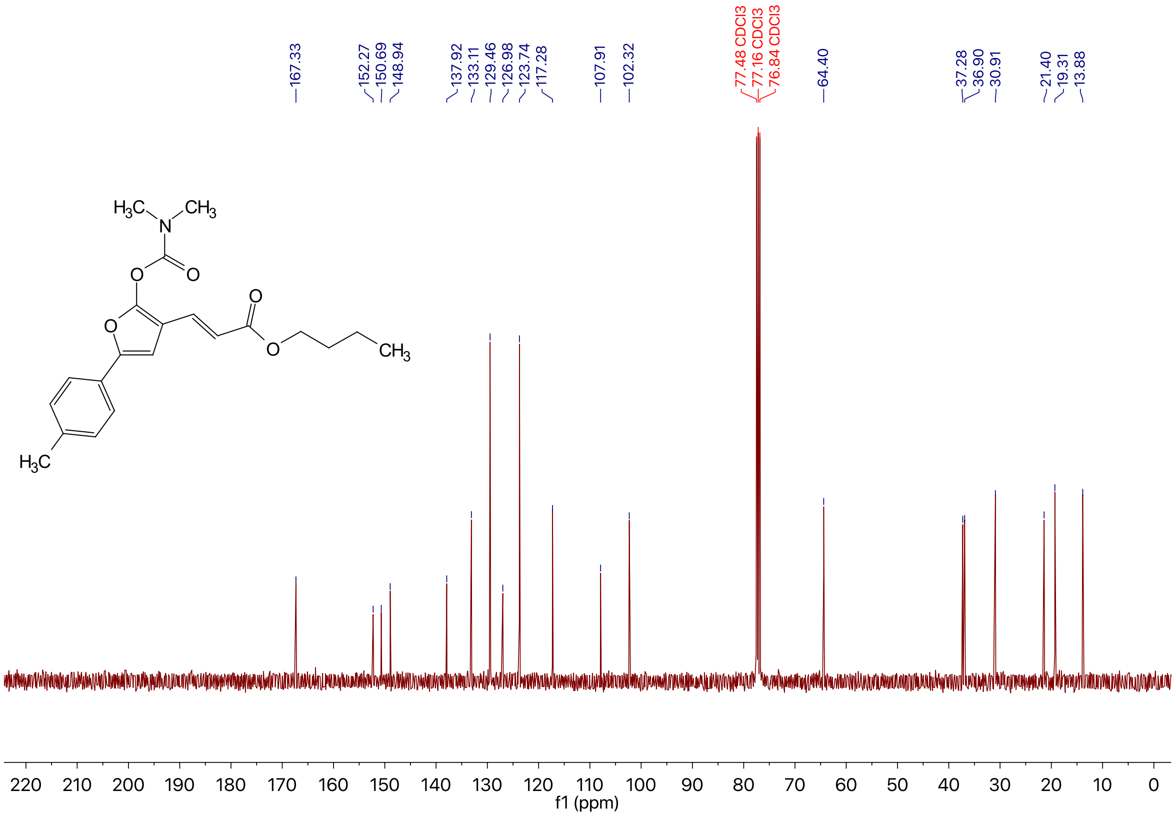

^{1}H NMR spectrum of butyl (*E*)-3-(2-((dimethylcarbamoyl)oxy)-5-(4-(trifluoromethyl)phenyl)furan-3-yl)acrylate (2i)

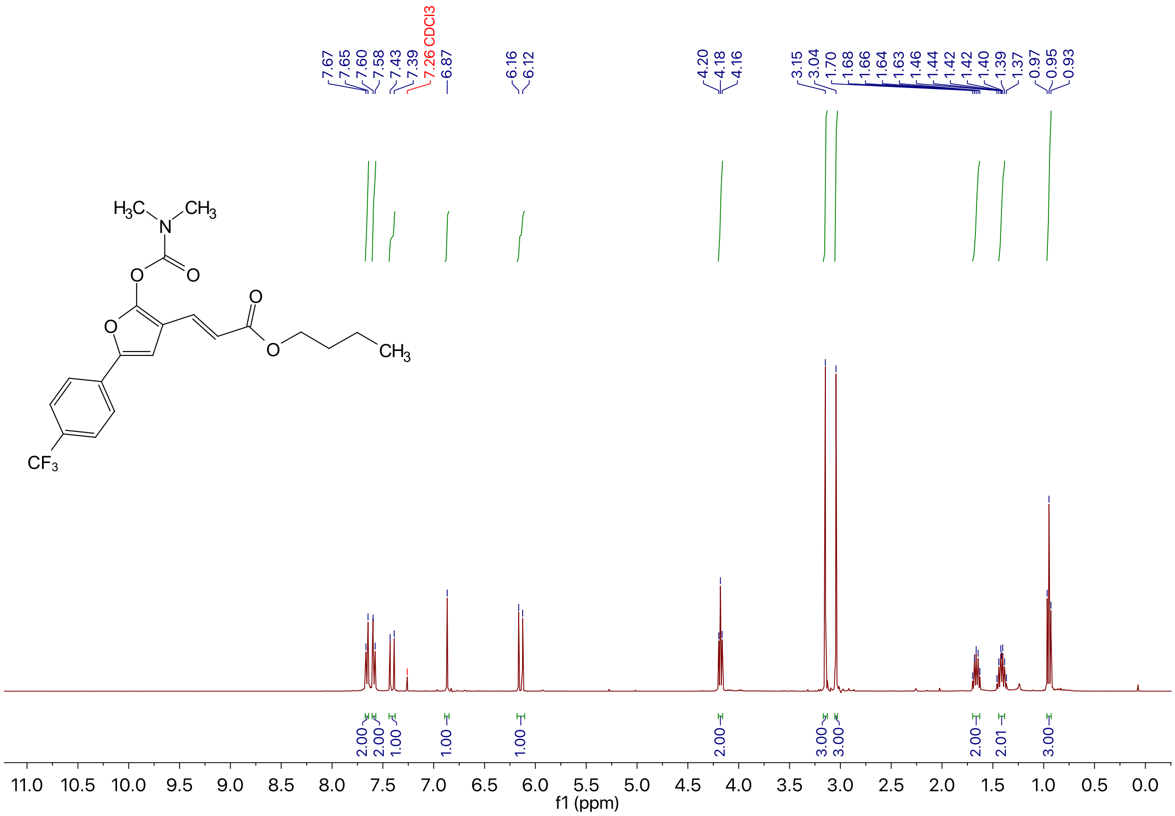

^{13}C NMR spectrum of (butyl (*E*)-3-(2-((dimethylcarbamoyl)oxy)-5-(4-(trifluoromethyl)phenyl)furan-3-yl)acrylate (2i)

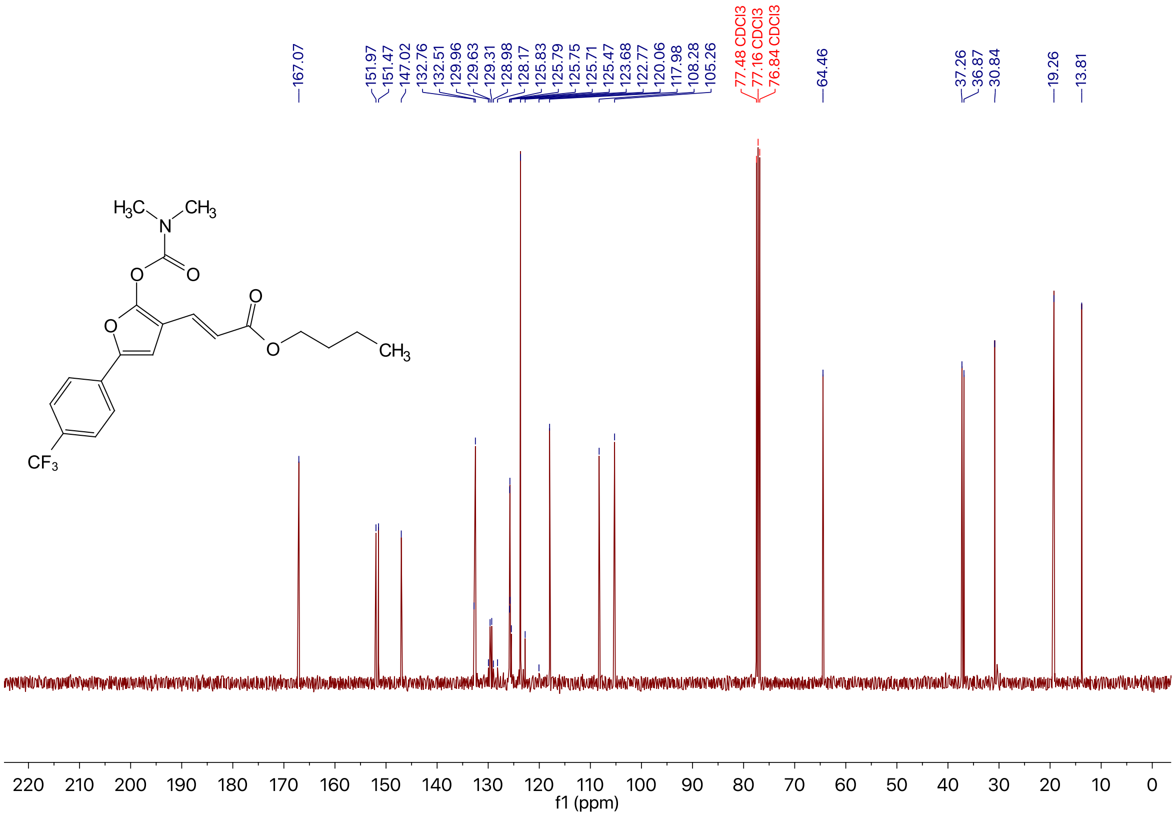

^{1}H NMR spectrum of (butyl (*E*)-3-(5-cyclohexyl-2-((dimethylcarbamoyl)oxy)furan-3-yl)acrylate (2j)

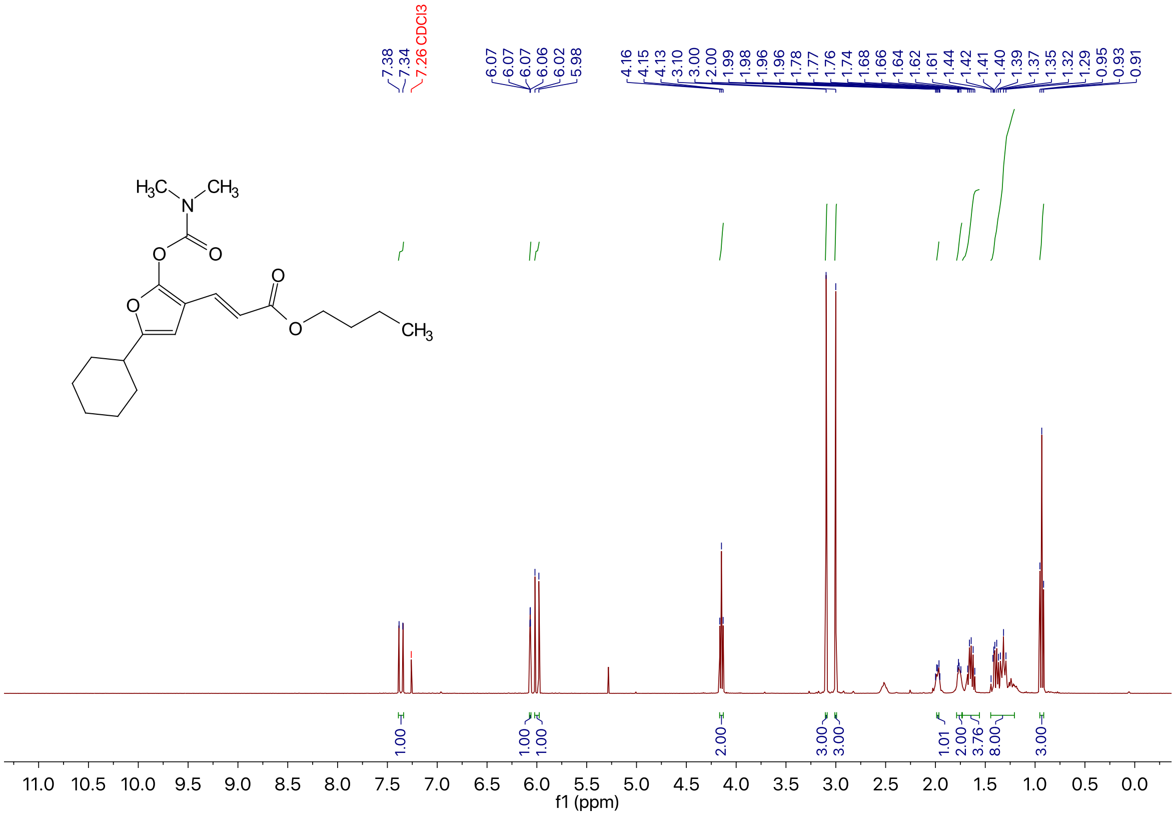

^{13}C NMR spectrum of butyl (*E*)-3-(5-cyclohexyl-2-((dimethylcarbamoyl)oxy)furan-3-yl)acrylate (2j)

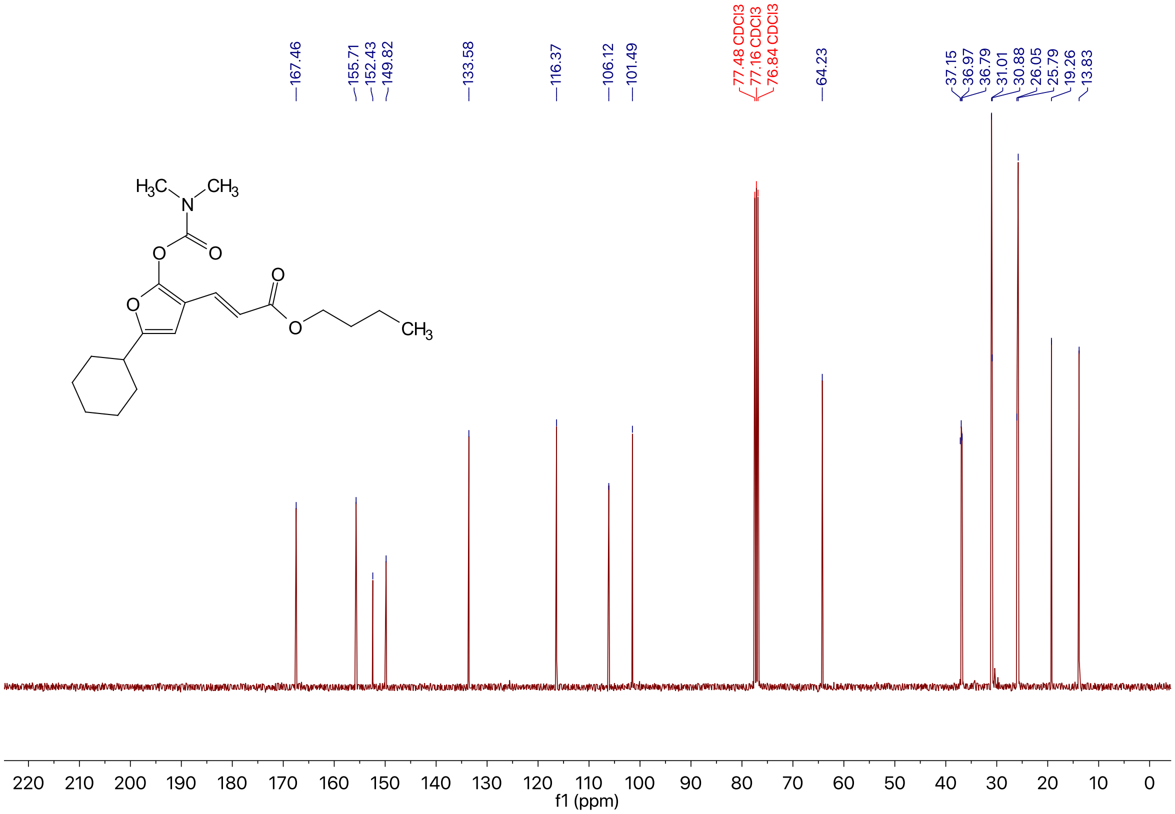

^{1}H NMR spectrum of butyl (*E*)-3-(5-cyclopropyl-2-((dimethylcarbamoyl)oxy)furan-3-yl)acrylate (2k)

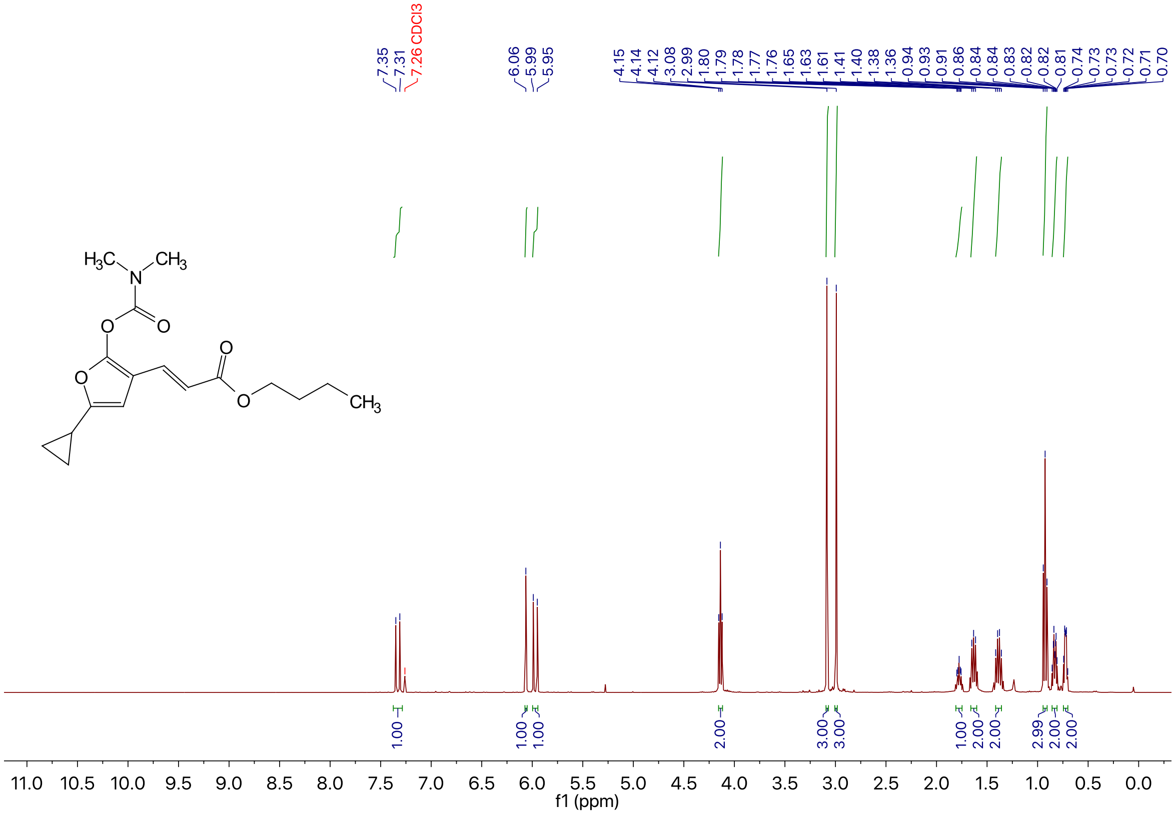

^{13}C NMR spectrum of butyl (*E*)-3-(5-cyclopropyl-2-((dimethylcarbamoyl)oxy)furan-3-yl)acrylate (2k)

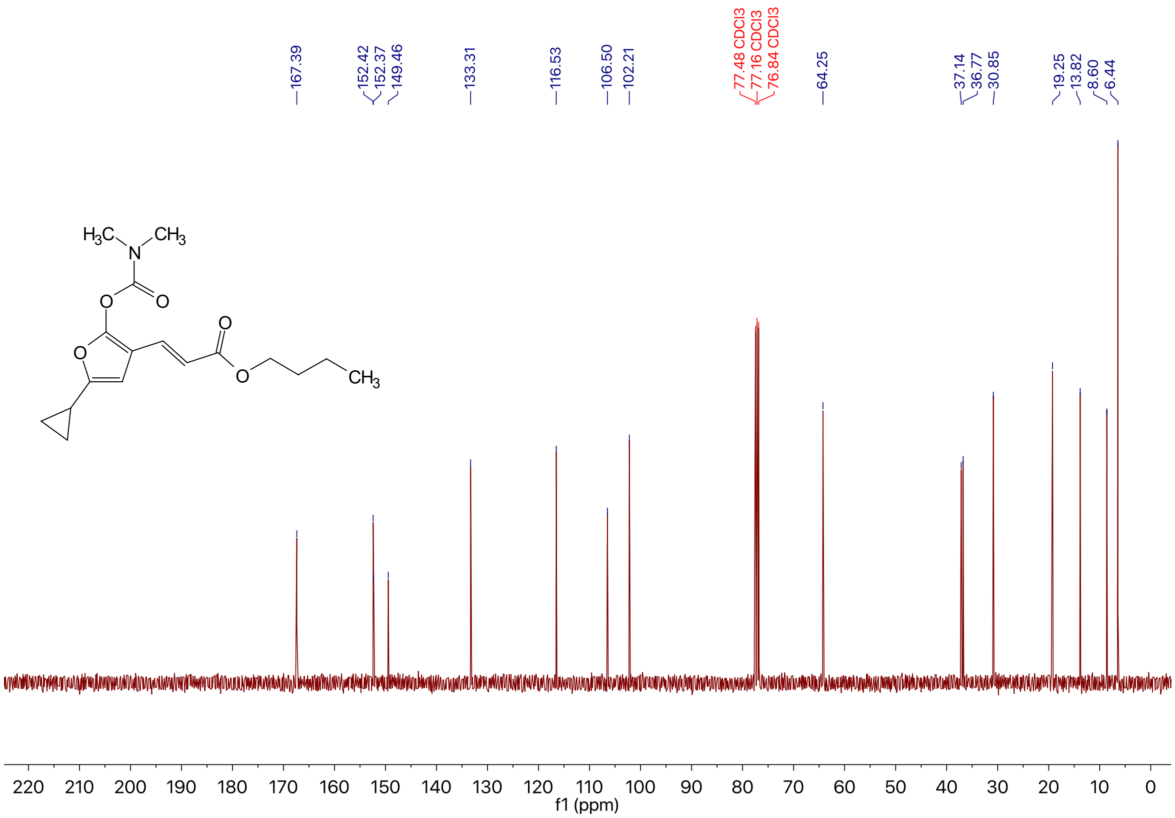

^{1}H NMR spectrum of (butyl (*E*)-3-(2-((dimethylcarbamoyl)oxy)-5-phenethylfuran-3-yl)acrylate (2l)

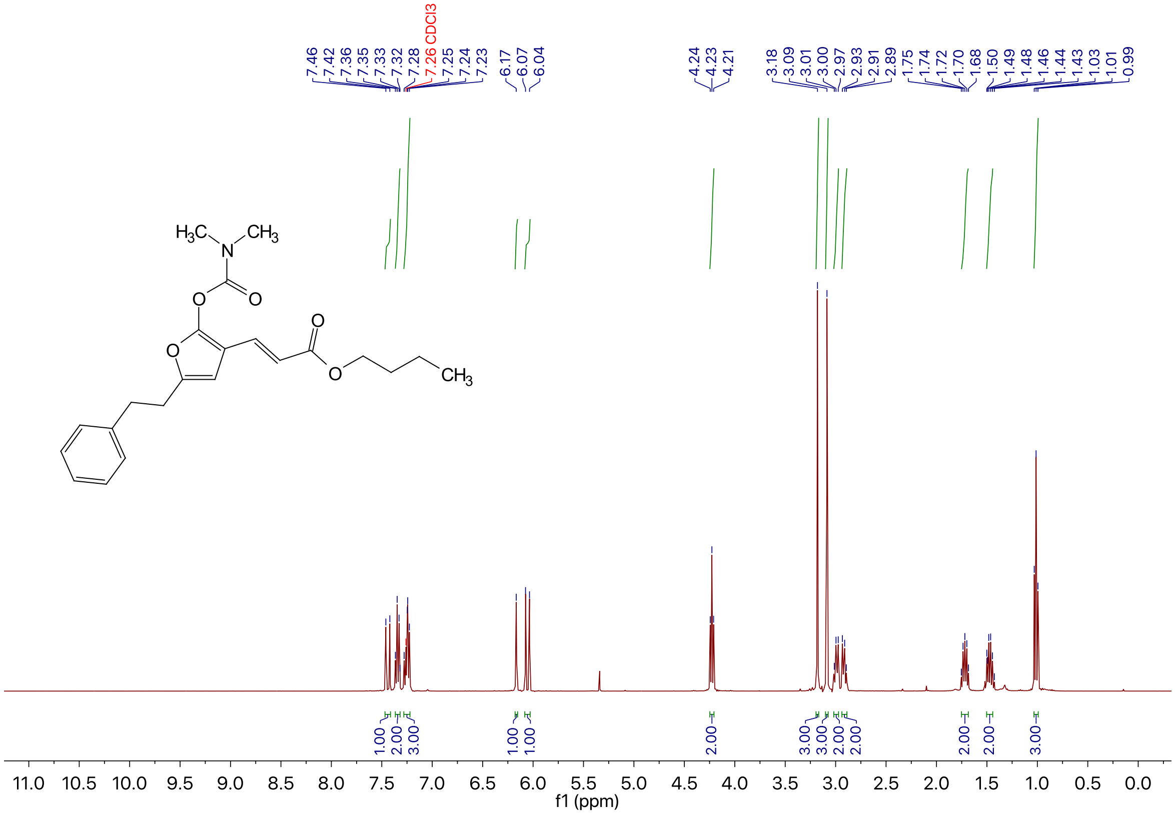

^{13}C NMR spectrum of butyl (*E*)-3-(2-((dimethylcarbamoyl)oxy)-5-phenethylfuran-3-yl)acrylate (2l)

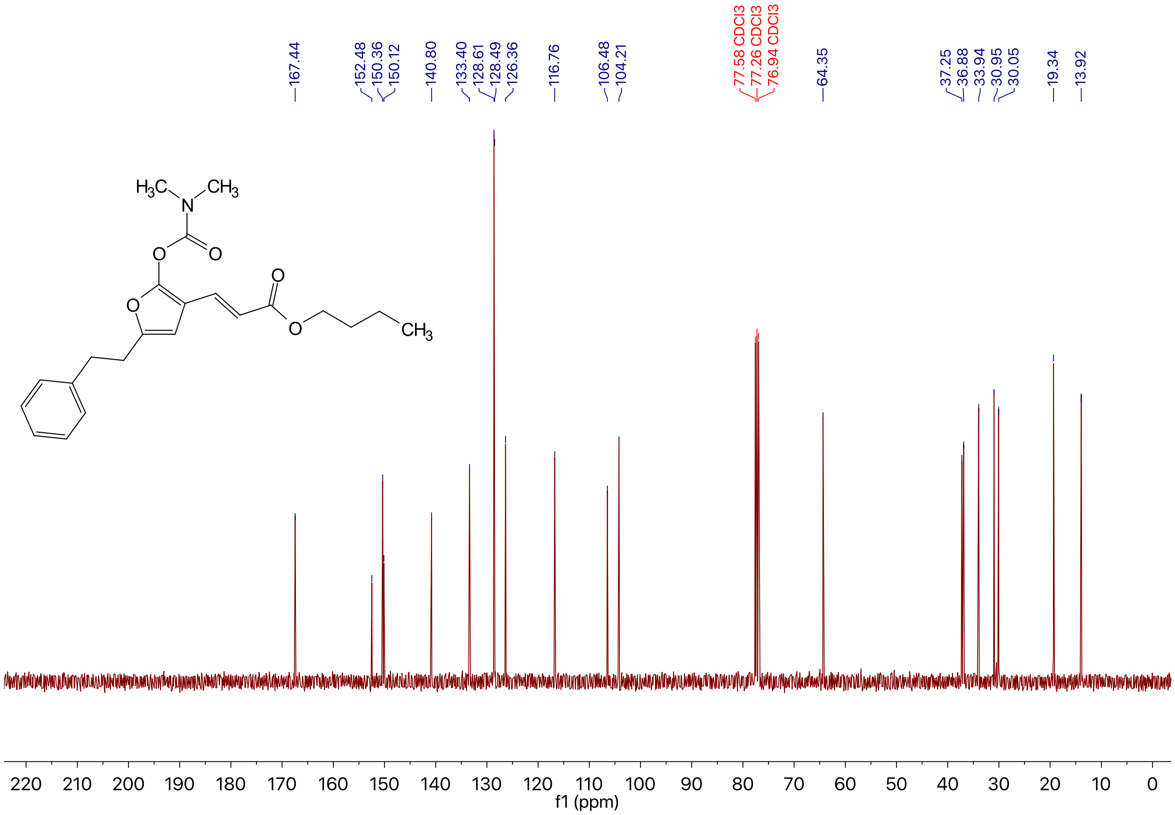

^{1}H NMR spectrum of butyl (*E*)-3-(2-((dimethylcarbamoyl)oxy)-4-methylfuran-3-yl)acrylate (2m)

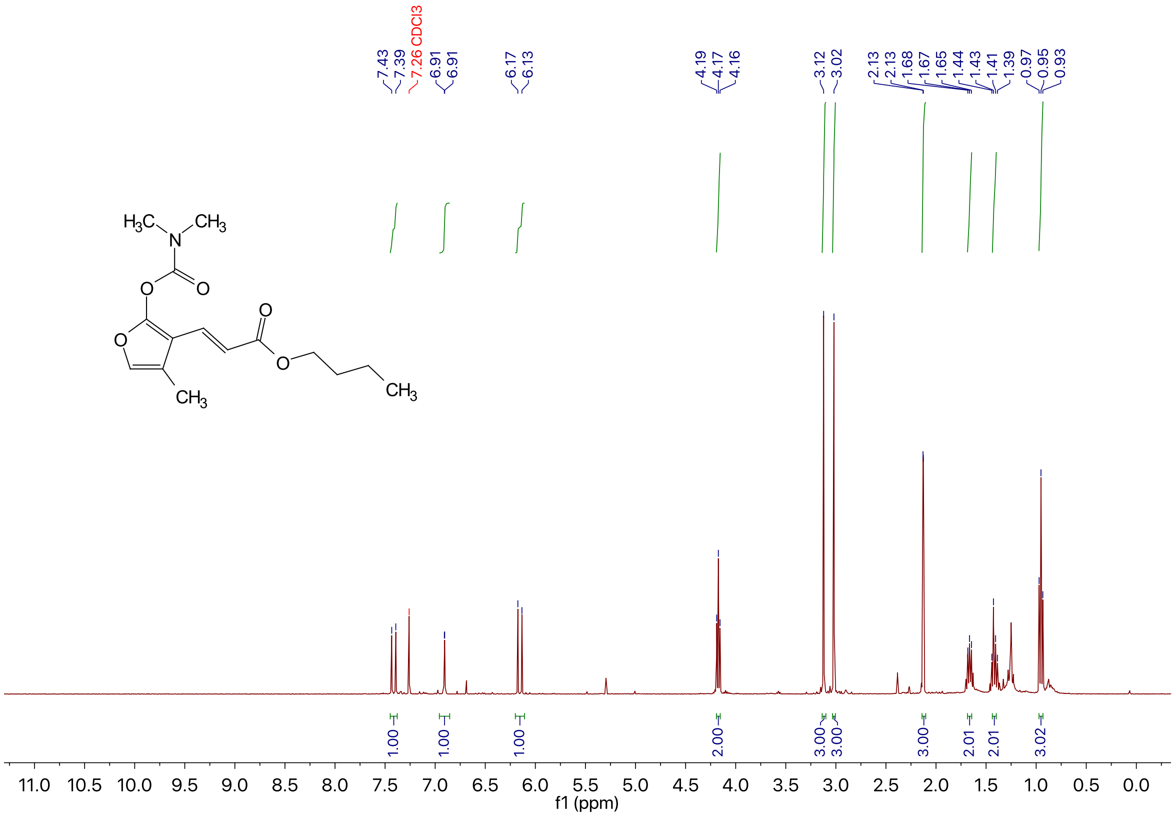

^{13}C NMR spectrum of (butyl (*E*)-3-(2-((dimethylcarbamoyl)oxy)-4-methylfuran-3-yl)acrylate (2m)

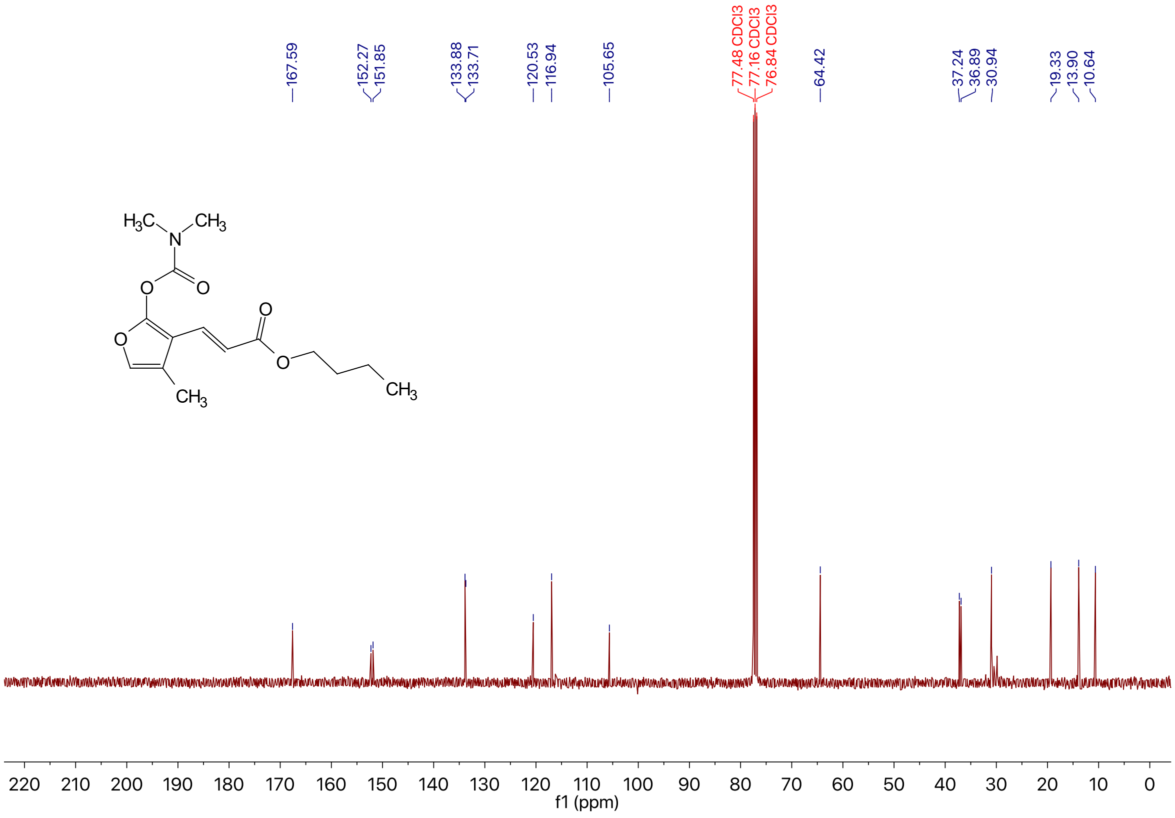

^{1}H NMR spectrum of butyl (*E*)-3-(2-((dimethylcarbamoyl)oxy)-5-(*o*-tolyl)furan-3-yl)acrylate (2n)

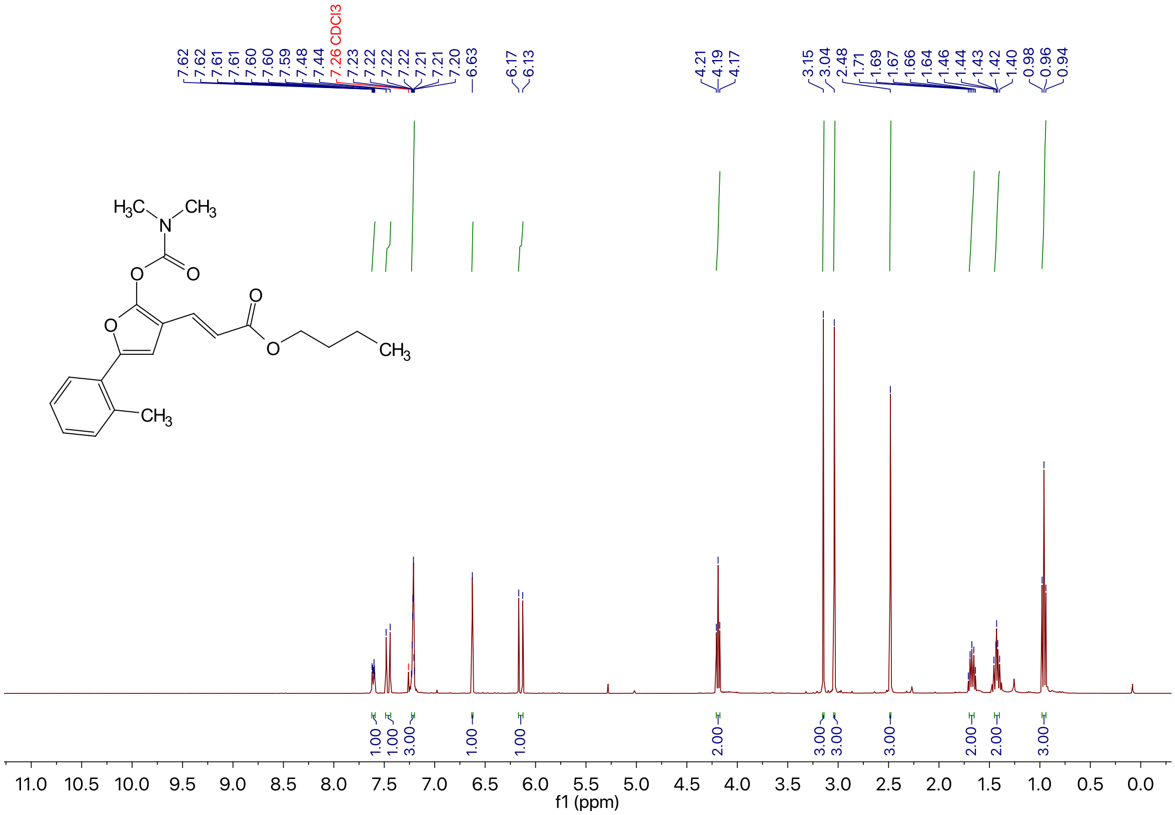

^{13}C NMR spectrum of butyl (*E*)-3-(2-((dimethylcarbamoyl)oxy)-5-(*o*-tolyl)furan-3-yl)acrylate (2n)

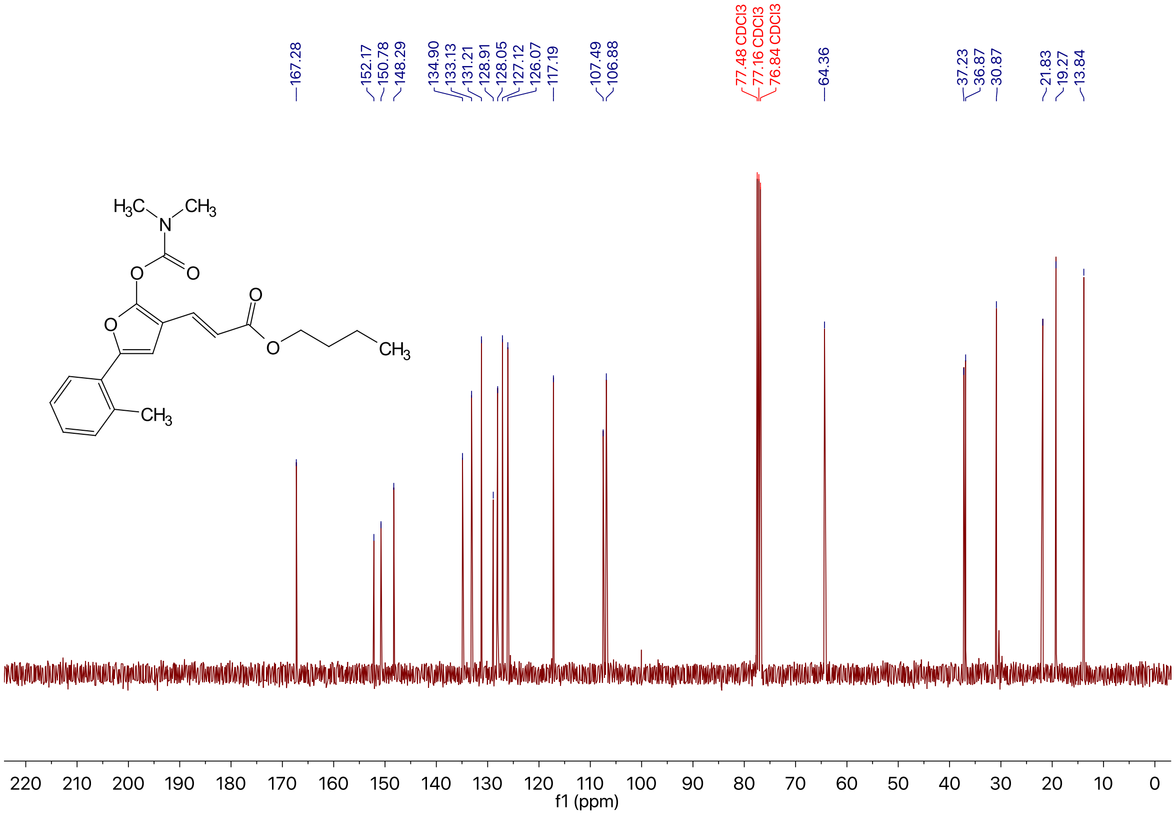

^{1}H NMR spectrum of butyl (*E*)-3-(5-(4-bromophenyl)-2-((dimethylcarbamoyl)oxy)furan-3-yl)acrylate (2o)

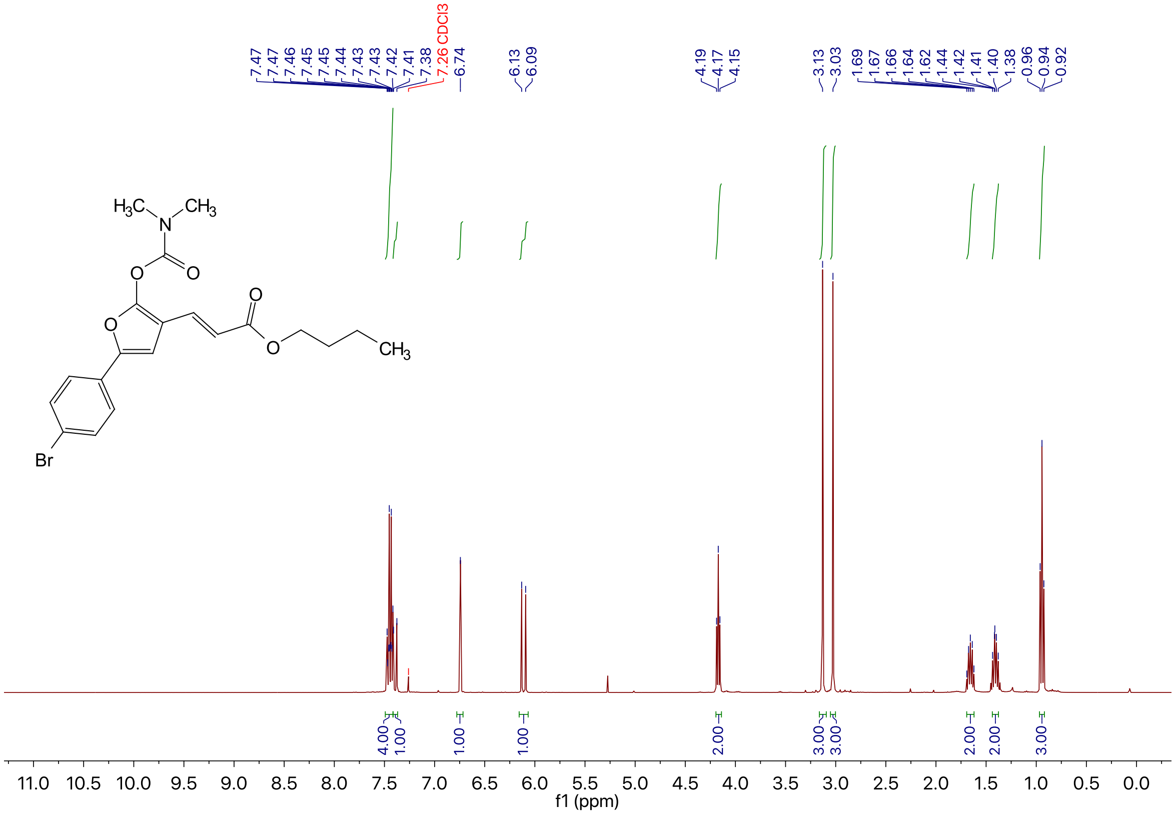

^{13}C NMR spectrum of butyl (*E*)-3-(5-(4-bromophenyl)-2-((dimethylcarbamoyl)oxy)furan-3-yl)acrylate (2o)

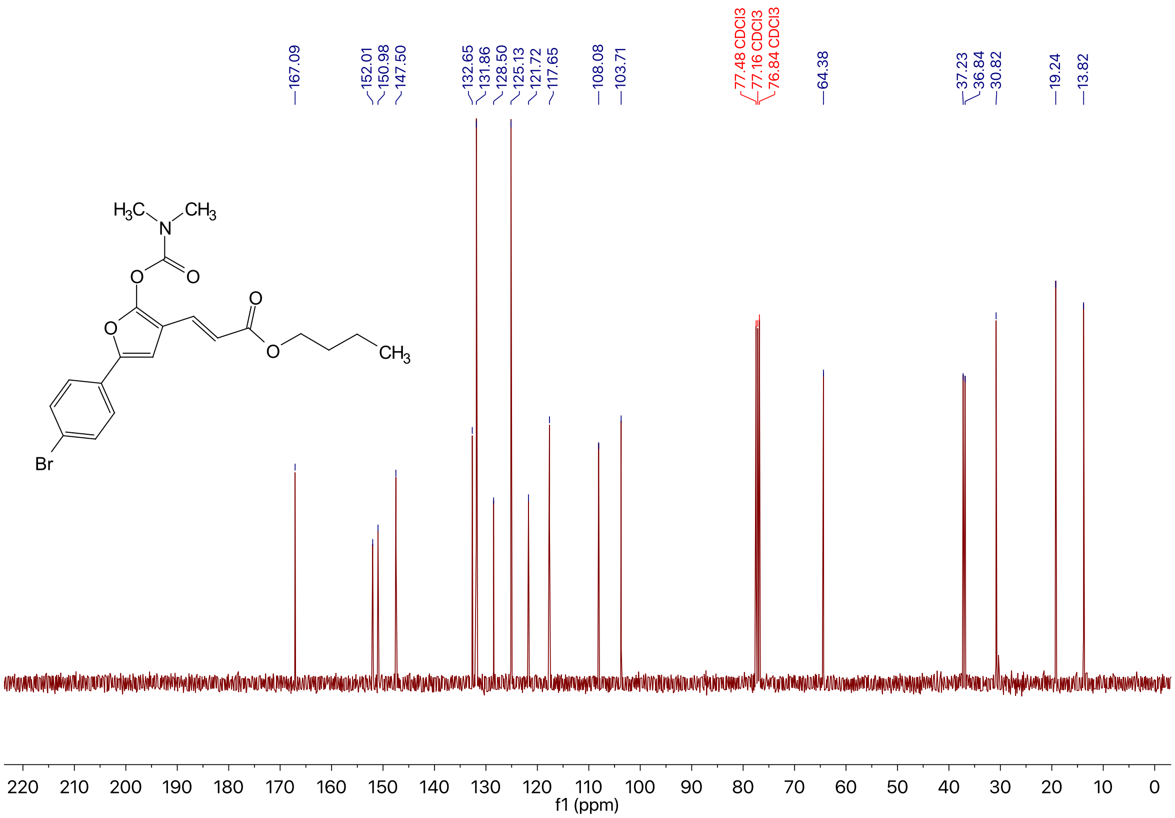

^{1}H NMR spectrum of butyl (*E*)-3-(2-((dimethylcarbamoyl)oxy)-5-(4-nitrophenyl)furan-3-yl)acrylate (2p)

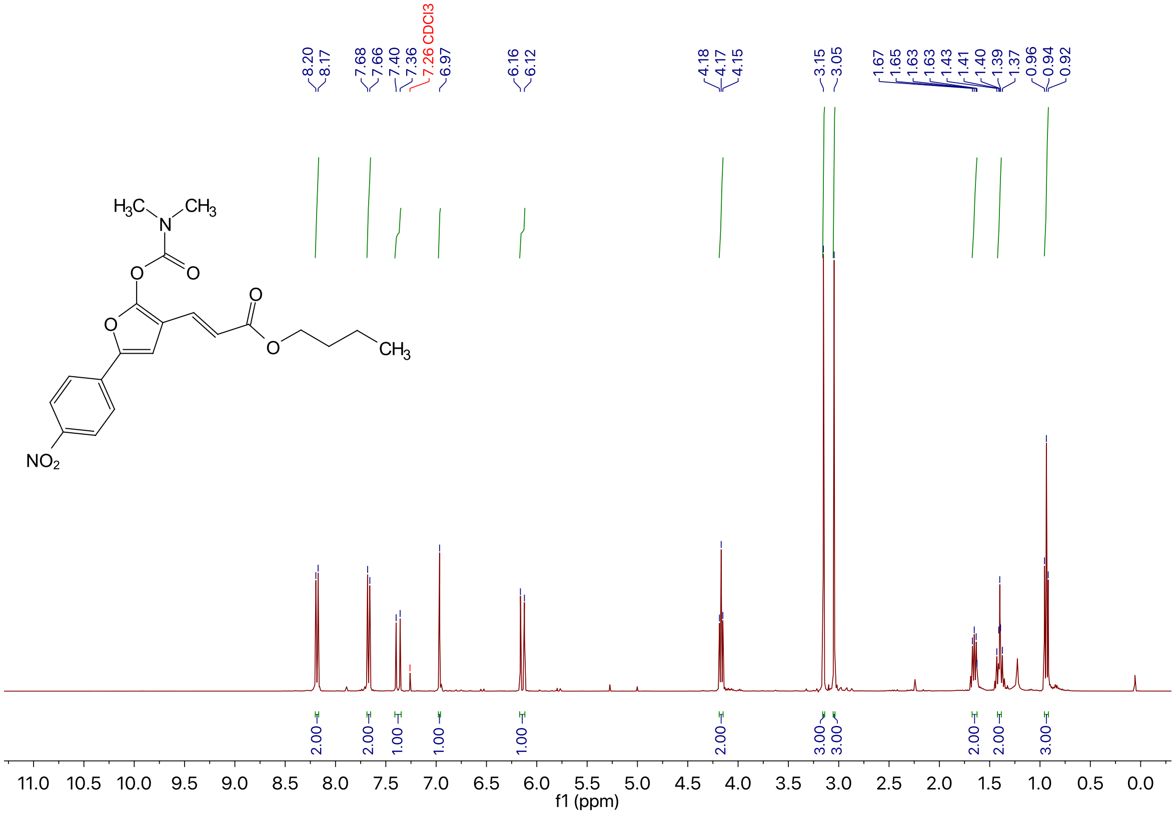

^{13}C NMR spectrum of butyl (*E*)-3-(2-((dimethylcarbamoyl)oxy)-5-(4-nitrophenyl)furan-3-yl)acrylate (2p)

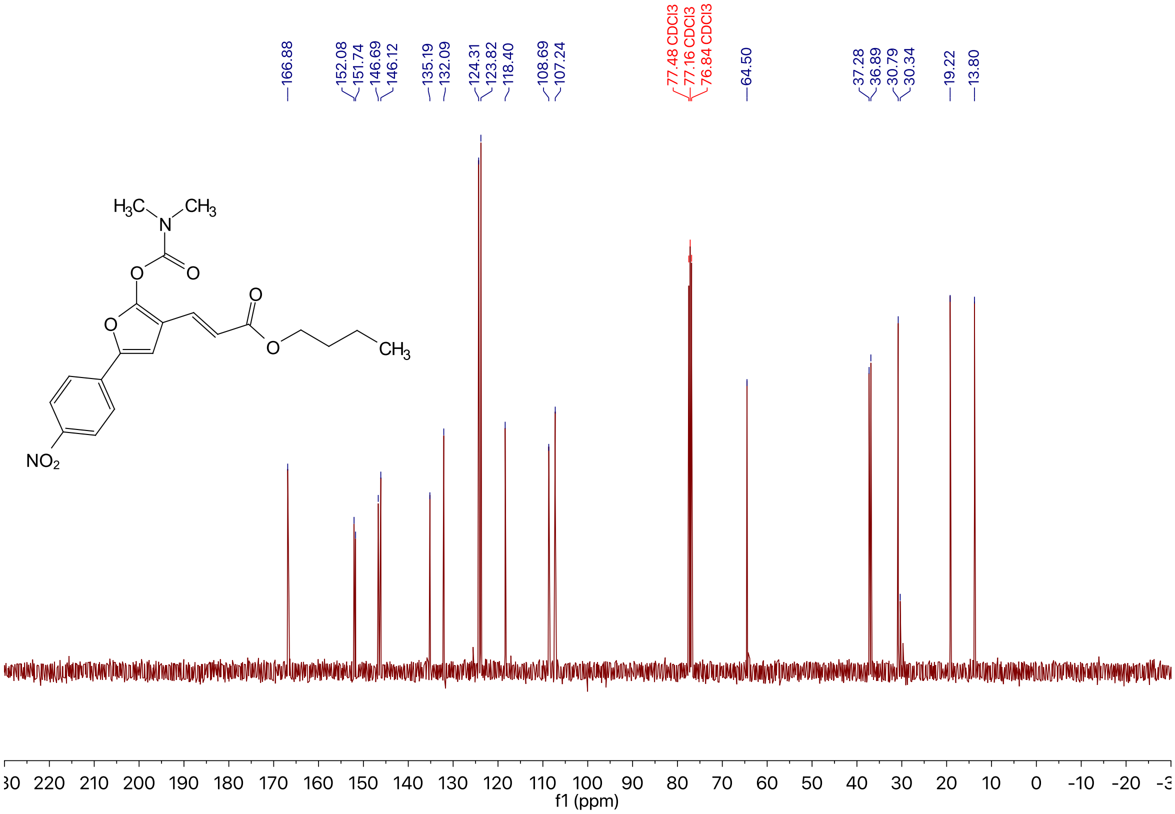

^{1}H NMR spectrum of methyl (*E*)-4-(4-(3-butoxy-3-oxoprop-1-en-1-yl)-5-((dimethylcarbamoyl)oxy)furan 2yl)benzoate (2q)

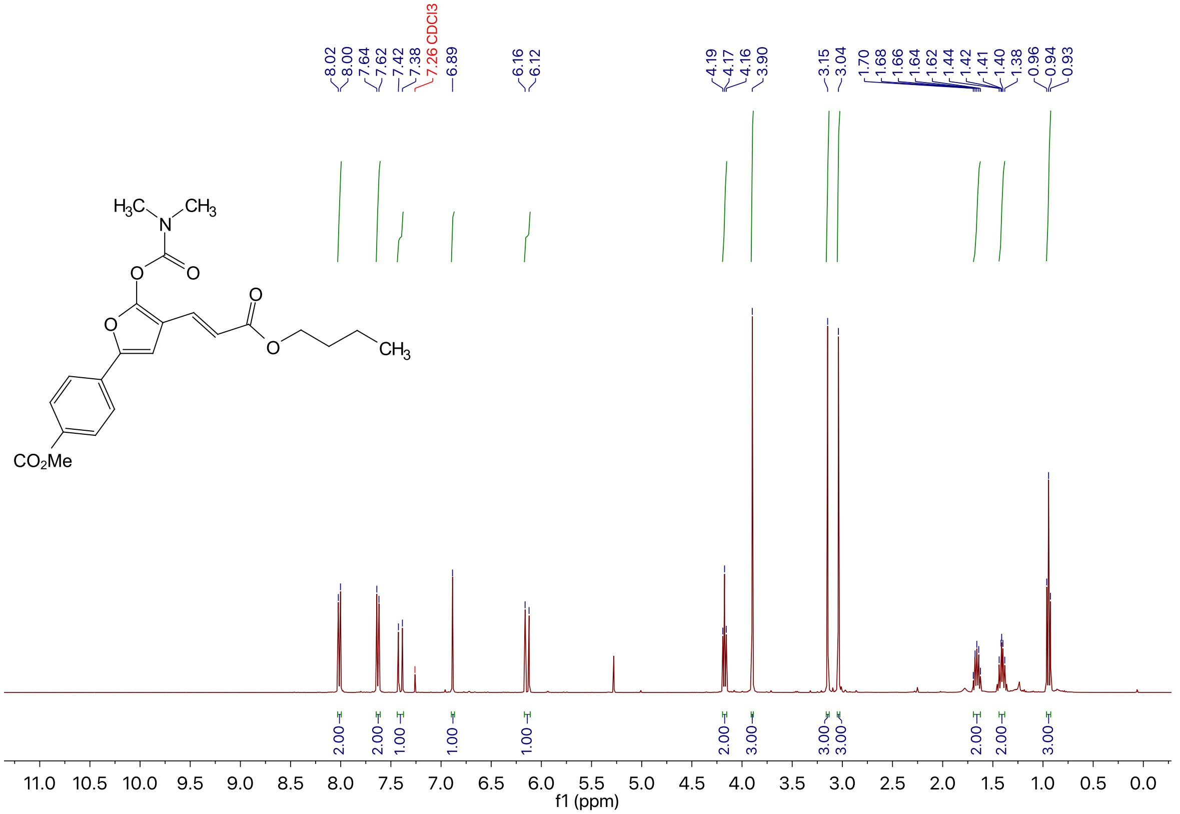

^{13}C NMR spectrum of methyl (*E*)-4-(4-(3-butoxy-3-oxoprop-1-en-1-yl)-5-((dimethylcarbamoyl)oxy)furan 2yl)benzoate (2q)

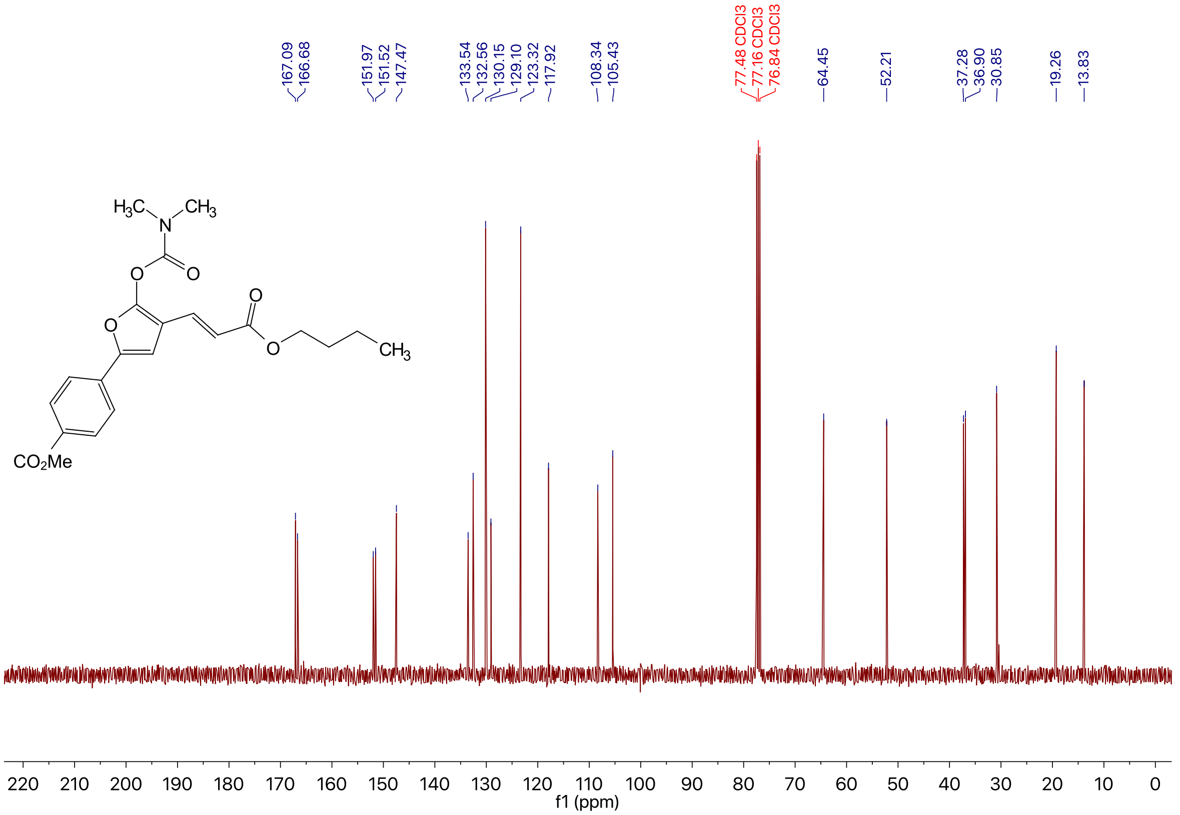

^{1}H NMR spectrum of butyl (*E*)-3-(2-((dimethylcarbamoyl)oxy)-5-(thiophen-2-yl)furan-3-yl)acrylate (2r)

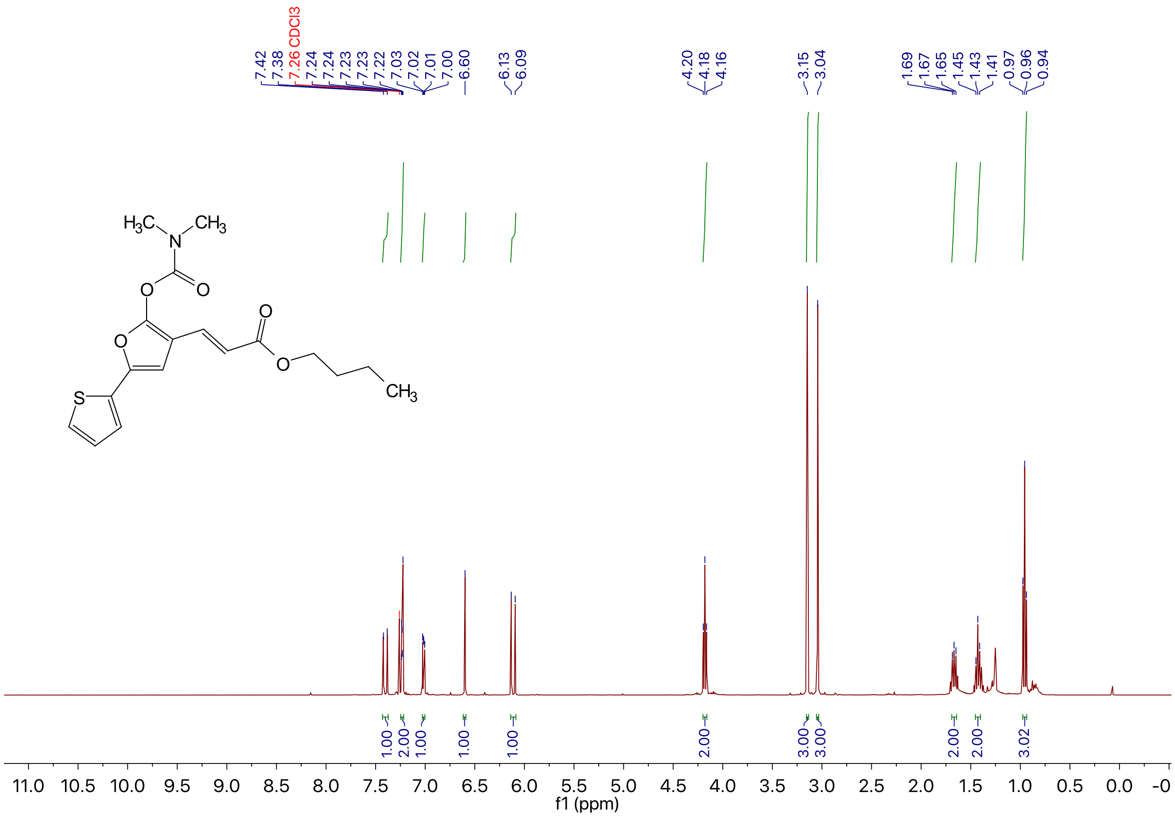

^{13}C NMR spectrum of butyl (*E*)-3-(2-((dimethylcarbamoyl)oxy)-5-(thiophen-2-yl)furan-3-yl)acrylate (2r)

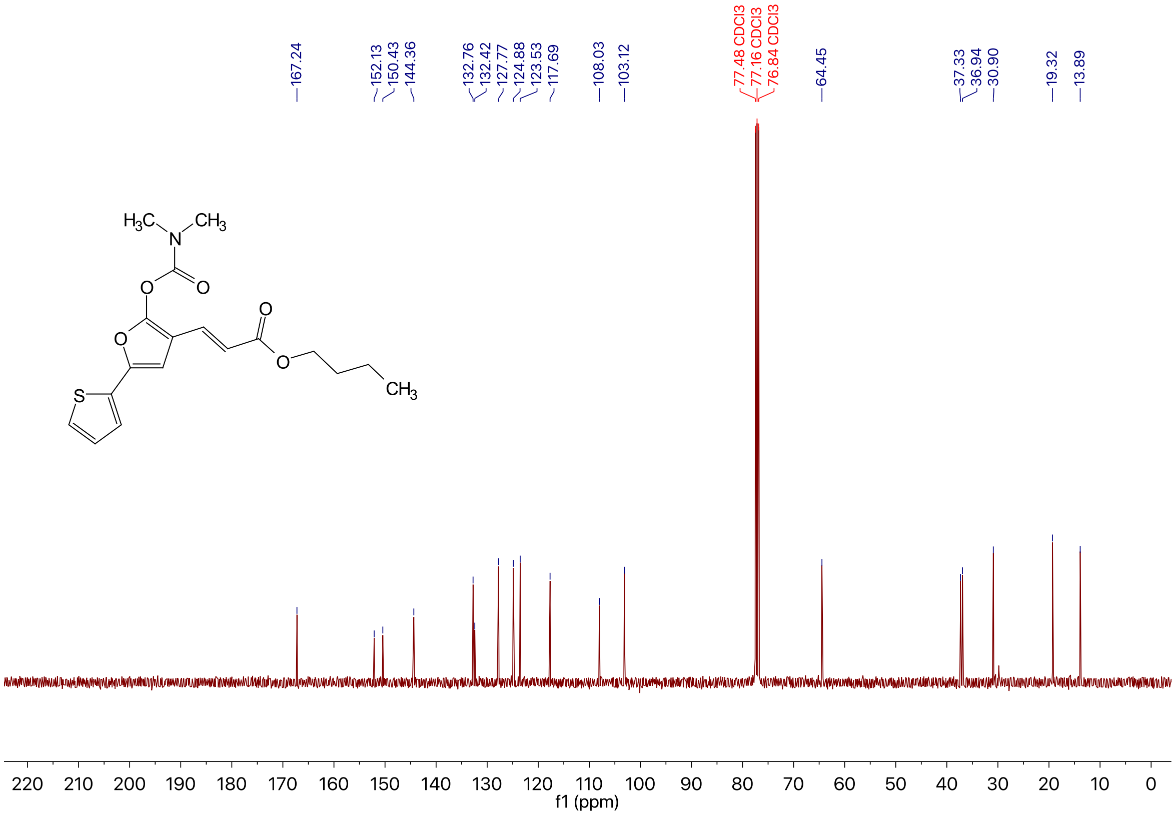

^{1}H NMR spectrum of butyl (*E*)-3-(2-((dimethylcarbamoyl)oxy)thiophen-3-yl)acrylate (2t)

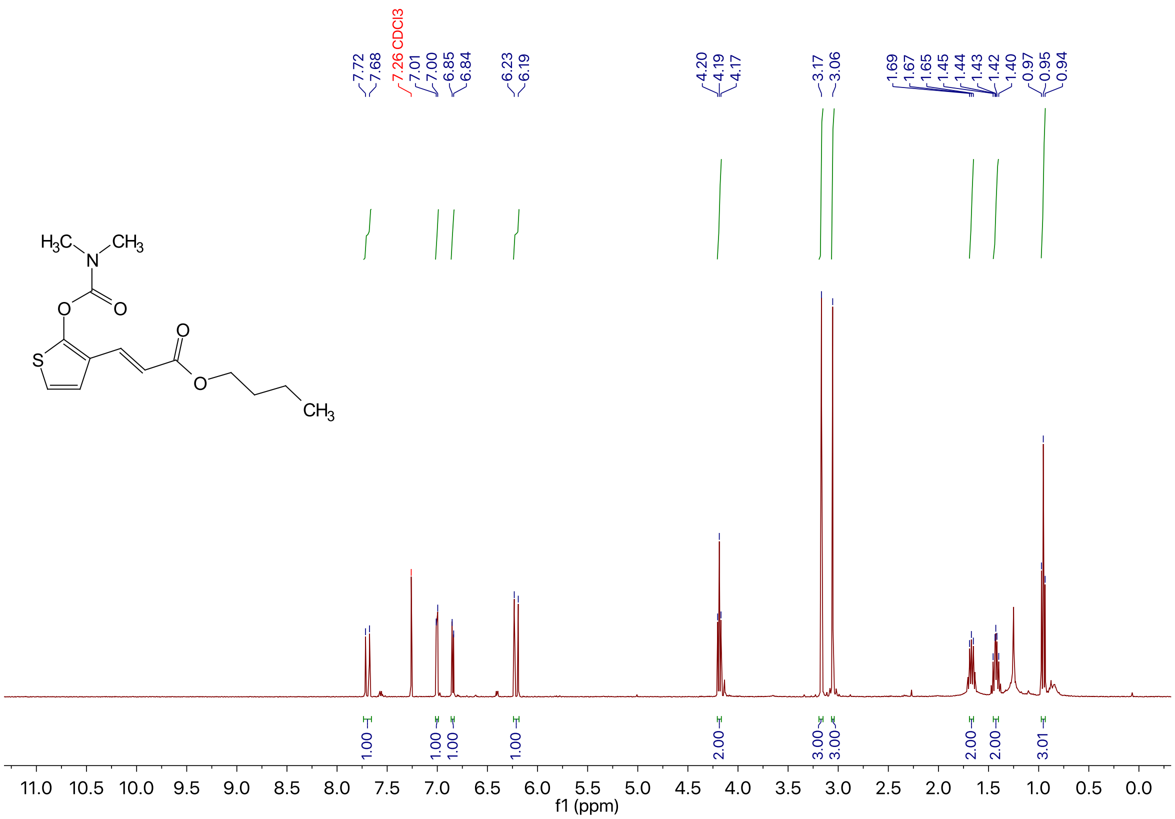

^{13}C NMR spectrum of butyl (*E*)-3-(2-((dimethylcarbamoyl)oxy)thiophen-3-yl)acrylate (2t)

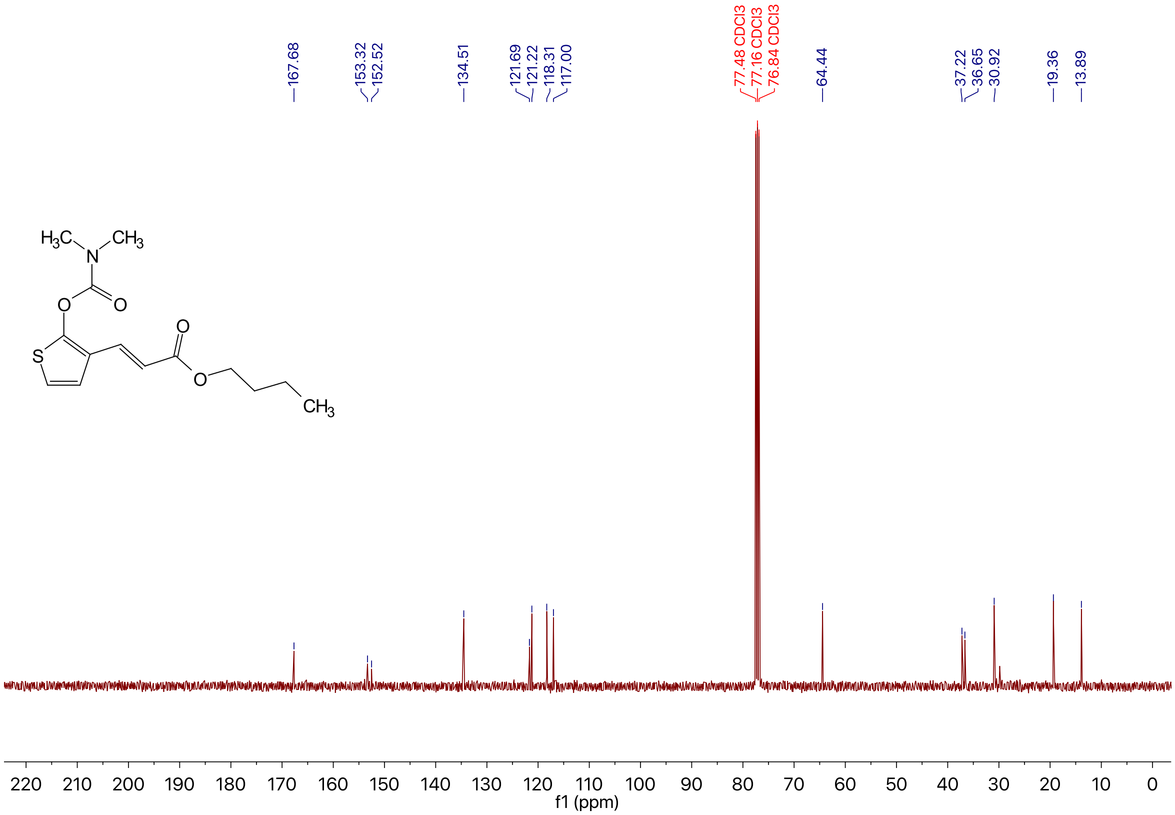

^{1}H NMR spectrum of benzyl (*E*)-3-(1-benzyl-2-((dimethylcarbamoyl)oxy)-1*H*-pyrrol-3-yl)acrylate (2u)

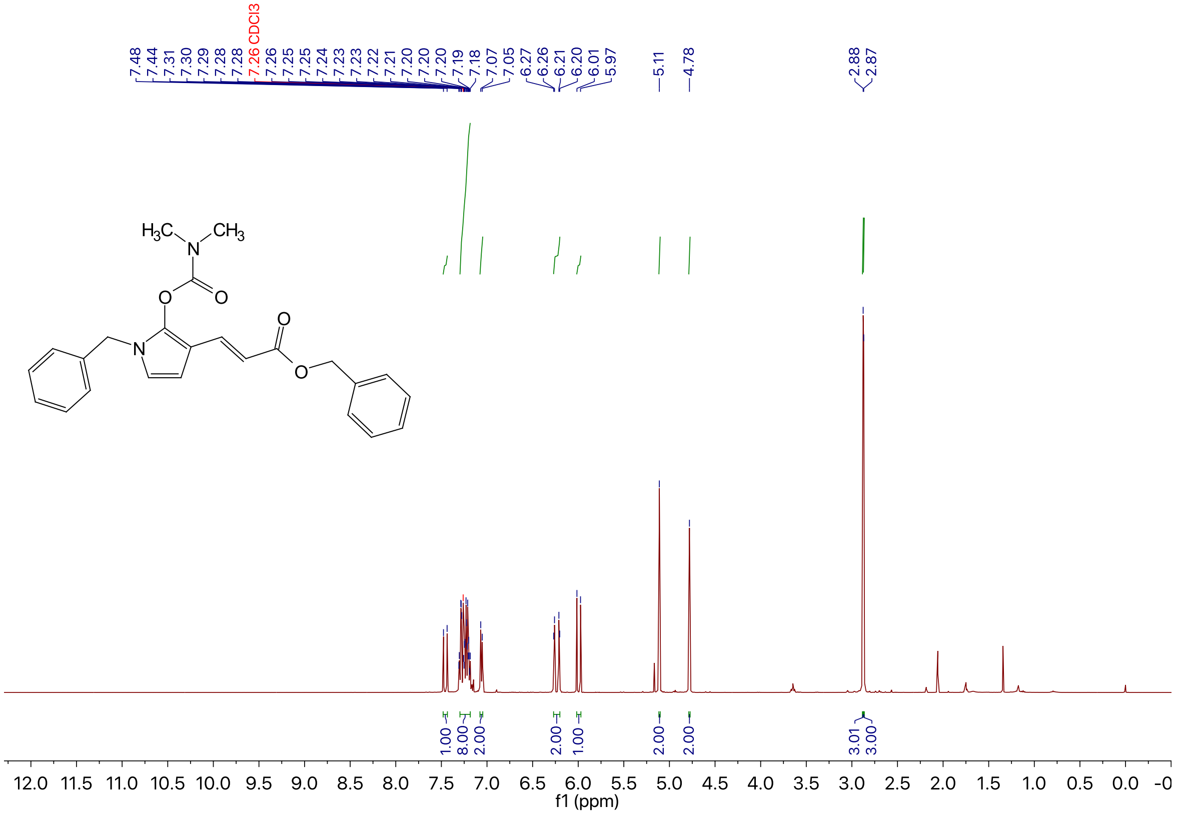

^{13}C NMR spectrum of benzyl (*E*)-3-(1-benzyl-2-((dimethylcarbamoyl)oxy)-1*H*-pyrrol-3-yl)acrylate (2u)

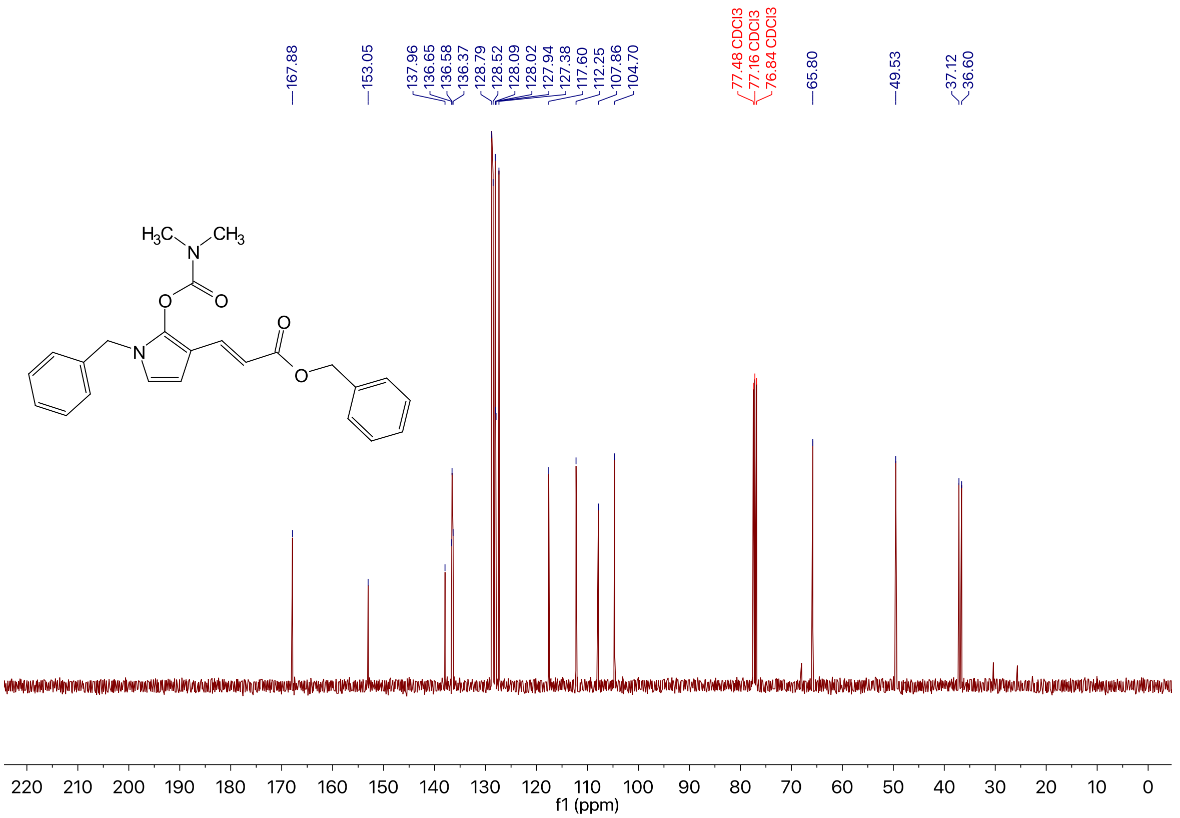

^{1}H NMR spectrum of methyl (*E*)-3-(1-benzyl-2-((dimethylcarbamoyl)oxy)-1*H*-pyrrol-3-yl)acrylate (2v)

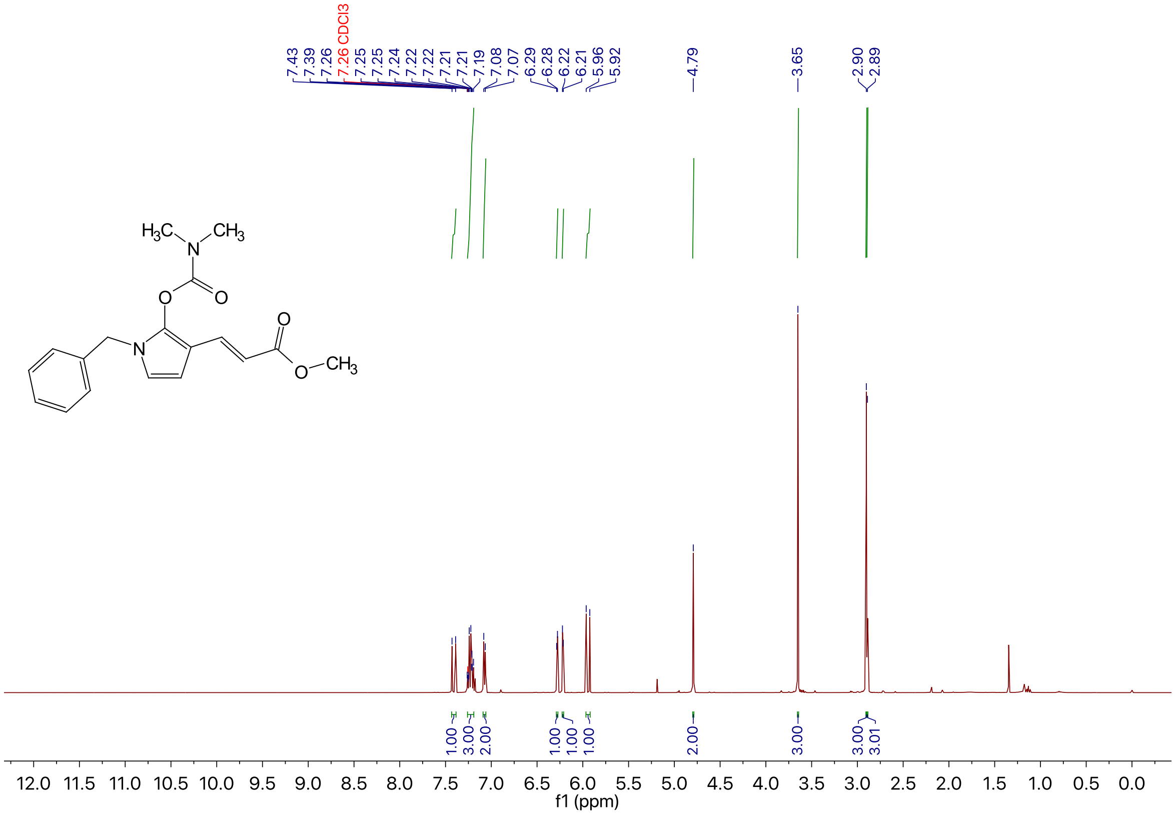

^{13}C NMR spectrum of methyl (*E*)-3-(1-benzyl-2-((dimethylcarbamoyl)oxy)-1*H*-pyrrol-3-yl)acrylate (2v)

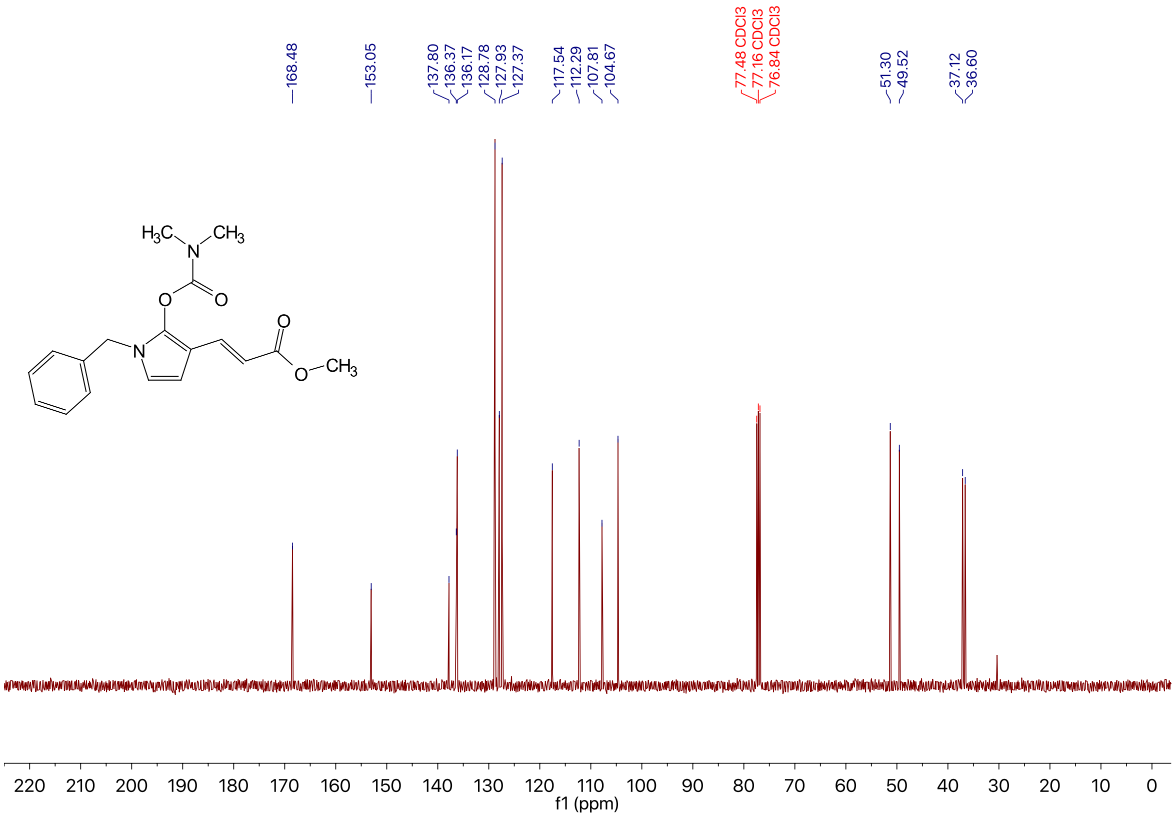

^{1}H NMR spectrum of butyl (*E*)-3-(2-((dimethylcarbamoyl)oxy)-1-tosyl-1*H*-pyrrol-3-yl)acrylate (2w)

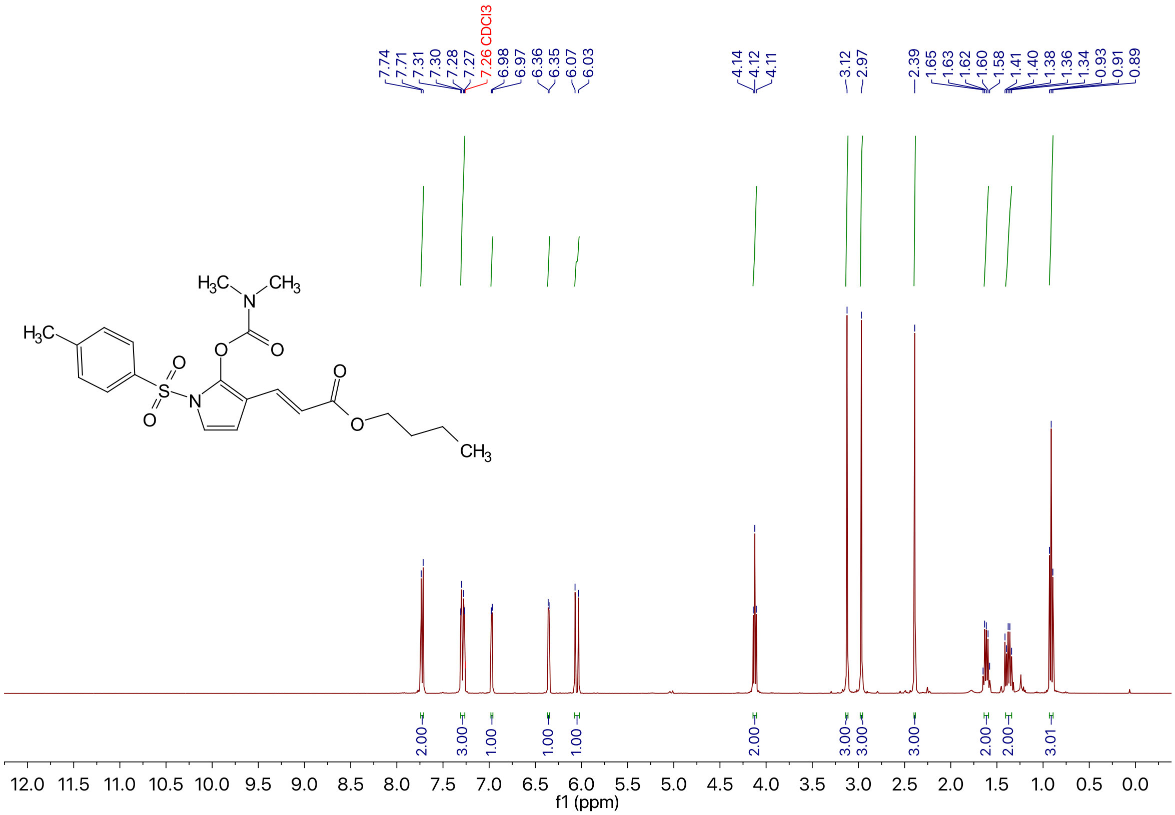

^{13}C NMR spectrum of butyl (*E*)-3-(2-((dimethylcarbamoyl)oxy)-1-tosyl-1*H*-pyrrol-3-yl)acrylate (2w)

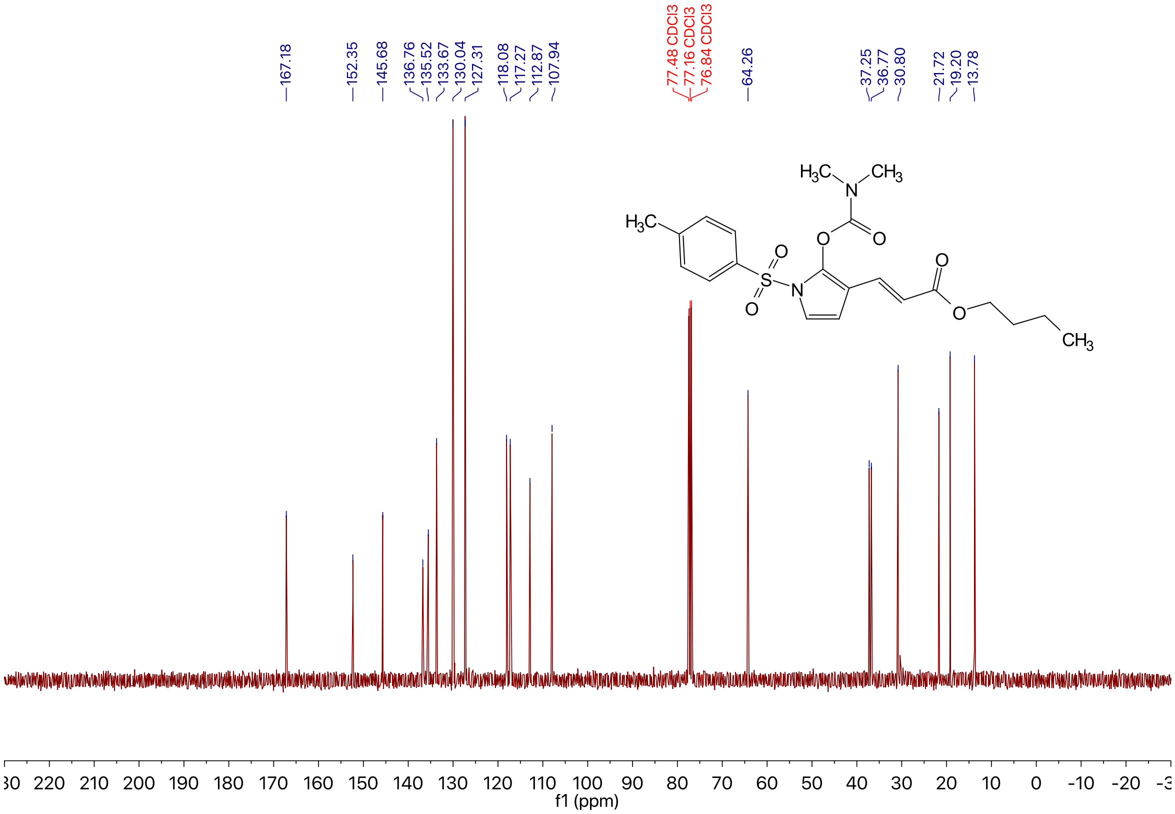

^{1}H NMR spectrum of butyl (*E*)-3-(1-benzyl-2-((dimethylcarbamoyl)oxy)-1*H*-pyrrol-3-yl)acrylate (2x)

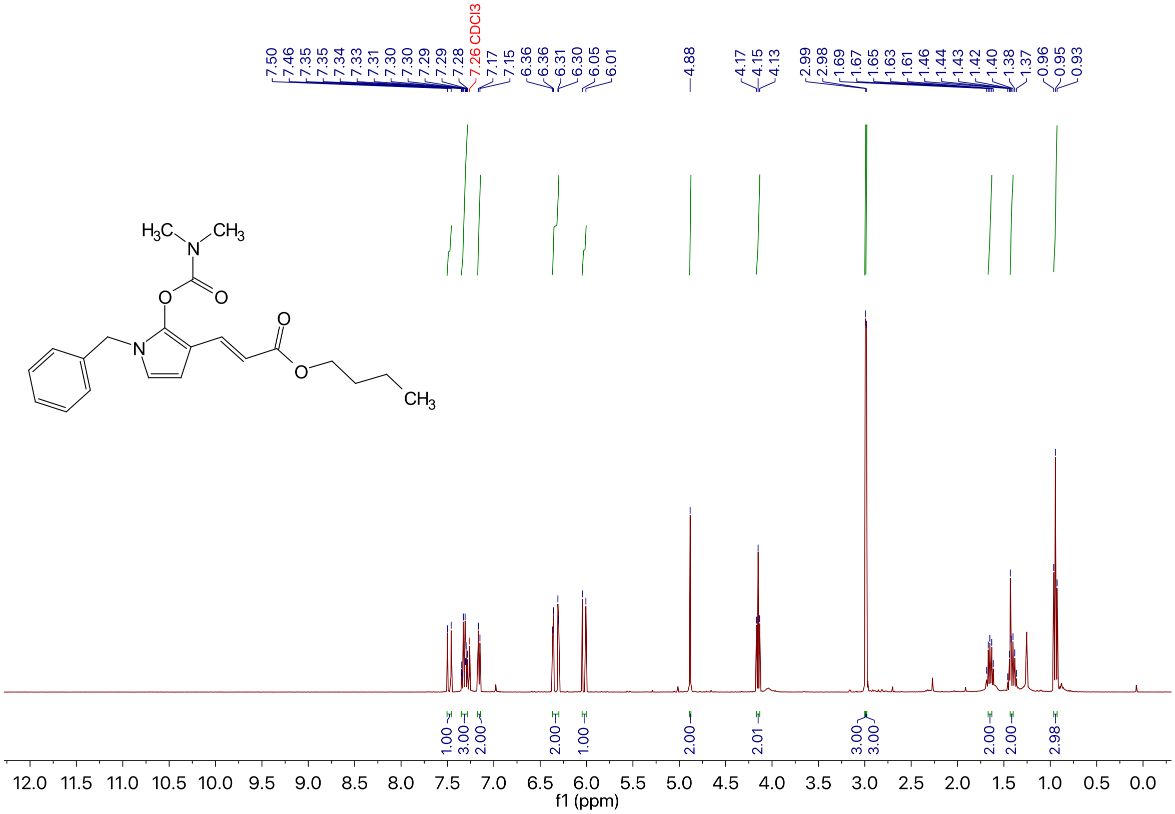

^{13}C NMR spectrum of butyl (*E*)-3-(1-benzyl-2-((dimethylcarbamoyl)oxy)-1*H*-pyrrol-3-yl)acrylate (2x)

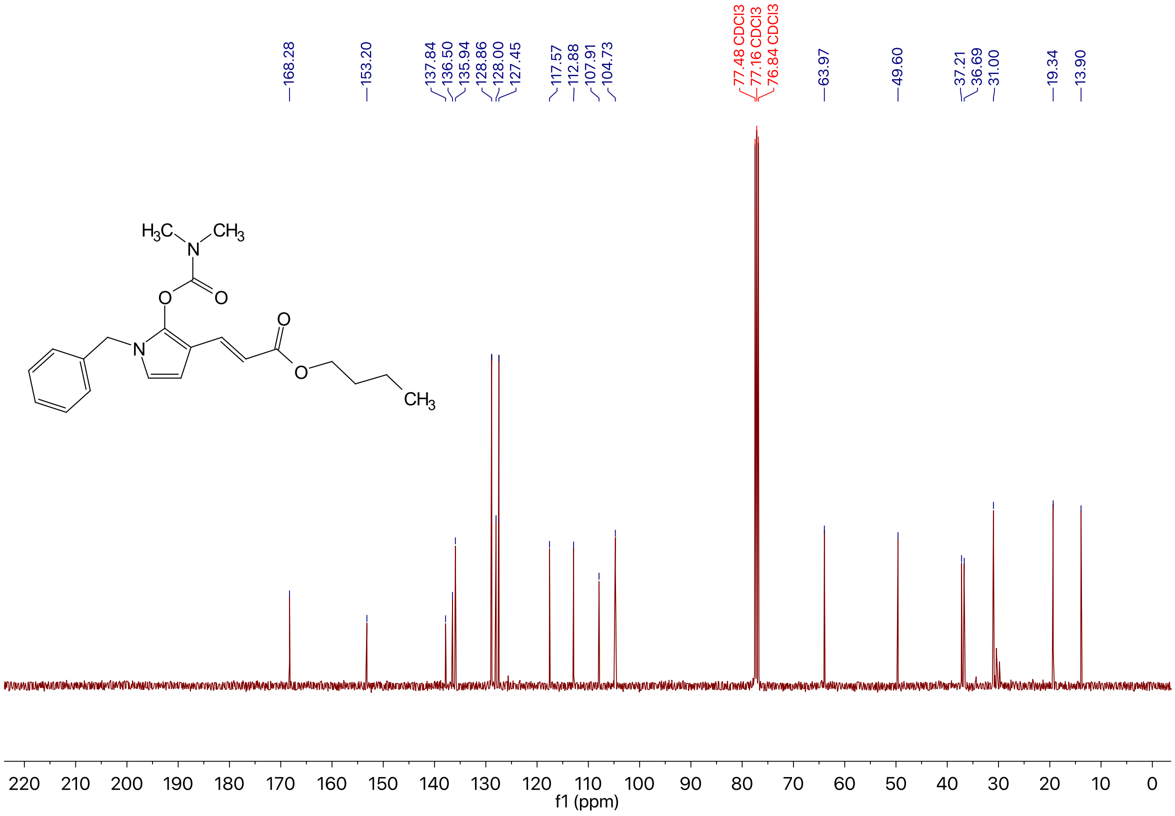

^{1}H NMR spectrum of methyl (*E*)-3-(5-((dimethylcarbamoyl)oxy)furan-2-yl)acrylate (3a)

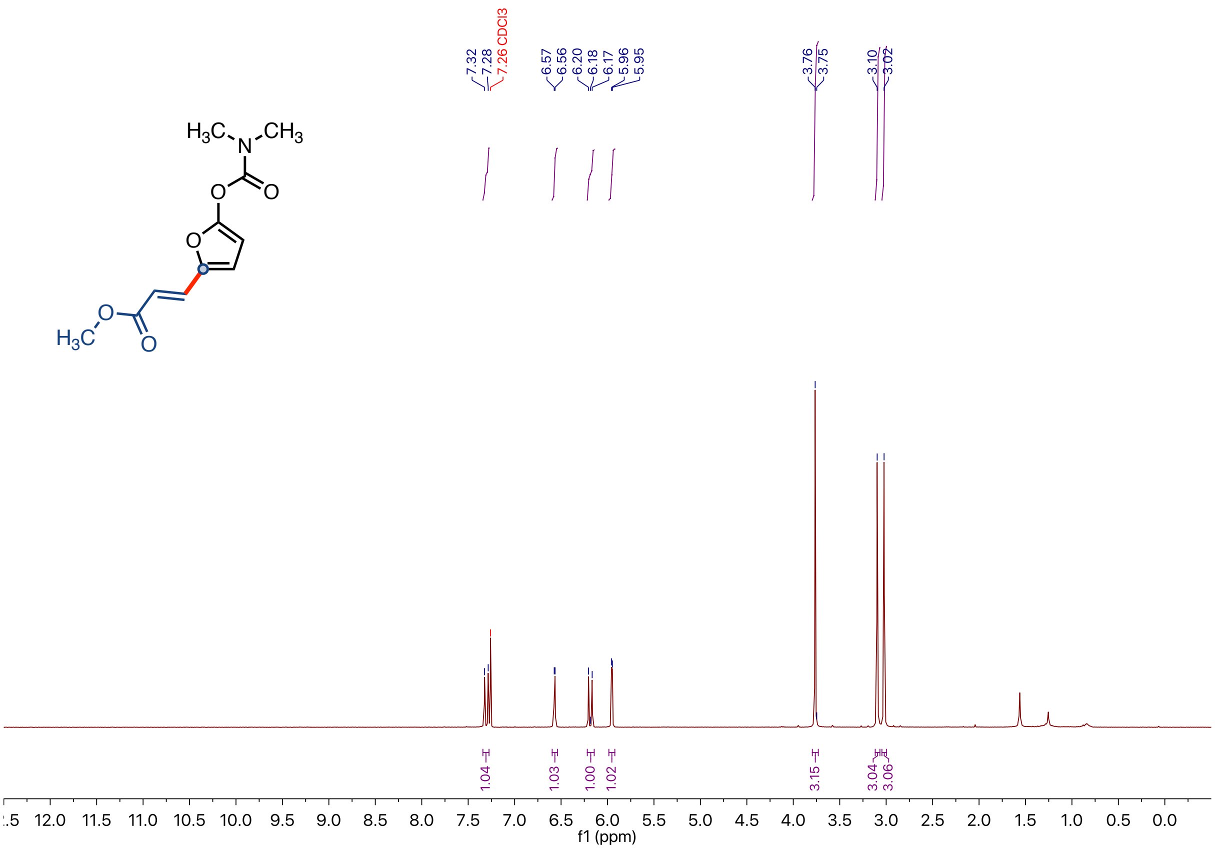

^{13}C NMR spectrum of methyl (*E*)-3-(5-((dimethylcarbamoyl)oxy)furan-2-yl)acrylate (3a)

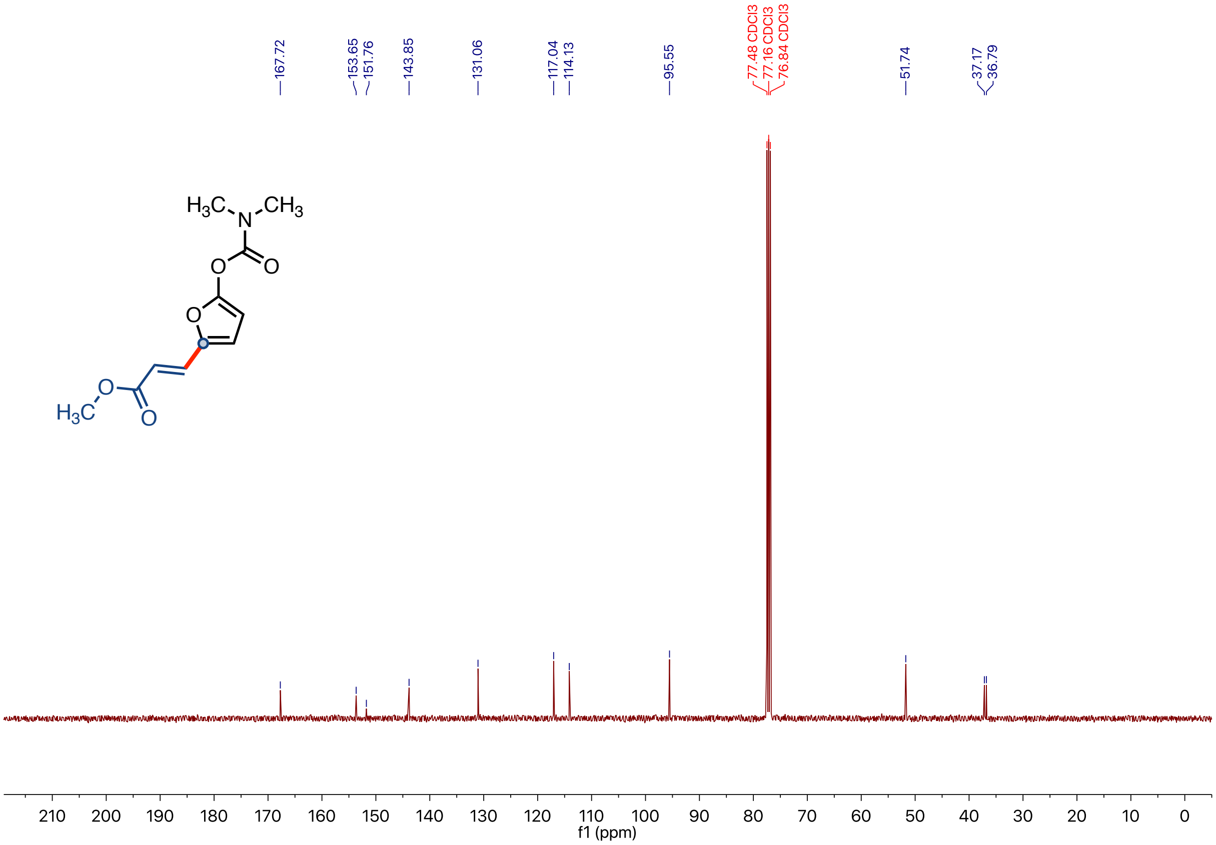

^{1}H NMR spectrum of ethyl (*E*)-3-(5-((dimethylcarbamoyl)oxy)furan-2-yl)acrylate (3b)

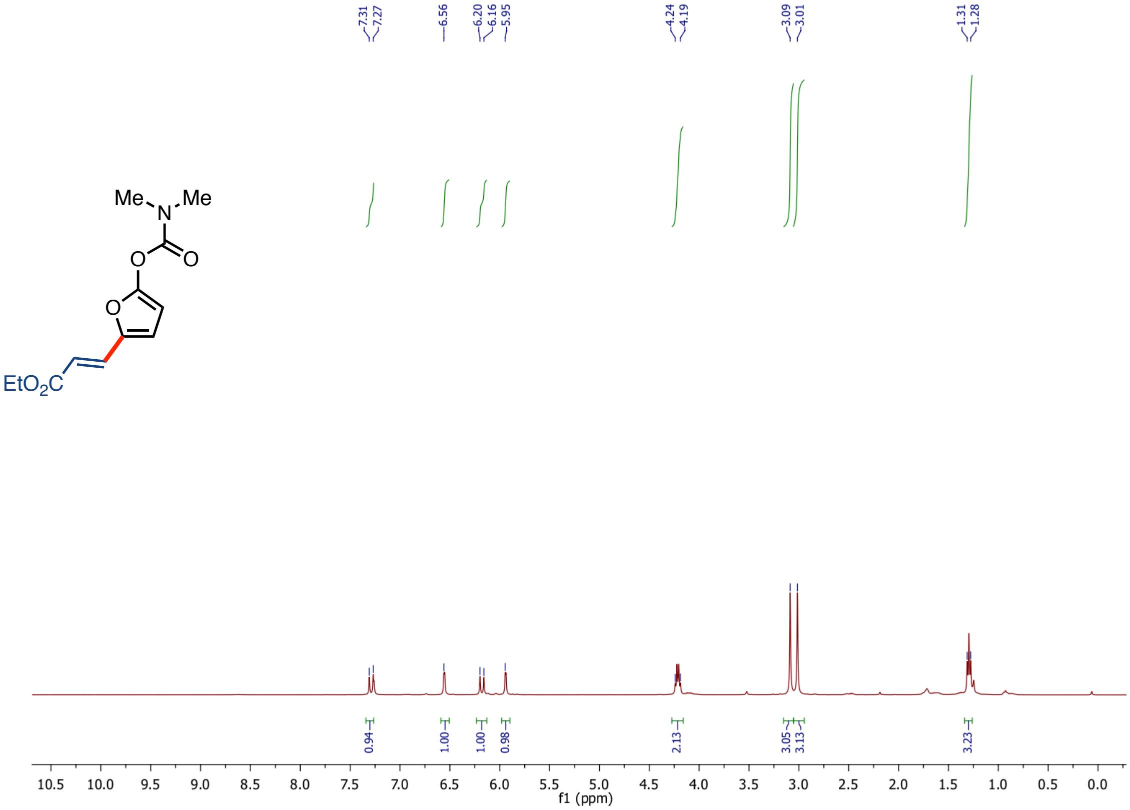

^{13}C NMR spectrum of ethyl (*E*)-3-(5-((dimethylcarbamoyl)oxy)furan-2-yl)acrylate (3b)

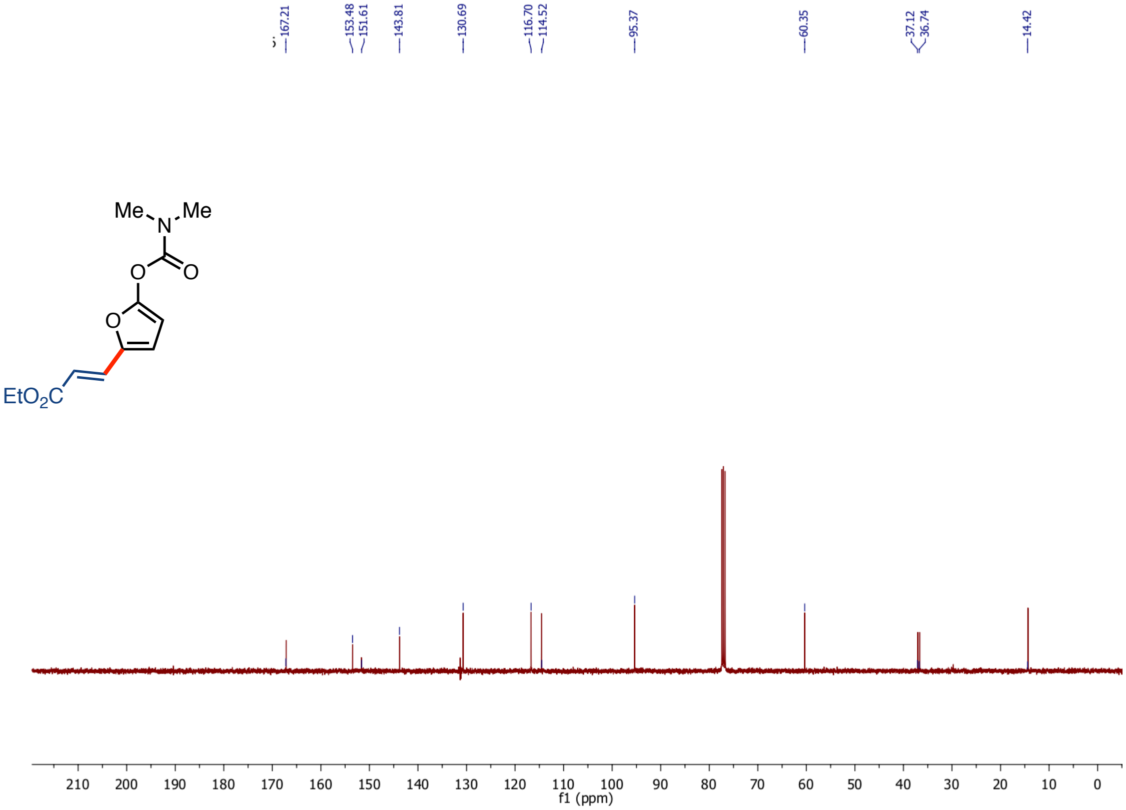

^{1}H NMR spectrum of butyl (*E*)-3-(5-((dimethylcarbamoyl)oxy)furan-2-yl)acrylate (3c)

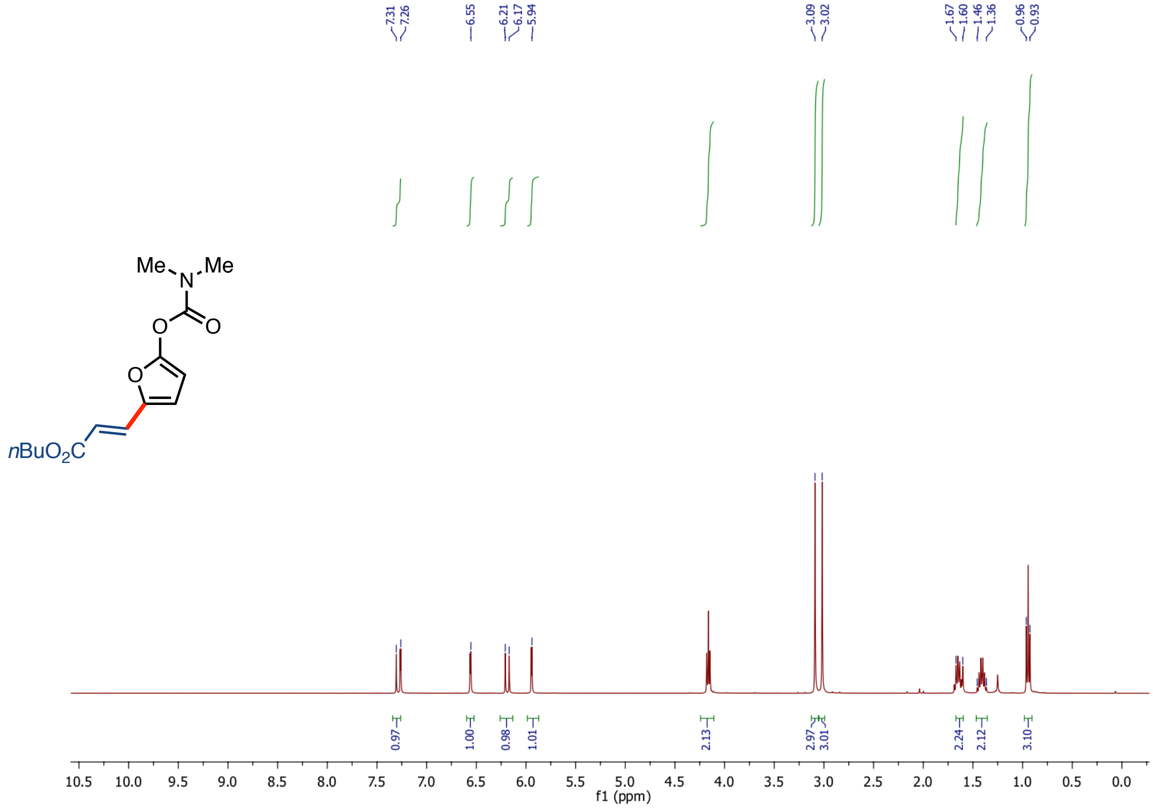

^{13}C NMR spectrum of butyl (*E*)-3-(5-((dimethylcarbamoyl)oxy)furan-2-yl)acrylate (3c)

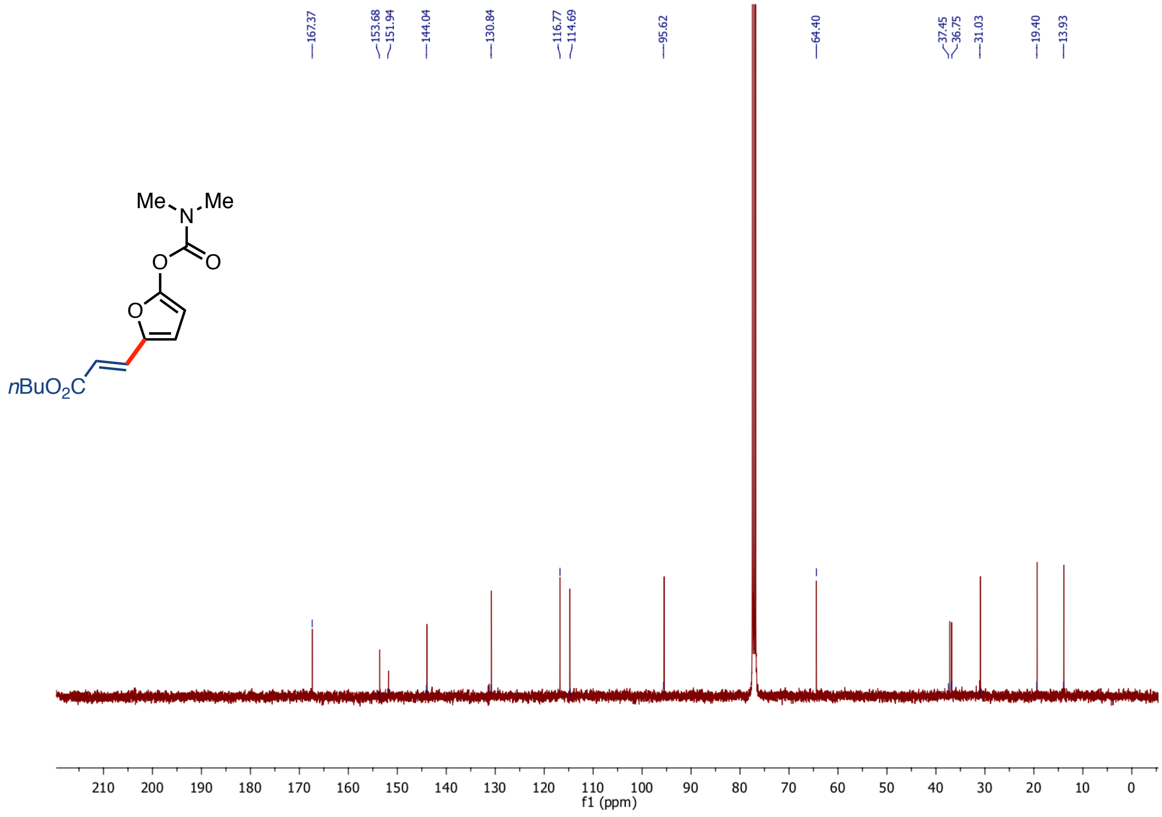

^{1}H NMR spectrum of *tert*-butyl (*E*)-3-(5-((dimethylcarbamoyl)oxy)furan-2-yl)acrylate (3d)

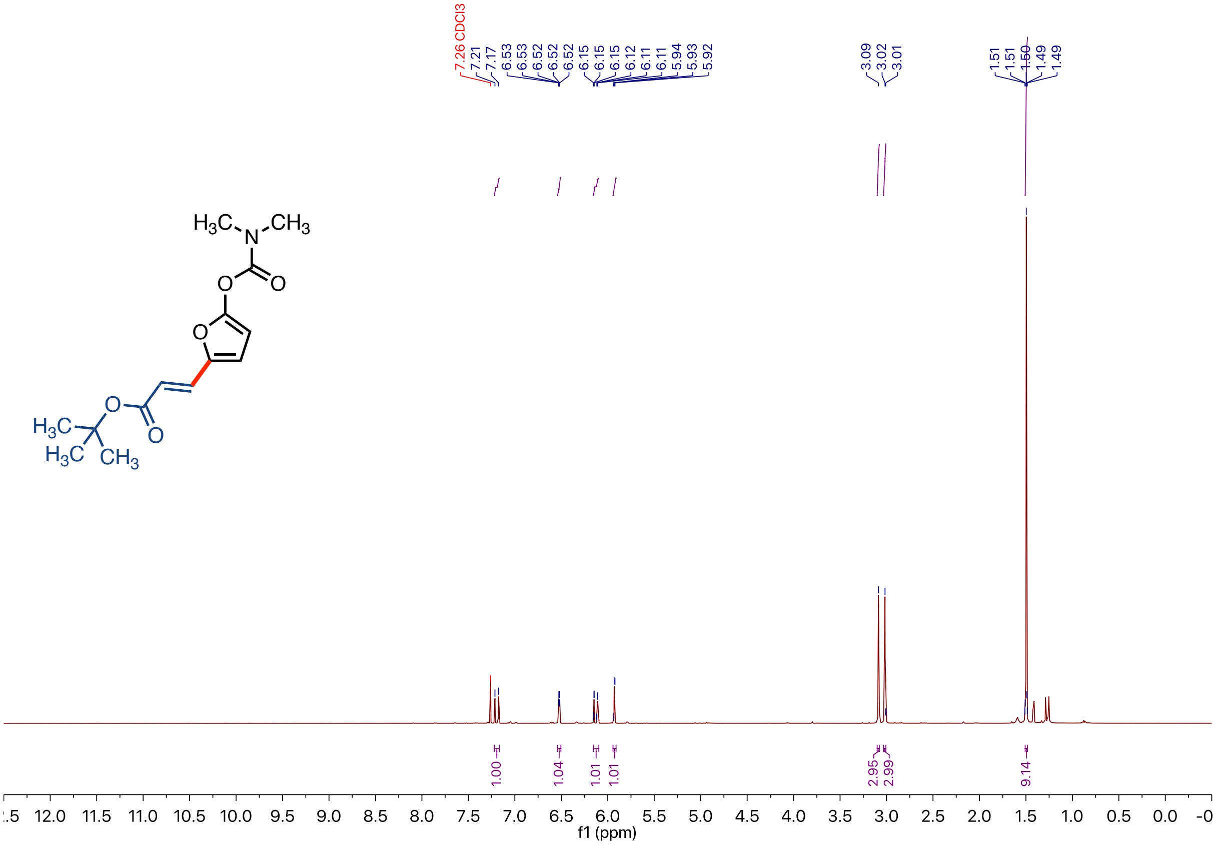

^{13}C NMR spectrum of *tert*-butyl (*E*)-3-(5-((dimethylcarbamoyl)oxy)furan-2-yl)acrylate (3d)

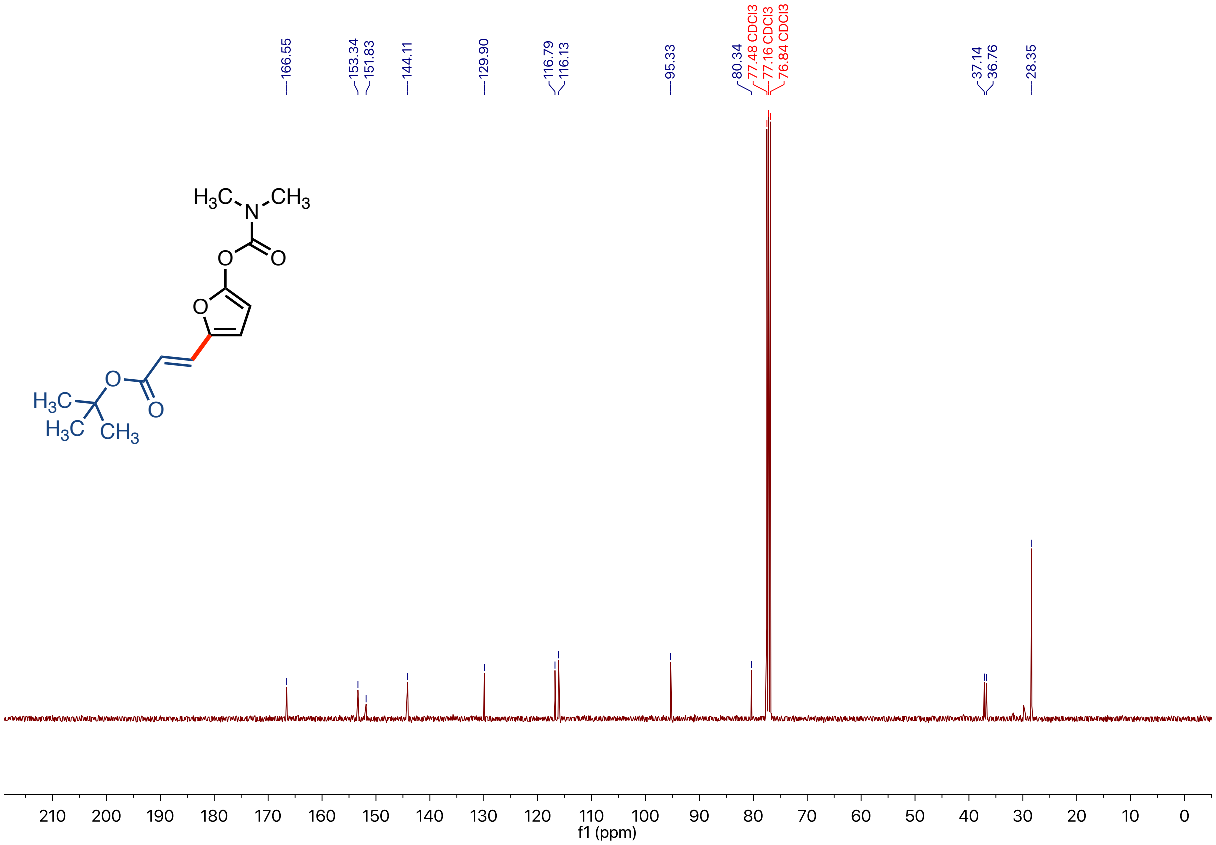

^{1}H NMR spectrum of benzyl (*E*)-3-(5-((dimethylcarbamoyl)oxy)furan-2-yl)acrylate (3e)

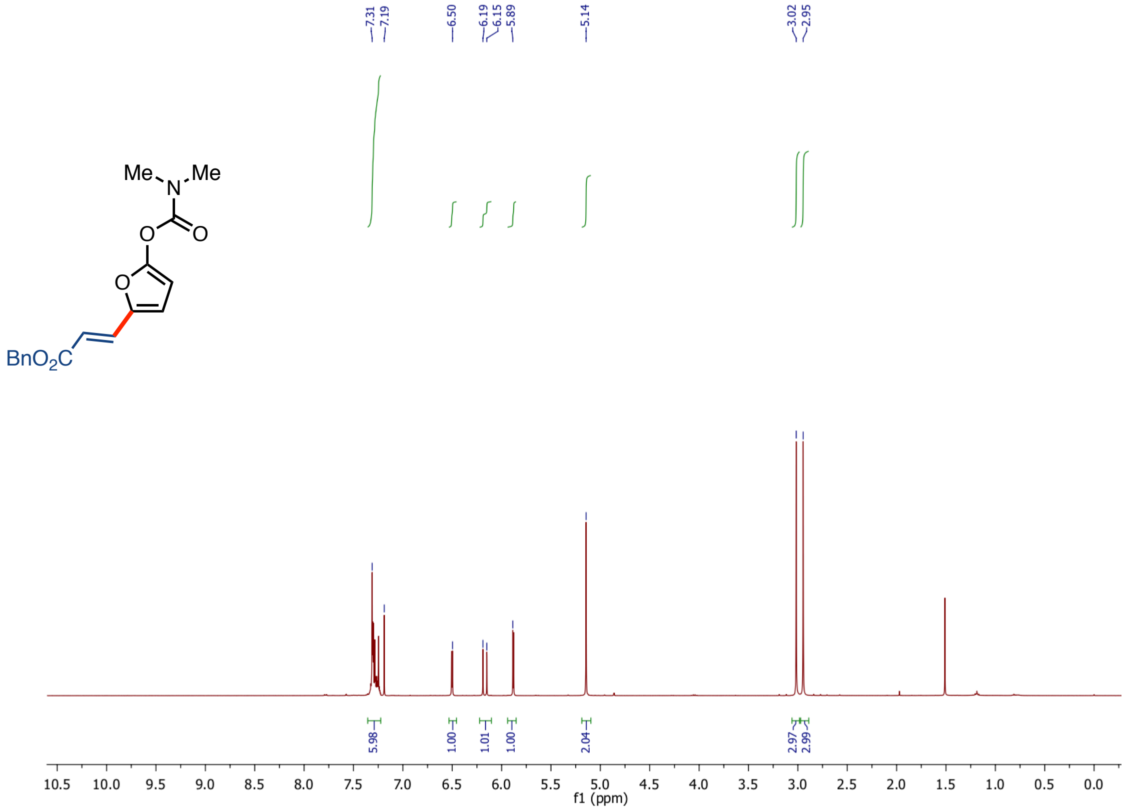

^{13}C NMR spectrum of benzyl (*E*)-3-(5-((dimethylcarbamoyl)oxy)furan-2-yl)acrylate (3e)

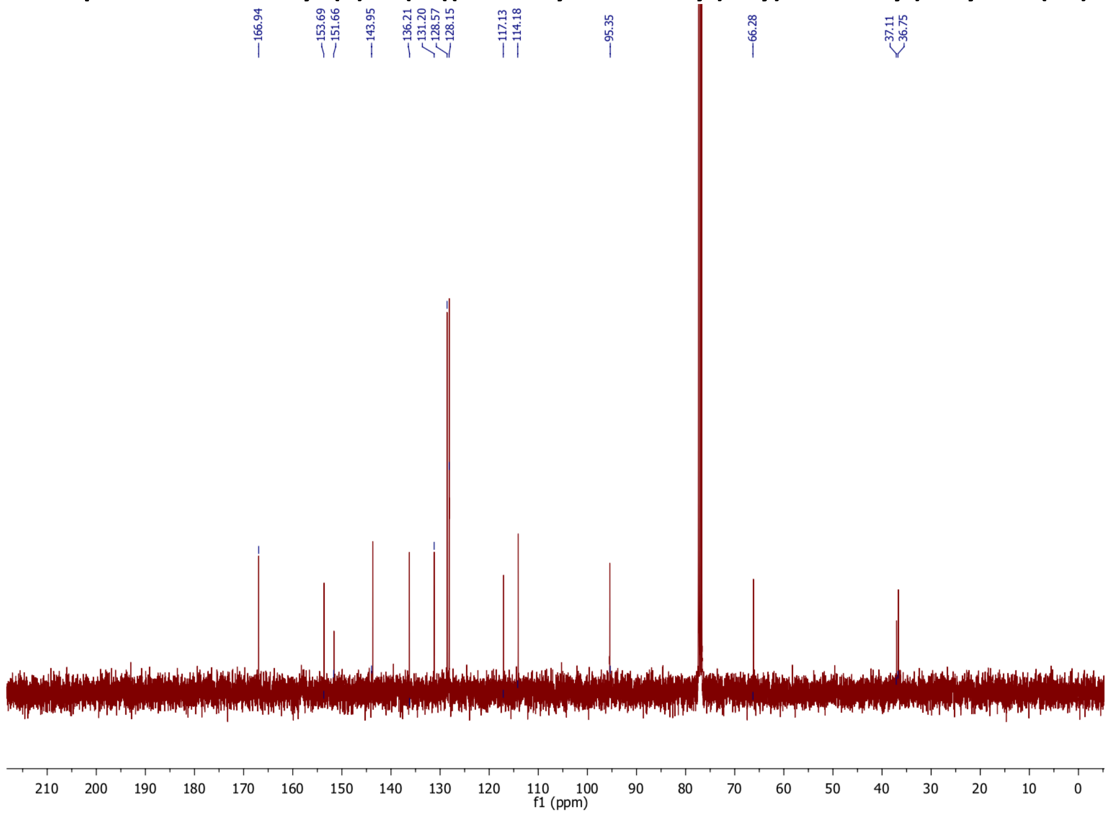

^{1}H NMR spectrum of methyl (*E*)-3-(5-((dimethylcarbamoyl)oxy)-4-methylfuran-2-yl)acrylate (3f)

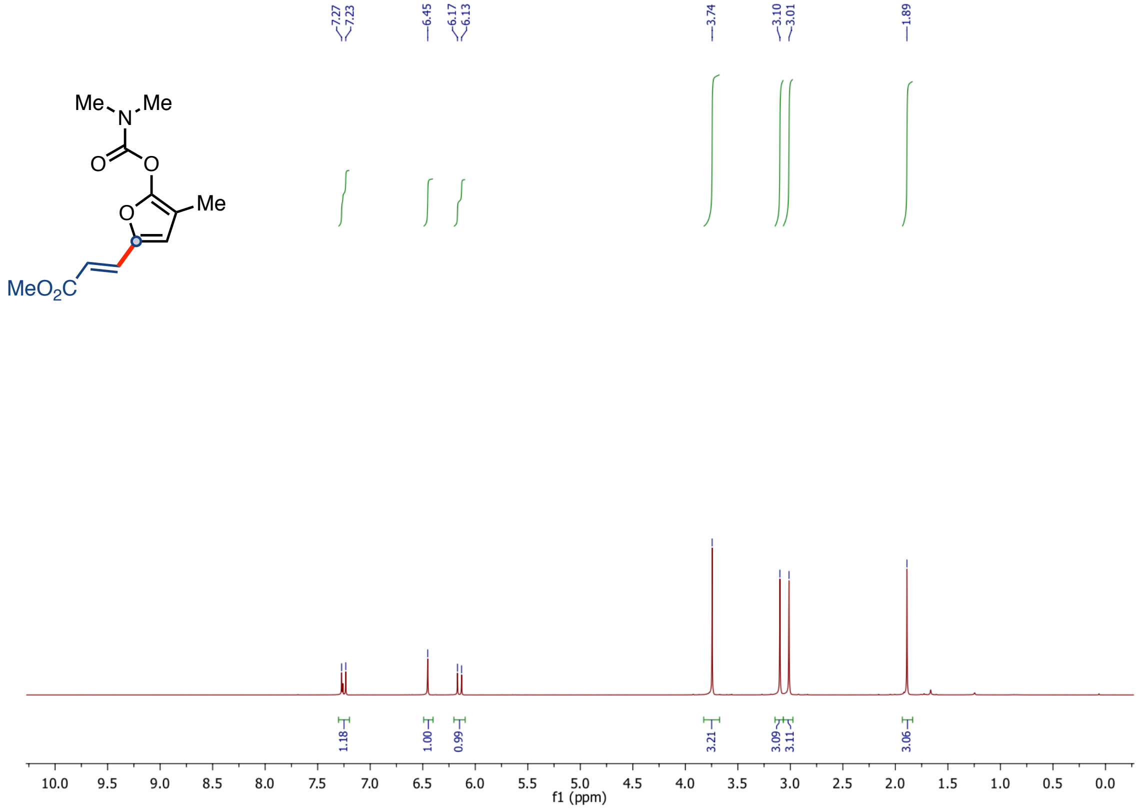

^{13}C NMR spectrum of methyl (*E*)-3-(5-((dimethylcarbamoyl)oxy)-4-methylfuran-2-yl)acrylate (3f)

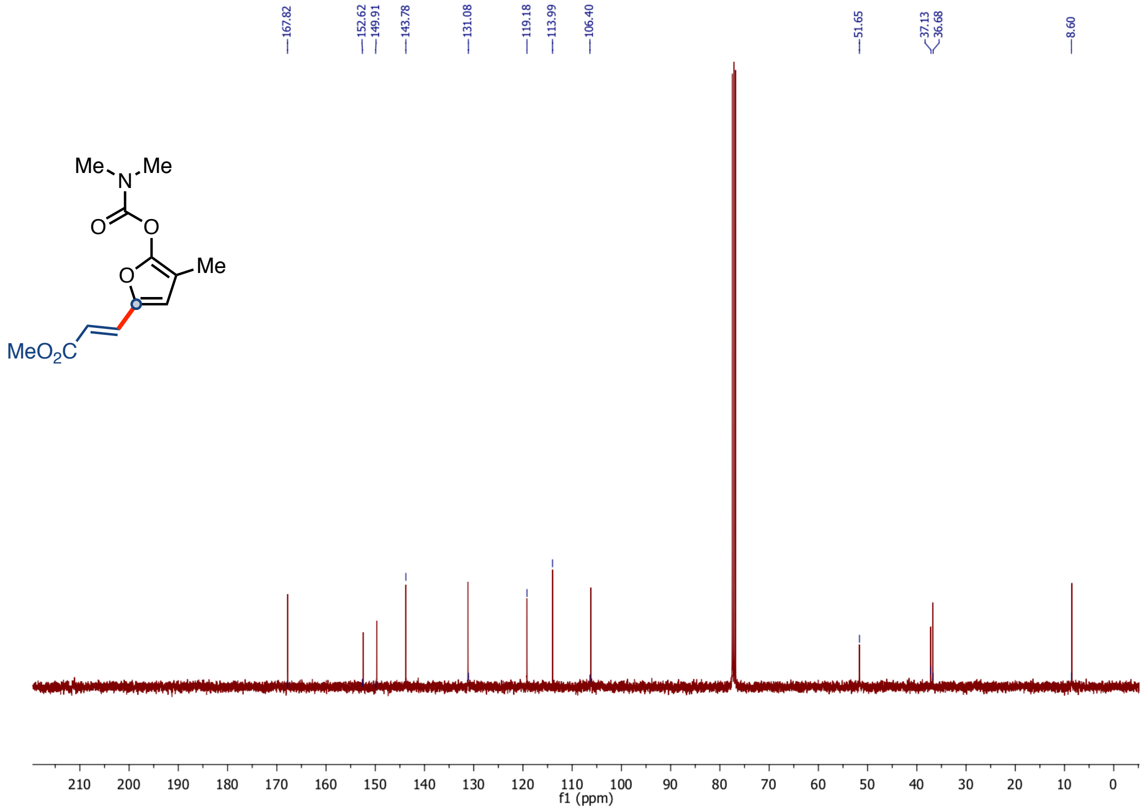

^{1}H NMR spectrum of methyl (*E*)-3-(5-((dimethylcarbamoyl)oxy)-4-phenylfuran-2-yl)acrylate (3g)

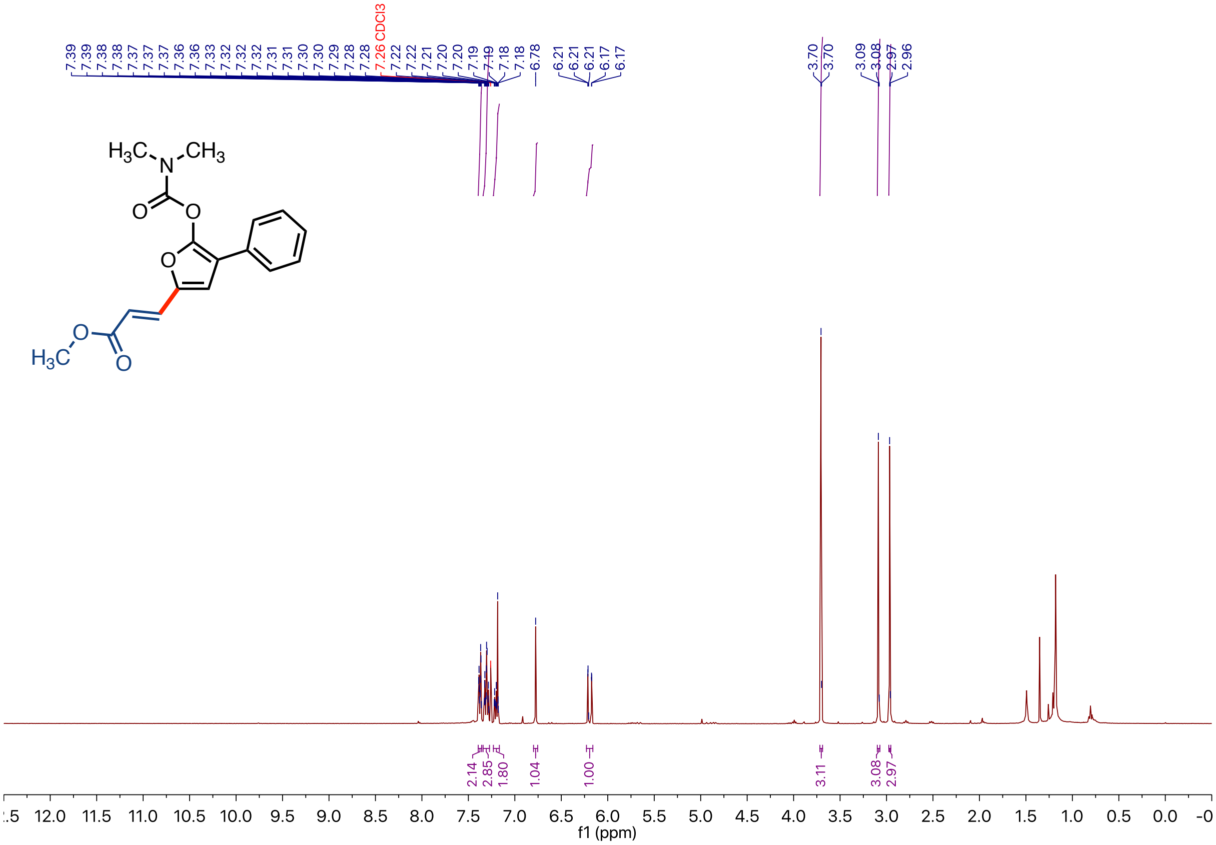

^{13}C NMR spectrum of methyl (*E*)-3-(5-((dimethylcarbamoyl)oxy)-4-phenylfuran-2-yl)acrylate (3g)

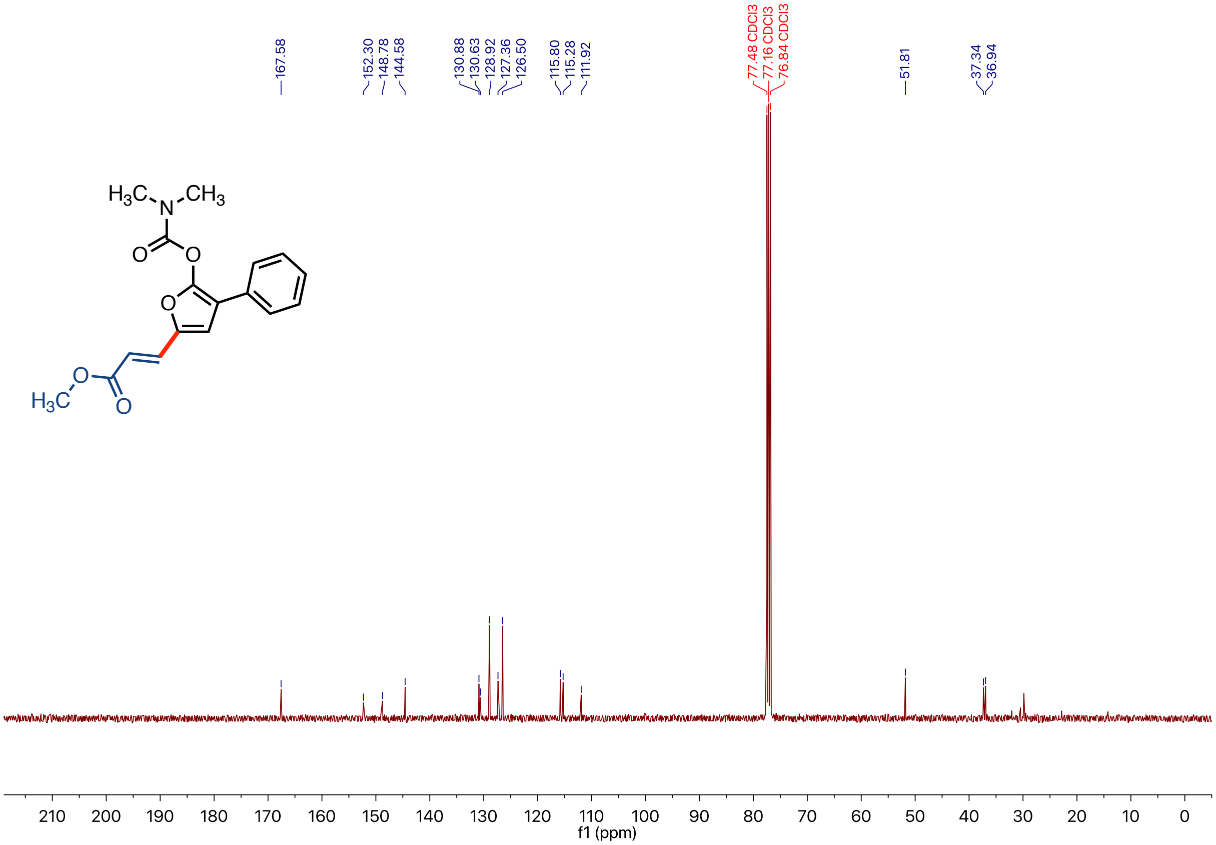

^{1}H NMR spectrum of methyl (*E*)-3-(5-((dimethylcarbamoyl)oxy)-4-(4-ethylphenyl)furan-2-yl)acrylate (3h)

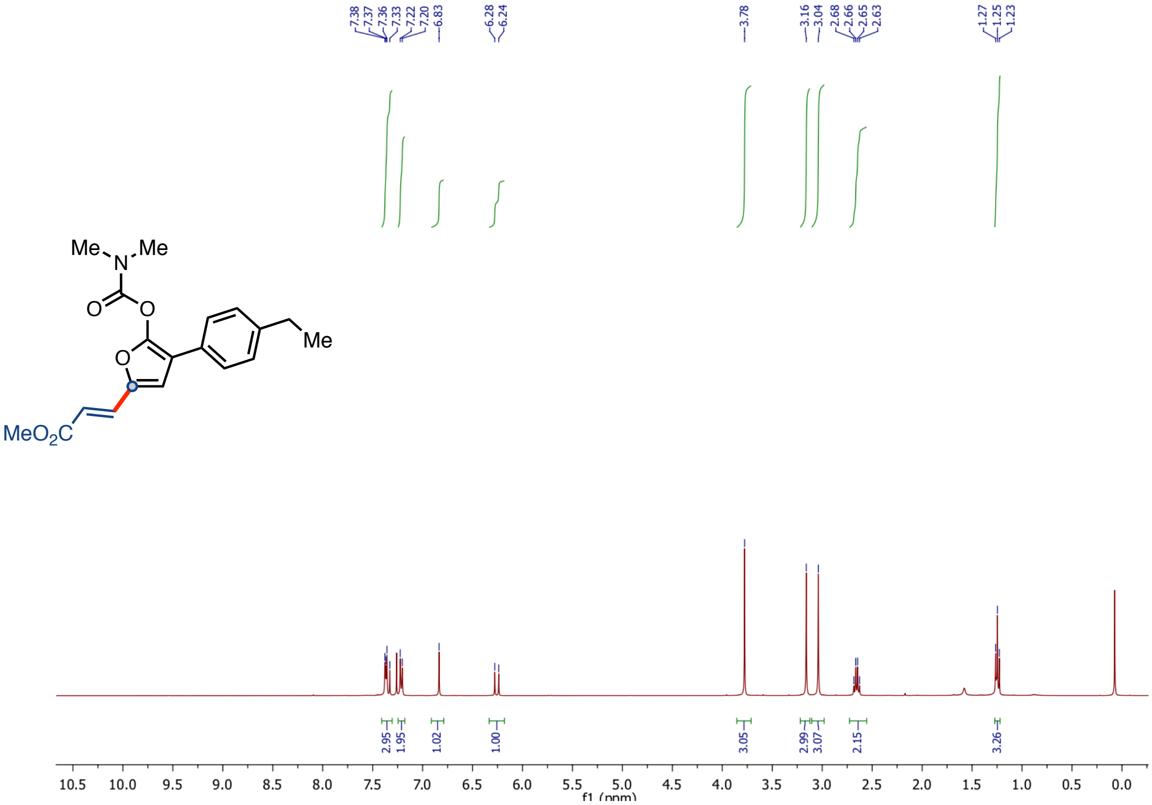

^{13}C NMR spectrum of methyl (*E*)-3-(5-((dimethylcarbamoyl)oxy)-4-(4-ethylphenyl)furan-2-yl)acrylate (3h)

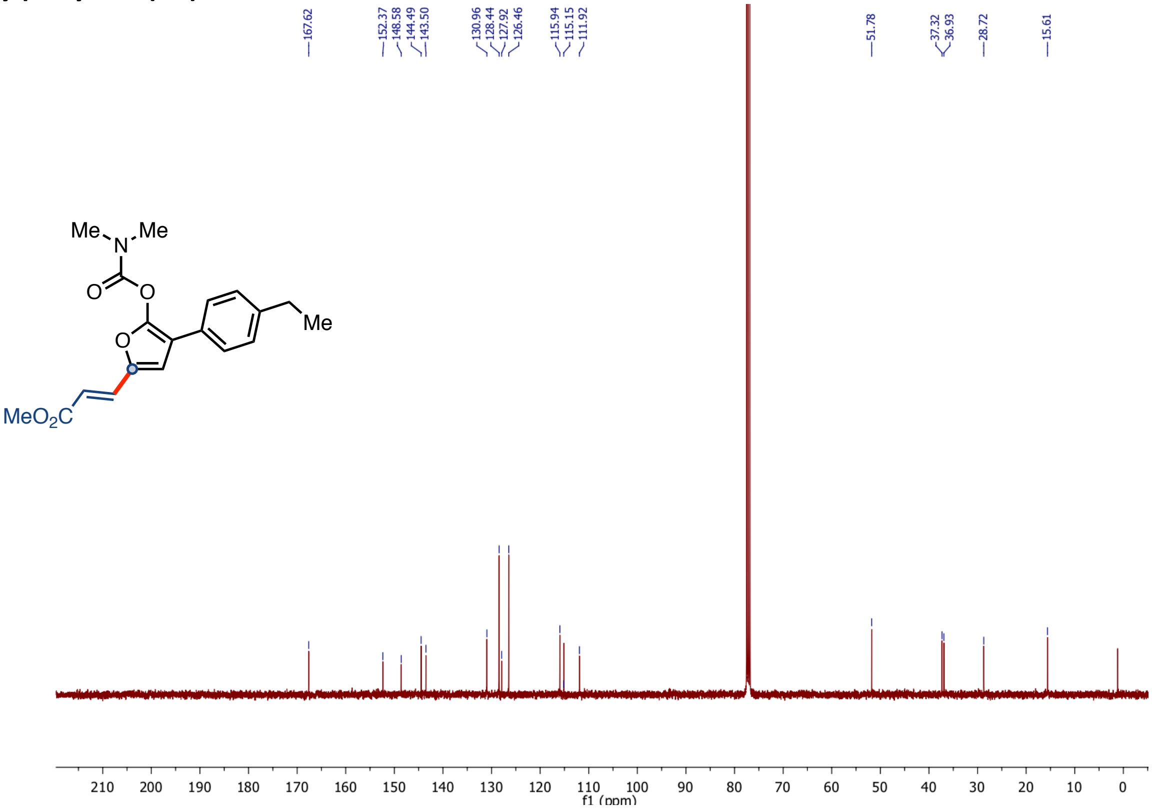

^{1}H NMR spectrum of methyl (*E*)-3-(5-((dimethylcarbamoyl)oxy)-4-(4-fluorophenyl)furan-2-yl)acrylate (3i)

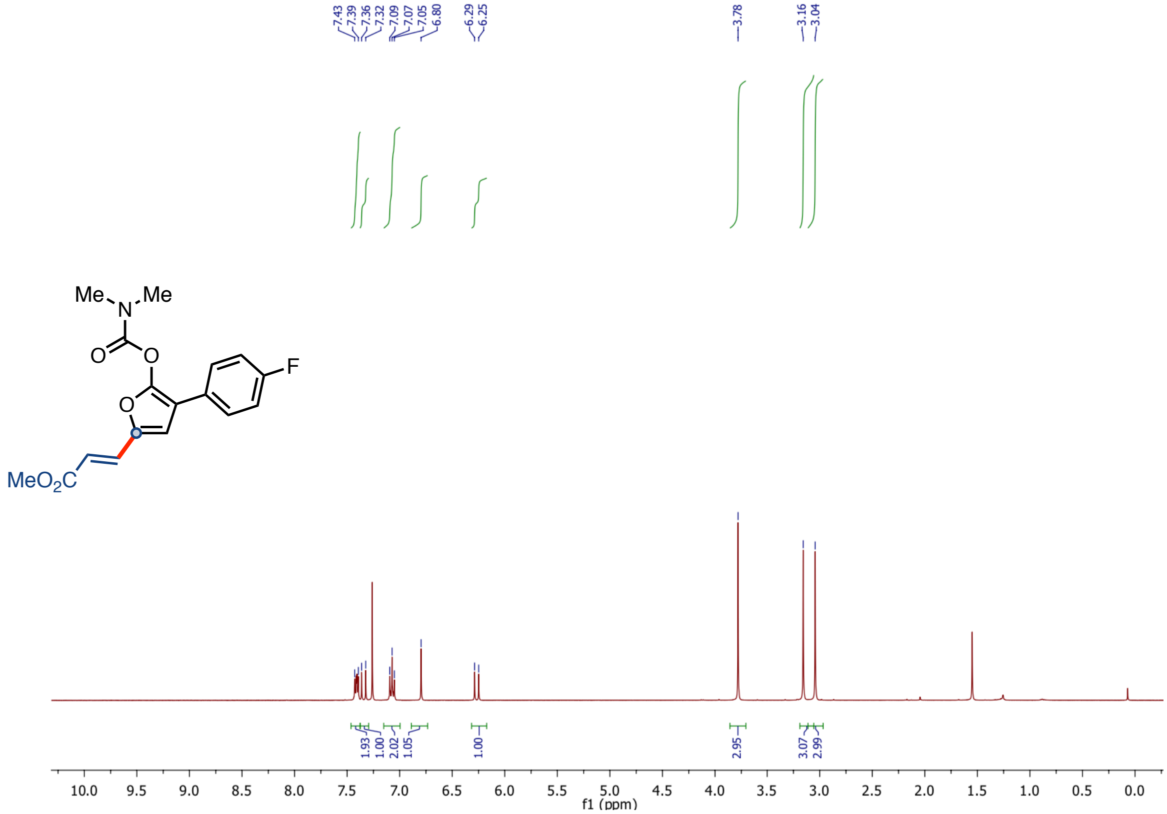

^{13}C NMR spectrum of methyl (*E*)-3-(5-((dimethylcarbamoyl)oxy)-4-(4-fluorophenyl)furan-2-yl)acrylate (3i)

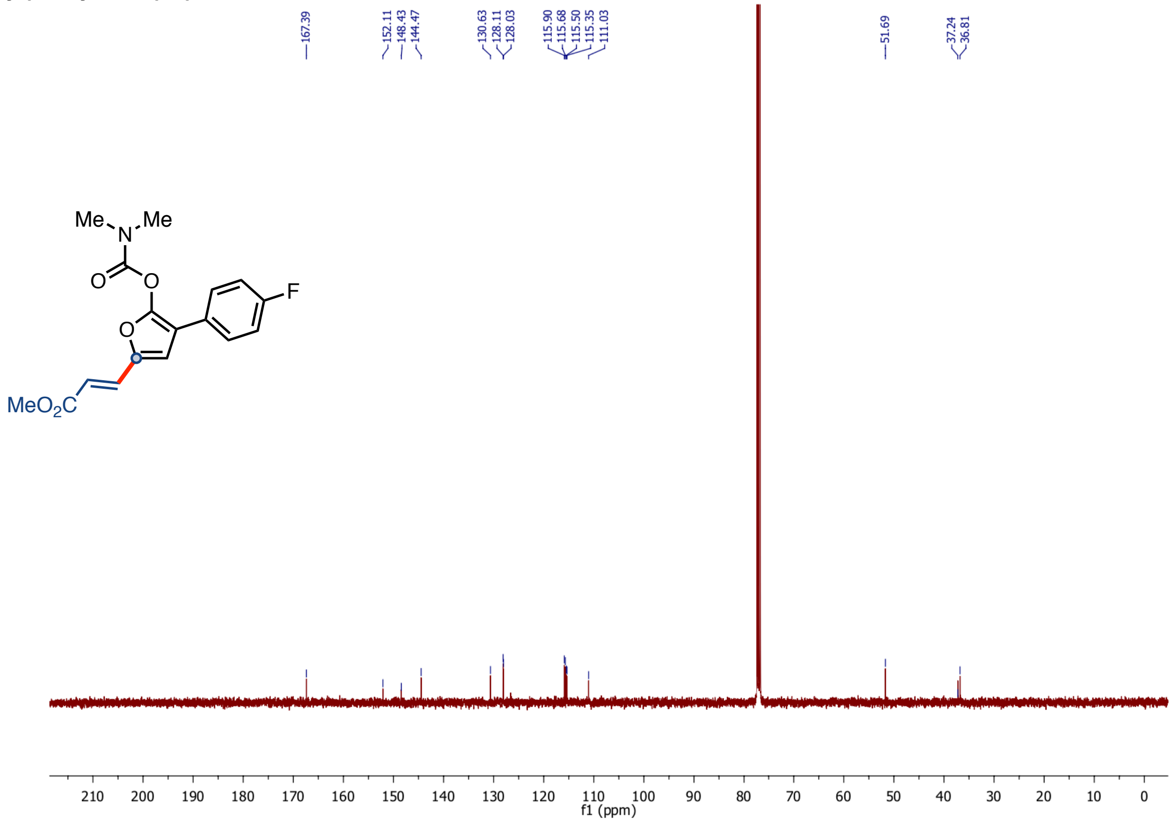

^{1}H NMR spectrum of methyl 4-((dimethylcarbamoyl)oxy)-5-methyl-7-oxabicyclo[2.2.1]hept-5-ene-2-carboxylate

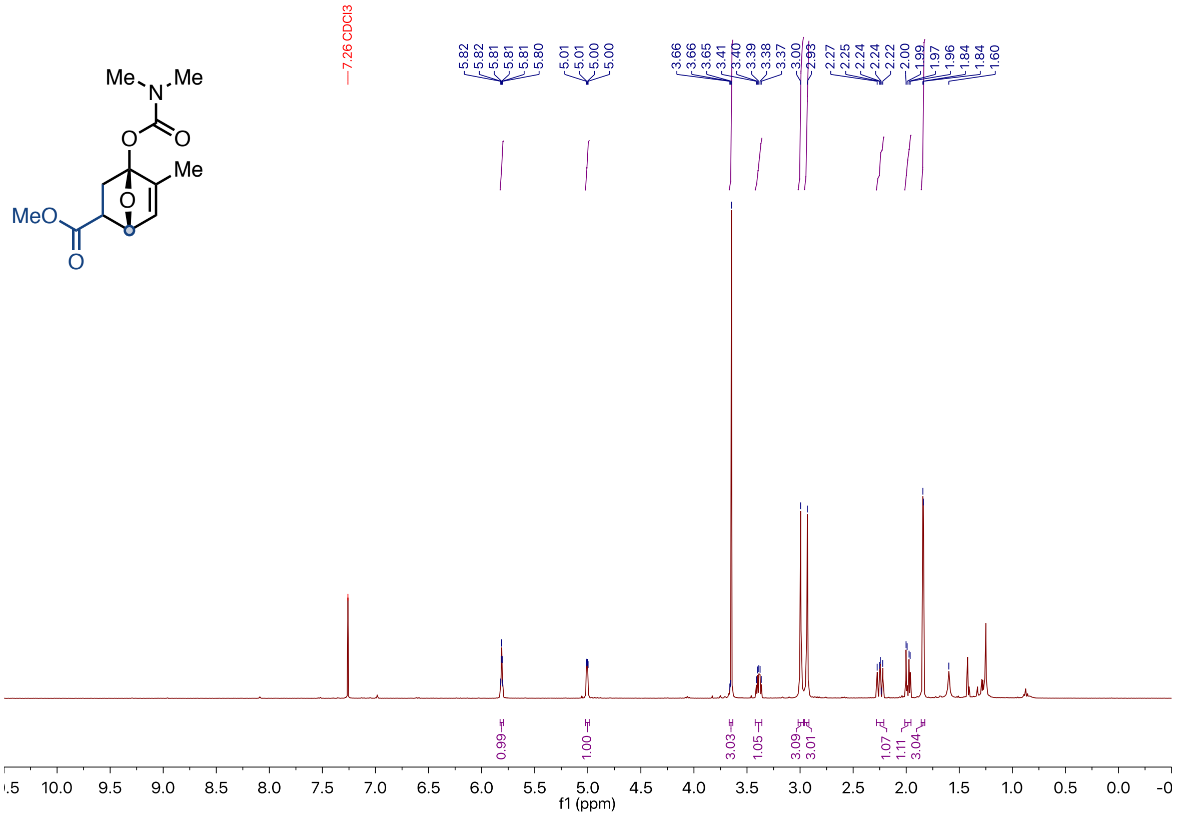

^{13}C NMR spectrum of methyl 4-((dimethylcarbamoyl)oxy)-5-methyl-7-oxabicyclo[2.2.1]hept-5-ene-2-carboxylate

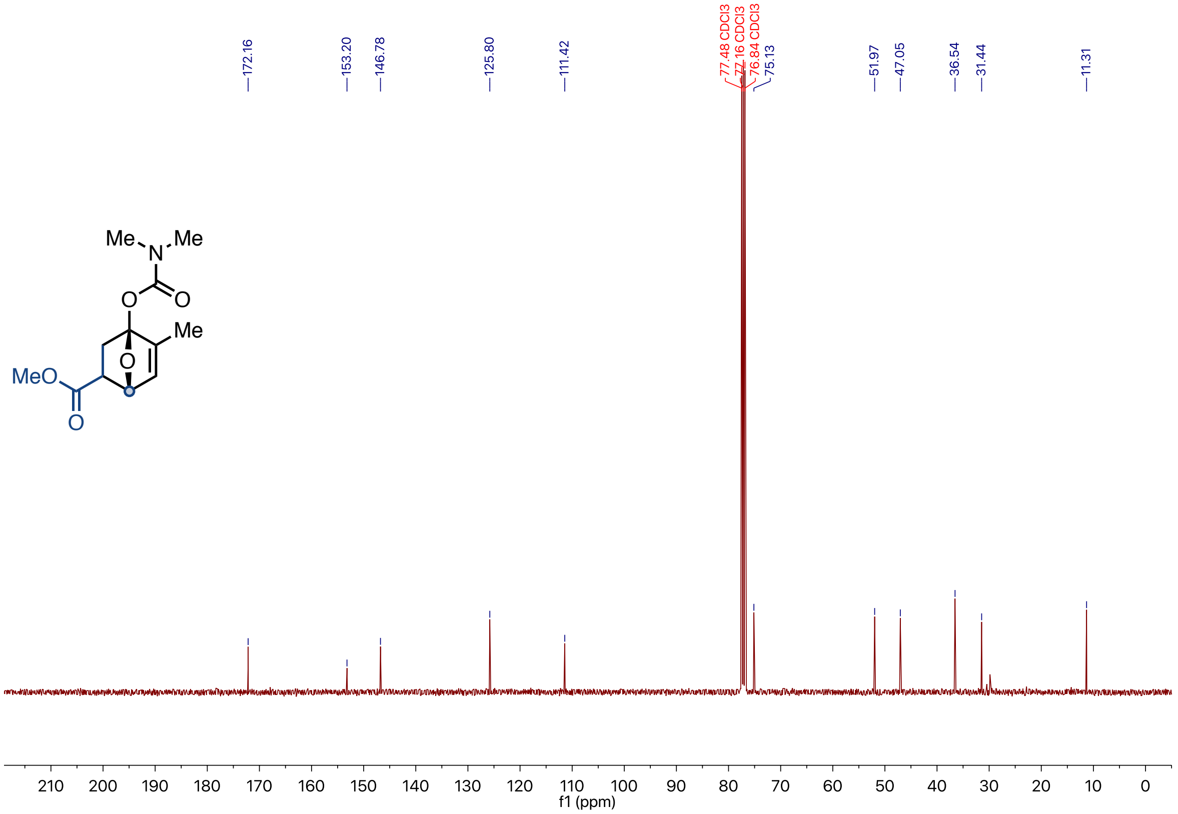

^{1}H NMR spectrum of ethyl (*E*)-3-(2-oxo-2,5-dihydrofuran-3-yl)acrylate (4)

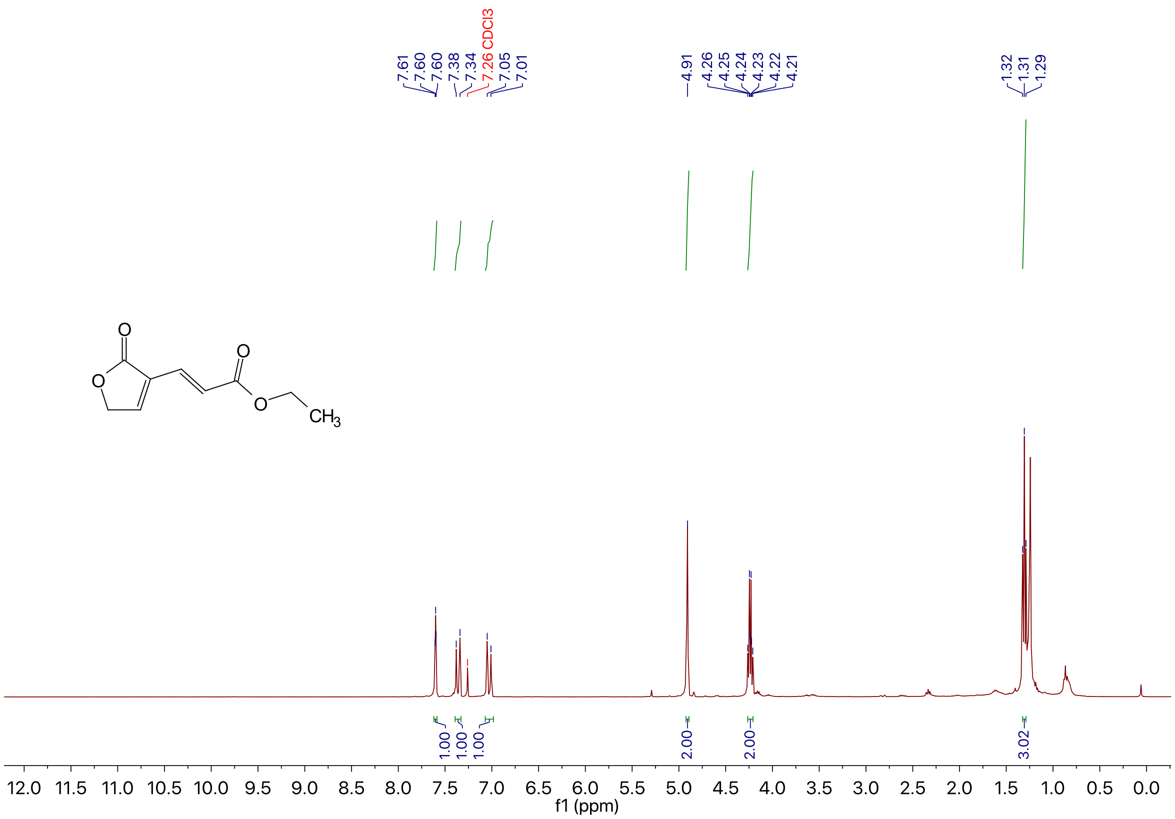

^{13}C NMR spectrum of ethyl (*E*)-3-(2-oxo-2,5-dihydrofuran-3-yl)acrylate (4)

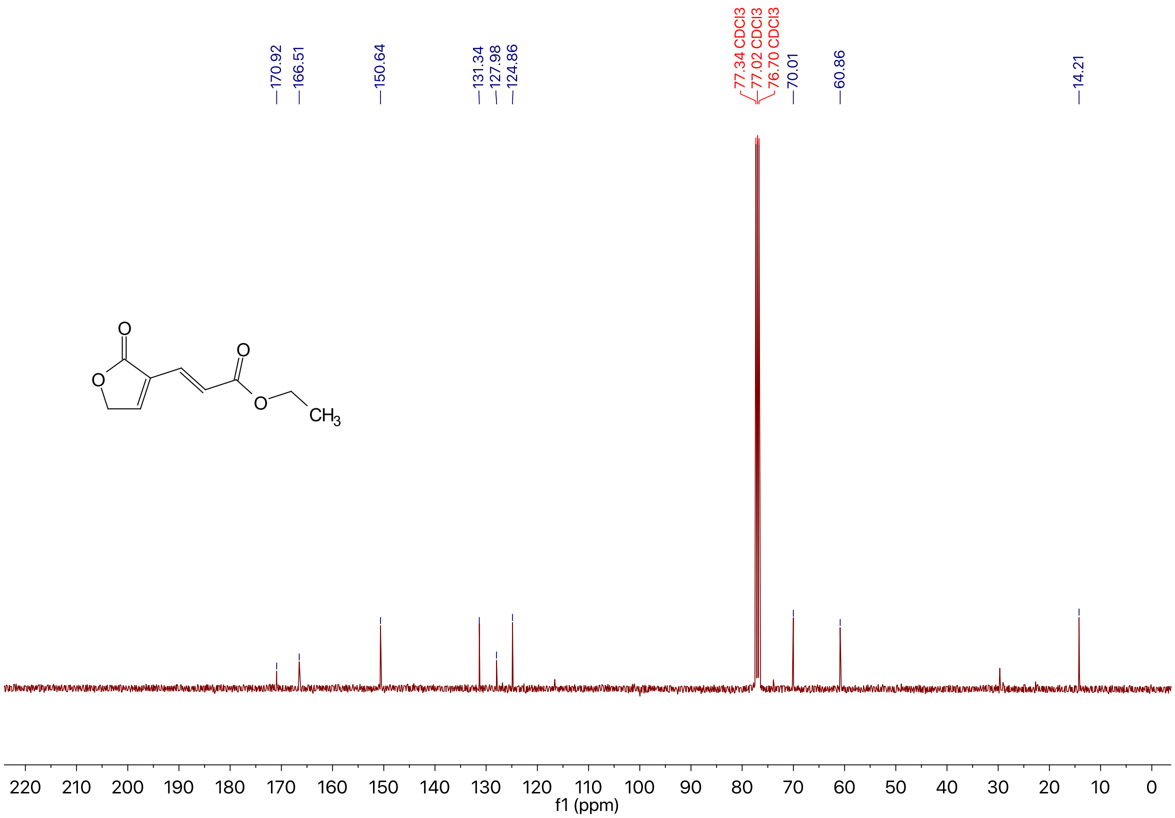

^{1}H NMR spectrum of butyl (*E*)-3-(2-hexylfuran-3-yl)acrylate (5)

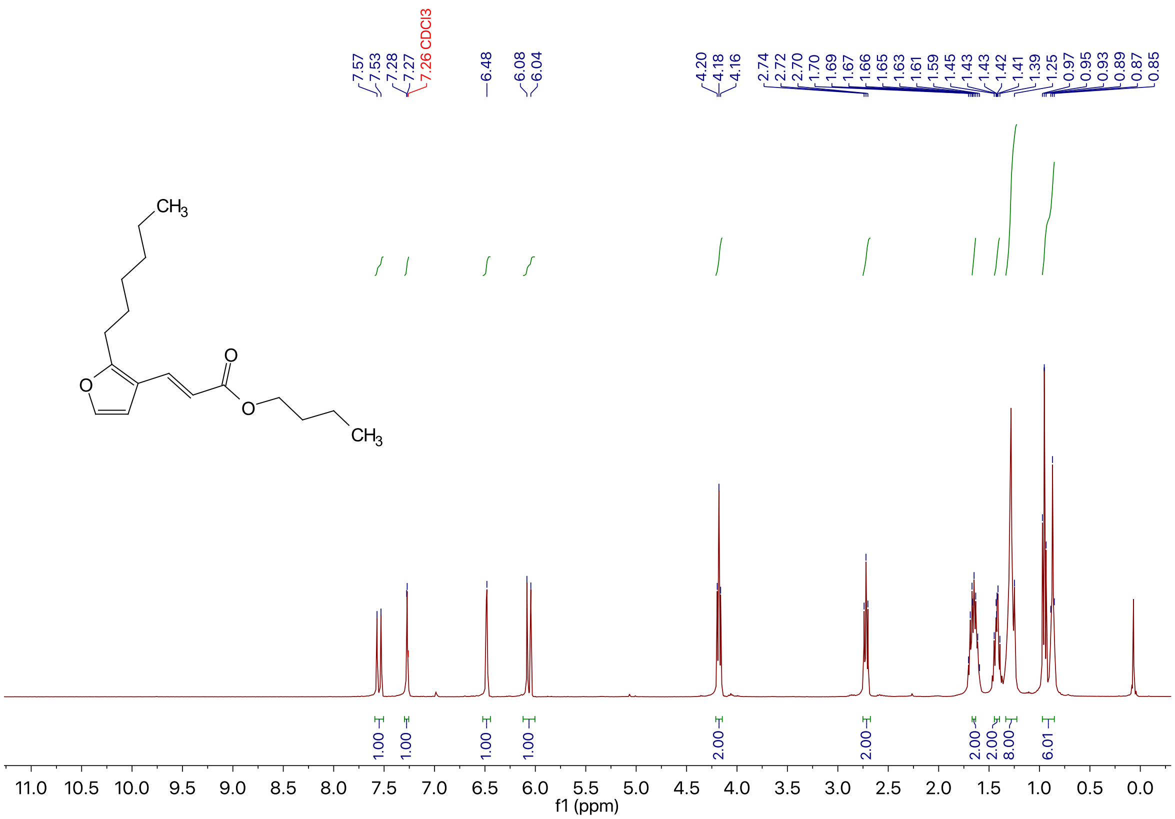

^{13}C NMR spectrum of butyl (*E*)-3-(2-hexylfuran-3-yl)acrylate (5)

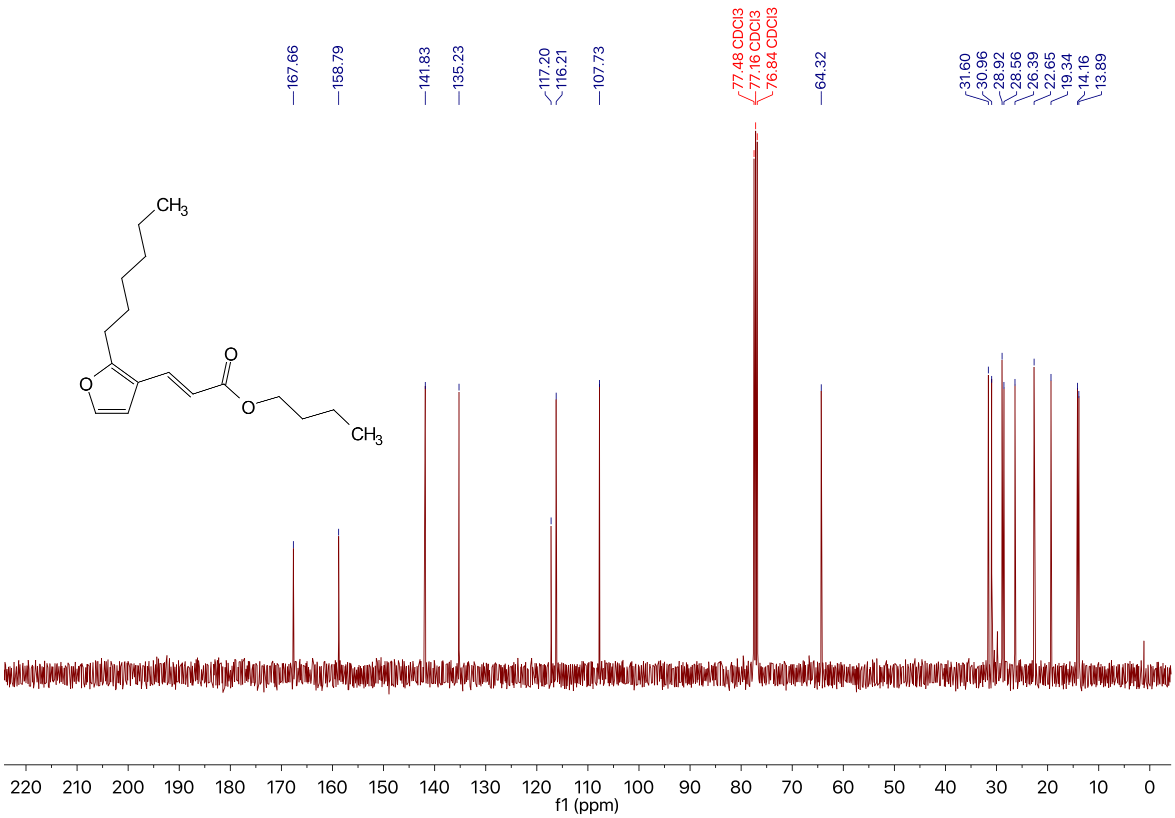

^{1}H NMR spectrum of butyl (*E*)-3-(furan-3-yl)acrylate (6)

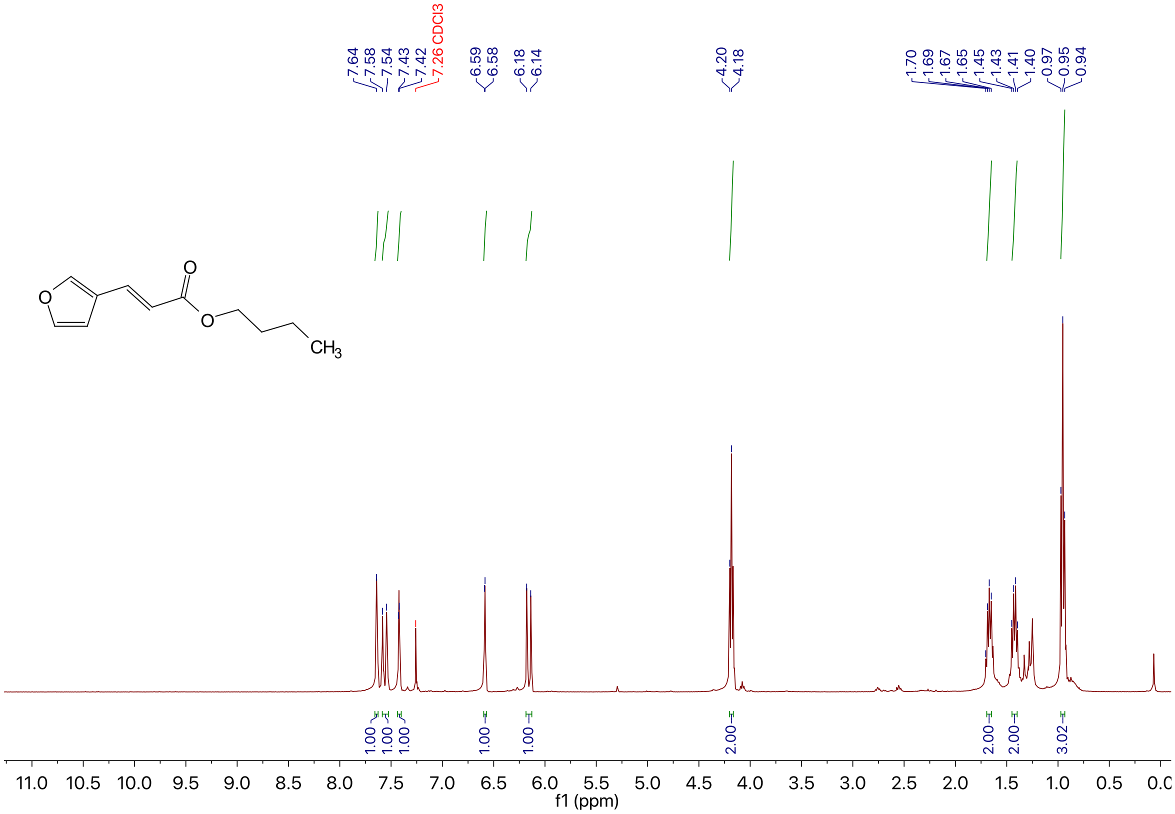

^{13}C NMR spectrum of butyl (*E*)-3-(furan-3-yl)acrylate (6)

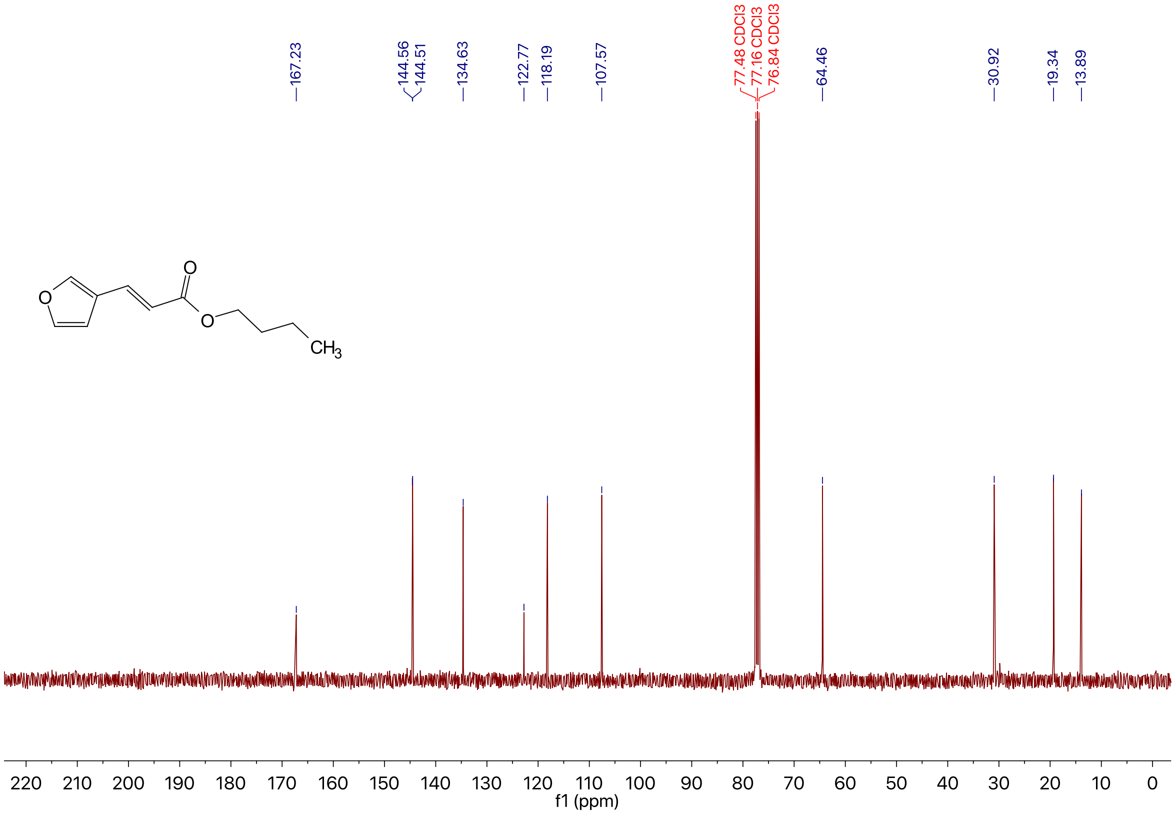

^{1}H NMR spectrum of butyl (*E*)-3-(2-(4-methoxyphenyl)furan-3-yl)acrylate (7)

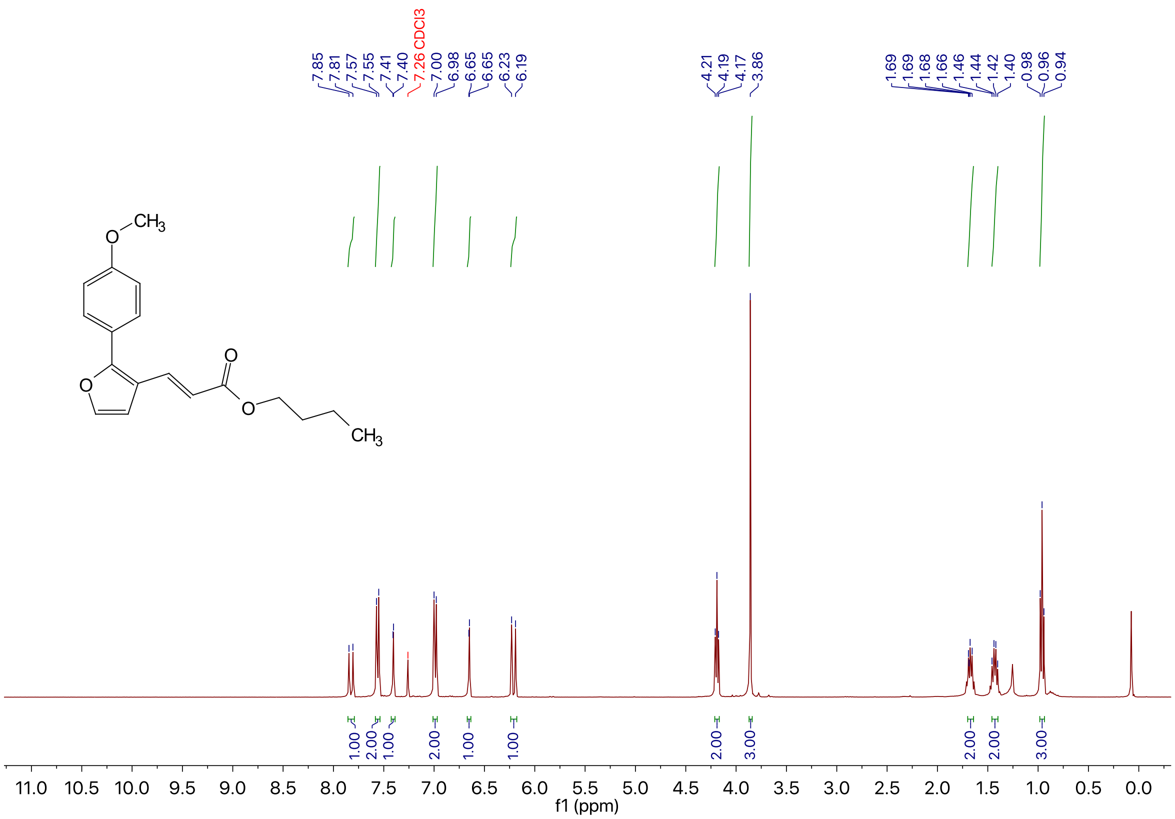

^{13}C NMR spectrum of butyl (*E*)-3-(2-(4-methoxyphenyl)furan-3-yl)acrylate (7)

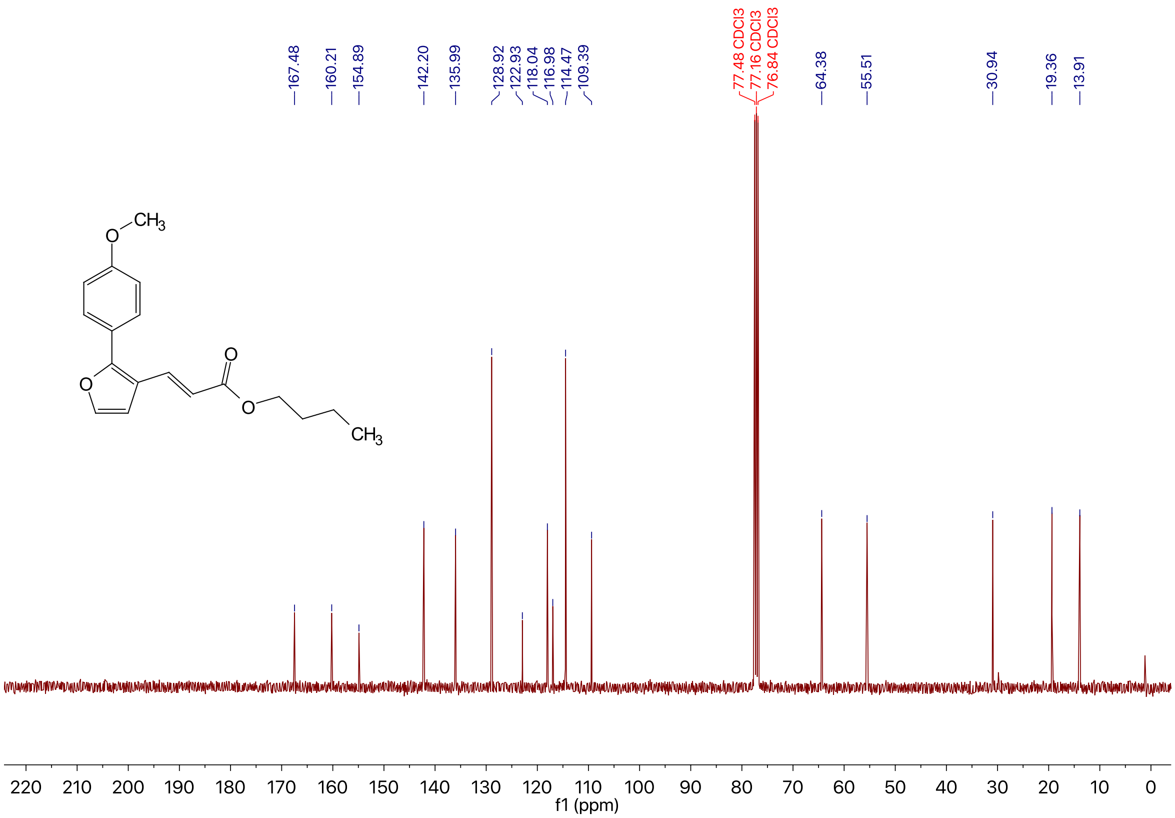

^{1}H NMR spectrum of (*E*)-3-(3-hydroxyprop-1-en-1-yl)furan-2-yl-dimethylcarbamate (8)

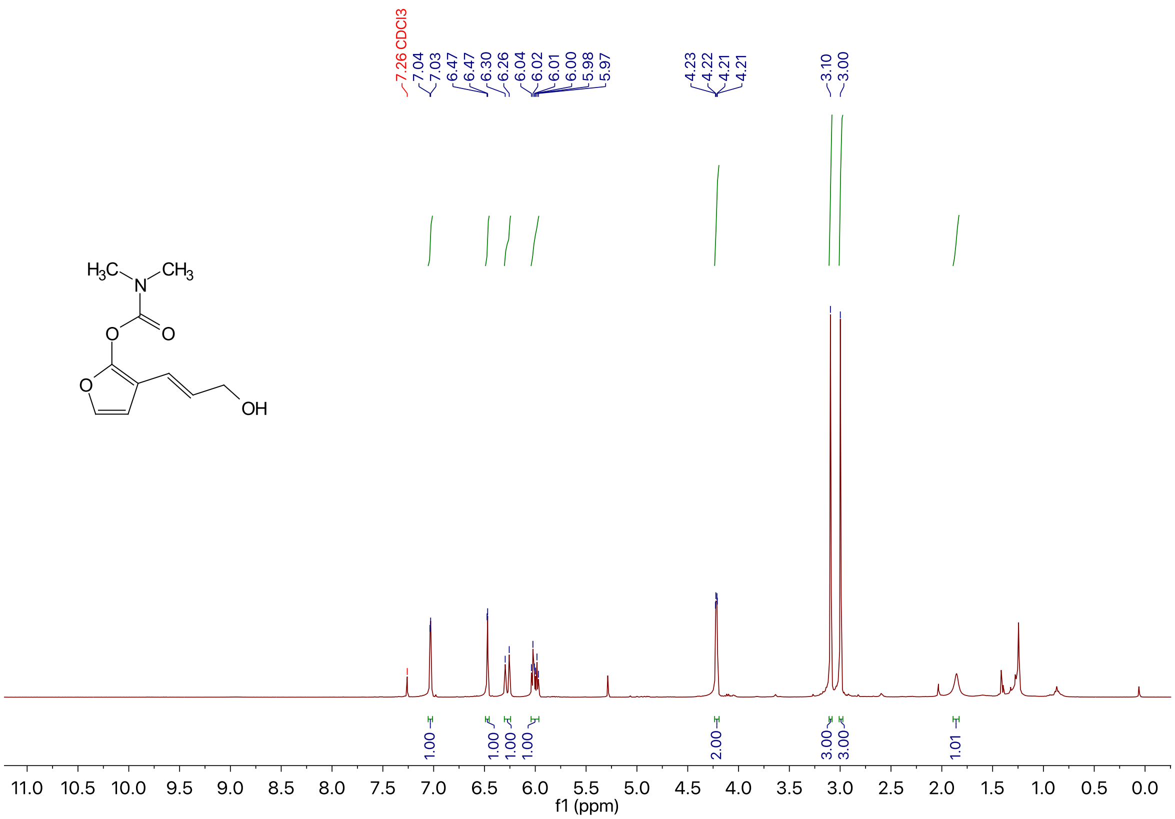

^{13}C NMR spectrum of (*E*)-3-(3-hydroxyprop-1-en-1-yl)furan-2-yl-dimethylcarbamate (8)

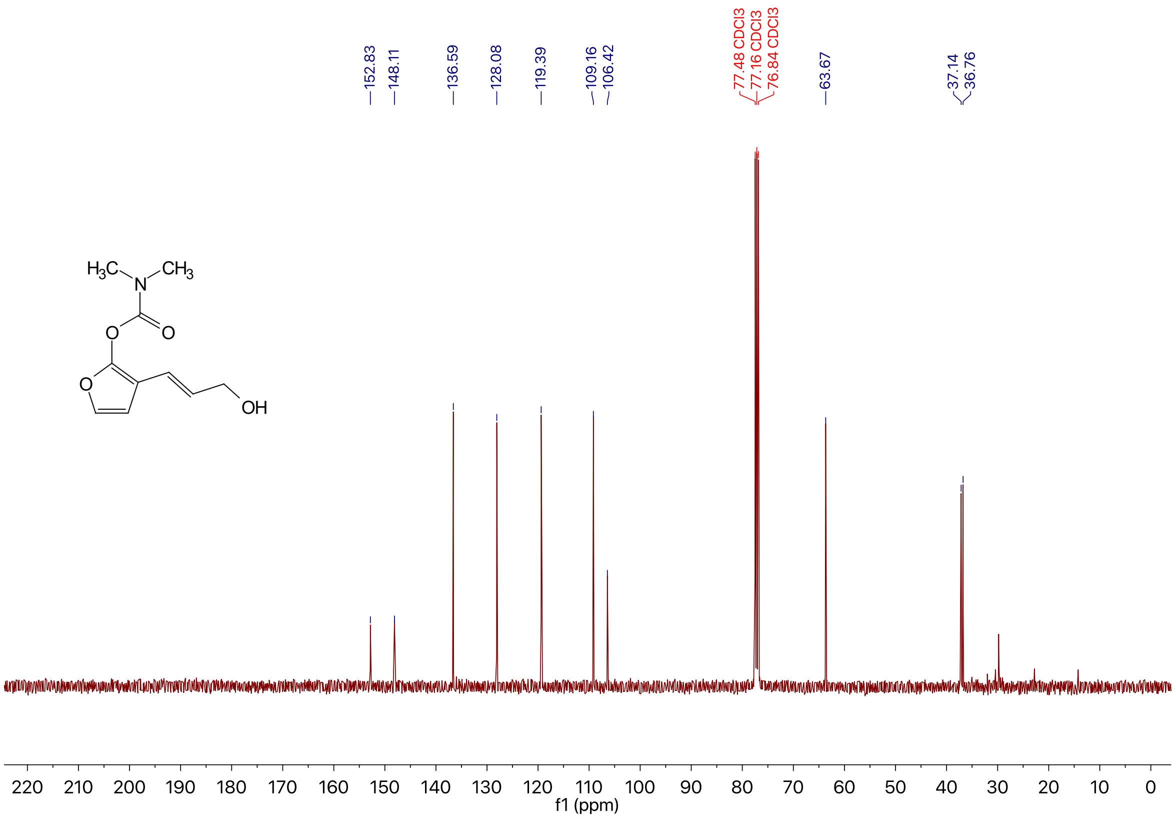

10. References

1 Fournier, J.; Lozano, O.; Menozzi, C.; Arseniyadis, S.; Cossy, J. *Angew. Chem. Int. Ed.* **2013**, *52*, 1257–1261.

2 Keck, G. E; Li, X; Knutson, C. E. *Org. Lett.* **1999**, *1*, 411–414.

3 Gronnier, C.; Kramer, S.; Odabachian, Y.; Gagosz, F. *J. Am. Chem. Soc.* **2012**, *134*, 828–831.

4 Kim, Y.; Chang, S. *Angew. Chem. Int. Ed.* **2016**, *55*, 218–222.

5 Browne D. M., Niyomura O., Wirth T. *Org. Lett.* **2007**, *9*, 3169–3171.

6 Travis B. R.; Sivakumar M.; Hollist G. O.; Borhan B. *Org. Lett.* **2003**, *5*, 1031–1034.

7 Kawamata Y., Hashimoto T., Maruoka K. *J. Am. Chem. Soc.* **2016**, *138*, 5206–5209.

8 Moradei, O. M.; Paquette, L. A. *Org. Synth.* **2003**, *80*, 66–74.

9 Mao, B.; Geurts, K.; Fañanás-Mastral, M.; van Zijl, A. W.; Fletcher, S. P.; Minnaard, A. J.; Feringa, B.L. *Org. Lett.* **2011**, *13*, 948–951.

10 Şardan M., Sezer S., Günel. A., Akkaya M., Tanyeli C, *Bioorg. Med. Chem. Lett.*, **2012**, *22*, 5814–5818.

11 Welbaneide Machado-Araujo, F.; Gore, J. *Tetrahedron Lett.* **1981**, *22*, 1969–1972.

12 Sharma, V.; Kelly, G. T.; Watanabe, C. M. H. *Org. Lett.* **2008**, *10*, 4815–4818.

13 Richard, F., Aubert, S., Katsina, T.; Reinalda L.; Palomas D.; Crespo-Otero R.; Huang J.; Leitch, D. C.; Mateos C.; Arseniyadis S. *Nat. Synth.* ***2022,*** 1, 641–648.

14 Curti, C.; Ranieri, B.; Battistini, L.; Rassu, G.; Zambrano, V.; Pelosi, G.; Casiraghi, G.; Zanardi, F. *Adv. Synth. Catal.* **2010**, *352*, 2011–2022.

15 Xie, Y.; Zhao, Y.; Qian, B.; Yang, L.; Xia, C.; Huang, H. *Angew. Chem. Int. Ed.* **2011**, *50*, 5682–5686.

16 Hao, X.-Y.; Zhou, Z.-M.; Xue, W.-Z.; Y, J.; Xu, B.-C. *Chin. J. Org. Chem.* **2008**, *28*, 1756–1760.

17 Li, B.-J.; Wu, Z.-H.; Guan, B.-T.; Sun, C.-L.; Wang, B.-Q.; Shi, Z.-J. *J. Am. Chem. Soc.* **2009**, *131*, 14656–14657.

18 Mesganaw, T.; Fine Nathel, N. F.; Garg, N. K. *Org. Lett.* **2012**, *14*, 2918–2921.

19 Quasdorf, K. W.; Antoft-Finch, A.; Liu, P.; Silberstein, A. L.; Komaromi, A.; Blackburn, T.; Ramgren, S. D.; Houk, K. N.; Snieckus, V.; Garg, N. K. *J. Am. Chem. Soc.* **2011**, *133*, 6352–6363.

20 Morin, J.; Zhao, Y.; Snieckus, V. *Org. Lett.* **2013**, *15*, 4102–4105.
